# Rationalizing (in)stability of pyridinium ketones in aqueous organic flow batteries

**DOI:** 10.1039/d6sc04767j

**Published:** 2026-09-15

**Authors:** Gabriel Gonzalez, Maxime Artault, Andrea Hamza, Mahsa Shahsavan, Jenna Hannonen, Amir Mahdian, Arsalan Hashemi, Kari Laasonen, Pekka Peljo, Petri M. Pihko

**Affiliations:** a Research Group of Battery Materials and Technologies, Department of Mechanical and Materials Engineering, University of Turku Vesilinnantie 5 Turku FI-20014 Finland; b Department of Chemistry, NanoScience Centre, University of Jyväskylä P.O.B. 35 FI-40014 JYU Finland petri.pihko@jyu.fi; c Institute of Organic Chemistry, HUN-REN Research Centre for Natural Sciences Magyar tudósok körutja 2 1117 Budapest Hungary; d Department of Chemistry and Materials Science, Aalto University P.O. Box 16100 Aalto Espoo 00076 Finland pekka.peljo@aalto.fi

## Abstract

Rationalizing the stability of organic molecules for aqueous flow battery applications is crucial for development of sustainable large scale energy storage technologies. Herein, the properties of two molecules featuring a pyridinium group next to a ketone either as an open structure (benzoylpyridinium) or as a fused ring structure (azoniafluorenone) were compared. The electrochemical investigations and flow battery experiments reveal that benzoylpyridinium electrolytes show very poor to poor stability depending on the pH while azoniafluorenones show good stability in neutral pH and poor stability in alkaline pH. The root of the problem appears to lie in the irreversible protonation of the benzylic position that takes place as the last step of the redox cycle of benzoylpyridinium electrolytes. The benzylic C–H proton of the doubly reduced molecule is readily protonated with a p*K*_a_ of 10.3, while the corresponding p*K*_a_ for the fused azoniaflurenone is only 6.0. Additionally, the computational studies indicate that the barrier for the deprotonation of the reduced form of azoniafluorenone appears to be on the order of 7–10 kcal mol^−1^ lower than that of benzoylpyridinium, and results from a structural transformation of the open benzoylpyridinium with a significantly widening dihedral angle from 6.5 to 70.5° for the two aromatic rings. This structural transformation strongly stabilizes the doubly reduced protonated species, preventing deprotonation and reoxidation in the battery, while the protonation of the reduced fused azoniafluorenone only takes place at much lower pH without significant structural changes. This structural destabilization allows reversible ketone/alcohol transition similarly to what has been observed earlier for fluorenones. The poor cycling stability under alkaline conditions results from decomposition of the oxidized species. The lessons learned in these studies strongly underline the need to consider complete redox cycles in the design of electrolytes for aqueous organic flow batteries.

## Abstract 2

Organic electrolytes for aqueous redox flow batteries must be able to complete full redox cycles. Simple changes in the molecular design, such as adding a ring fusion, can result in irreversible steps in the redox cycle.

## Graphical abstract

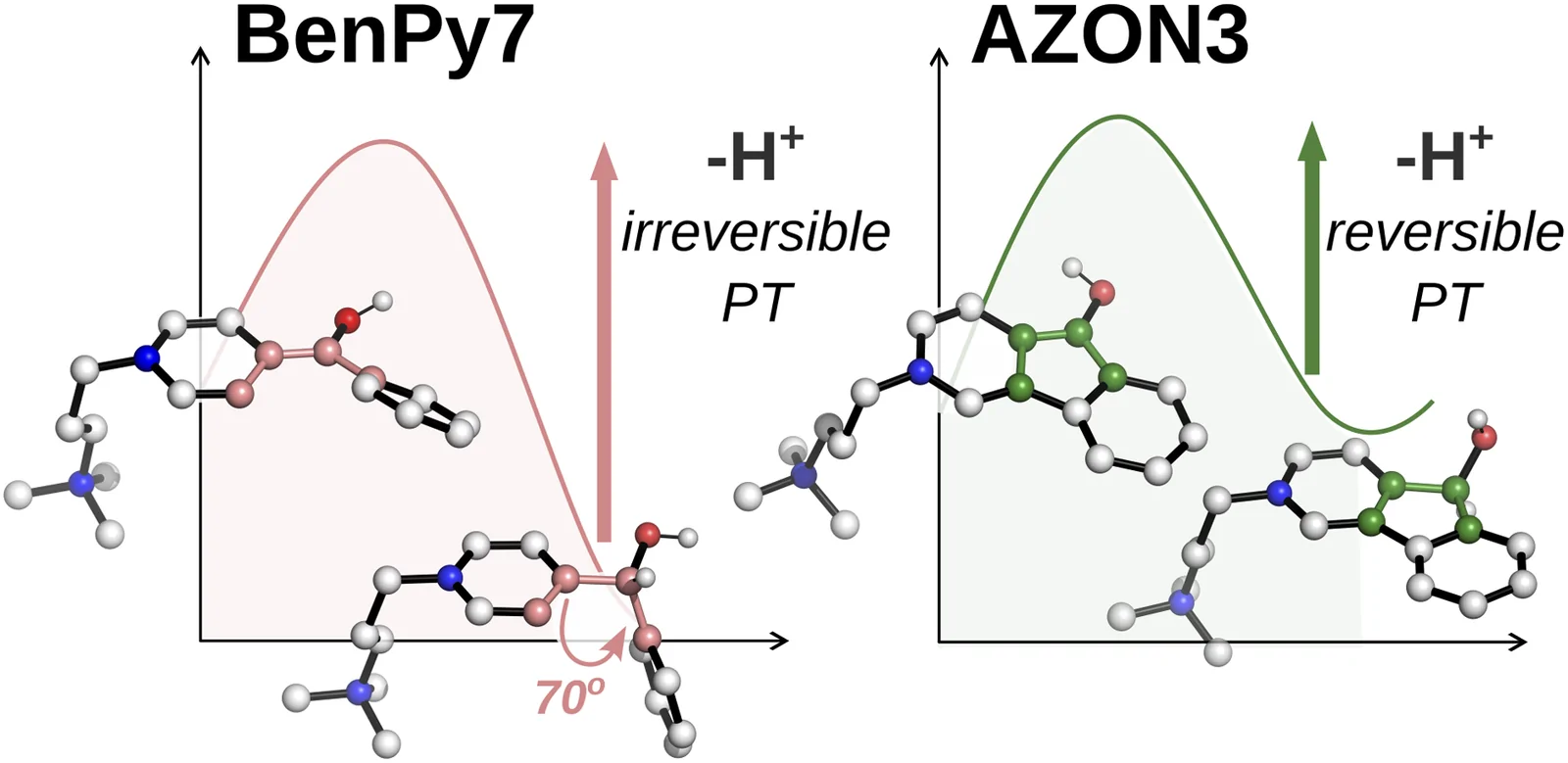

## Introduction

The high demand of mankind for energy is a challenge, particularly because our dependence on fossil fuels increases greenhouse gas emissions.^1^ Renewable energy sources, such as solar and wind are increasingly used in the energy mix, but their intermittent generation requires the use of energy storage systems to level up the energy consumption demand. Effective storage systems such as flow batteries are quite promising to overcome this intermittent nature of renewable energy sources.^2,3^ The most developed flow batteries use vanadium as the redox active material, but the volatile price and scarcity of vanadium remain the major obstacles for its adoption in large scale applications.^4^ Aqueous organic flow batteries can offer great alternatives to vanadium systems for improving cost and allowing high production and the utilization in large scale applications. Furthermore, the organic chemical diversity permits us to modulate the molecular properties to improve technical parameters such as energy and power density.^5^

Among the organic redox-active molecules, pyridinium salts have been intensively explored. Bipyridiniums such as viologen^6,7^ and diquat^8^ derivatives have been proposed as negative electrolytes (negolytes). However, examples of mono-pyridinium-based compounds are scarce. In 2017, Sanford's group reported the first example of easily accessible benzoyl pyridinium 1^+^ that is reversible for a one-electron redox process with a low redox potential (−0.85 V *vs.* Ag/AgCl 3 M) (see Fig. 1a). However, 1^+^ presented poor solubility in aqueous media, presumably due to lack of ionic/hydrophilic side chains, as well as poor charge–discharge stability in the studied aqueous electrolytes.^9^ Mono-pyridinium based aqueous organic batteries show also poor stability when utilized as pyridinium-benzoxazole functionalized polymers.^10^ Recently, our group investigated potentially more hydrophilic pyridoxal (vitamin B_6_) based pyridinium salts as redox active compounds both experimentally^11^ and computationally.^12,13^ These compounds exhibited good solubility but low stability of the reduced form in aqueous media in electrochemical and battery tests.^11^

**Fig. 1:**
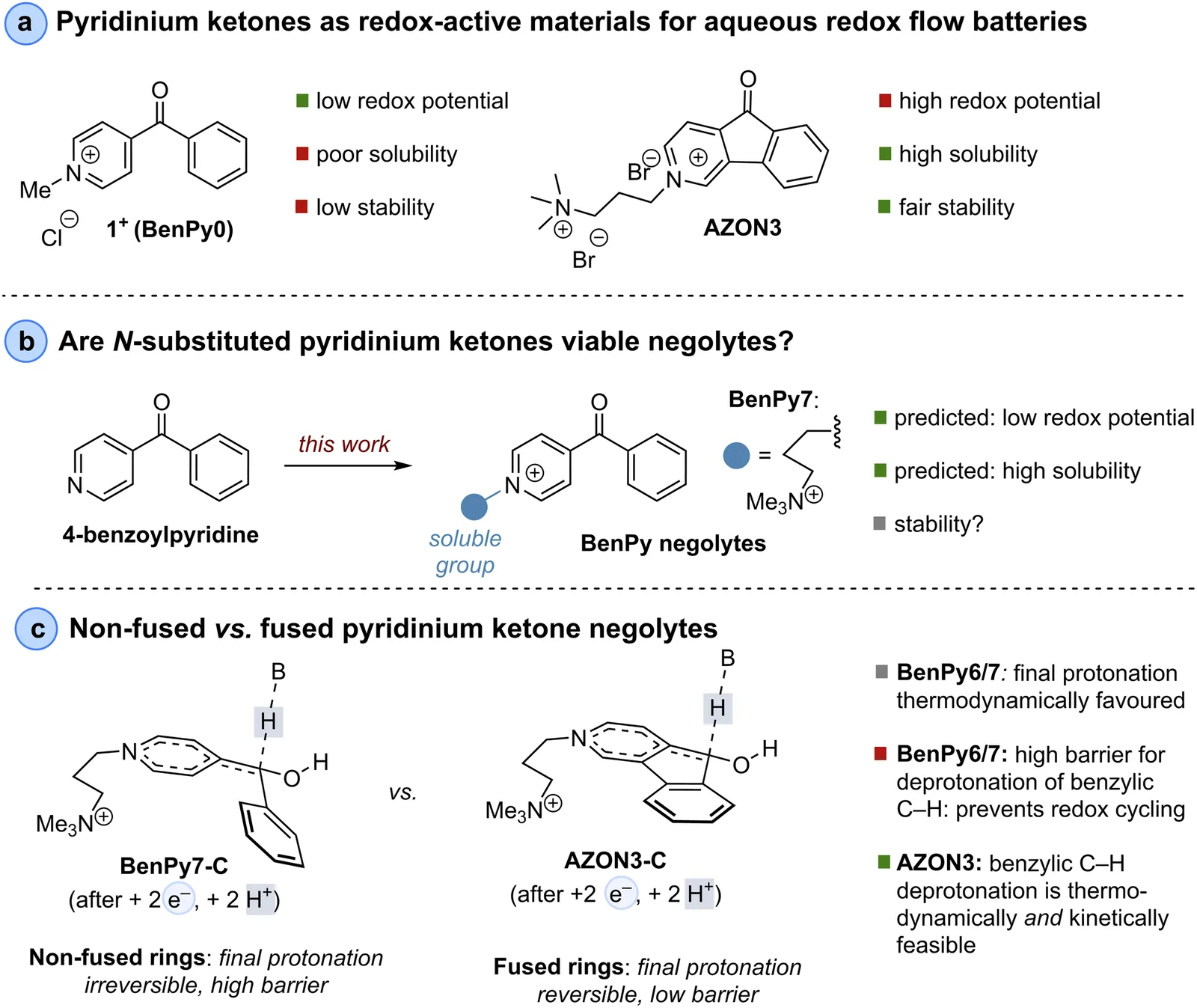
(a) Pyridiniums as potential negolytes in FBs: 1^+^ and AZON3 for AOFBs. (b) Predictions for the utility of pyridinium ketones functionalized with a solubilizing group in AOFBs. (c) Comparison of non-fused (*e.g.*BenPy7) and fused (*e.g.*AZON3) pyridinium ketone negolytes: final protonation after the two-electron reduction is irreversible with non-fused BenPy derivatives, but reversible in fused AZON3 derivatives.

Considering that increased conjugation might increase stability, in 2024, we disclosed azoniafluorenone AZON3 as the first mono-pyridinium based derivative with sufficient stability to be considered as a negolyte for AOFBs, but the relatively high redox potential of this compound (−0.31 and −0.58 V *vs.* Ag/AgCl (3 M) in a near neutral pH electrolyte) and the 4-step-synthesis should still be improved (see Fig. 1a).^14^ The related example is a dipyridinium-functionalized fluorenone, also showing decent cycling stability.^15^ Another related class of compounds are the fluorenones that do not incorporate the pyridinium ring.^16–19^ The common factor between benzoyl pyridiniums, azoniafluorenones and fluorenones is that they all undergo 2e^−^/H^+^ reduction that can be followed by additional protonation. This last protonation results in formation of a stable reduction product that cannot be easily dehydrogenated, resulting in poor battery cycling performance. For stable battery cycling, special tricks are required to either prevent the last protonation (as in the case of AZON3 described in this work) or increase the kinetics of deprotonation by functionalization in the case of fluoreneones.^16^ Additionally, reversible cycling of fluorenones has been realized with formation of hydrophobic solvation environments.^20^ Additionally, somewhat similar aza-anthraquinones have been shown to be stable in flow batteries.^21^ To answer the question why the pyridinium ketones could not be electrochemically cycled, whereas azoniafluorenones could, we decided to use the simple core of the previously mentioned benzoyl pyridinium 1^+^ and to explore variations of the *N*-substituents using hydrophilic functionalities. Herein, we demonstrate that BenPy6 and BenPy7 (Fig. 1b), including a hydroxyethyl and a quaternary ammonium functional group, respectively, have excellent solubility in water (approximately 3.5 and 2.2 M, respectively) with two quasireversible electron processes when working in alkaline media, but stability in the battery tests still remains poor. BenPy6 and especially BenPy7 are non-fused analogues of AZON3, and the ring fusion of AZON3 was found out to be the critical design feature: the irreversible protonation of the ketyl/enol form of the reduced BenPy with a p*K*_a_ of 10.2 evaluated based on the Pourbaix diagram prevented discharge of the battery, while for AZON3 the protonation is reversible, and only occurs in acidic media with a p*K*_a_ of 6.0, enabling stable battery operation in neutral pH. Computational studies with AZON3 and BenPy7 suggest that the origin of the irreversibility lies in the relatively high barrier for C–H deprotonation of the fully reduced form of BenPy7, whereas in AZON3, the corresponding C–H is deprotonated with a low barrier (Fig. 1c). Additionally, the oxidized species BenPy7 and AZON3 decompose in alkaline media.

## Results and discussion

### Synthesis of BenPy variants

We developed a robust and straightforward method to generate a wide range of pyridinium salts named BenPy. We chose 4-benzoylpyridine as the starting material because of its low cost and commercial availability, and examined the impact of the hydrophilic *N*-substituents on the properties of the pyridinium core. BenPy1–BenPy7 were readily synthesized from the parent ketone (Scheme 1). We studied the impact of –COOMe, –COOH, –OH, sulfonate, phosphonate, and quaternary amines, as anthraquinone with these functional groups have previously shown promising performance in FBs.^22^ The reaction was conducted in DMF or MeCN with various electrophiles to produce the desired pyridinium salts (from BenPy1 to BenPy7) in yields ranging from 10 to 94% (see SI Section 2). The scalability and the low-cost of this method are great advantages in this field where the AOFBs must be economically viable.

**Scheme 1:**
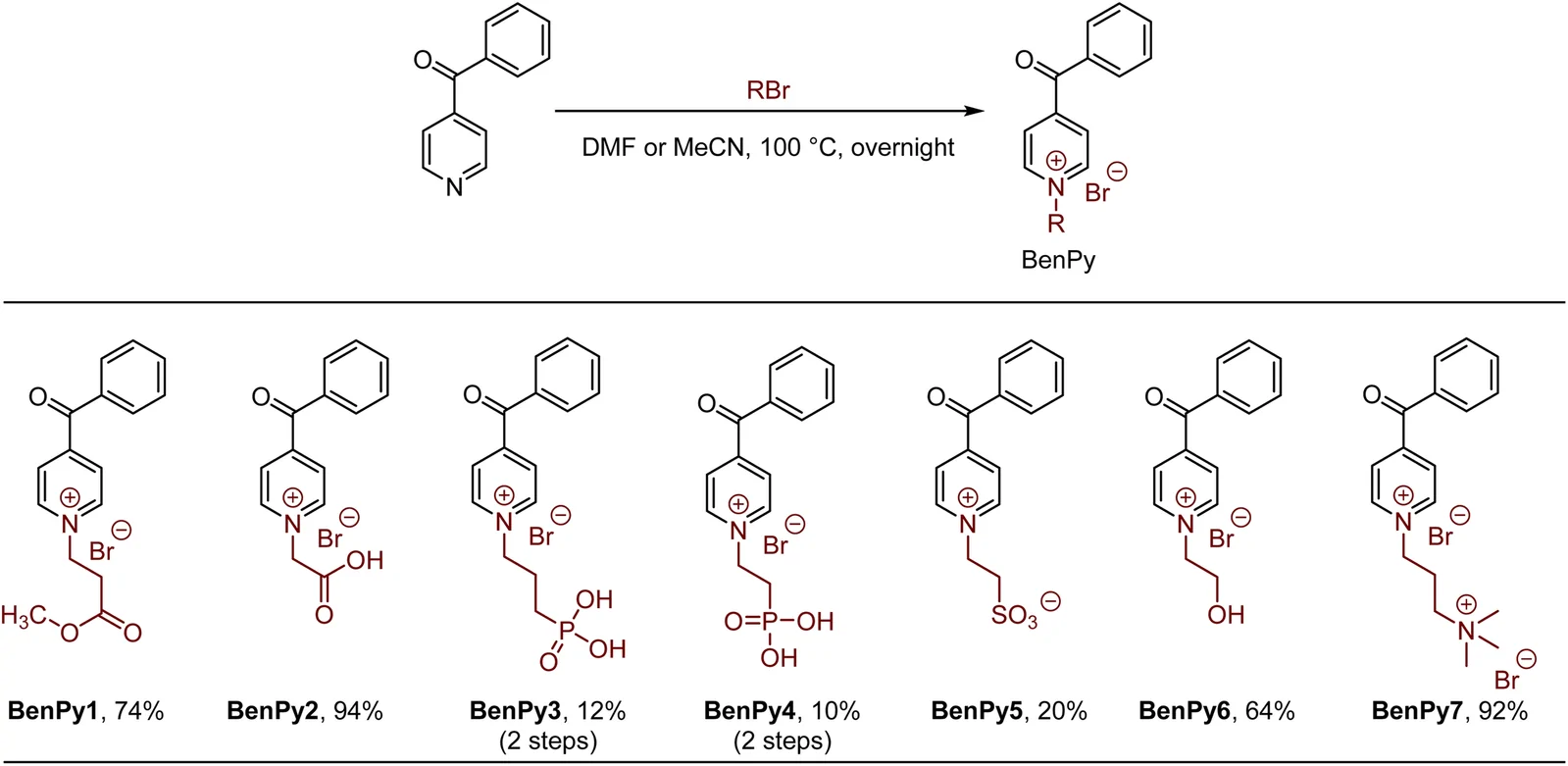
General method and scope for the synthesis of BenPy salts (see SI Section 2).

### Cyclic voltammetry and solubility measurements of BenPy derivatives

The electrochemical behaviour of BenPy1–BenPy7 was first examined using cyclic voltammetry (CV) in 1 M potassium chloride (KCl) and 0.1 M sodium hydroxide (NaOH) as supporting electrolytes (see SI Section 3 for details). When working at neutral pH, using 1 M KCl, all the studied derivatives demonstrated a single irreversible reduction peak at around −0.6 V *vs.* Ag/AgCl (3 M), indicating low stability of the species after reduction. On the other hand, in basic media, the voltammograms exhibited two separate processes at redox potentials in the range of −0.60 and −0.90 V *vs.* Ag/AgCl (3 M). It is worth noting that bromide used as the counter anion is only redox active at *ca.* 1 V *vs.* SHE,^23^ so it is not expected to affect the redox processes. Table 1 summarizes the values of the reduction peaks of BenPy1–BenPy7 in both 1 M KCl and 0.1 M NaOH and the peak separation in 0.1 M NaOH (reported against Ag/AgCl 3 M). These values varied depending on the *N*-substituents incorporated into the pyridinium structure. The negative shift of the redox potentials at higher pH suggests that the reduction involves proton-coupled electron-transfer, which was further confirmed with computational calculations (see square diagrams in SI Section 8.3). The redox potentials display shifts depending on the nature of the *N*-substituents, moving to more positive or negative values with the introduction of electron-withdrawing or -donating functional groups, respectively.^24,25^ Finally, two additional irreversible peaks are observed at redox potentials of −1.00 and −1.40 V *vs.* Ag/AgCl 3 M KCl. These last two redox processes were not further considered given their irreversibility and, in the second case, the unsuitable redox potential for aqueous systems as it overlaps with the hydrogen evolution reaction (HER). The CV results are discussed in detail in SI Section 3.

**Table 1:** Potentials and reversibility of the reduction peaks of BenPy species in 1 M KCl and 0.1 M NaOH as supporting electrolytes. Concentration of the active material: 1 mM. Scan rate: 100 mV s^−1^. Redox potentials are reported against reference Ag/AgCl (3 M KCl). Solubility in water

Compound	1 M KCl	0.1 M NaOH			Solubility
		1st peak	2nd peak	Reversibility (Δ*E*_p1_ & Δ*E*_p2_)	
BenPy1	−0.597	−0.692	−0.877	Quasi (91 & 113 mV)	Low
BenPy2	−0.555	−0.669	−0.845	Quasi (90 & 100 mV)	Low
BenPy3	−0.596	−0.700	−0.881	Quasi (85 & 111 mV)	—
BenPy4	−0.603	−0.702	−0.884	Quasi (88 & 104 mV)	—
BenPy5	−0.561	−0.646	−0.828	Quasi (75 & 98 mV)	Low
BenPy6	−0.607	−0.744	−0.902	Quasi (84 & 78 mV)	3.5 M
BenPy7	−0.576	−0.700^a^	−0.862	Quasi (73 & 78 mV)	2.2 M

a Estimated value as the 1st and 2nd peaks are overlapped.

For fast kinetics, peak separation of *ca.* 60 mV is expected for one electron processes. BenPy1 and BenPy2 showed sluggish charge transfer processes with peak separations of *ca.* 90 mV for the first reduction and 100 to 113 mV for the second reduction, and low solubility in aqueous media, while phosphonates containing BenPy3 and BenPy4 showed similar behaviour. A sulfonate containing BenPy5 displayed slightly faster electrochemical kinetics with peak separations of 75 and 98 mV for the two peaks but also low solubility. Finally, we focused on the compounds BenPy6 and BenPy7, bearing a hydroxyethyl and a quaternary ammonium functional group, respectively. In alkaline media, both compounds display two quasireversible redox processes with peak separations of *ca.* 80 mV (Fig. 2), coupled with an excellent solubility in water.

**Fig. 2:**
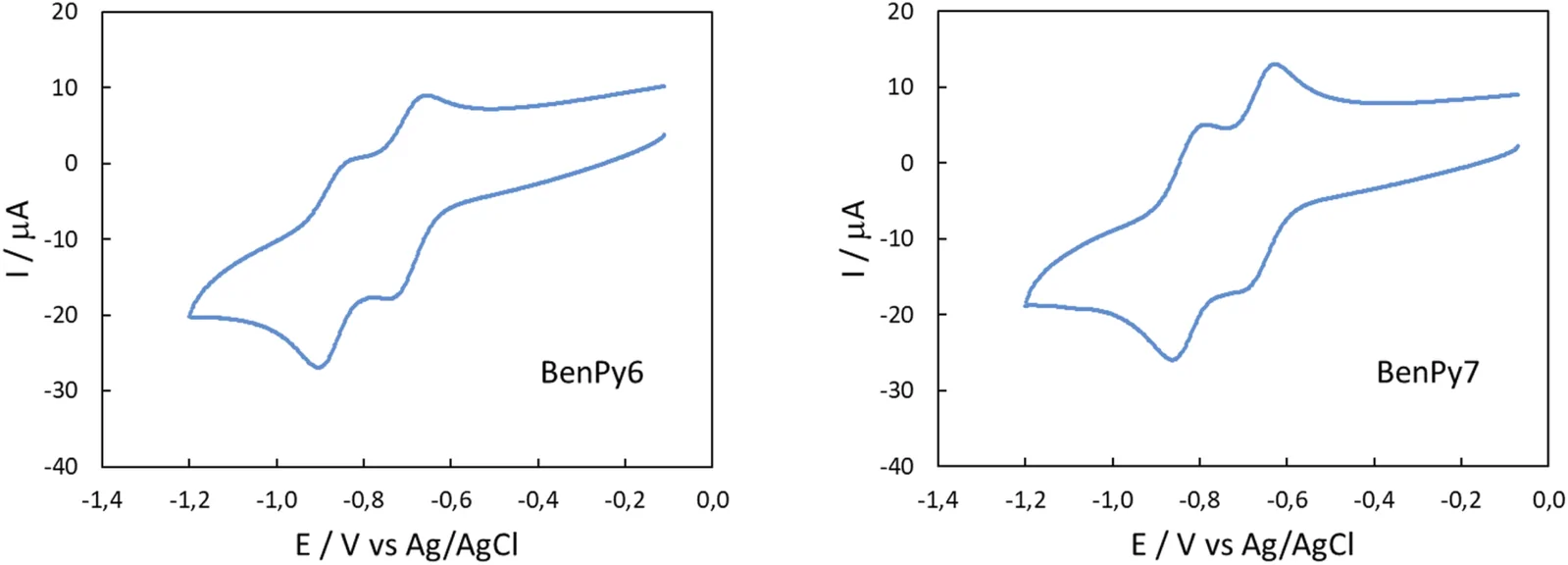
Cyclic voltammograms of BenPy6 and BenPy7 in 0.1 M NaOH as the supporting electrolyte. Concentration of the active material: 1 mM. Scan rate: 100 mV s^−1^. Redox potentials are reported against reference Ag/AgCl (3 M KCl).

The approximate maximum solubility in water was determined using UV-vis measurements (see SI Section 5) to be 3.5 M for BenPy6 and 2.2 M for BenPy7, which would lead to very high storage capacities of 188 and 118 Ah L^−1^, respectively, if we consider the utilization of the two electron processes. However, the solubility tests are based on a sample size of *N* = 1 and should therefore be treated as approximate estimations.

Rationalizing solubility is always challenging as it requires predicting both solvation energy as well as lattice energy of the ionic solid.^26^ Generally, charged groups (such as –COO^−^, –SO_3_^−^, and –NMe_3_^+^) as well as –OH groups are expected to enhance aqueous solubility, especially when the counterions are relatively small (Na^+^, K^+^, and halide ions).^27^ On this basis, the relatively high solubility of BenPy6 and BenPy7 with –(CH_2_)_2_–OH and –(CH_2_)_3_–NMe_3_^+^ functionalization was expected, and the poor solubility of BenPy2 and BenPy5 might be related to their zwitterionic character: the poor hydrogen bond donor ability of the –NMe_3_^+^ groups renders them weakly hydrated and chaotropic, and they apparently prefer to pair with the –COO^−^ and –SO_3_^−^ groups (matched weakly hydrated pair^28^). BenPy1 features a neutral –COOMe group, and its solubility was also low. Based on the high solubility and promising results obtained with BenPy6 and BenPy7, we decided to study the performance of these two derivatives in lab-scale flow batteries.

### Flow battery tests of BenPy6 and BenPy7

We next conducted flow battery experiments with a 5 cm^2^ lab-scale flow battery cell inside a nitrogen-filled glovebox to study the stability of BenPy6 and BenPy7 under alkaline conditions. No flow battery experiments were carried out under neutral conditions as the CV results show that these compounds are not stable at pH 7. The tests consisted of galvanostatic cycling at 10 mM of the BenPy derivative dissolved in different supporting electrolytes: 0.1 M and 2 M NaOH, and 1 M KCl (pH adjusted to slightly basic pH). An excess amount of posolyte (ferri/ferrocyanide) was used to ensure the negolyte as the capacity limiting side. All experiments employed a cation exchange membrane (Nafion 212) and carbon felt electrodes. Further details about the battery experiments are presented in SI Section 4. For BenPy6, in both 0.1 and 2 M NaOH, the battery was able to charge over 90% of the theoretical capacity corresponding to two electrons. However, this derivative demonstrated poor stability in 0.1 M NaOH with some improvements when working under more alkaline conditions: the battery loses half of the theoretical capacity after 4 cycles in 0.1 M NaOH with coulombic efficiency of 78% for the first cycle and stabilizing at 92% for the following cycles. In 2 M NaOH the number of cycles increased to 23 to lose the same capacity (Fig. 3). Now the coulombic efficiency remained close to 100% apart from *ca.* 90% for the first cycle. The coulombic efficiency and discharge capacity for each cycle are plotted in Section 4.1 of the SI. Interestingly, the cell voltage increases by *ca.* 150 mV when increasing the NaOH concentration from 0.1 M to 2 M, while PCET processes should shift only 59 mV per pH unit. The origin of this large shift is not clear but could be attributed to activity changes for both positive and negative electrolytes. The postmortem electrolyte analysis showed crossover of BenPy6 to the positive side, evidenced by cyclic voltammetry measurements, while UV-vis analysis did not show new bands that would indicate decomposition of this derivative in the studied media (see SI Section 6). The coulombic efficiencies indicate that the capacity loss in 0.1 M NaOH is largely due to side-reactions of the reduced state, while almost 100% coulombic efficiency in 2 M NaOH indicates a capacity loss due to degradation of the oxidized species.

**Fig. 3:**
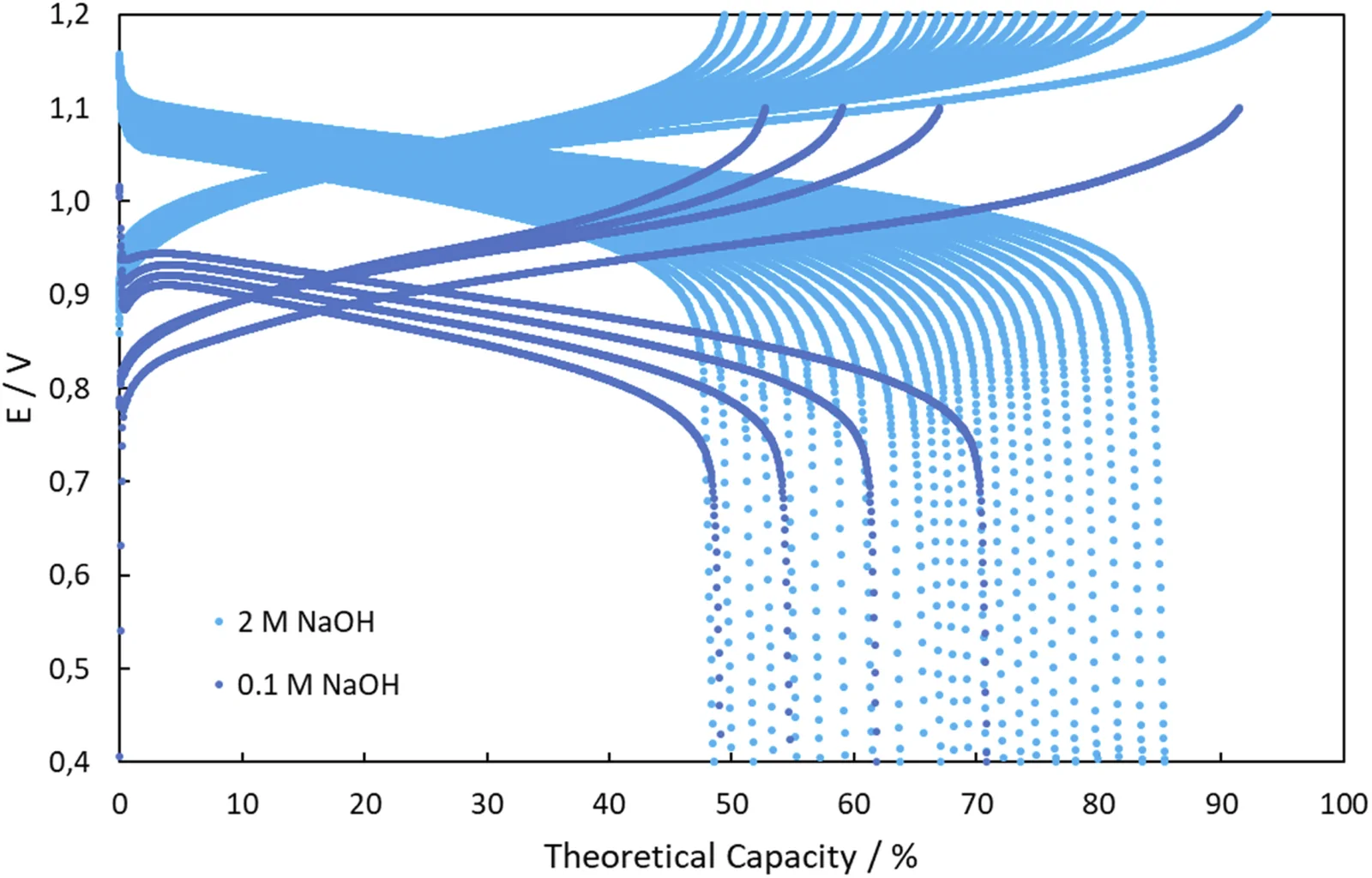
Cycling figures of the lab-scale flow battery of BenPy6 in different supporting electrolytes: 0.1 and 2 M NaOH against ferro/ferricyanide with a Nafion 212 membrane. Concentration of BenPy6: 10 mM. Theoretical capacity calculated considering a 2-electron process.

In the case of BenPy7, the batteries also demonstrated low performance under alkaline conditions. The battery was able to charge 80% of the theoretical capacity in 0.1 M NaOH (capacity corresponding to a 2-electron process), but nothing was discharged back. In 2 M NaOH only 44% of the total capacity was accessed upon charge and nothing was discharged (Fig. 4). Finally, we assembled a battery of BenPy7 under less alkaline conditions, using 1 M KCl and adjusting the pH to 9.2 after dissolving the active material. In this case, the battery was able to cycle shortly but lost 50% of the accessed capacity in just 5 cycles, with a coulombic efficiency of *ca*. 60% of the first cycle stabilizing above 95% for the following cycles. This behaviour indicates significant side reactions of the reduced species taking place at pH 9.2. In this case, the cyclic voltammograms of the electrolyte after cycling did not show crossover of the benzoylpyridinium derivative. Additionally, the cell voltage shows *ca*. 50 mV increase in the beginning of the discharge step. This kind of behaviour can originate from two effects: the resistance of the cell is reduced or the activation overpotential of the electrode reactions decreases. As the kinetics of the redox reactions seem to be relatively facile, the origin of the decreasing overpotential could be dissolution of species blocking the membrane or the electrode.

**Fig. 4:**
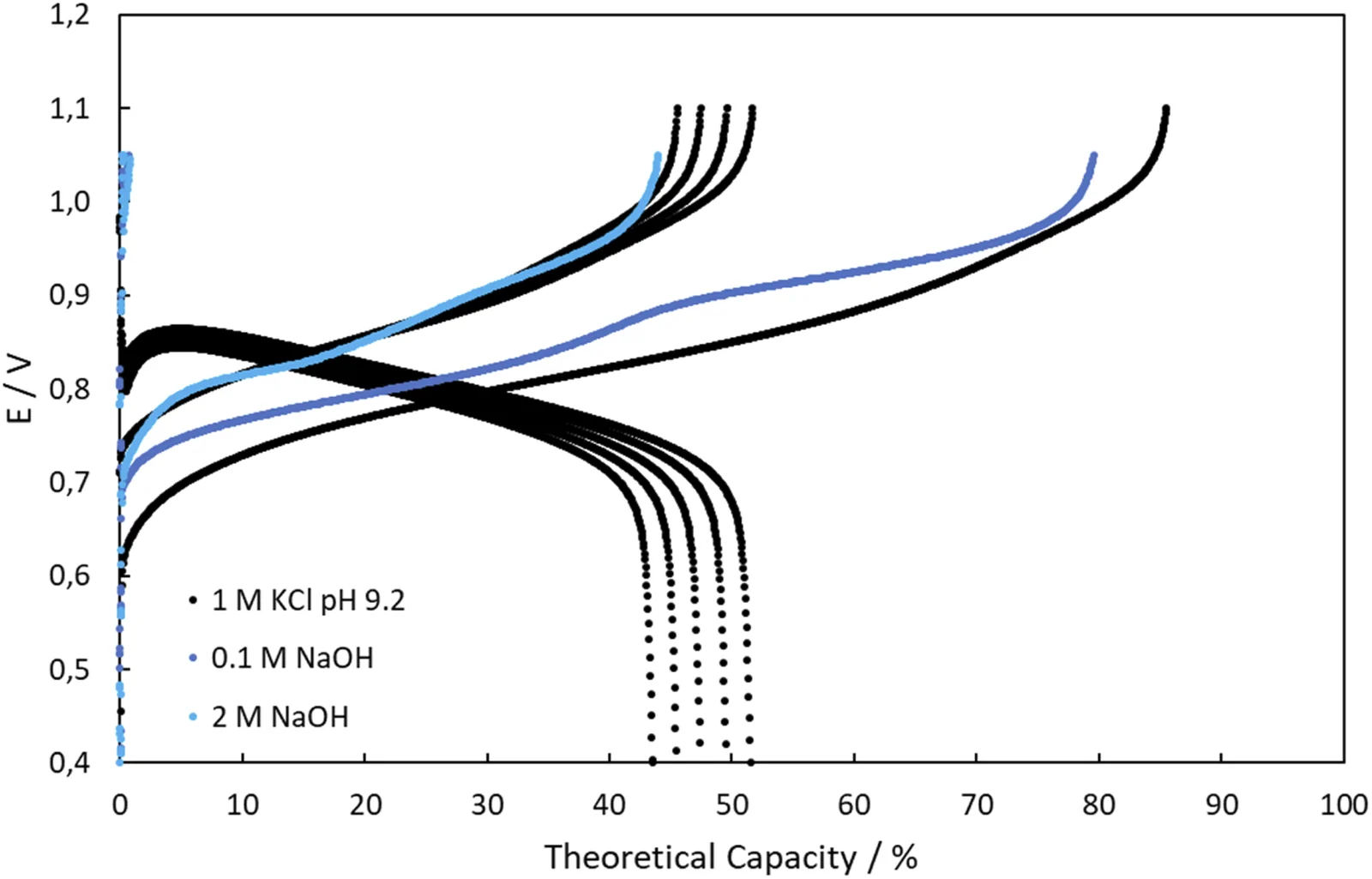
Lab-scale flow battery cycling figures of BenPy7 in different supporting electrolytes: 0.1 and 2 M NaOH and 1 M KCl (pH adjusted to 9.2) against 1,1′-bis[3-(trimethylammonio)propyl]ferrocene positive electrolyte with a Selemion DSVN membrane. Concentration of BenPy7: 10 mM. Theoretical capacity was calculated considering a 2-electron process. Five first cycles are shown.

The results discussed above demonstrate different behaviour of BenPy6 and BenPy7 as well as the corresponding azoniafluorenone derivatives AZONE3 and AZON3 reported in ref. 14. The AZON3 battery in 0.1 M NaOH showed loss of almost all capacity during the first cycle, with a coulombic efficiency of *ca.* 30% while discharging only <5% of the theoretical capacity. For cycling with a concentration of 5 mM in 1 M KCl at pH 7 against an excess amount of BTMAP-Fc as the posolyte, the battery displayed stable one electron cycling without any capacity fade.^14^ For 2-electron cycling, the capacity decay was 0.179% per cycle (3.5% per day). AZONE3 was more unstable, with capacity fade for the first electron of 0.52% per cycle in 1 M KCl.^14^

### Rationalizing the contrasting performances of BenPy6 and BenPy7*vs.*AZON3

The next step is to rationalize the contrasting performances of BenPy6 and BenPy7 as well as the corresponding azoniafluorenone derivatives AZONE3 and AZON3,^14^ including hydroxyethyl and quaternary ammonium functional groups, when tested in alkaline media. First, we consider the stability of the oxidized forms in alkaline media of 10, 100 and 2000 mM NaOH as described in SI Section 6. While BenPy6 and BenPy7 are stable in 0.01 M NaOH, both AZON derivatives decompose quite fast in this electrolyte, which evidences the lower stability of the azoniafluorenone structure in alkaline solutions. When increasing the alkalinity using 0.1 M NaOH, BenPy7 starts showing traces of decomposition after 1 hour, while BenPy6 remains stable for the whole duration of the test of 24 h, confirming the higher stability stemming from the side chain hydroxy group. Finally, 2 M NaOH seems to be too aggressive an electrolyte even for BenPy6, which slowly decomposes under these conditions. Therefore, poor cycling stability of BenPy7 can be attributed to decomposition of the oxidized species in alkaline media.

The higher stability of BenPy6 suggests a slower deprotonation process compared to BenPy7. To investigate this, we computed the p*K*_a_ values of the relevant deprotonation sites of BenPy6 and BenPy7 (see SI Section 8.5). The results confirm that BenPy7 is more easily deprotonated: at the C2 position, the p*K*_a_ values are 25.8 for BenPy6 and 8.1 for BenPy7. For BenPy6, the most favourable deprotonation site is instead the terminal OH group, with a computed p*K*_a_ of 11.1. These differences help explain the enhanced stability of the oxidized form of BenPy6 in basic electrolyte media and allows accessing most of the theoretical capacity in the first cycle when tested in a flow cell, as well as a short cycling process. However, stability issues persist in the doubly reduced state as indicated by the significant capacity decay and low coulombic efficiencies observed during the battery tests in 0.1 M NaOH (see above). As the oxidized form is stable, this poor performance must result from the reduced form. This decay is partially mitigated at higher NaOH concentrations, consistent with reduced protonation at elevated pH. Similar behaviour was previously observed for the pyridoxal derivatives.^11^

Next, we studied the effect of pH on the redox mechanism of BenPy6 experimentally by measuring the CVs in Britton–Robinson buffer across a range of pH values, complemented by computational analysis according to the literature procedures (see SI Sections 8.1 and 8.2).^29,30^ The CVs show a single peak in the pH range of 7–10 that shifts to more negative values at a rate of 30 mV per unit of pH. At higher pH, the peak shows a shoulder and splits into two processes at pH 11. The first reduction step remains unchanged, although a noticeable negative shift appears at pH 13. In contrast, the second process continues to shift to more negative potentials with increasing pH. These observations are summarized in the Pourbaix diagram (see SI Section 7). As some protonations seem to be irreversible as discussed below, this complicates the interpretation of the experimental Pourbaix diagram.

The calculated values in Scheme 2 agree well with the experimental results. The first electron transfer (ET1) at −0.661 V can be followed up by the second electron transfer (ET2) or the alternative proton-coupled electron transfer (PCET3) pathway. ET2 is thermodynamically unfavourable (−1.335 V), but PCET3 is significantly more accessible (−0.635 V at pH 7, shifts by −59 mV pH^−1^). Notably, this PCET step occurs at a potential very close to that of the first electron transfer ET1, resulting in strong thermodynamic coupling between the two processes. As a result, the intermediate formed after the first reduction is rapidly converted *via* PCET, and the overall reaction is experimentally observed as a single 2e^−^/H^+^ process. Because the PCET step involves proton transfer, the apparent redox potential shifts with pH, giving rise to the observed slope of approximately 30 mV per pH unit. This step is then followed by a protonation in PT3 with a calculated p*K*_a_ of 10.3. The predominance diagram of the doubly reduced species calculated based on the p*K*_a_ values is shown in SI Section 7.

**Scheme 2:**
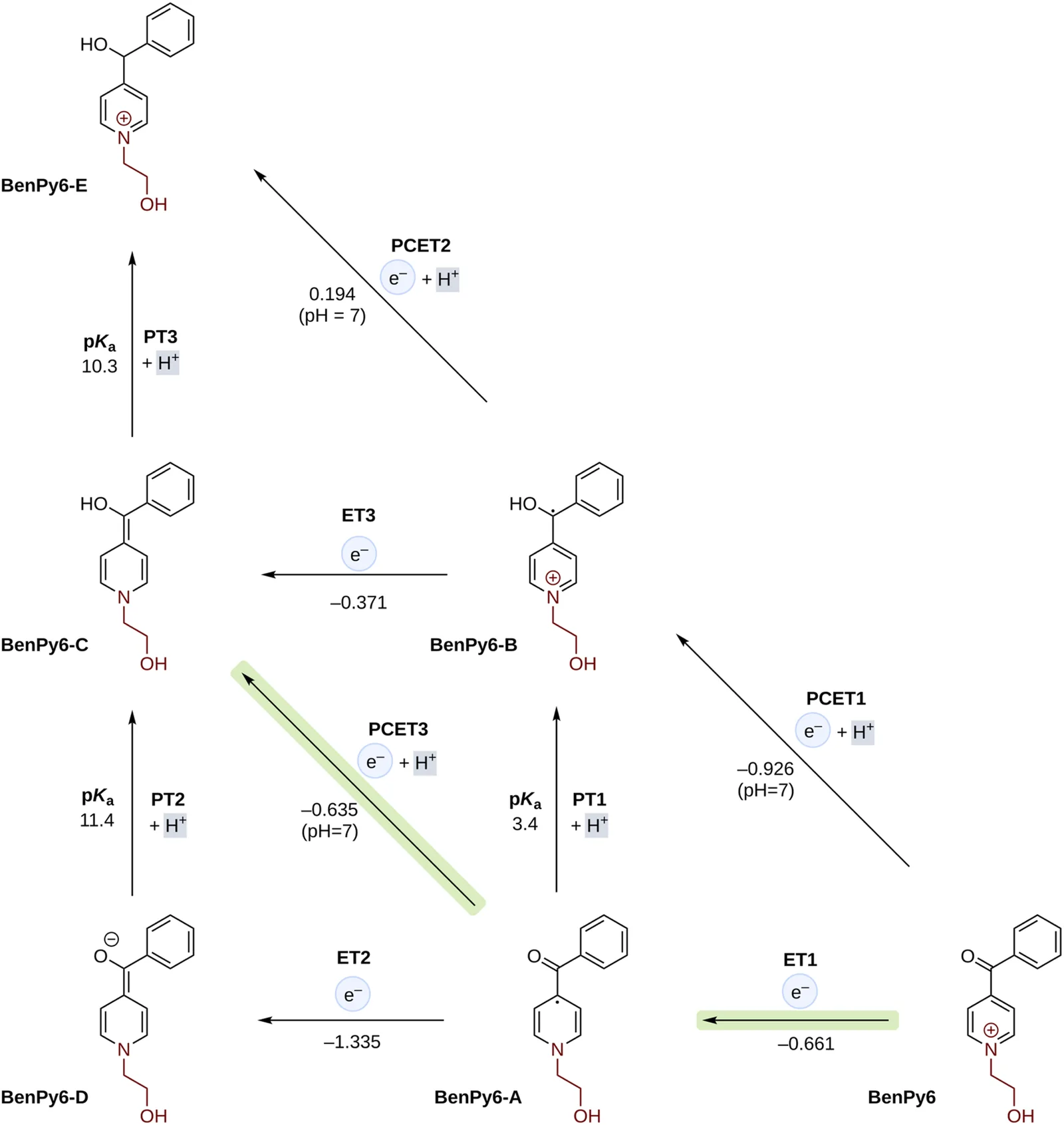
Thermodynamic square scheme representing the electron transfer (ET), proton transfer (PT) and proton-coupled electron transfer (PCET) steps for the redox-active compound BenPy6. Horizontal arrows correspond to electron transfer steps and the numbers are standard one-electron reduction potentials (in V *vs.* Ag/AgCl). Vertical arrows indicate proton transfer steps labelled with p*K*_a_ values. Diagonal arrows represent concerted PCET reactions and the numbers are effective reduction potentials (in V *vs.* Ag/AgCl). All values were computed using DFT in the solvent phase under standard conditions (*T* = 298 K, *p* = 1 atm, 1 M concentration and pH = 0). Where applicable, p*K*_a_ and PCET values were adjusted to reflect pH-dependent free energy shifts. At pH above 7, the source of protons is water.

At high pH, the PCET3 step shifts to lower potentials. As a result, the coupled 2e^−^/1H^+^ process observed at lower pH splits into two separate redox steps, with the transition around pH ∼9.5 reflecting this shift in protonation equilibria. BenPy7 shows similar behaviour with p*K*_a_ values of 10.2 and 10.9 for doubly reduced species, while AZON3 has significantly lower p*K*_a_ values of 6.0 and 10.7. Therefore, at pH levels above 6, AZON3 is not protonated after the reduction, while BenPy species are. Based on the combined electrochemical and computational results, we believe that the two-electron reduced species is finally protonated (proton transfer) to form the corresponding alcohol that cannot be oxidized back. This mechanism is illustrated in Scheme 3 and supported by calculated redox potentials and p*K*_a_ values (see SI Section S8.2). At neutral pH, this protonation is favored (p*K*_a_ ≈ 9.5 experimentally and 10.3 computationally), leading to the formation of the protonated species BenPy6-E. Its reoxidation *via* PCET2 requires a significantly higher potential (by ∼800 mV), resulting in the irreversible behavior observed in cyclic voltammetry. At higher pH values, above 11, protonation becomes less favorable, and the redox process appears reversible. However, under battery operating conditions, the reduced species is exposed for longer times, allowing slow protonation to occur and leading to capacity decay. This observation is validated when using stronger basic electrolytes, which reduce the protonation rate of BenPy6-C and increase the stability of the reduced form during the battery tests, consistent with previous studies performed for pyridoxal derivatives.

**Scheme 3:**
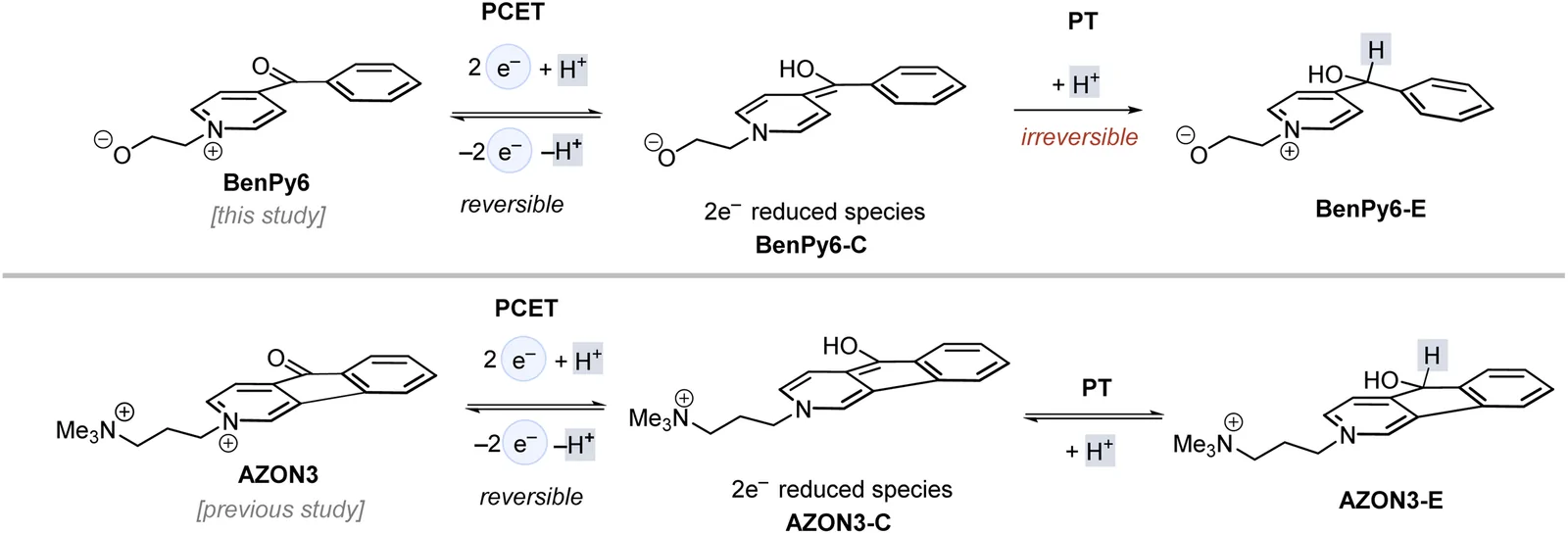
Mechanism of electrochemical reduction and protonation process of BenPy6 in aqueous electrolytes. Comparison with electrochemical reduction of AZON3. At pH above 7, the source of protons is water.

We can now explain the origin of differing stability, utilization and degradation pathways of BenPy negolytes at varied alkali concentrations. BenPy6 showed almost full capacity utilization in 0.1 M NaOH, but low coulombic efficiency. In 2 M NaOH both capacity utilization and coulombic efficiency remain high. This is now attributed to irreversible protonation of the reduced species in 0.1 M NaOH, and to hydroxide induced slow degradation of the oxidized species in 2 M NaOH. In the case of BenPy7 80% capacity utilization in 0.1 M NaOH drops to <50% in 2 M NaOH, indicating hydroxide induced degradation of the oxidized species taking place at a faster rate than for BenPy6. As the coulombic efficiency is close to 0% in 0.1 M NaOH also, irreversible protonation of reduced BenPy7 takes place at a faster rate than for BenPy6 despite the similar computational p*K*_a_ values of 10.3 and 10.2. When pH is decreased to 9.2, BenPy7 again shows high capacity utilization on the first cycle, followed by fast capacity degradation due to the irreversible protonation of the reduced species as evident by the low coulombic efficiency.

### Computational comparison of protonation of azoniafluorenone (AZON3) and benzoylpyridine (BenPy7)

To gain a deeper understanding of the factors influencing the cycling stability of these redox-active compounds, we performed an extensive comparative computational study of BenPy7 and AZON3 utilizing DFT calculations to understand why protonation is irreversible for BenPy7. These analyses focused on geometries, electronic structures, and protonation behaviour with detailed comparisons of redox state schemes, spin density distributions, geometric distortions and protonation energetics (SI Sections 8.3–8.8).

The two molecules are structurally very similar, differing only by an additional C–C bond in AZON3 that fuses the two aromatic rings, whereas BenPy7 retains a flexible benzoylpyridinium framework. Despite this relatively minor structural difference, AZON3 exhibits significantly higher cycling stability than BenPy7 when tested in a battery in a near-neutral pH electrolyte motivating a detailed computational comparison of their redox and protonation behaviour.

To understand the origin of this stability difference, we analyzed two complementary key factors: charge delocalization and protonation-induced geometric strain. While delocalization can stabilize the reduced species electronically, it is not sufficient to ensure reversible cycling on its own. Analysis of the protonation of the doubly reduced species, illustrated in Fig. 5, indicates that the ability of the molecular framework to accommodate protonation-induced structural changes plays a crucial role in both thermodynamic stability and kinetic reversibility.

**Fig. 5:**
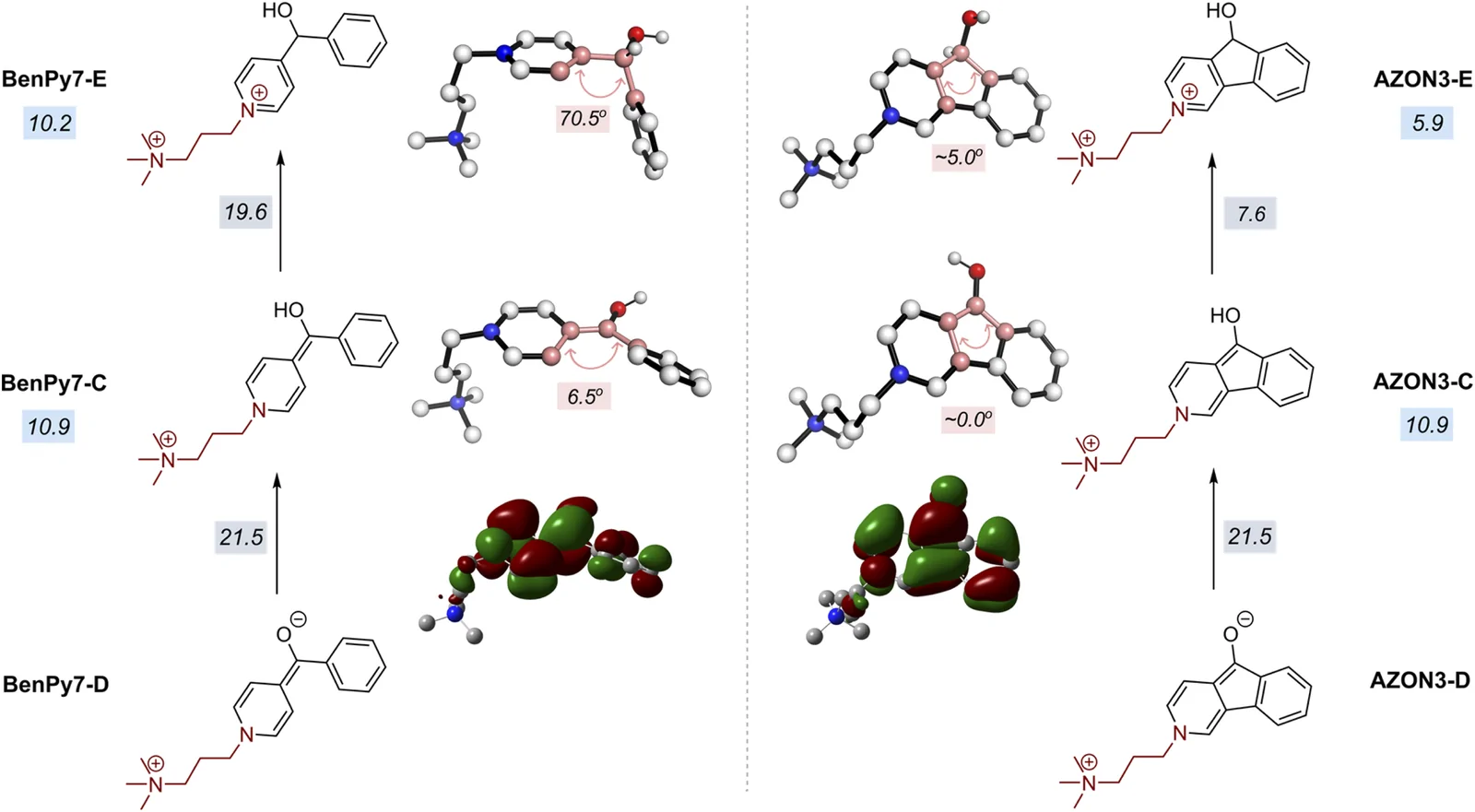
Optimized structures of doubly reduced BenPy7 and AZON3 with computed p*K*_a_ values (blue boxed numbers) and proton affinities (gray boxed values on the arrows, in kcal mol^−1^). The corresponding bending dihedral angles are indicated. The HOMOs of the one-electron protonated species are also shown.

The first protonation occurs at the carbonyl oxygen of the doubly reduced species. The resulting protonated forms are associated with a p*K*_a_ of 10.9 for both compounds and exhibit only modest geometric distortion, corresponding to relatively low strain energies of 3.9 and 2.4 kcal mol^−1^ for BenPy7-C and AZON3-C, respectively. In contrast, the second protonation occurs at the carbon atom of the carbonyl group, yielding protonated species with p*K*_a_ values of 10.2 for BenPy7-E and 5.9 for AZON3-E. In BenPy7, this step involves a change in hybridization from sp^2^ to sp^3^, causing substantial geometric distortion. The molecule adopts a bent conformation, with the two flexible rings forming an inter-ring angle of 70.5°, accompanied by a large energetic strain of 32.3 kcal mol^−1^ (for details on strain energy see Section 8.7 in the SI). In AZON3, however, the rigid framework largely preserves planarity upon second protonation. The resulting strain energy is therefore smaller, by approximately 4 kcal mol^−1^, arising mainly from a slight out-of-plane orientation of the hydroxyl group. These results illustrate that protonation becomes energetically more costly when it requires large-scale structural deformation, as observed in BenPy7, whereas the minor side-group distortion in AZON3 does not significantly hinder protonation.

Overall, while electronic delocalization can influence affinities and p*K*_a_ values, these results indicate that protonation-induced geometric strain is the dominant factor governing protonation energetics and cycling stability. In particular, the extent to which the molecular framework can accommodate structural distortion upon protonation plays a critical role. Charged BenPy7-C undergoes irreversible protonation at neutral pH, preventing discharge of the battery, while charged AZON3-C would only be protonated in acidic pH, allowing successful battery operation in the neutral pH range. As the large geometric distortion takes place only when doubly reduced BenPy species is protonated for a second time.

To understand better why the protonation is irreversible for BenPy7-C, proton-transfer reactions in solution were modelled by using formic acid as an explicit proton donor. Formic acid was chosen as it is a well-defined molecular proton donor, whereas an explicit water molecule would not adequately represent the solvent-mediated proton-transfer process in aqueous solution. This approach provides a well-defined reaction coordinate for proton transfer. To rationalize the experimentally observed irreversibility of the second protonation step, we computed the activation free energies for proton transfer to the carbonyl carbon. Fig. 6 shows the optimized transition state structures and the corresponding reaction free-energy profile for proton transfer from formic acid to the doubly reduced species.

**Fig. 6:**
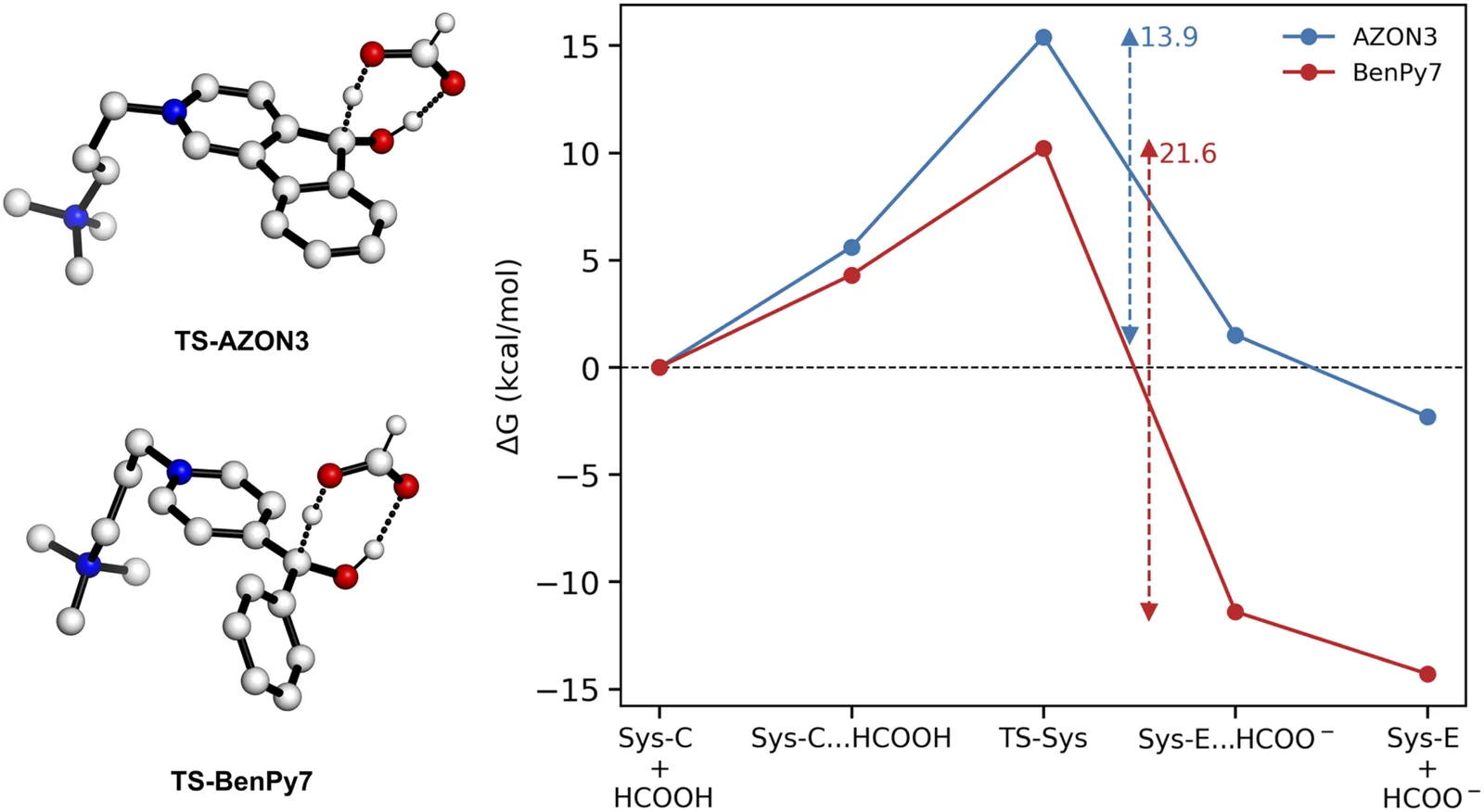
Optimized transition-state structures for AZON3 and BenPy7 (TS-AZON3 and TS-BenPy7) in the presence of formic acid, together with the corresponding reaction free-energy profiles for the protonation. In the energy diagrams, Sys refers to either AZON3 or BenPy7, and the labels Sys-C and Sys-E denote the corresponding reactant and protonated product state, respectively. The values indicated on the arrows represent the reverse activation free energies (in kcal mol^−1^).

Despite their close structural similarity, the two systems display markedly different kinetic behavior. In particular, the reverse barrier for BenPy7 is higher by approximately 7.7 kcal mol^−1^ compared to that for AZON3, indicating that once protonated, BenPy7 is significantly less prone to deprotonation. Structural analysis along the reaction coordinate shows that protonation of BenPy7-C induces pronounced pyramidalization at the carbon center along with significant backbone bending, whereas AZON3 undergoes only minor geometric rearrangement consistent with the strain analysis (see SI Sections 8.7, 8.8.1 and 8.8.2).

To assess the robustness of this trend, additional calculations were performed using a simplified model system in which the ammonium side chain was replaced by a methyl group to reduce the conformational complexity and facilitate a systematic comparison. Proton transfer was then evaluated using a series of proton donors spanning a range of acidities (CF_3_COOH, HCOOH, CH_3_COOH, and H_2_CO_3_). While the absolute barrier heights depend on the identity of the proton donor, the relative difference between BenPy7 and AZON3 remains remarkably consistent (Table S4), with BenPy7 consistently exhibiting a reverse barrier higher by approximately 7.7–10 kcal mol^−1^ (for details, see SI Section 8.8.3). This indicates that the observed kinetic preference is an intrinsic property of the molecular framework rather than an artefact of the chosen proton donor and suggests that the relative proton-transfer preference is relevant to aqueous systems.

These results highlight a key molecular design principle for pyridinium-ketone-based aqueous battery materials: increased conformational rigidity can suppress irreversible protonation of the doubly reduced species. Rigid frameworks such as AZON3 therefore provide a promising strategy to enhance cycling stability. More generally, computational evaluation of the p*K*_a_ values of the doubly reduced intermediates may serve as a useful descriptor for assessing the susceptibility of candidate molecules to protonation under battery operating conditions.

Despite the lower stability, the studied benzoylpyridinium compounds present some advantages compared to their corresponding azoniafluorenone derivatives. First, they exhibit higher solubility in aqueous solutions: BenPy7 maximum solubility was determined to be 2.2 M, compared with 1.64 M of AZON3. Furthermore, in the case of BenPy6 this value increases to 3.5 M that would lead to an outstanding volumetric capacity of 188 Ah L^−1^ if considering a stable 2-electron process. Additionally, BenPy derivatives offer improvements in terms of cell voltage, as they present more negative redox potentials than the corresponding AZON compounds. For example, BenPy7 redox processes are located at −0.70 and −0.86 V (*vs.* Ag/AgCl 3 M) compared with AZON3 redox potentials at −0.42 and −0.60 V, when considering 0.1 M NaOH as the supporting electrolyte, which leads to a cell voltage improvement of around 270 mV.

## Conclusion

This study highlights the potential of pyridinium ketone-based compounds as versatile functionalities capable of tunable redox potential and high solubility for AOFBs. However, the irreversible protonation of the ketyl/enol form of the reduced species, and the low stabilities of either the oxidized or reduced forms of pyridinium ketones suggest that more fruitful designs of AOFB materials based on pyridinium ketones should focus on conformationally more restricted analogues, such as AZON3-type compounds. Alternatively, strategies to lower the barrier for deprotonation of the reduced species for example by introducing additives or even by increasing temperature should also be considered. Another possible approach is to test successful strategies for stabilization of bipyridines such as phenyl bridging^31^ or utilization of bridged bis(bipyridines)^32^ for monopyridines as well. We also believe it is important to include stability and electrochemical tests early in the design studies of successful AOFB materials so that the entire redox cycle is considered. This work also demonstrates that computational work is crucial for rationalizing the experimental results.

## Author contributions

GG: conceptualization, data curation, formal analysis, investigation, methodology, visualization, writing – original draft, writing – review & editing. MA: conceptualization, data curation, formal analysis, investigation, methodology, visualization, writing – original draft, writing – review & editing. AnHa: conceptualization, data curation, formal analysis, investigation, methodology, validation, visualization, writing – original draft, writing – review & editing. MS: formal analysis, investigation, methodology. JH: formal analysis, investigation, methodology. AM: investigation, methodology. ArHa: investigation, methodology. KL: funding acquisition, supervision, methodology. PP: conceptualization, funding acquisition, project administration, supervision, resources, writing – original draft, writing – review & editing. PMP: conceptualization, funding acquisition, project administration, supervision, resources, writing – original draft, writing – review & editing.

## Conflicts of interest

There are no conflicts to declare.

## Supplementary Material

SC-OLF-D6SC04767J-s001

SC-OLF-D6SC04767J-s002

## Data Availability

The data supporting this article have been included as part of the supplementary information (SI): experimental details, characterization data, electrochemical and flow battery studies, stability tests, computational studies, copies of NMR spectra, and NMR FAIRDATA files. See DOI: https://doi.org/10.1039/d6sc04767j.
