# Supplementary material for: Rationalizing (in)stability of pyridinium ketones in aqueous organic flow batteries
Source: Chem Sci. 2026 Sep 15;17(43):21236–46. doi: 10.1039/d6sc04767j (PMC13629303; doi:10.1039/d6sc04767j)
Supplement: Supplementary file 1 [file SC-017-D6SC04767J-s001.pdf]

Supplementary Information (SI) for Chemical Science.
This journal is © The Royal Society of Chemistry

Supplementary Information

Rationalizing (In)stability of Pyridinium Ketones in Aqueous Organic Flow Batteries

Gabriel Gonzalez†, Maxime Artault†, Andrea Hamza, Mahsa Shahsavan, Jenna Hannonen, Amir Mahdian, Arsalan Hashemi, Kari Laasonen, Pekka Peljo* and Petri M Pihko*.

Dr. G. Gonzalez, Dr. Mahsa Shahsavan, Dr. J. Hannonen, Prof. Prof. P. Peljo
Research Group of Battery Materials and Technologies, Department of Mechanical and Materials Engineering, University of Turku, Vesilinnantie 5, Turku, FI-20014, Finland
e-mail: pekka.peljo@utu.fi

Dr. M. Artault, Prof. P. M. Pihko
Department of Chemistry and NanoScience Centre, University of Jyväskylä, P.O.B. 35, FI- 40014 JYU, Finland
e-mail: petri.pihko@jyu.fi

Dr. Andrea Hamza
Institute of Organic Chemistry, HUN-REN Research Centre for Natural Sciences, Magyar tudósok körutja 2, 1117 Budapest, Hungary

A. Mahdian, Dr. A. Hashemi, Prof. Dr. K. Laasonen, Prof. Dr. P. Peljo
Department of Chemistry and Materials Science, Aalto University, P.O. Box 16100 Aalto, Espoo, 00076 Finland

† G.G. and M.A. contributed equally to this paper.

Contents

1 General information 4
2 Synthesis of pyridinium electrolytes 5
2.1 4-Benzoyl-1-(3-methoxy-3-oxopropyl)pyridin-1-ium bromide **BenPy1** 5
2.2 4-benzoyl-1-(carboxymethyl)pyridin-1-ium bromide **BenPy2** 5
2.3 4-benzoyl-1-(3-phosphonopropyl)pyridin-1-ium bromide **BenPy3** 6
2.4 4-benzoyl-1-(2-phosphonoethyl)pyridin-1-ium bromide **BenPy4** 7
2.5 2-(4-benzoylpyridin-1-ium-1-yl)ethane-1-sulfonate **BenPy5** 7
2.6 4-benzoyl-1-(2-hydroxyethyl)pyridin-1-ium bromide **BenPy6** 8
2.7 4-benzoyl-1-(3-(trimethylammonio)propyl)pyridin-1-ium bromide **BenPy7** 8
3 Cyclic voltammetry measurements 10
3.1 **BenPy1** 11
3.2 **BenPy2** 12
3.3 **BenPy3** 12
3.4 **BenPy4** 13
3.5 **BenPy5** 14
3.6 **BenPy6** 14
3.7 **BenPy7** 15
3.8 Electrolyte post-mortem analysis after cycling 16
3.8.1 **BenPy6** battery in 0.1 M NaOH 16
3.8.2 **BenPy6** battery in 2 M NaOH 16
3.8.3 **BenPy7** battery in 1 M KCl pH 9.2 16
4 Flow battery studies 17
4.1 **BenPy6** 17
4.1.1 Battery in 0.1 M NaOH 17
4.1.2 Battery in 2 M NaOH 18
4.2 **BenPy7** 19
4.2.1 Battery in 0.1 M NaOH 19
4.2.2 Battery in 2 M NaOH 20
4.2.3 Battery in 1 M KCl pH 9.2 21
5 Maximum solubility measurements 23

5.1 **BenPy6** ... 23
5.2 **BenPy7** ... 24
6 Stability tests - UV-Vis and NMR measurements ... 25
6.1 **BenPy6** ... 26
6.1.1 Battery in 0.1 M NaOH ... 27
6.1.2 Battery in 2 M NaOH ... 27
6.2 **BenPy7** ... 28
6.2.1 Battery in 0.1 M NaOH ... 28
6.2.2 Battery in 2 M NaOH ... 29
6.2.3 Battery in 1 M KCl pH 9.2 ... 30
6.2.4 NMR Measurements in 0.1 M NaOD ... 31
7 Pourbaix diagram and predominance diagram ... 33
8 Computational studies ... 36
8.1 Methodology ... 36
8.2 Electrochemical square representation and thermodynamic considerations ... 36
8.3 Square diagrams for **AZON3** and **BenPy7** ... 39
8.4 Thermodynamic cycles ... 40
8.5 Deprotonation sites for **BenPy6** and **BenPy7** ... 41
8.6 Spin density analysis ... 43
8.7 Energetic strain analysis upon protonation-induced geometric distortion ... 44
8.7.1 First protonation ... 45
8.7.2 Second protonation ... 45
8.8 Model calculations for the second protonation ... 47
8.8.1 Protonation of model systems **AZON3_Me** and **BenPy7_Me** ... 47
8.8.2 TS structural evolution ... 49
8.8.3 Proton donor dependence ... 51
8.9 Energy data for the calculated structures ... 53
8.10 Cartesian coordinates of the calculated structures ... 55
9 References ... 101

1 General information

All reactions were carried out under an argon atmosphere in flame-dried glassware, unless otherwise noted. Toluene was obtained by passing deoxygenated solvents through activated alumina columns (MBraun SPS-800 Series solvent purification system). Other solvents and reagents were used as obtained from supplier, unless otherwise noted. Analytical TLC was performed using Merck silica gel F254 (230-400 mesh) plates and analysed by UV light. For silica gel chromatography, the flash chromatography technique was used, with Merck silica gel 60 (230-400 mesh) and p.a. grade solvents unless otherwise noted.

The ^{1}H NMR, $^{13}C\{^{1}H\}$ NMR and ^{31}P spectra were recorded in $CDCl_3$, D_2O or DMSO on Bruker Avance 500 or 300 spectrometers. The chemical shifts are reported in ppm relative to $CHCl_3$ (δ 7.26), D_2O (4.79) or DMSO (2.50) for ^{1}H NMR. For the ^{13}C NMR spectra, $CDCl_3$ (δ 77.16), or DMSO (39.51) were used as the internal standards. Melting points (Mp) were determined in open capillaries using Gallenkamp melting point apparatus. IR spectra were recorded on a Tensor27 FT-IR spectrometer. High resolution mass spectrometric data were measured using 6200 series TOF/6500 series Q-TOF B.09.00.

2 Synthesis of pyridinium electrolytes

2.1 4-Benzoyl-1-(3-methoxy-3-oxopropyl)pyridin-1-ium bromide BenPy1

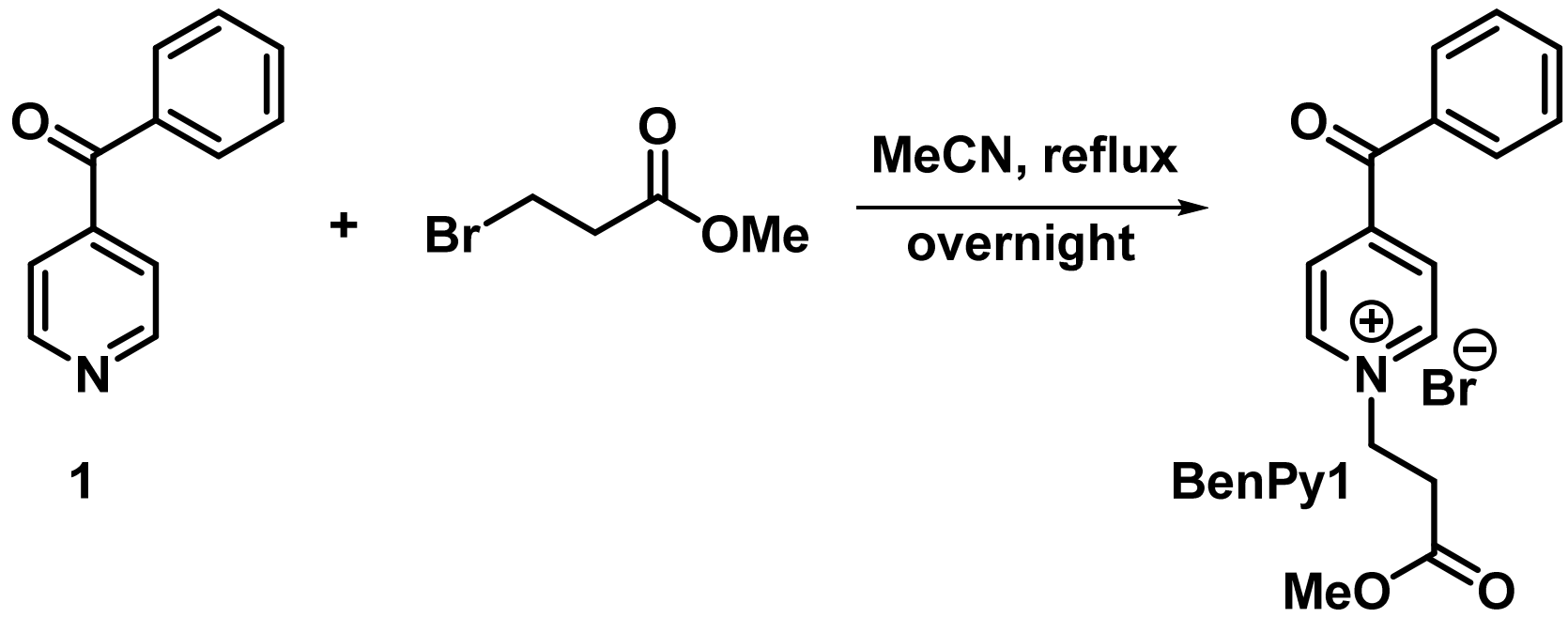

To a solution of **1** (0.400 g, 2.18 mmol, 1 equiv) in MeCN (1.5 mL) was added Methyl 3-bromopropionate (0.435 g, 2.61 mmol, 1.2 equiv) at rt in one portion. The reaction mixture was stirred at reflux overnight. The mixture was concentrated under reduced pressure. Purification of the residue via CombiFlash (0% to 10% DCM/MeOH) afforded the desired product **BenPy1** as white solid (0.568 g, 74%). **^{1}H NMR** (300 MHz, $CDCl_3$) *δ* 9.87 (d, *J* = 6.4 Hz, 2H), 8.18 (d, *J* = 6.6 Hz, 2H), 7.86 (d, *J* = 7.2 Hz, 2H), 7.71 (t, *J* = 7.4 Hz, 1H), 7.56 (t, *J* = 7.7 Hz, 2H), 5.43 (t, *J* = 5.9 Hz, 2H), 3.65 (s, 3H), 3.44 (t, *J* = 5.9 Hz, 2H); **^{13}C NMR** (75 MHz, $CDCl_3$) δ 191.2, 170.8, 151.8, 147.2, 135.1 (2 CH), 133.8, 130.4 (2 CH), 129.3 (2 CH), 127.0 (2 CH), 57.7, 52.4, 35.0; **IR** (film, cm^{-1}): 3250, 1723, 1668, 1313, 795; **mp** 173 – 176 °C; **HRMS** (ESI^{+}): m/z calculated for $[C_{16}H_{16}NO_3]^{+}$ = 270.1130 found 270.1144, Δ = 5.2 ppm.

2.2 4-benzoyl-1-(carboxymethyl)pyridin-1-ium bromide BenPy2

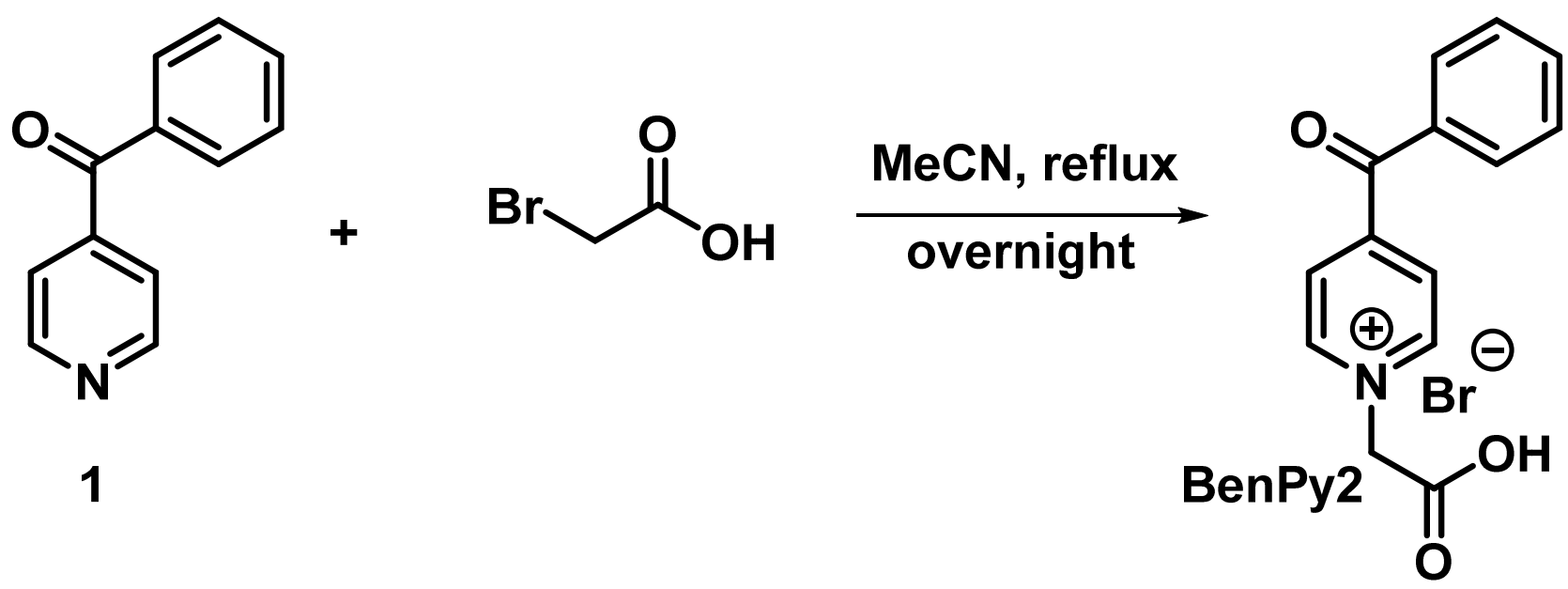

To a solution of pyridyl ketone **1** (0.500 g, 2.73 mmol, 1 equiv) in MeCN (3.0 mL) was added 2-bromoacetic acid (0.758 g, 5.46 mmol, 2 equiv) at rt. The resulting yellow solution was heated to reflux and refluxed overnight. The mixture was cooled to rt and concentrated under reduced pressure. Purification of the residue via recrystallization (from EtOH with drops of H_2O) afforded the desired product **BenPy2** as yellow solid (0.831 g, 94%). **^{1}H NMR** (500 MHz, D_2O) δ 9.07 (d, *J* = 6.2 Hz, 2H), 8.37

(d, J = 6.2 Hz, 2H), 7.89 (d, J = 7.6 Hz, 2H), 7.82 (t, J = 7.4 Hz, 1H), 7.64 (t, J = 7.7 Hz, 2H), 5.57 (s, 2H); **^{13}C NMR** (126 MHz, D_2O) δ 193.9, 169.0, 152.2, 146.7 (2 CH), 135.4, 133.8, 130.5 (2 CH), 129.1 (2 CH), 127.5 (2 CH), 62.1; **IR** (film, cm^{-1}): 2787, 1736, 1445, 1396, 1194, 937, 711, 683; **mp** 217 – 220 °C; **HRMS** (ESI+): m/z calcd for $[C_{14}H_{12}NO_3]^+$ 242.0817, found 242.0806, Δ = - 4.5 ppm.

2.3 4-benzoyl-1-(3-phosphonopropyl)pyridin-1-ium bromide BenPy3

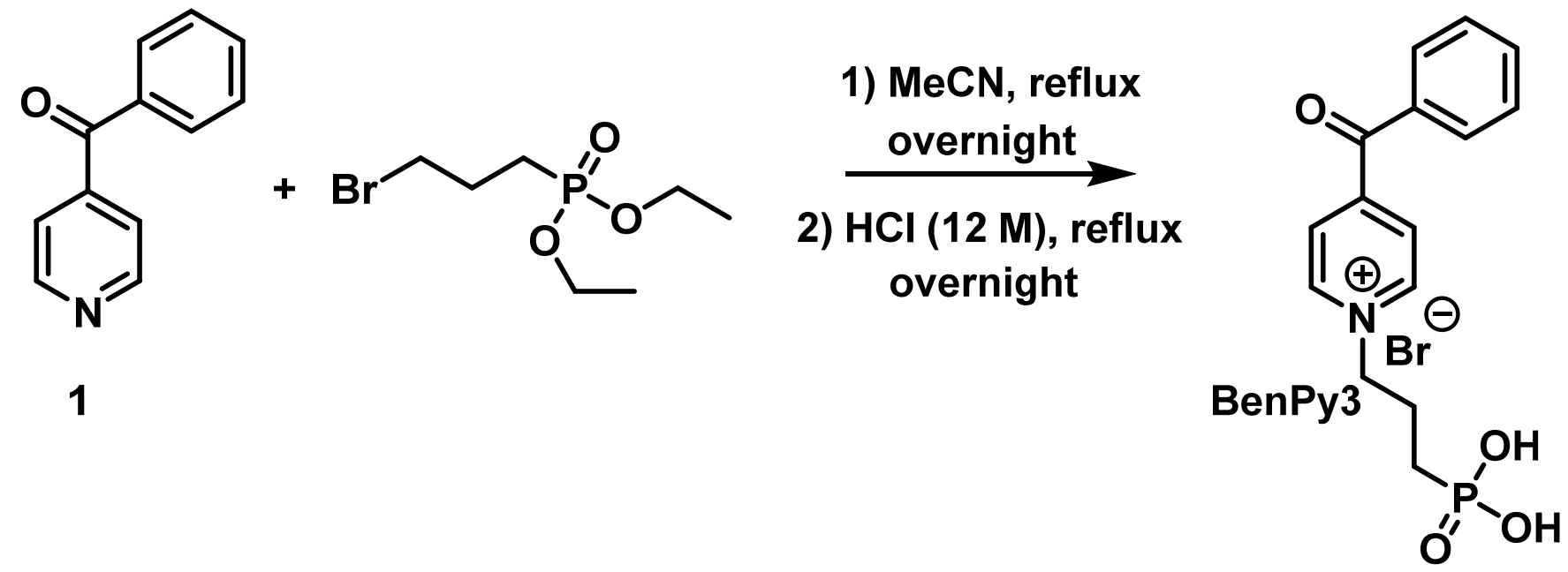

To a solution of pyridyl ketone **1** (0.400 g, 2.18 mmol, 1 equiv) in MeCN (1.5 mL) was added diethyl (3-bromopropyl)phosphonate (0.676 g, 2.61 mmol, 1.2 equiv) at rt. The reaction mixture was heated to reflux and refluxed overnight. After cooling to rt, the resulting mixture was concentrated under reduced pressure. The residue was dissolved in 2 mL of aq HCl (12 M) and the resulting solution was heated to reflux and refluxed overnight. After cooling to rt, the solution was concentrated under reduced pressure. Purification of the residue via recrystallization (EtOH and drops of H_2O) afforded the desired product **BenPy3** as yellow solid (0.100 g, 12%). **^{1}H NMR** (300 MHz, DMSO) δ 9.30 (d, J = 6.8 Hz, 2H), 8.37 (d, J = 6.7 Hz, 2H), 7.91 – 7.85 (m, 2H), 7.81 (t, J = 7.5 Hz, 1H), 7.64 (t, J = 7.6 Hz, 2H), 4.77 (t, J = 7.1 Hz, 2H), 2.20 (m, 2H), 1.73 – 1.58 (m, 2H); **^{13}C NMR** (126 MHz, DMSO) δ 192.7, 152.1, 146.3 (2 CH), 135.3, 130.8, 129.6 (2 CH), 127.5 (2 CH), 61.7 (d, J = 17 Hz), 25.8, 24.6 (d, J = 137 Hz); **^{31}P NMR** (122 MHz, DMSO) δ 25.2; **IR** (film, cm^{-1}): 3012, 1648, 1450, 1323, 1248, 844, 693; **mp** 195 – 198 °C; **HRMS** (ESI+): m/z calcd for $[C_{15}H_{17}NO_4P]^+$ 306.0890, found 306.0878, Δ = - 3.9 ppm.

2.4 4-benzoyl-1-(2-phosphonoethyl)pyridin-1-ium bromide BenPy4

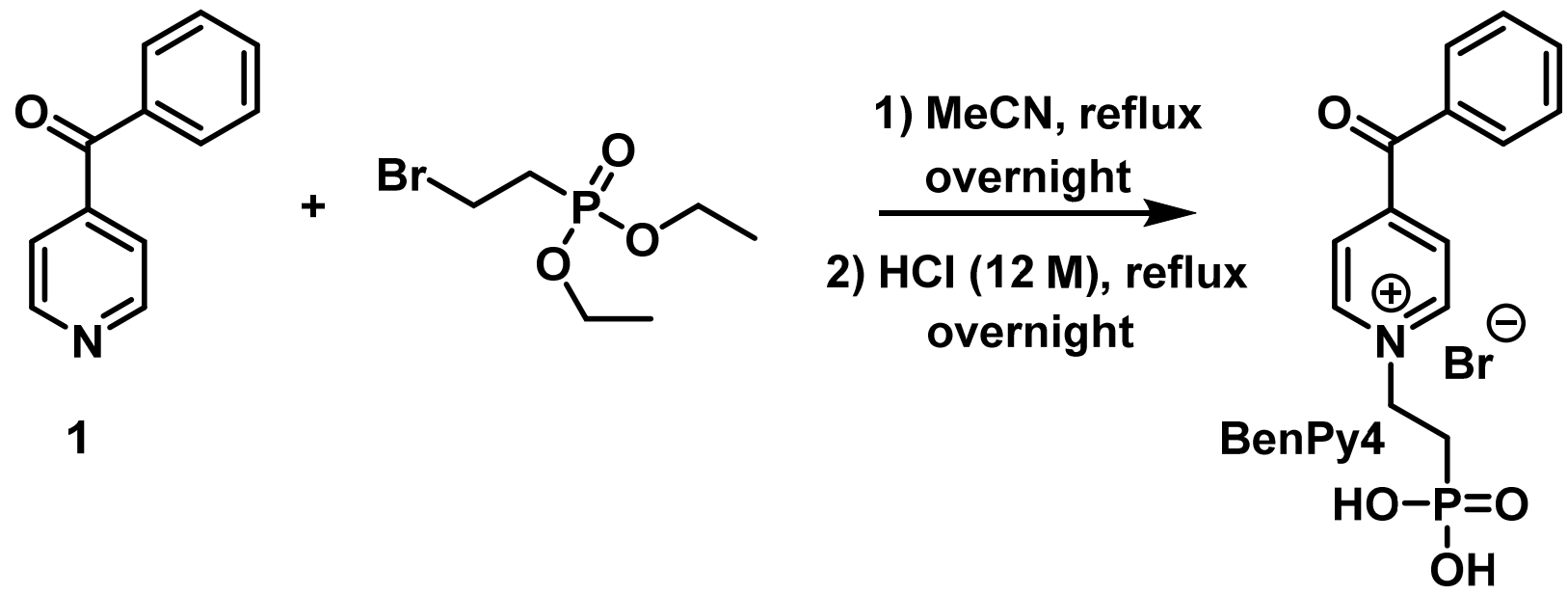

To a solution of compound **1** (0.400 g, 2.18 mmol, 1 equiv) in MeCN (1.5 mL) was added Diethyl (2-bromoethyl)phosphonate (0.642 g, 2.61 mmol, 1.2 equiv) at rt. The reaction mixture was stirred at reflux overnight. The mixture was concentrated under reduced pressure. The crude was dissolved in 2 mL of HCl (12 M) and the mixture was heated to reflux and refluxed overnight. After cooling to rt, the mixture was concentrated under reduced pressure. Purification of the residue by recrystallization (from EtOH with drops of H_2O) afforded the desired product **BenPy4** as yellow solid (0.080 g, 10% 2 steps). **^{1}H NMR** (500 MHz, D_2O) δ 9.17 (d, J = 6.3 Hz, 2H), 8.35 (d, J = 6.1 Hz, 2H), 7.89 (d, J = 7.6 Hz, 2H), 7.83 (t, J = 7.5 Hz, 1H), 7.65 (t, J = 7.7 Hz, 2H), 4.99 – 4.90 (m, 2H), 2.44 (dt, J = 16.7, 7.9 Hz, 2H); **^{13}C NMR** (126 MHz, D_2O) δ 194.1, 151.8, 145.5 (2 CH), 135.3, 133.9, 130.5 (2 CH), 129.1 (2 CH), 127.7 (2 CH), 58.0, 29.7 (d, J = 131.8 Hz); **^{31}P NMR** (122 MHz, DMSO) δ 26.1; **IR** (film, cm^{-1}): 3006, 1650, 1447, 1334, 1244, 810, 693; **mp** 180 – 183 °C; **HRMS** (ESI^+): m/z calcd for $[C_{14}H_{15}NO_4P]^+$ 292.0733, found 292.0720, Δ = - 4.4ppm.

2.5 2-(4-benzoylpyridin-1-ium-1-yl)ethane-1-sulfonate BenPy5

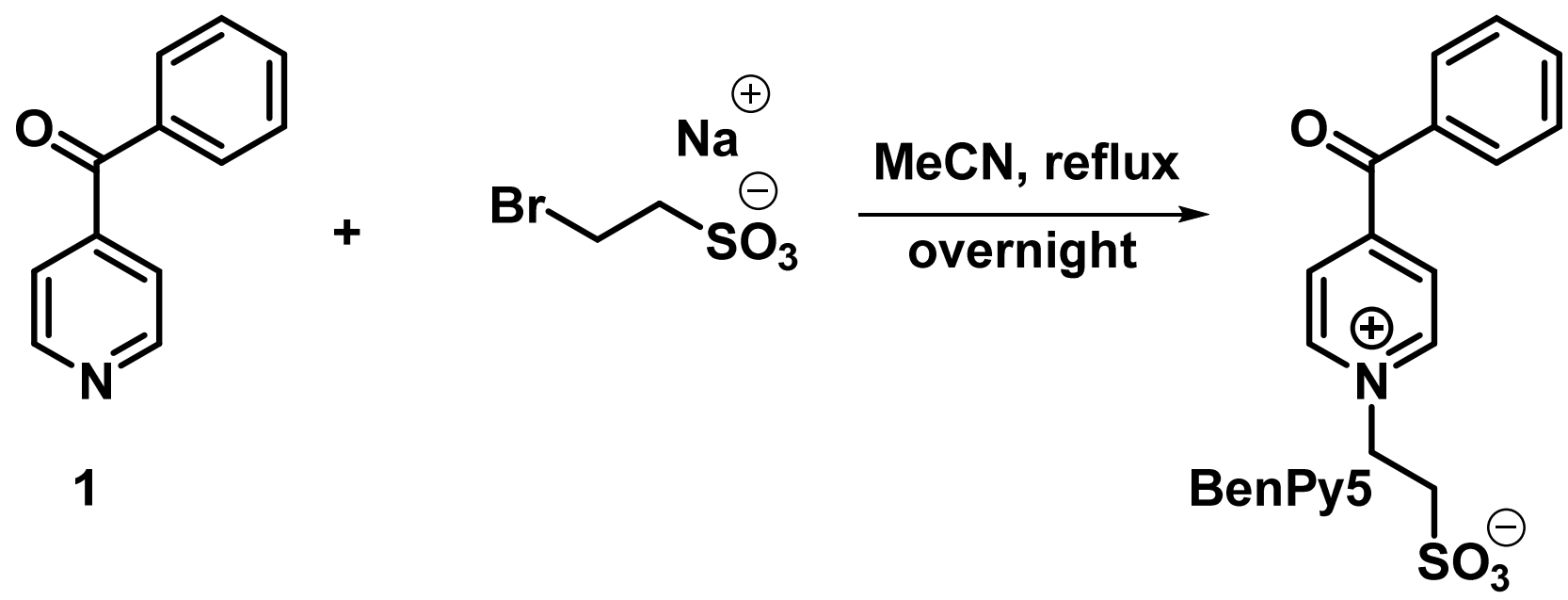

To a solution of compound **1** (0.500 g, 2.73 mmol, 1 equiv) in H_2O (1.5 mL) was added Sodium 2-bromoethane-1-sulfonate (0.865 g, 4.09 mmol, 1.5 equiv) at rt. The reaction mixture was stirred at reflux overnight. The mixture was concentrated under reduced pressure. Purification of the residue via recrystallization (from EtOH and drops of H_2O) afforded the desired product **BenPy5** as white solid (0.162 g, 20%). **^{1}H NMR** (300 MHz, D_2O) δ 9.11 (d, J = 6.8 Hz, 2H), 8.28 (d, J = 6.7 Hz, 2H), 7.85 – 7.70 (m, 3H), 7.58 (t, J = 7.8 Hz, 2H), 5.13 – 5.03 (m, 2H), 3.66 – 3.56 (m, 2H); **^{13}C NMR** (75

MHz, D_2O) δ 194.0, 152.3, 146.1 (2 CH), 135.4, 133.9, 130.6 (2 CH), 129.2 (2 CH), 127.6 (2 CH), 57.7, 50.2; **IR** (film, cm^{-1}): 3056, 1666, 1464, 1324, 1286, 946, 867, 748; **mp** 300 – 303 °C; **HRMS** (ESI+): m/z calcd for $[C_{14}H_{14}NO_4S]^+$ 292.0598, found 292.0625, Δ = 9.2 ppm.

2.6 4-benzoyl-1-(2-hydroxyethyl)pyridin-1-ium bromide BenPy6

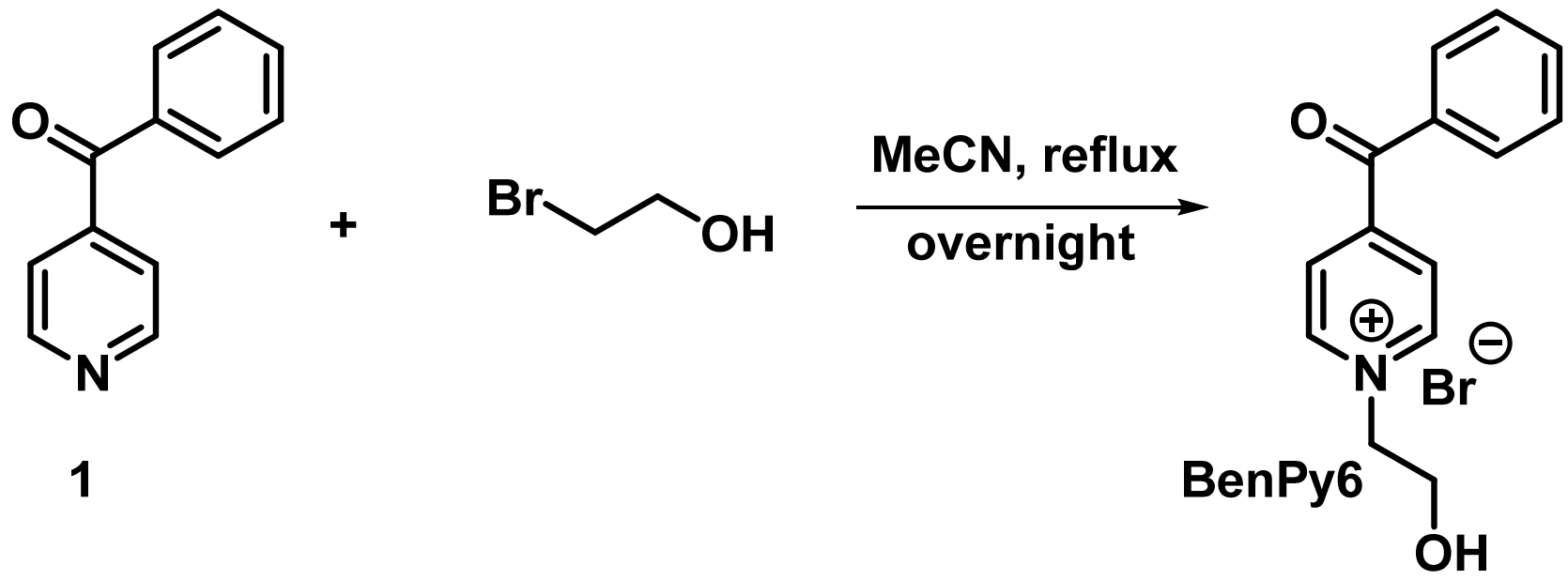

To a solution of pyridyl ketone **1** (0.400 g, 2.18 mmol, 1 equiv) in MeCN (1.0 mL) was added 2-bromoethanol (0.545 g, 4.36 mmol, 2 equiv) at rt. The reaction mixture was heated to reflux and refluxed overnight. After cooling to rt, the mixture was concentrated under reduced pressure. Purification of the residue via CombiFlash (0% to 10% MeOH/DCM) afforded the desired product **BenPy6** as yellow solid (0.357 g, 64%).**^{1}H NMR** (300 MHz, D_2O) δ 9.18 (d, J = 6.5 Hz, 2H), 8.40 (d, J = 6.2 Hz, 2H), 8.00 – 7.81 (m, 3H), 7.69 (t, J = 7.7 Hz, 2H), 4.94 (t, J = 4.9 Hz, 2H), 4.23 (t, J = 4.9 Hz, 2H); **^{13}C NMR** (75 MHz, D_2O) δ 194.1, 151.9, 145.9, 135.5, 133.9, 130.6 (2 CH), 129.2 (2 CH), 127.7 (2 CH), 64.0, 60.4; **IR** (film, cm^{-1}): 3261, 1670, 1445, 1323, 1278, 937, 847, 790; **mp** 170 – 173 °C; **HRMS** (ESI+): m/z calcd for $[C_{14}H_{14}NO_2]^+$ 228.1025, found 228.1018, Δ = 3.0 ppm.

2.7 4-benzoyl-1-(3-(trimethylammonio)propyl)pyridin-1-ium bromide BenPy7

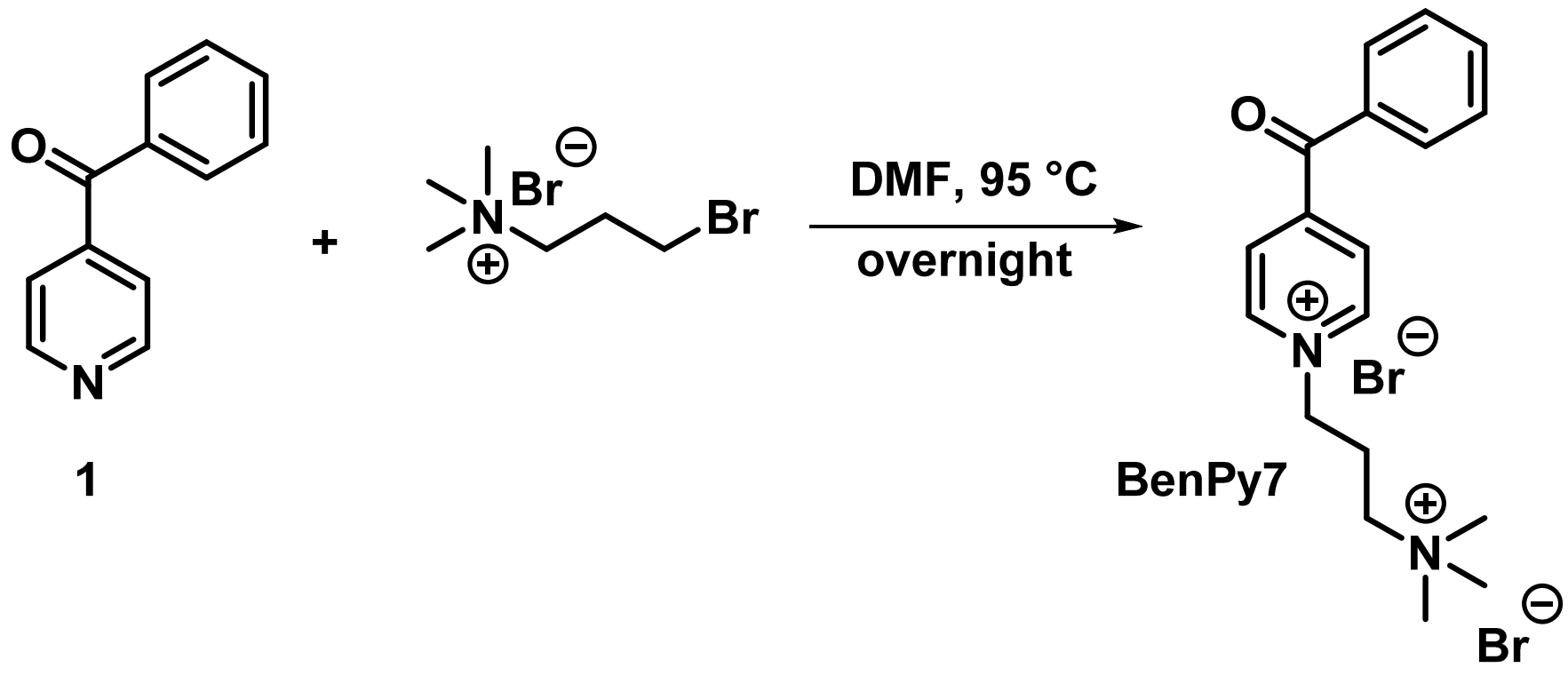

To a solution of pyridyl ketone **1** (2.0 g, 10.9 mmol, 1.0 equiv.) in DMF (25 mL) at rt was added 3.42 g of 3-bromopropyl-trimethylammonium bromide (13.1 mmol, 1.2

equiv.) in one portion. The resulting yellow mixture was heated to 90 °C with stirring under argon, during which time the mixture became orange. After 24h, the crude mixture was diluted with 20 mL of toluene to give a yellow precipitate. The resulting solid was recovered by filtration and washed with toluene (3 × 20 mL), $CHCl_3$ (3 × 20 mL) and hexane (3 × 20 mL). The solid cake was dried overnight under high vacuum to give 4.5 g (77%) of **BenPy7** as an orange powder. **^{1}H NMR** (500 MHz, D_2O) δ 9.14 – 9.09 (m, 2H), 8.34 (d, *J* = 6.1 Hz, 2H), 7.83 (dd, *J* = 8.4, 1.2 Hz, 2H), 7.81 – 7.74 (m, 1H), 7.65 – 7.56 (m, 2H), 4.85 – 4.75 (m, 2H), 3.50 (m, 2H), 3.20 – 3.13 (m, 9H), 2.63 (m, 2H); **^{13}C NMR** (126 MHz, D_2O) δ 193.8, 152.1, 145.6, 135.4 (2 CH), 133.8, 130.6, 129.1 (2 CH), 128.2 (2 CH), 62.4, 58.4, 53.3 (3 CH_3), 24.7; **IR** (film, cm^{-1}): 2523, 1754, 1412, 1221, 1101, 912, 710, 601; **mp** 250 – 253 °C; **HRMS** (ESI+): m/z calcd for $[C_{18}H_{24}N_2O]^{2+}$ 142.0938, found 142.0955, Δ =12.0 ppm.

3 Cyclic voltammetry measurements

The cyclic voltammetry tests were performed using different potentiostasts: Biologic SP-240, Palmsense4 or Gamry Gamry Reference 600+. All the test were conducted using a three-electrode set-up, with glassy carbon (3.0 mm diameter), Ag/AgCl (3 M KCl), and platinum wire as the working electrode, reference electrode, and counter electrode; respectively. Before each test, the glassy carbon was polished with 1 µm alumina and rinsed with DI water. When using a new electrolyte, the solution was degassed by bubbling nitrogen for 10 minutes and, after that, a continuous flow of nitrogen was hold above the electrolyte to minimize the oxygen content. Before the experiments, the resistance of the solution was measured using potentiometric electrochemical impedance spectroscopy (PEIS).

All preliminary studies were conducted using a concentration of 1 mM of the benzoylpyridinium derivative dissolved in 10 ml of the supporting electrolyte. The electrolyte post-mortem voltammetry analysis were performed straightforward without any preliminary dilution.

All potentials are reported against Ag/AgCl (3 M) reference, and all voltammograms presented in this section were performed at the scan rate of 100 mV s^{-1}. The peak-to-peak separation (AE_P) of the two main redox reactions is reported in the voltammograms as an indication of the process reversibility. The scheme S1 below presents the structures of the benzoylpyridinium derivatives studied during this research work.

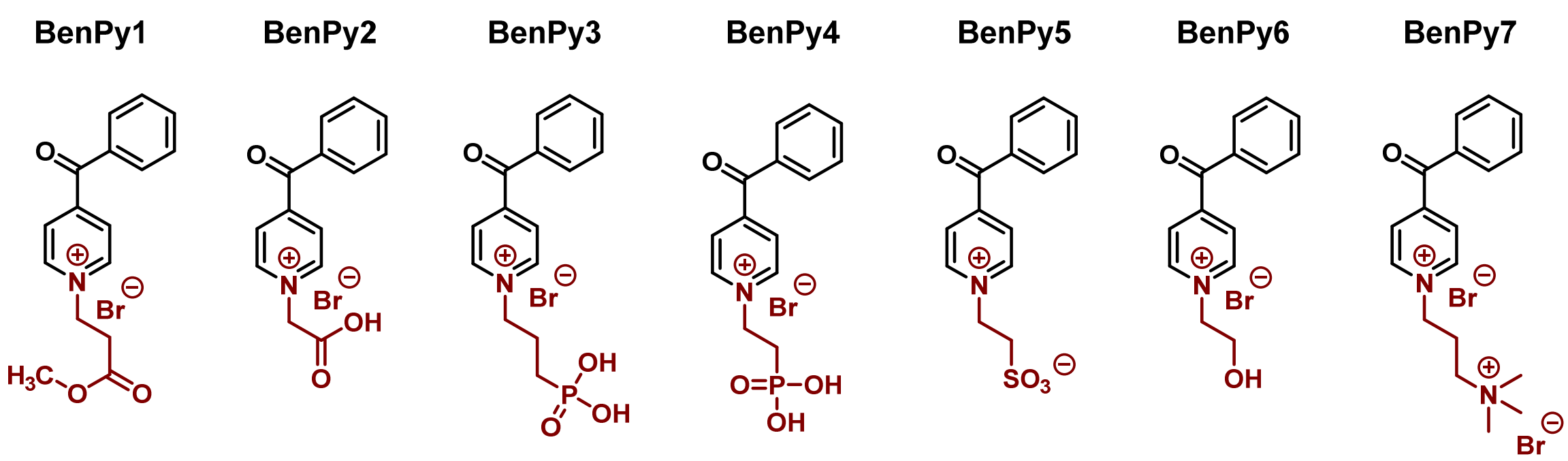

Scheme S1. Structures of the benzoylpyridinium derivatives studied during this research work

The redox potentials display shifts depending on the nature of the *N*-substituents, moving to more positive or negative values with the introduction of electron-withdrawing or -donating functional groups, respectively.[14,15] The more negative redox potentials are exhibited by **BenPy6**, featuring a hydroxy functional group, in agreement with the strong electron-

donating effect of this substituent. Focusing on alkaline media, **BenPy6** presents potentials of −0.744 and −0.902 V for the first and second reduction peaks, respectively. On the other hand, positive shifts were observed for the derivatives including electron-withdrawing substituents, like **BenPy2** and **BenPy5**, featuring carboxylate and sulfonate groups, respectively. In the case of **BenPy5**, the reduction peaks for the first and second redox process are located at −0.646 and −0.828 V, respectively, corresponding to the more positive values of the materials studied in this work.

Focusing on alkaline media, the cyclic voltammograms of **BenPy1** and **BenPy2** in section 3.1 indicated sluggish charge transfer process, Phosphonate containing **BenPy3** and **BenPy4** in section 3.2 also showed two poorly reversible processes in basic media with **BenPy3** presenting slightly higher redox potentials than **BenPy4**, in accordance with the shorter alkyl chain. This observation points out that the chain length can be used to modulate the redox potential of the material, as it has been also demonstrated in a previous study.[17] A sulfonate group on the 4-benzopyridine core with **BenPy5** resulted in high reversibility for the two peaks. However, the low solubility of **BenPy5** did not encourage further investigation. This is similar to what we observed with the azoniafluorenone core in our previous work, where the solubility of the zwitterionic compound **AZONS3** was very low in aqueous media.[11] Finally, we focused on the compounds **BenPy6** and **BenPy7**, bearing a hydroxyethyl and a quaternary ammonium functional group, respectively. In alkaline media, both compounds display two reversible redox processes, coupled with an excellent solubility in water. The maximum solubility in water was determined using UV-Vis measurements (see Supporting Information S5) as 3.5 for **BenPy6** and 2.2 M for **BenPy7**, which would lead to very high storage capacities of 188 and 118 Ah L^{-1}, respectively, if considering the utilization of the two electron processes. Based on these promising preliminary results, we decided to study the performance of these two derivatives in lab-scale flow batteries.

3.1 BenPy1

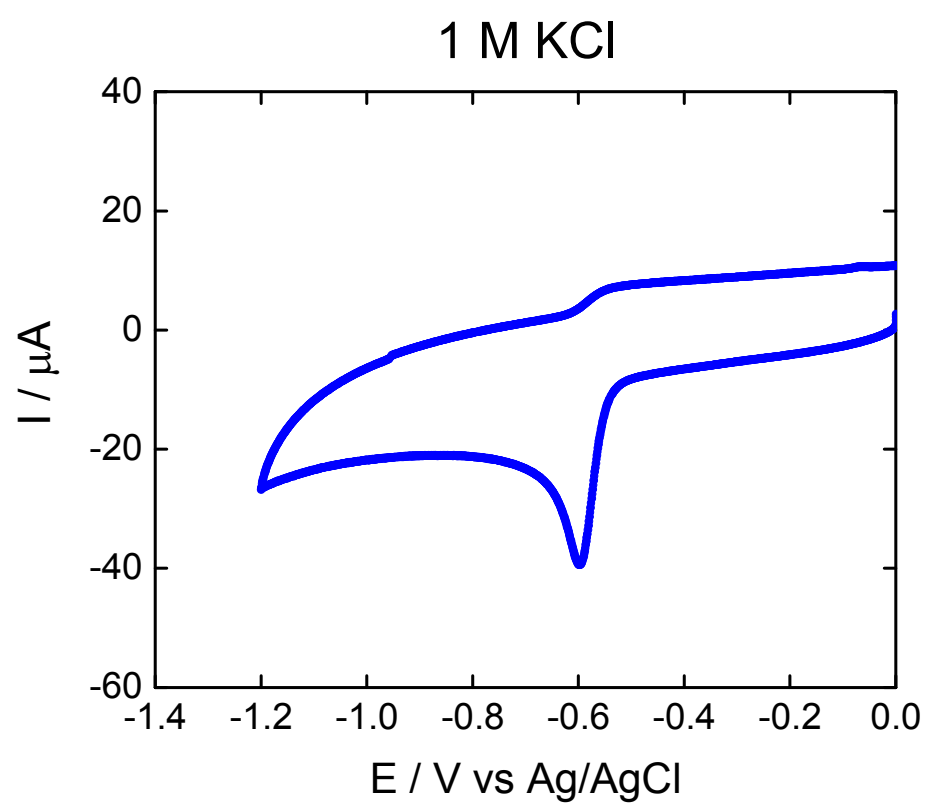

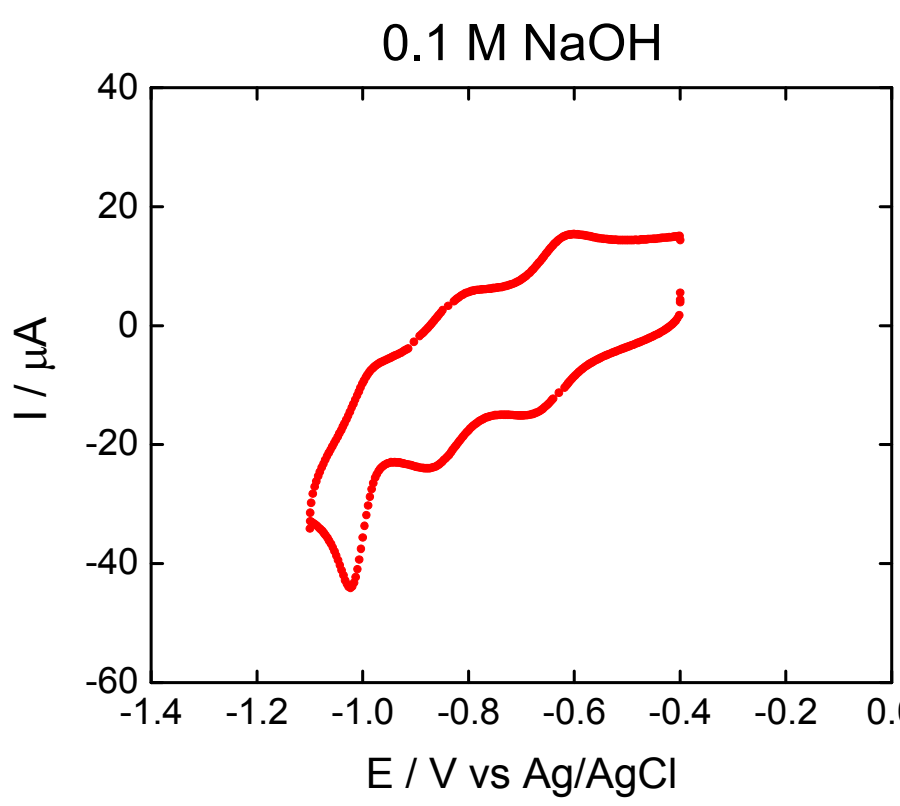

AE_{P1} = 91 mV

AE_{P2} = 113 mV

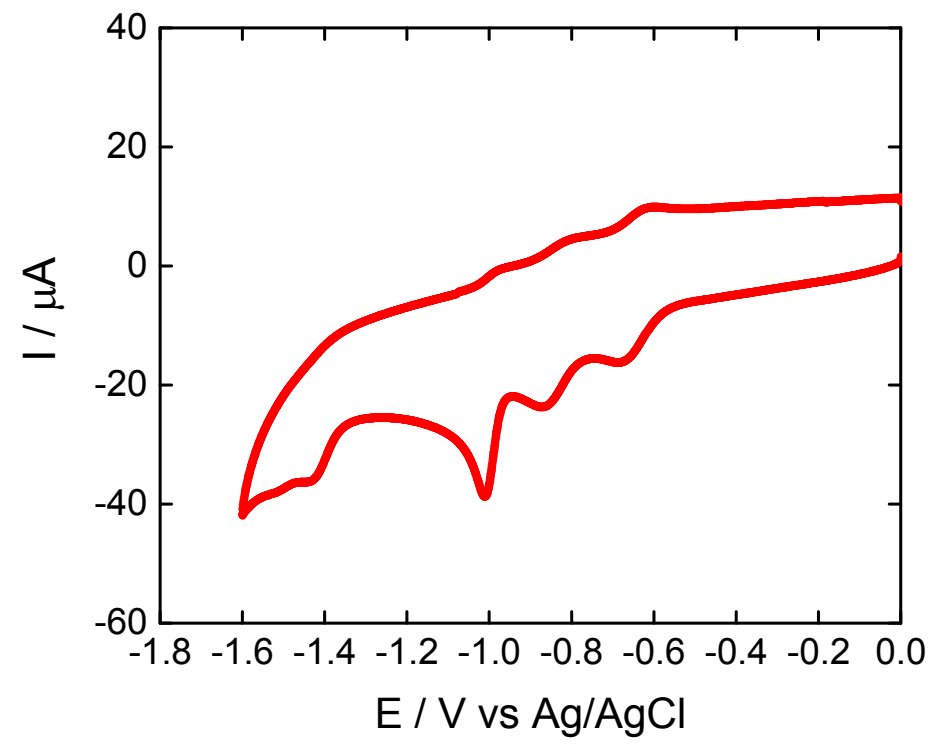

3.2 BenPy2

1 M KCl

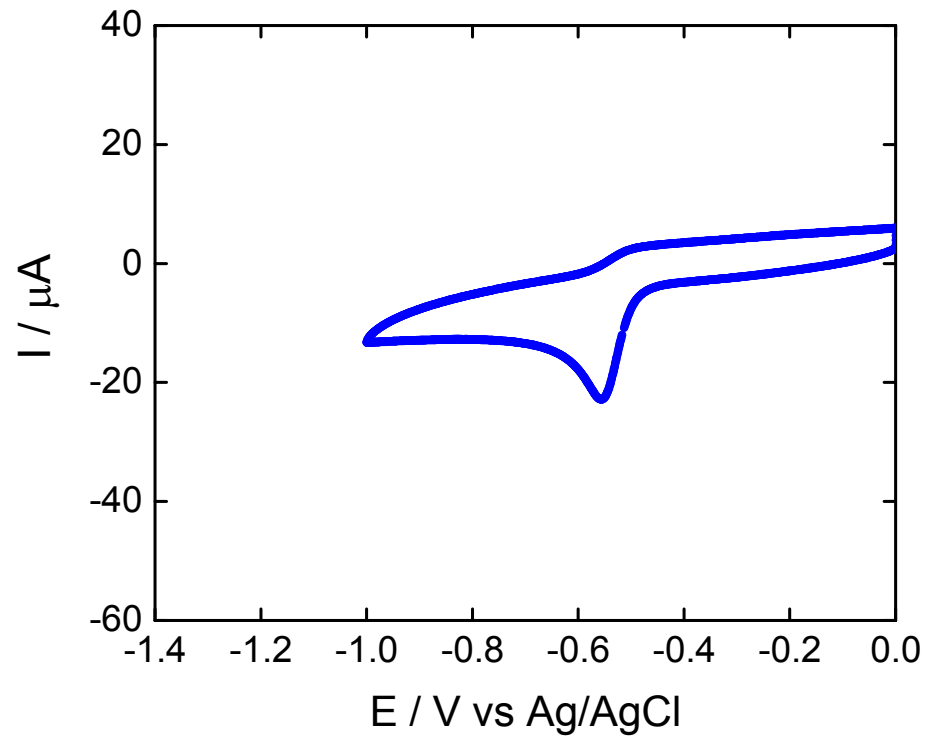

0.1 M NaOH

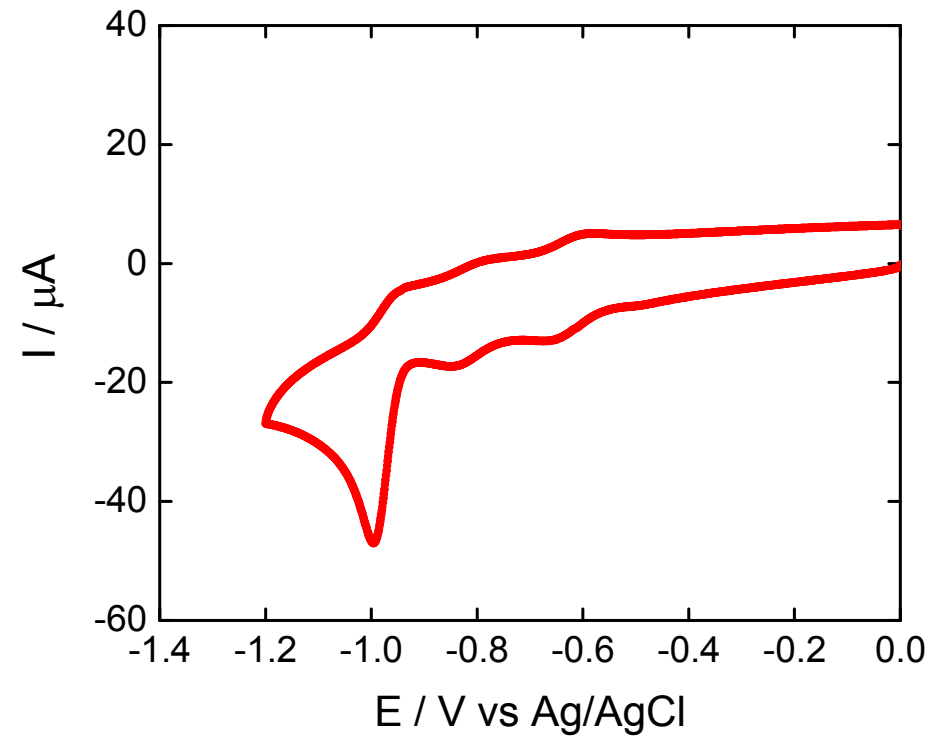

AE_{P1} = 90 mV

AE_{P2} = 100 mV

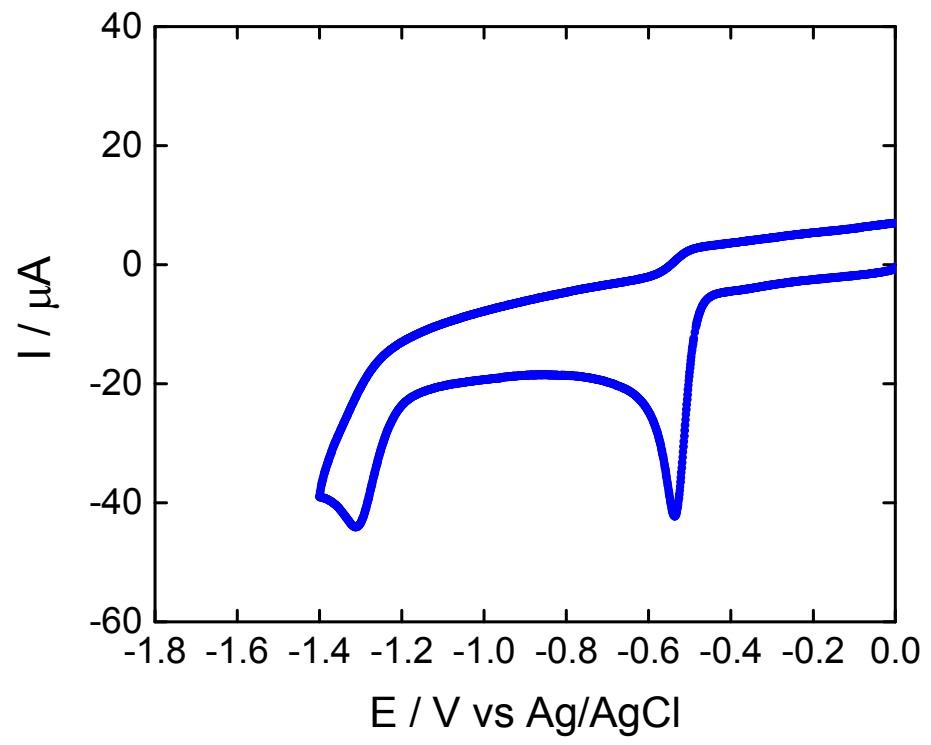

3.3 BenPy3

1 M KCl

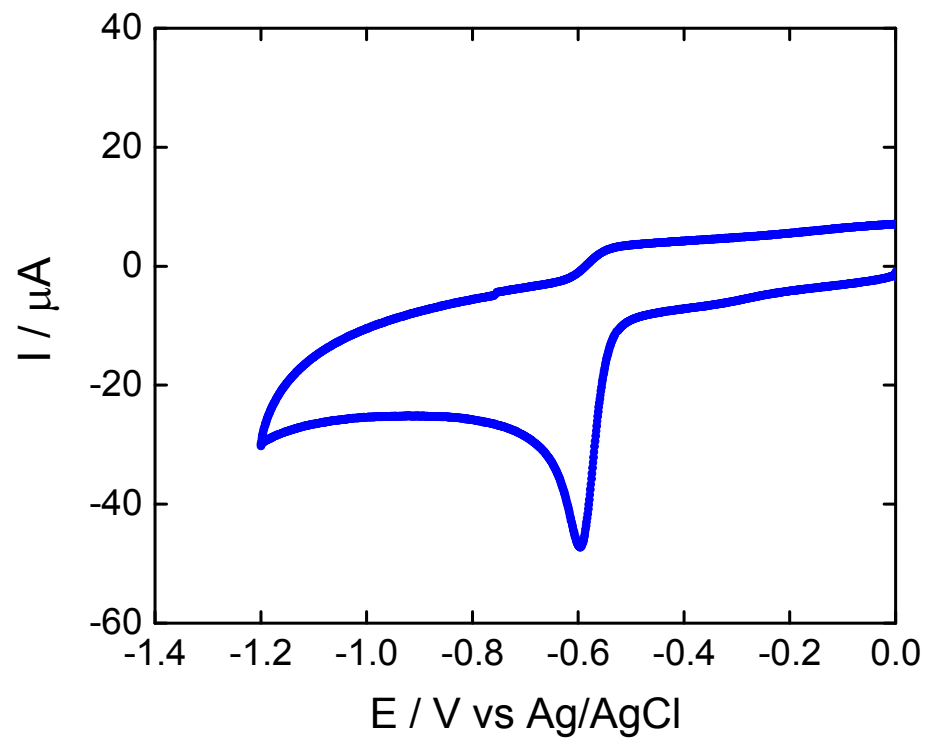

0.1 M NaOH

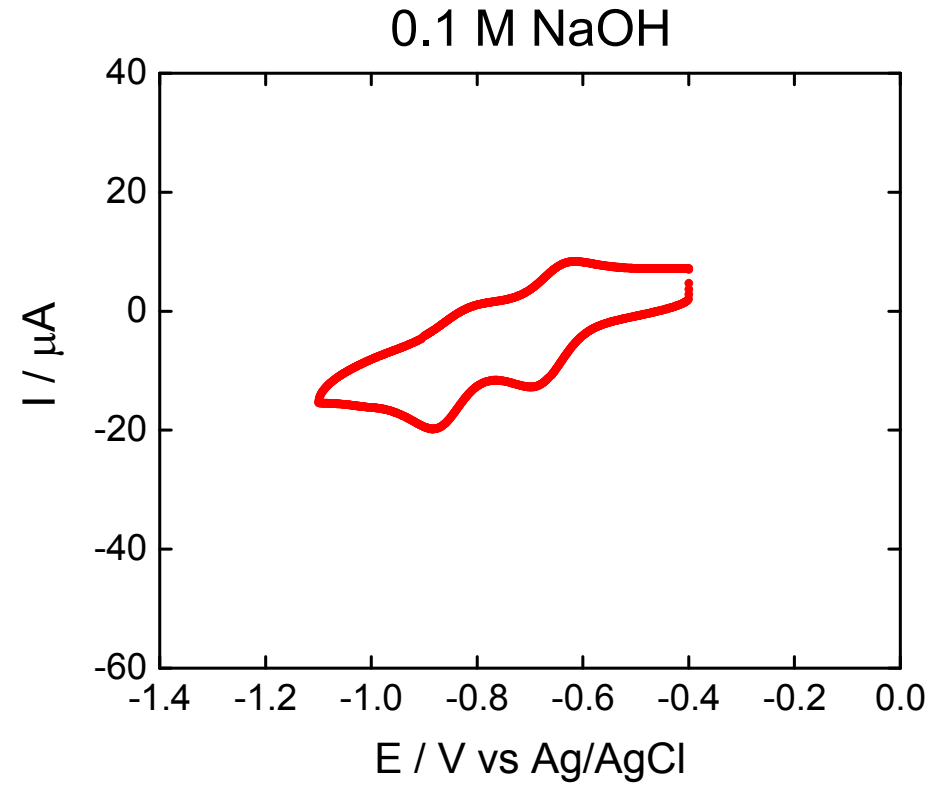

AE_{P1} = 85 mV

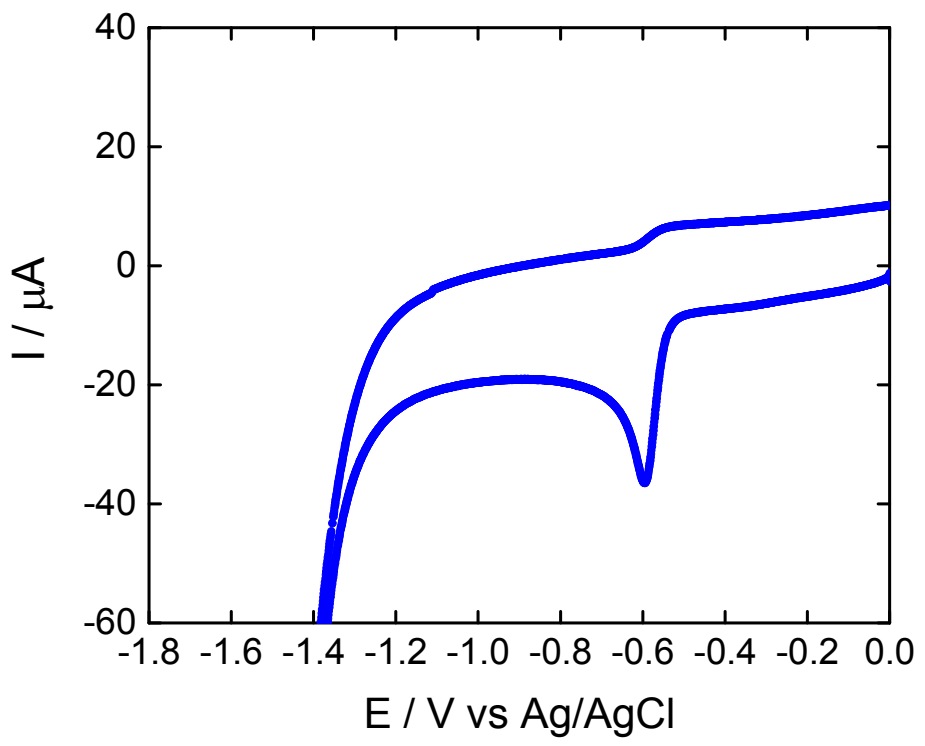

AE_{P2} = 111 mV

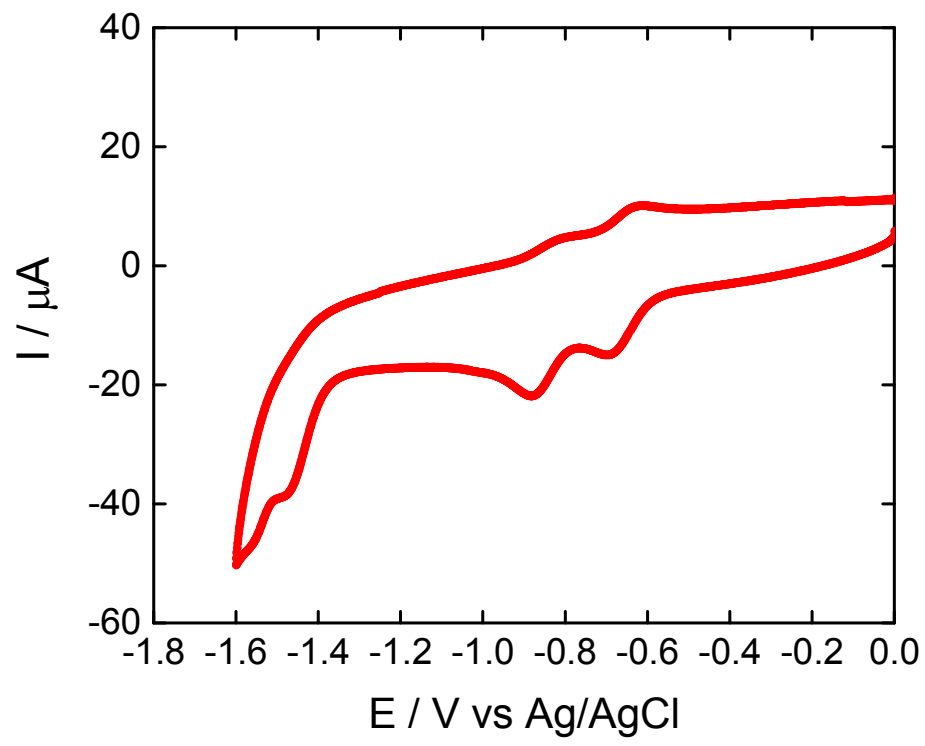

3.4 BenPy4

1 M KCl

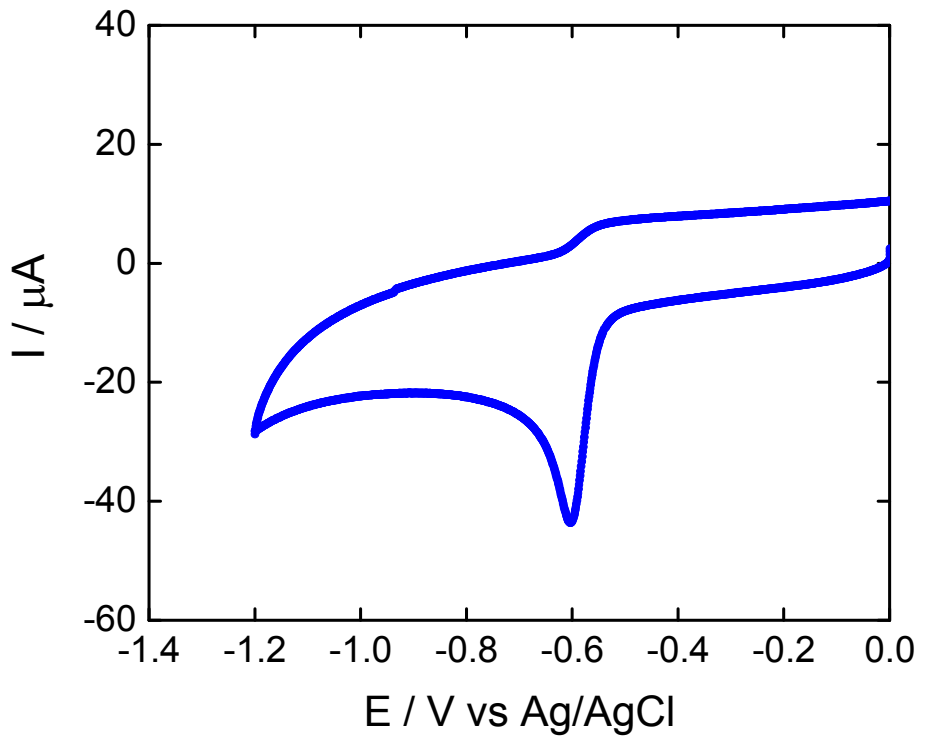

0.1 M NaOH

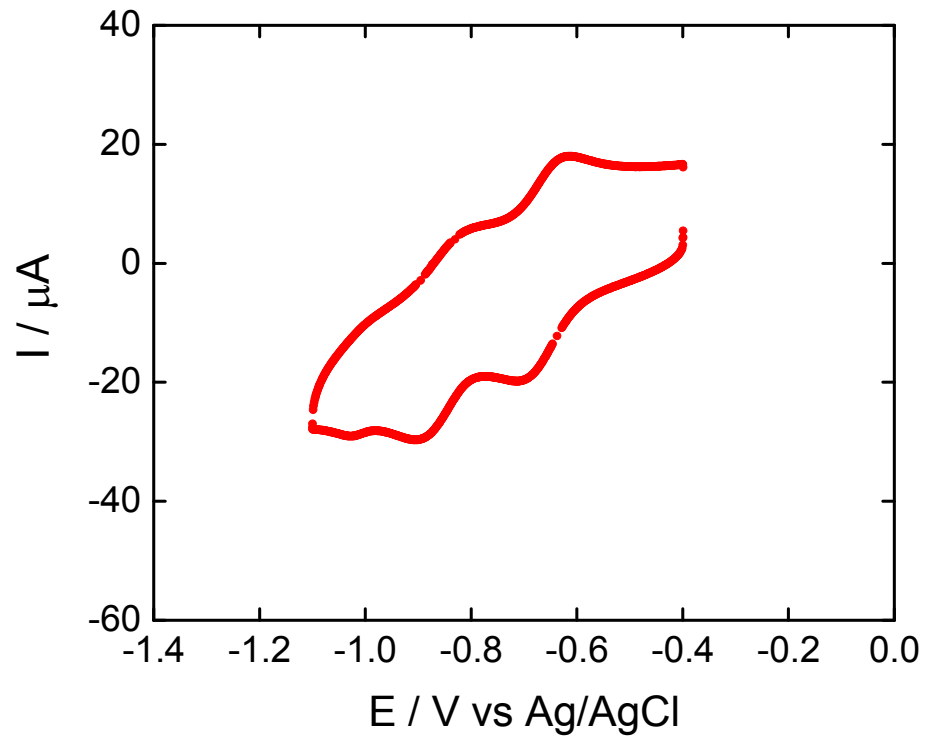

AE_{P1} = 88 mV

AE_{P2} = 104 mV

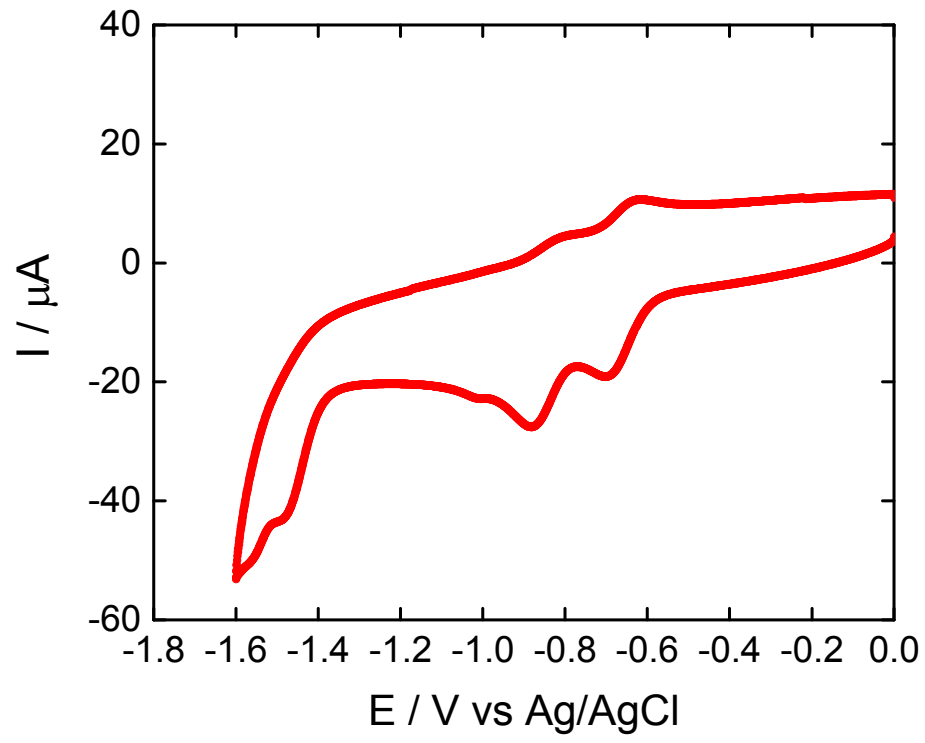

3.5 BenPy5

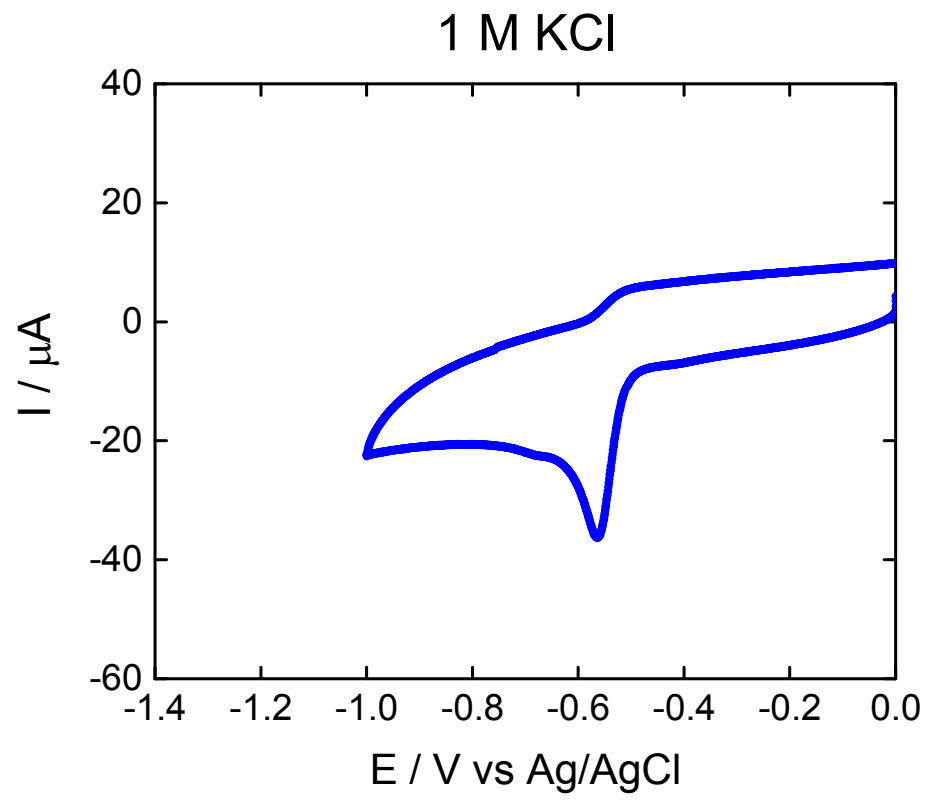

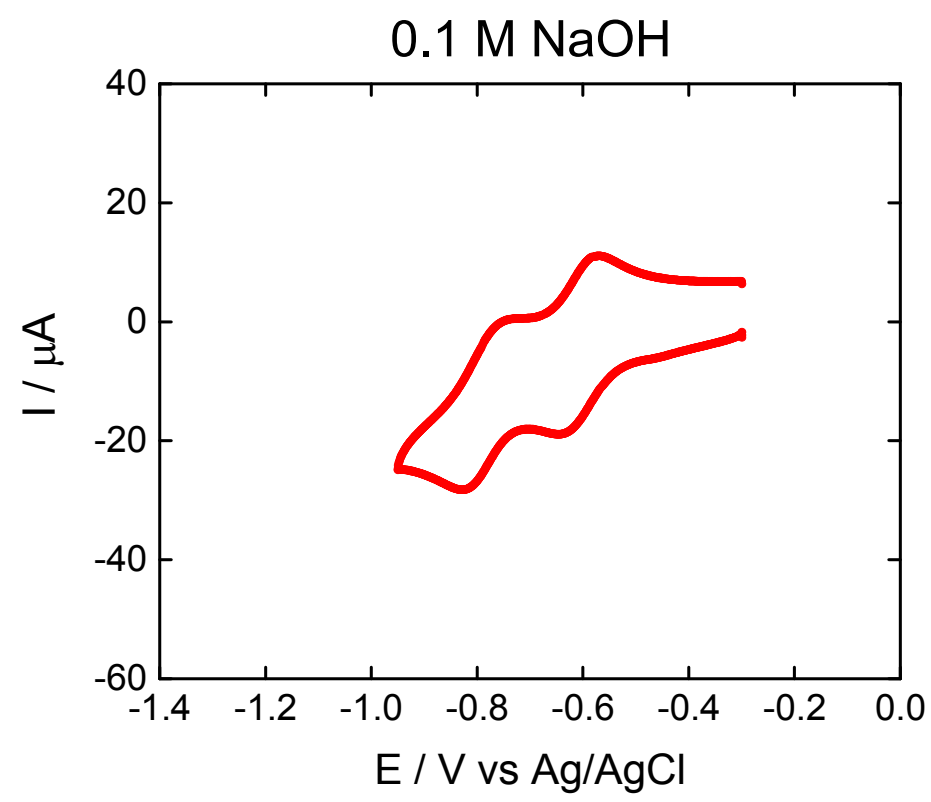

AE_{P1} = 75 mV

AE_{P2} = 98 mV

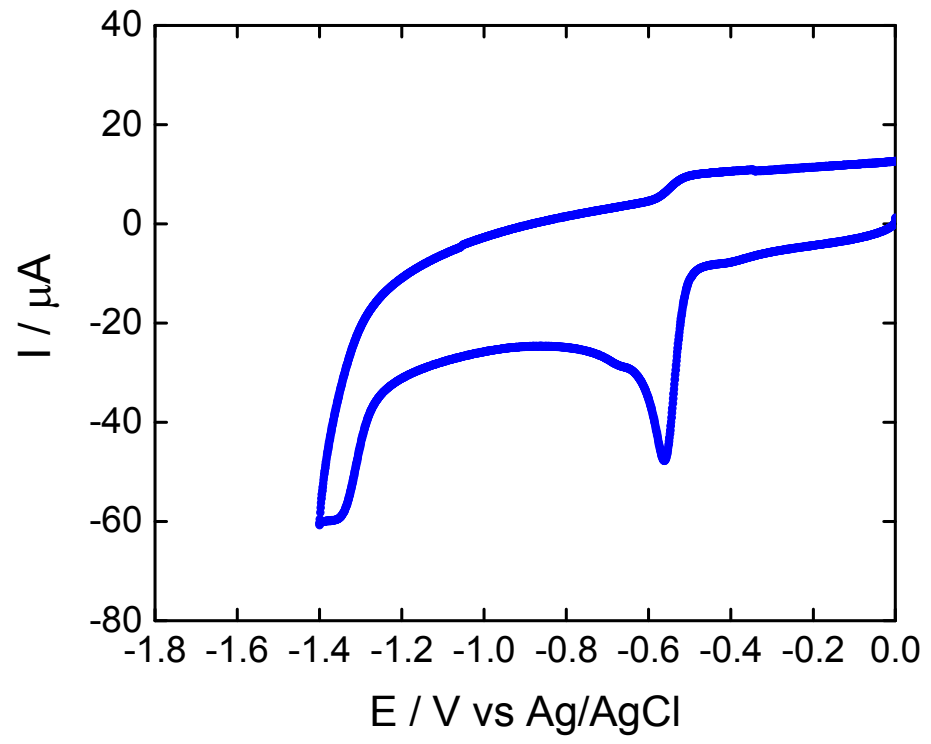

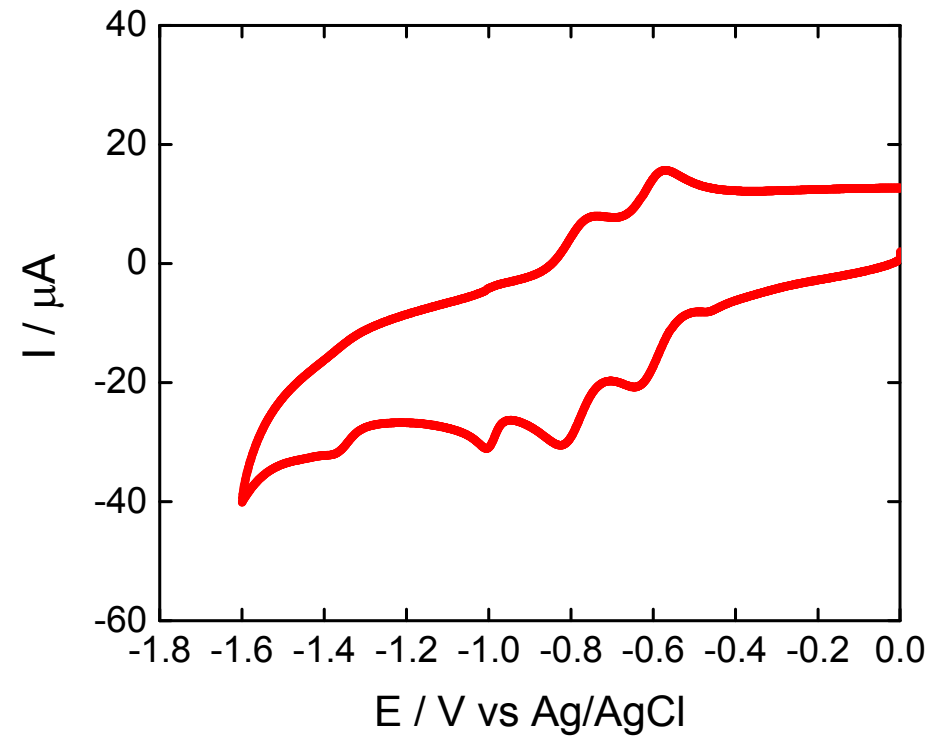

3.6 BenPy6

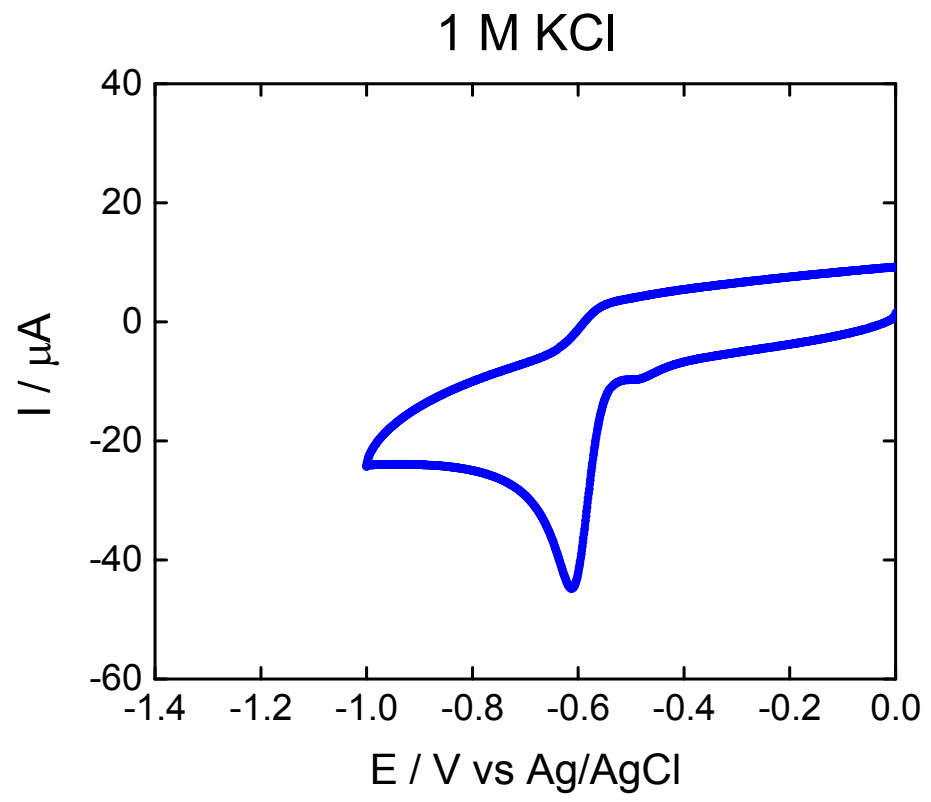

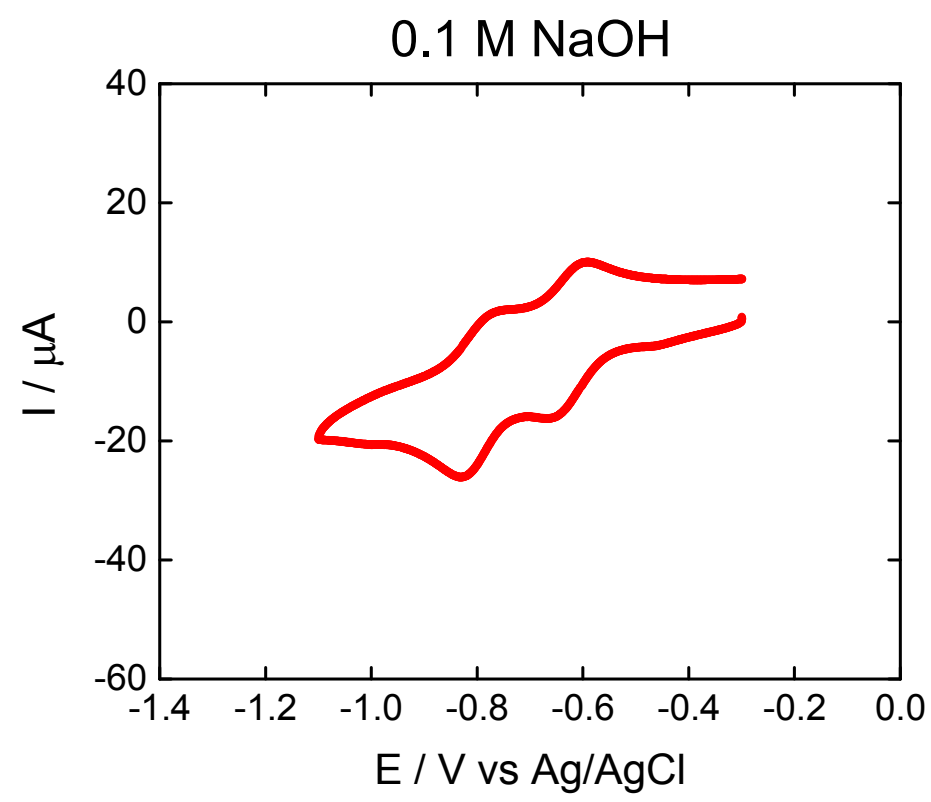

AE_{P1} = 84 mV

AE_{P2} = 78 mV

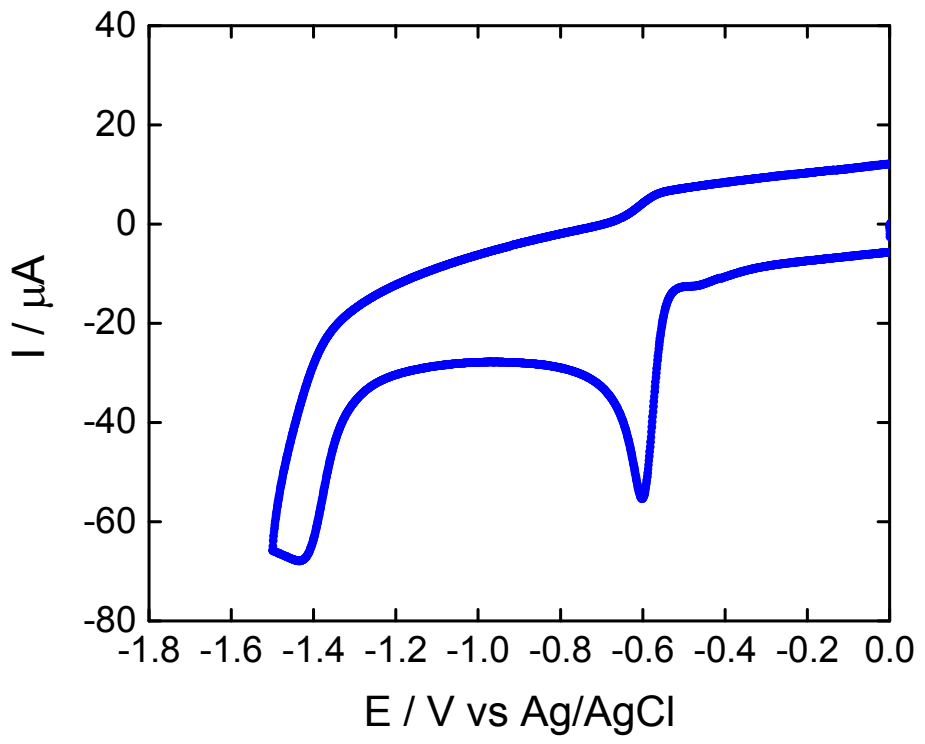

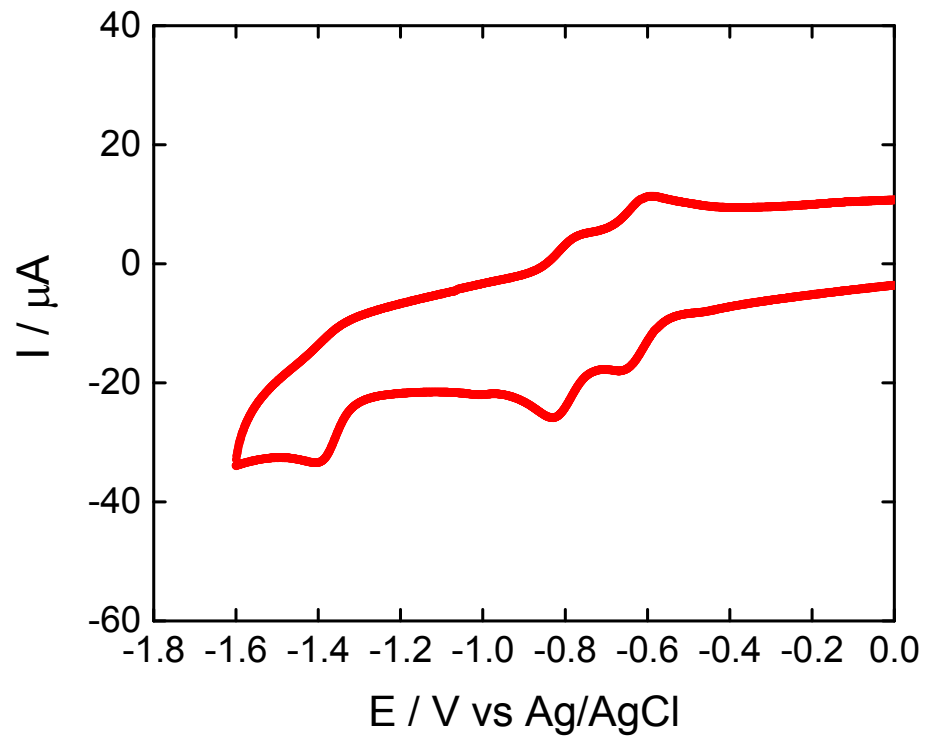

3.7 BenPy7

1 M KCl

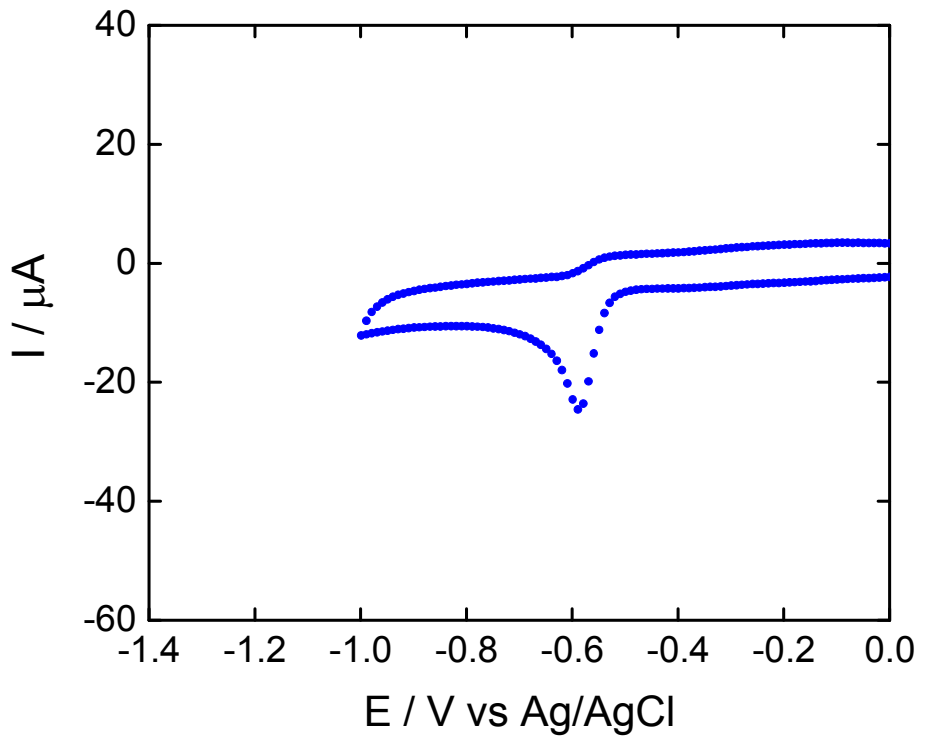

0.1 M NaOH

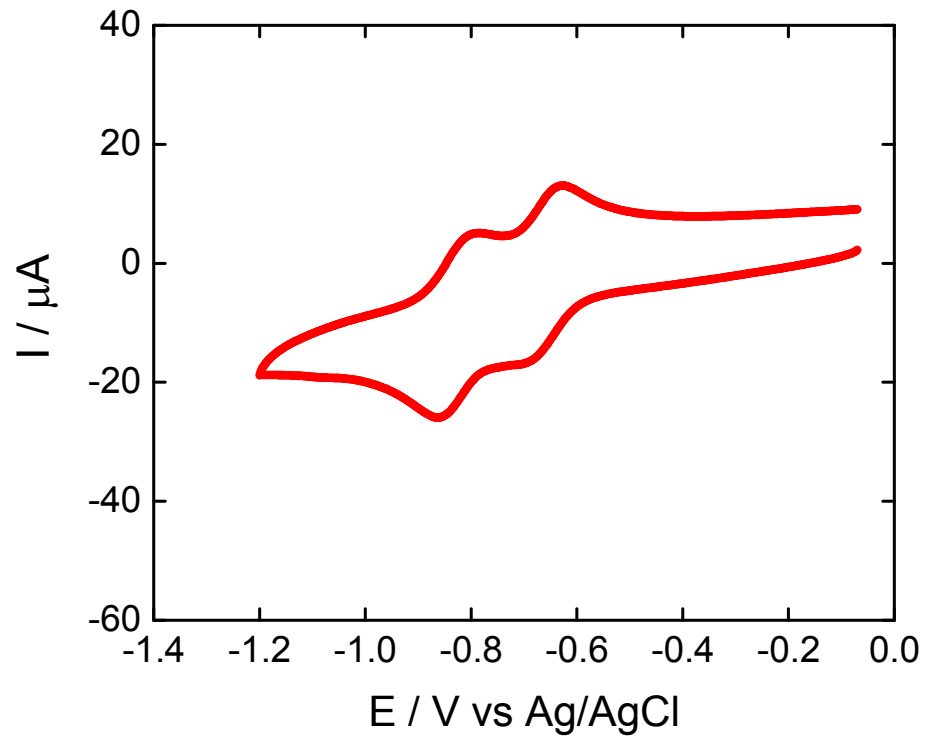

AE_{P1} = 73 mV

AE_{P2} = 78 mV

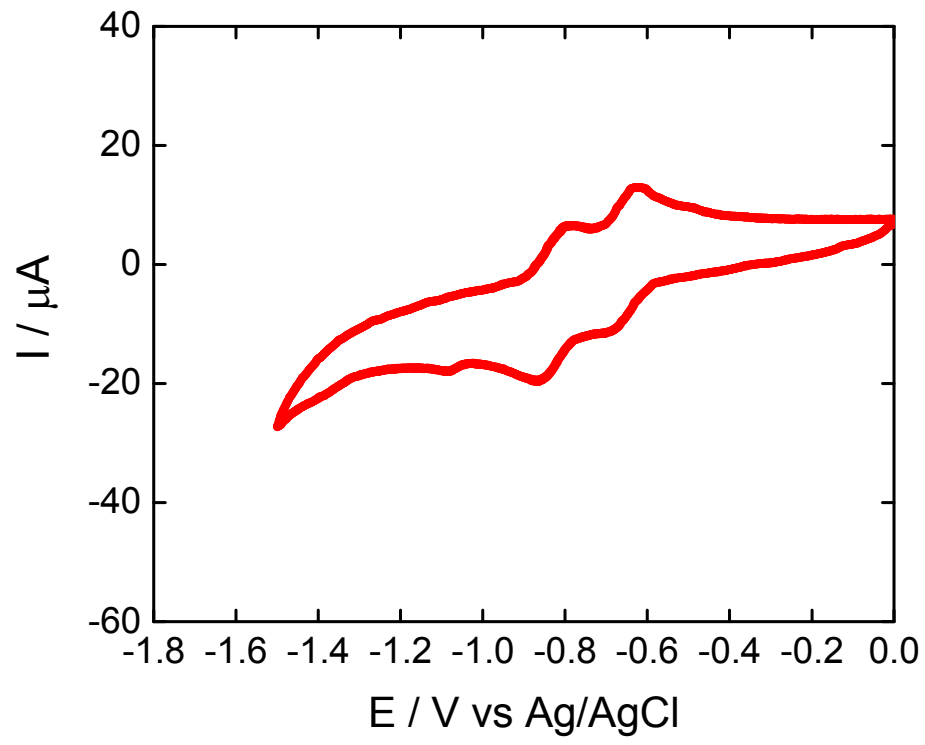

3.8 Electrolyte post-mortem analysis after cycling

3.8.1 BenPy6 battery in 0.1 M NaOH

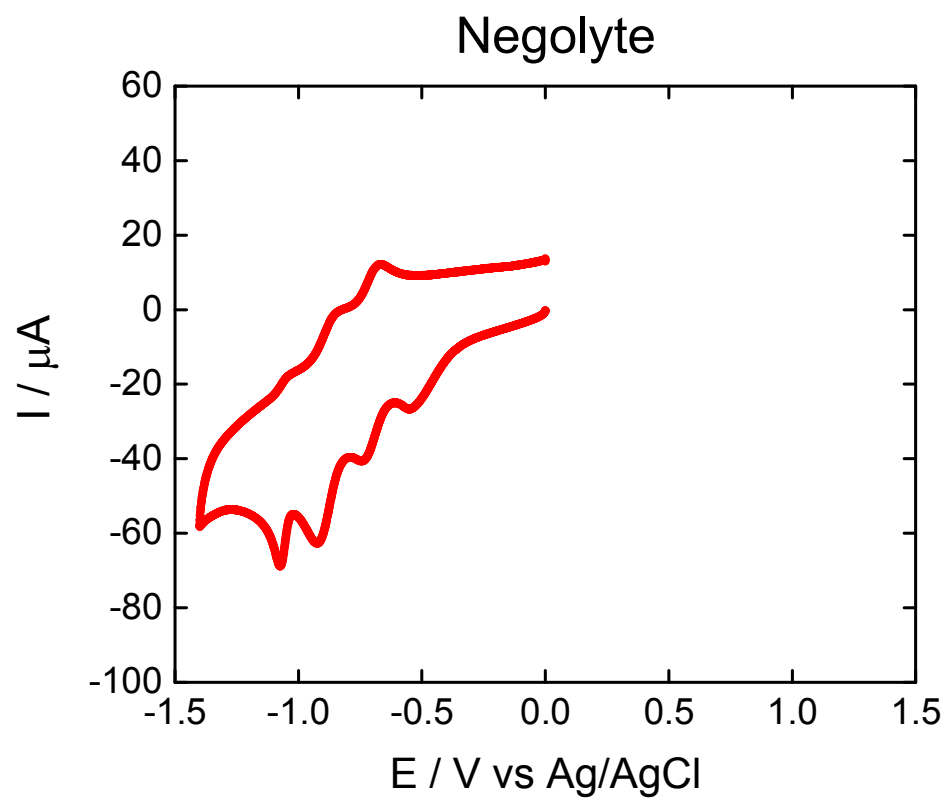

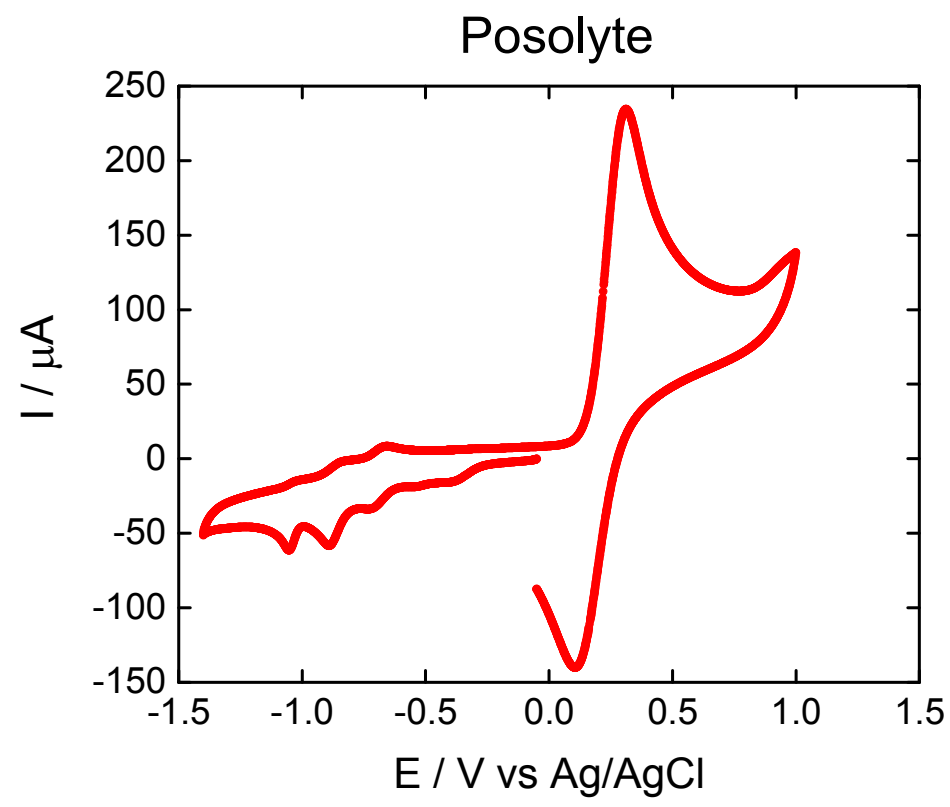

3.8.2 BenPy6 battery in 2 M NaOH

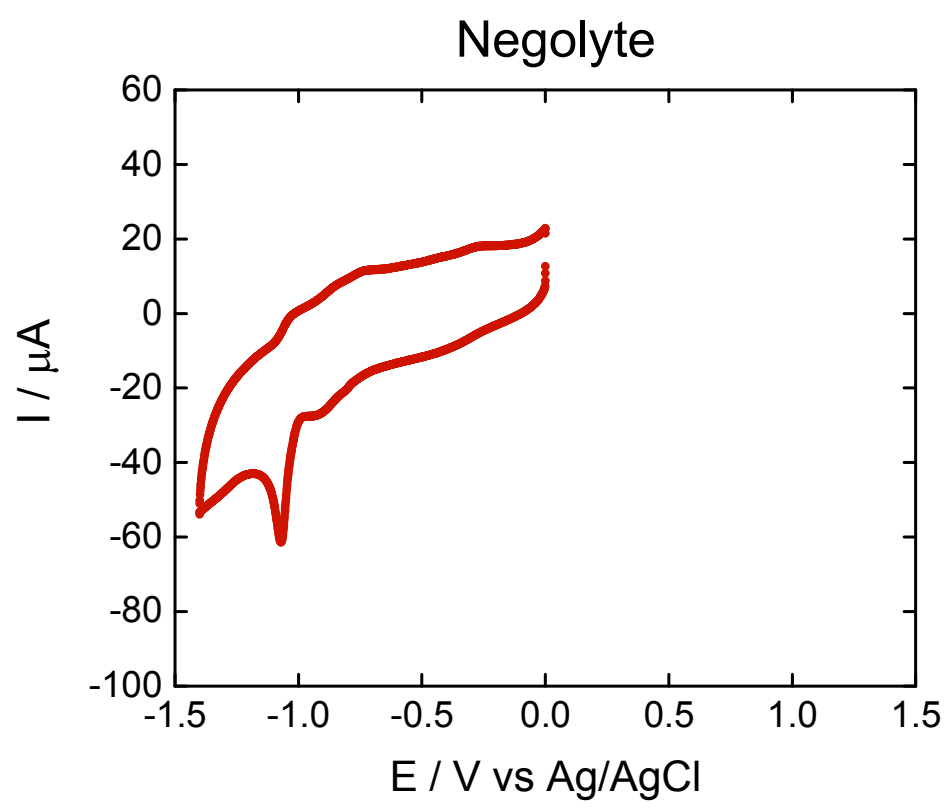

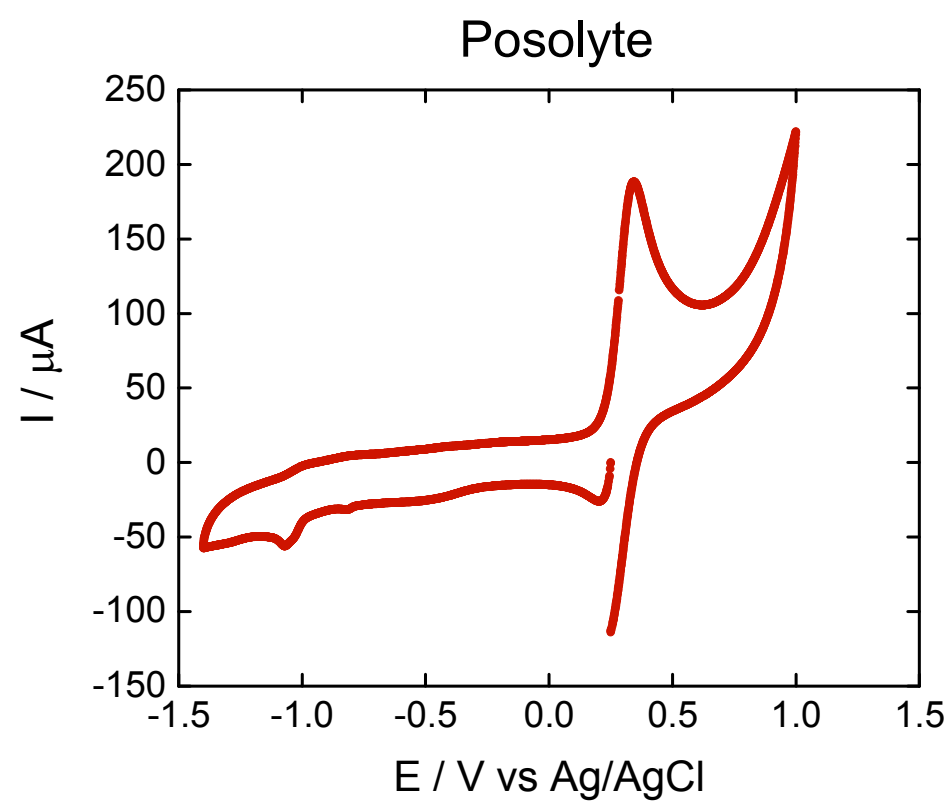

3.8.3 BenPy7 battery in 1 M KCl pH 9.2

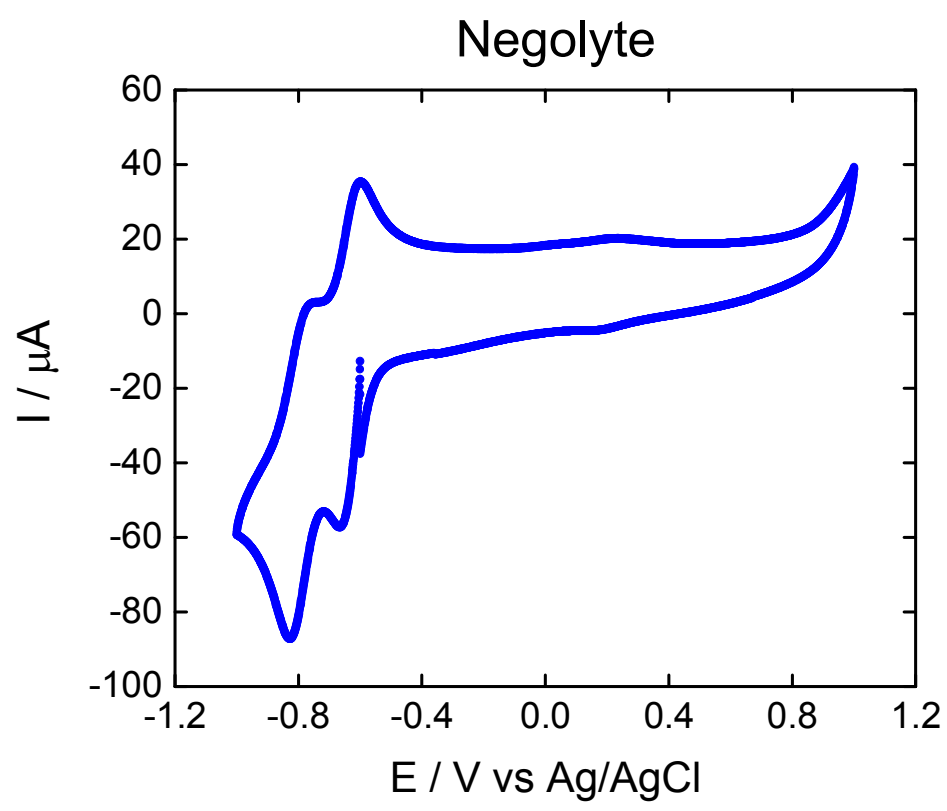

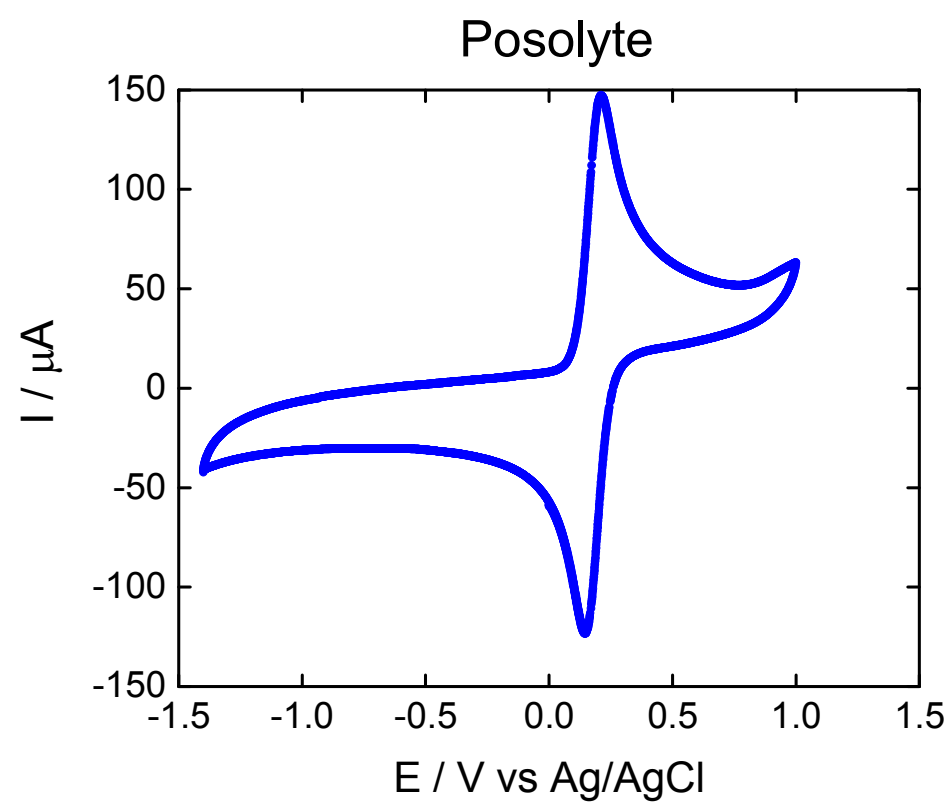

4 Flow battery studies

The stability of the new benzoylpyridinium materials was studied in lab-scale flow batteries using a concentration of 10 mM of the active material in different supporting electrolytes. The tests were performed using 5 cm^2 flow cells fabricated at the workshop of University of Turku, consisting on endplates (made of PVC), bipolar plates (fabricated from carbon composite obtained from Pinflow) and PTFE gaskets (3 mm thickness, cut from 24SH-ePTFE gasket sheets from TEADIT, Switzerland). The electrodes were thermally activated SGL carbon felts 4.6 mm from SIGRACELL (GFD 4.65 EA), which were sonicated in DI water for 10 minutes before assembling the cell. In the case of the membranes, we used anionic exchange membrane Selemion DSVN (from AGC) for the tests including BenPy7 and cationic exchange membrane Nafion212 (from IonPower) for the BenPy6 batteries.

All the experiments were conducted in a nitrogen-filled glovebox (MBRAUN) at room temperature. The cycling tests were performed galvanostatically using a battery cycler LANHE Battery Testing System G340A. The electrolytes were pumped with peristaltic pumps Baoding Chuangrui and tubing Masterflex C-Flex, with a constant flow rate of 50 ml min^{-1}.

The electrolytes were prepared outside the glovebox and degassed with Argon prior taking them inside. When required, the pH of the solution (after dissolving the material) was adjusted using diluted NaOH. An excess amount of posolyte was used to ensure the negolyte as the capacity limiting side, considering a two-electron process. For the BenPy6 batteries, the posolyte consisted on a mixture of sodium ferrocyanide/ferricyanide, while 1,1'-Bis[3-(trimethylammonio)propyl]ferrocene (BTMAP-Fc) was the posolyte for the BenPy 7 tests.

4.1 BenPy6

4.1.1 Battery in 0.1 M NaOH

Negolyte: 12 ml of 10 mM BenPy6 in 0.1 M NaOH
Posolyte: 30 ml of 10 mM $Na_4Fe(CN)_6$ + 3 mM $K_3Fe(CN)_6$ in 0.1 M NaOH

- Cut-off voltages: 0.40 – 1.10 V
- Cycling current: 2 mA cm^{-2}

Theoretical capacity expected for 2-electrons: 6.43 mAh

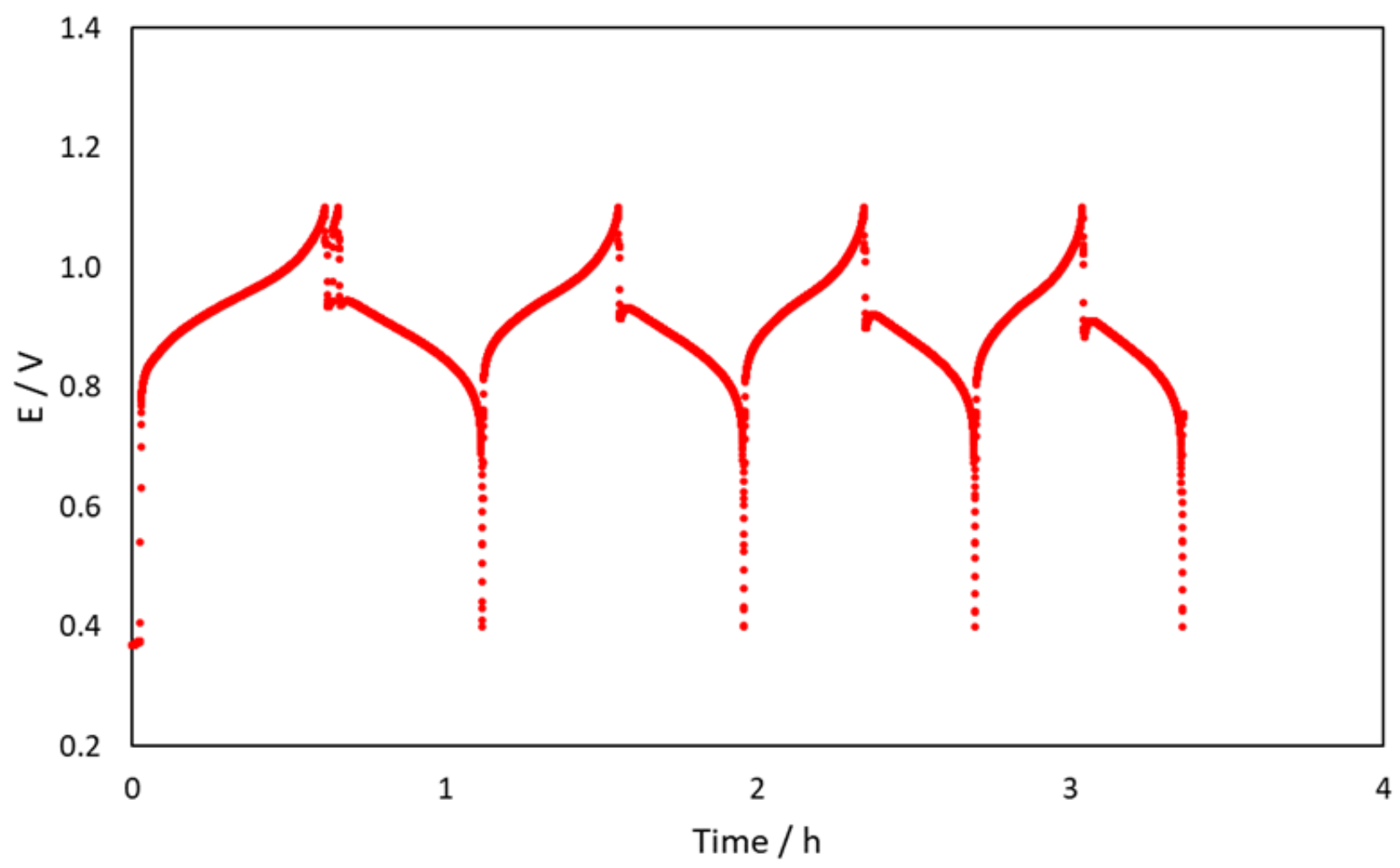

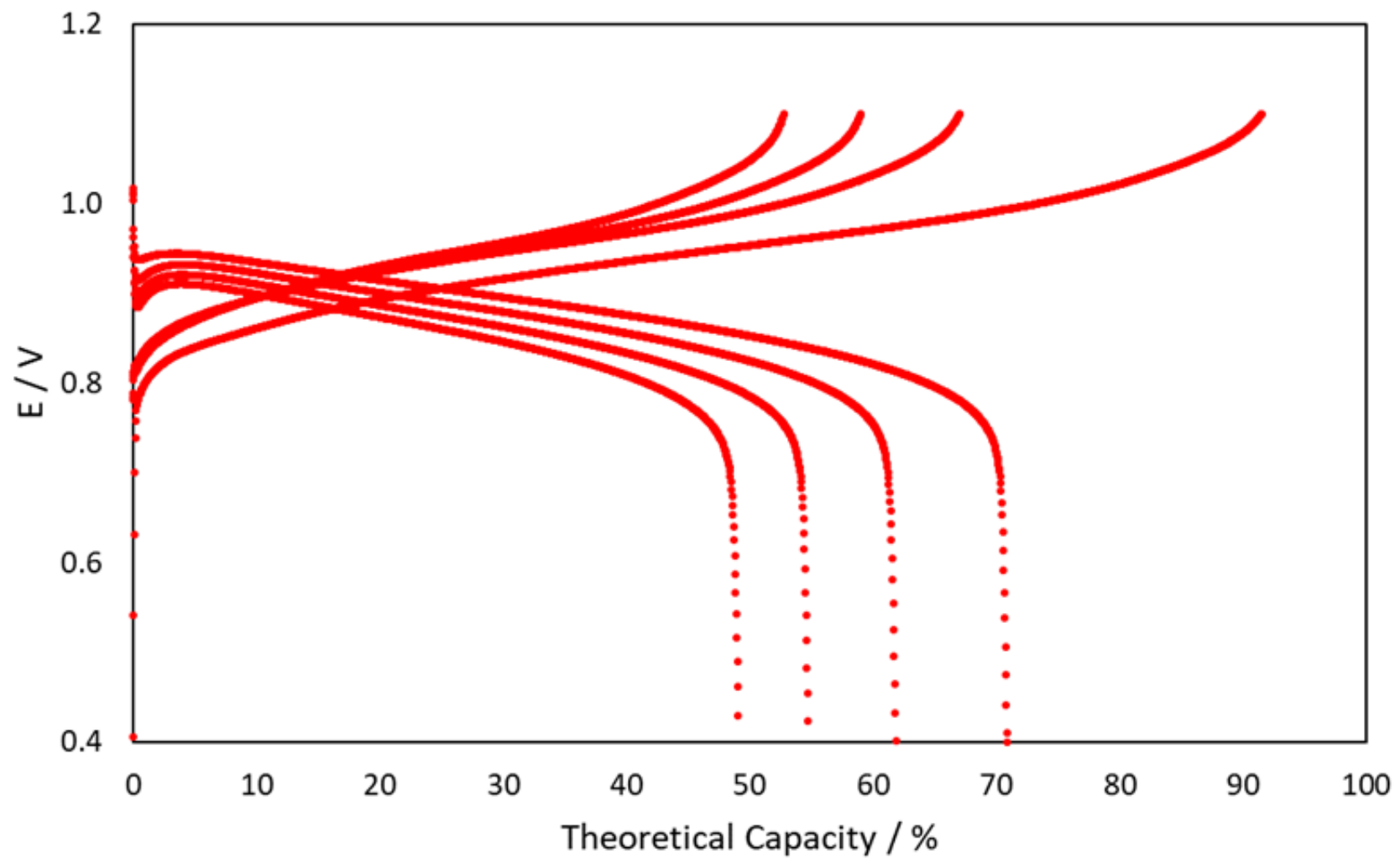

4.1.2 Battery in 2 M NaOH

Negolyte: 12 ml of 10 mM BenPy6 in 2 M NaOH
Posolyte: 30 ml of 10 mM $Na_4Fe(CN)_6$ + 3 mM $K_3Fe(CN)_6$ in 2 M NaOH

- Cut-off voltages: 0.40 – 1.20 V
- Cycling current: 2 mA cm^{-2}

Theoretical capacity expected for 2-electrons: 6.43 mAh

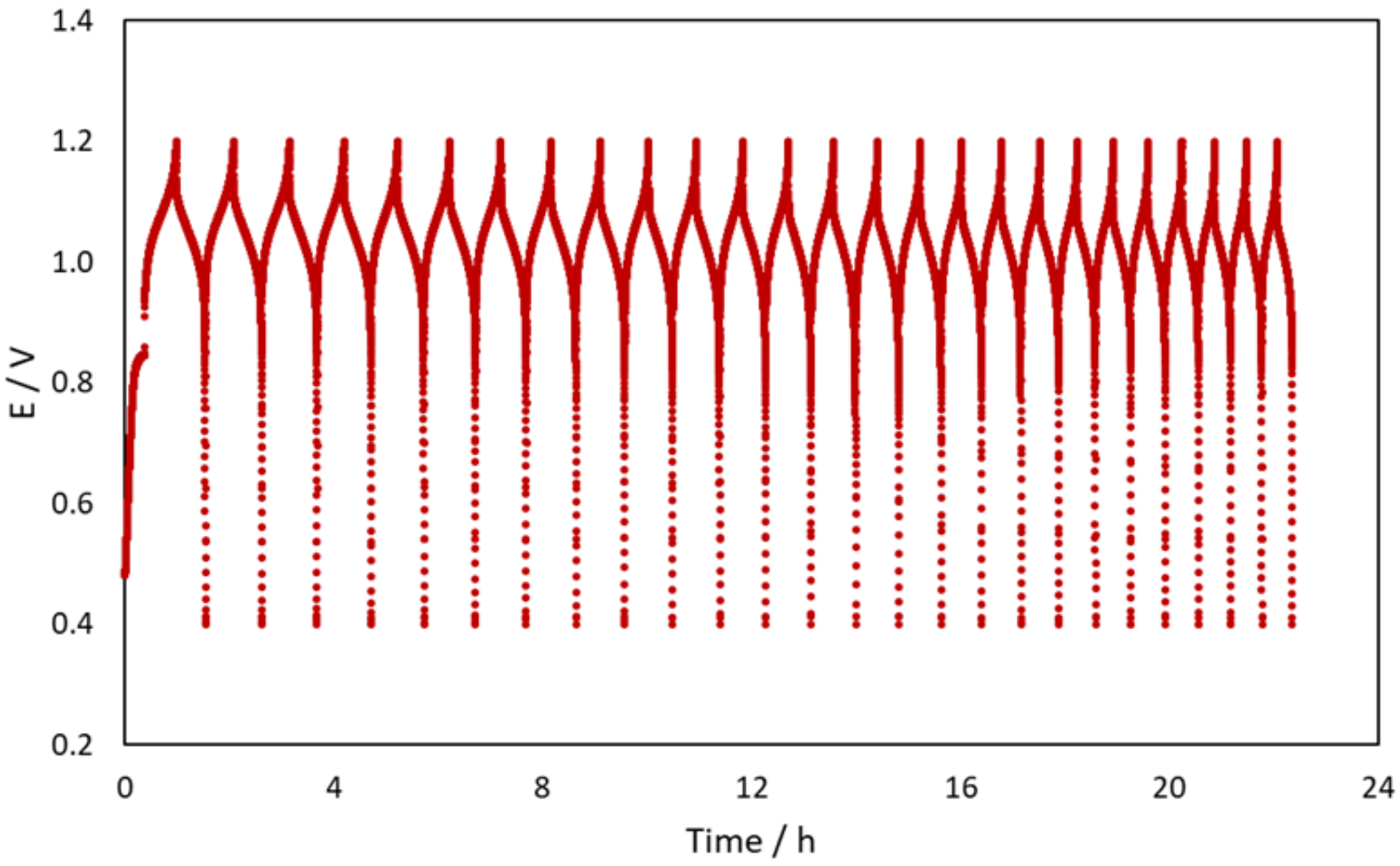

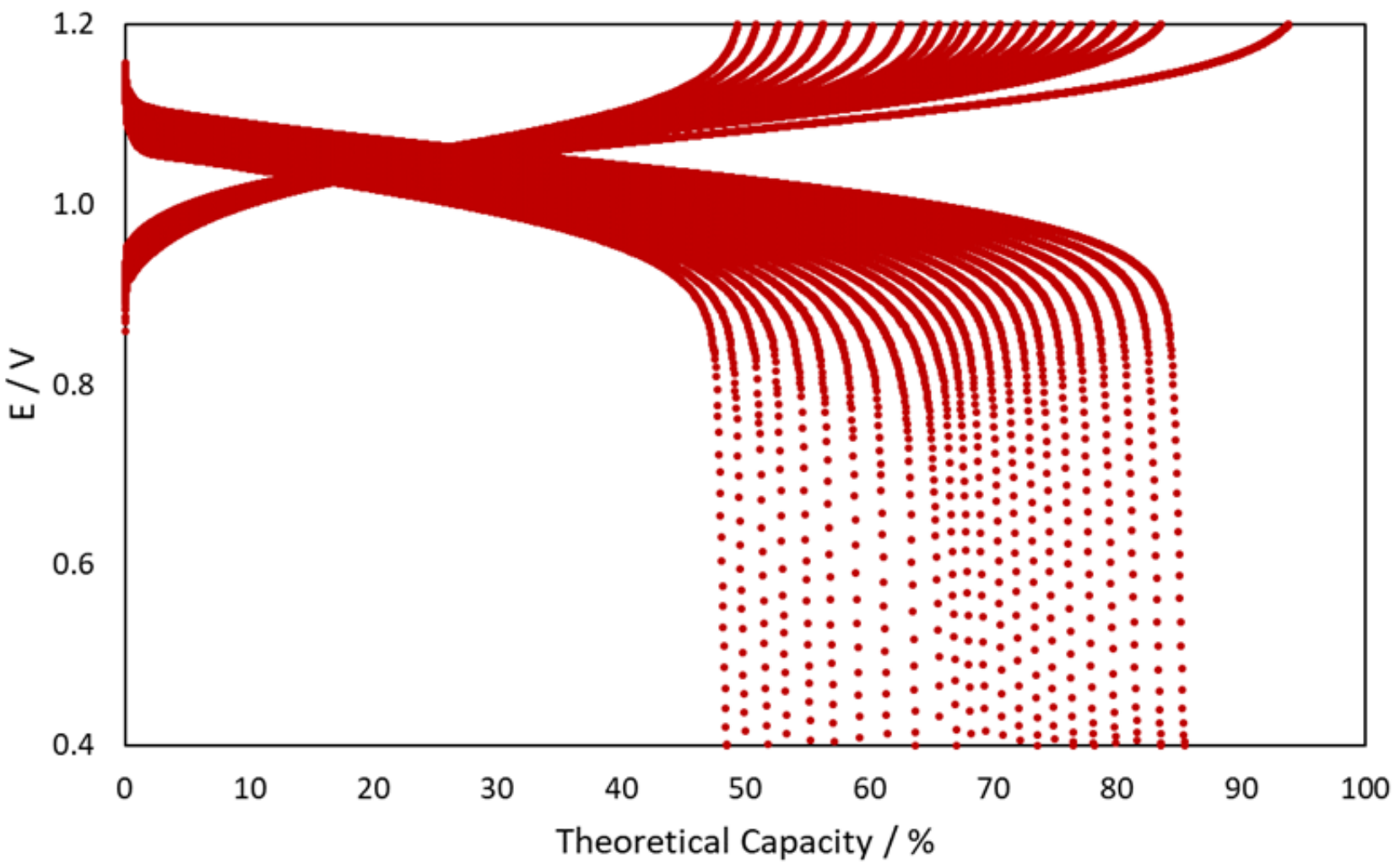

4.2 BenPy7

4.2.1 Battery in 0.1 M NaOH

Negolyte: 11 ml of 10 mM BenPy7 in 0.1 M NaOH
Posolyte: 25 ml of 10 mM BTMAP-Fc in 0.1 M NaOH

- Cut-off voltages: 0.40 – 1.05 V
- Cycling current: 2 mA cm^{-2}

Theoretical capacity expected for 2-electrons: 5.90 mAh

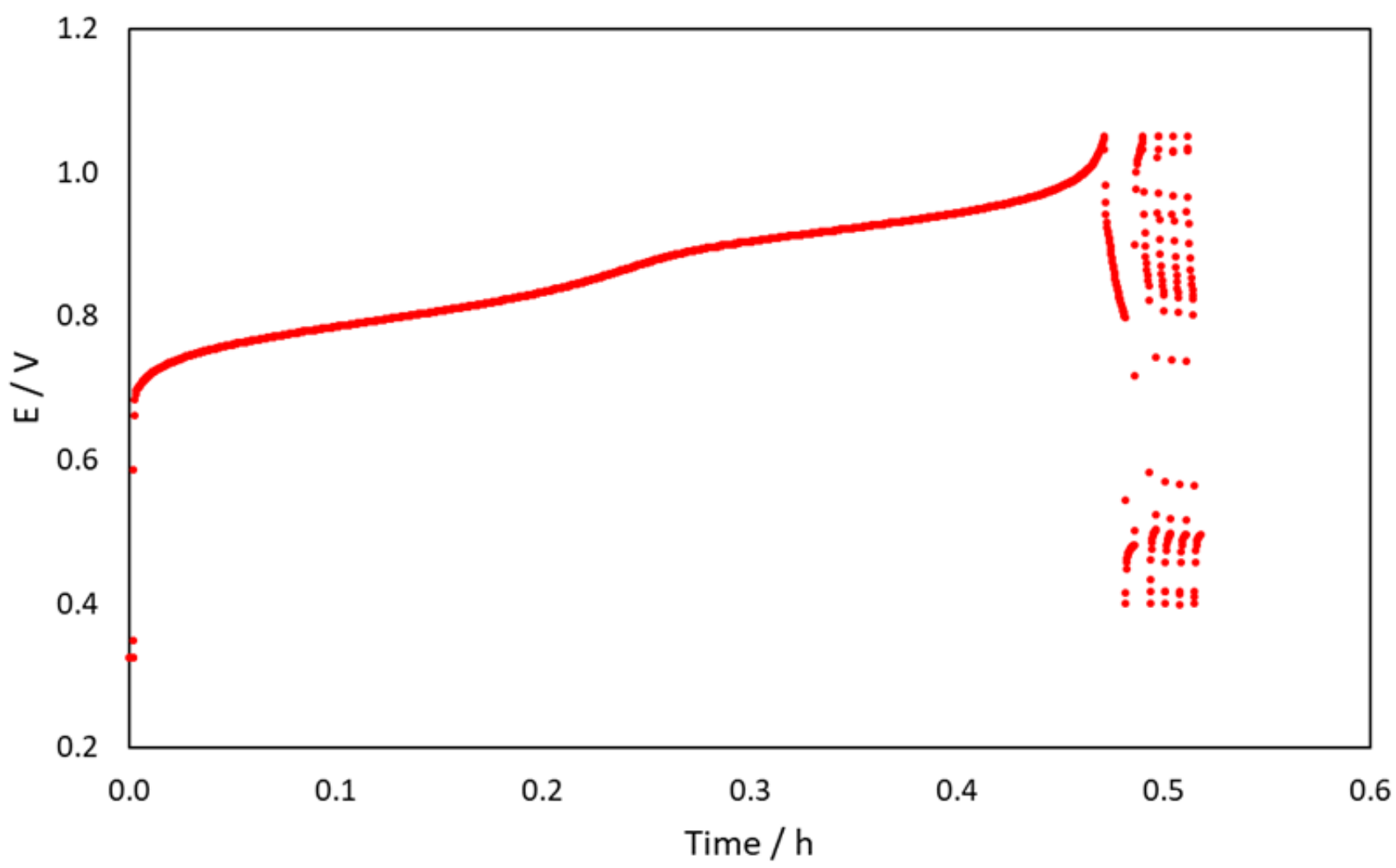

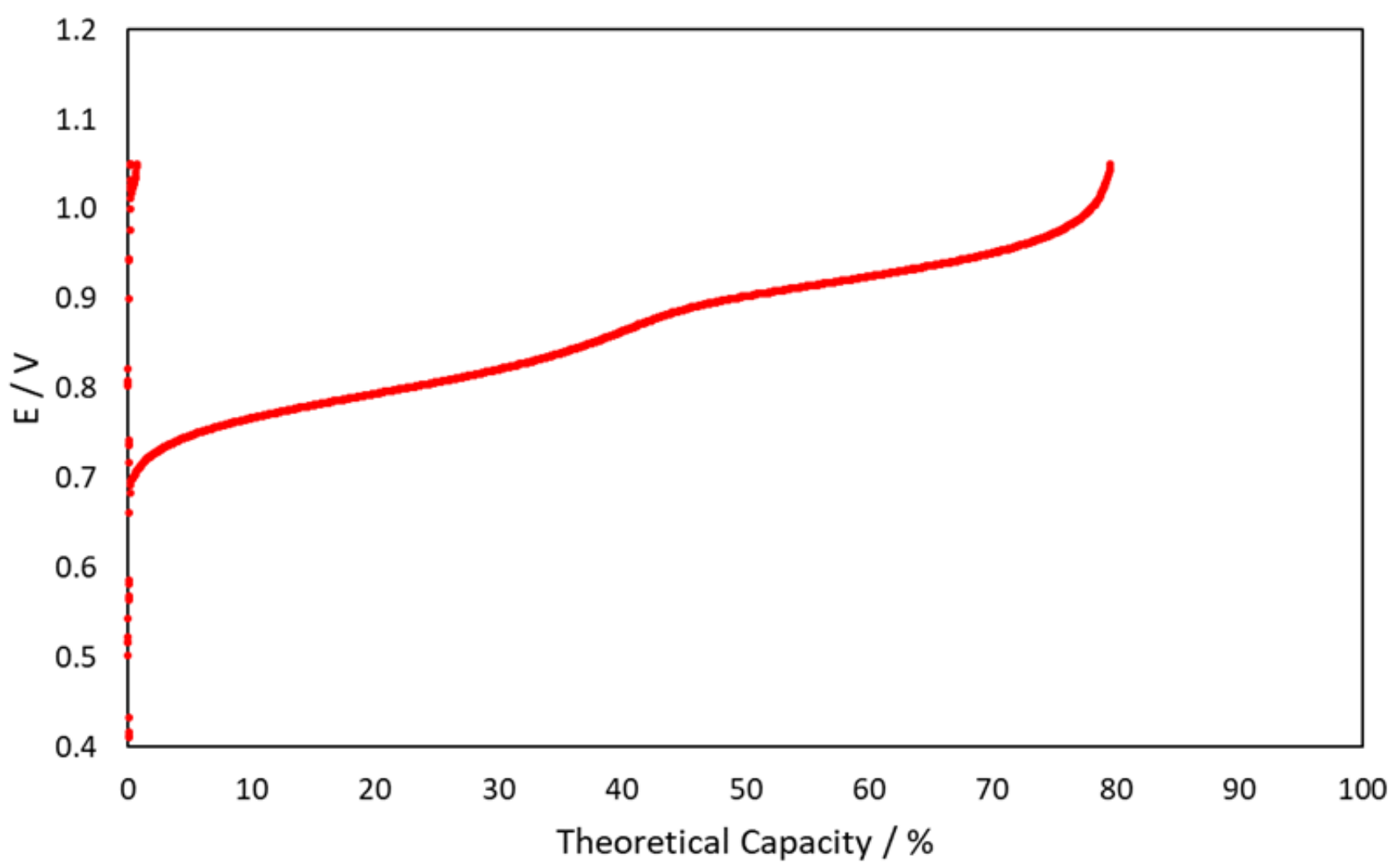

4.2.2 Battery in 2 M NaOH

Negolyte: 11 ml of 10 mM BenPy7 in 2 M NaOH
Posolyte: 25 ml of 10 mM BTMAP-Fc in 2 M NaOH

- Cut-off voltages: 0.40 – 1.05 V
- Cycling current: 2 mA cm^{-2}

Theoretical capacity expected for 2-electrons: 5.90 mAh

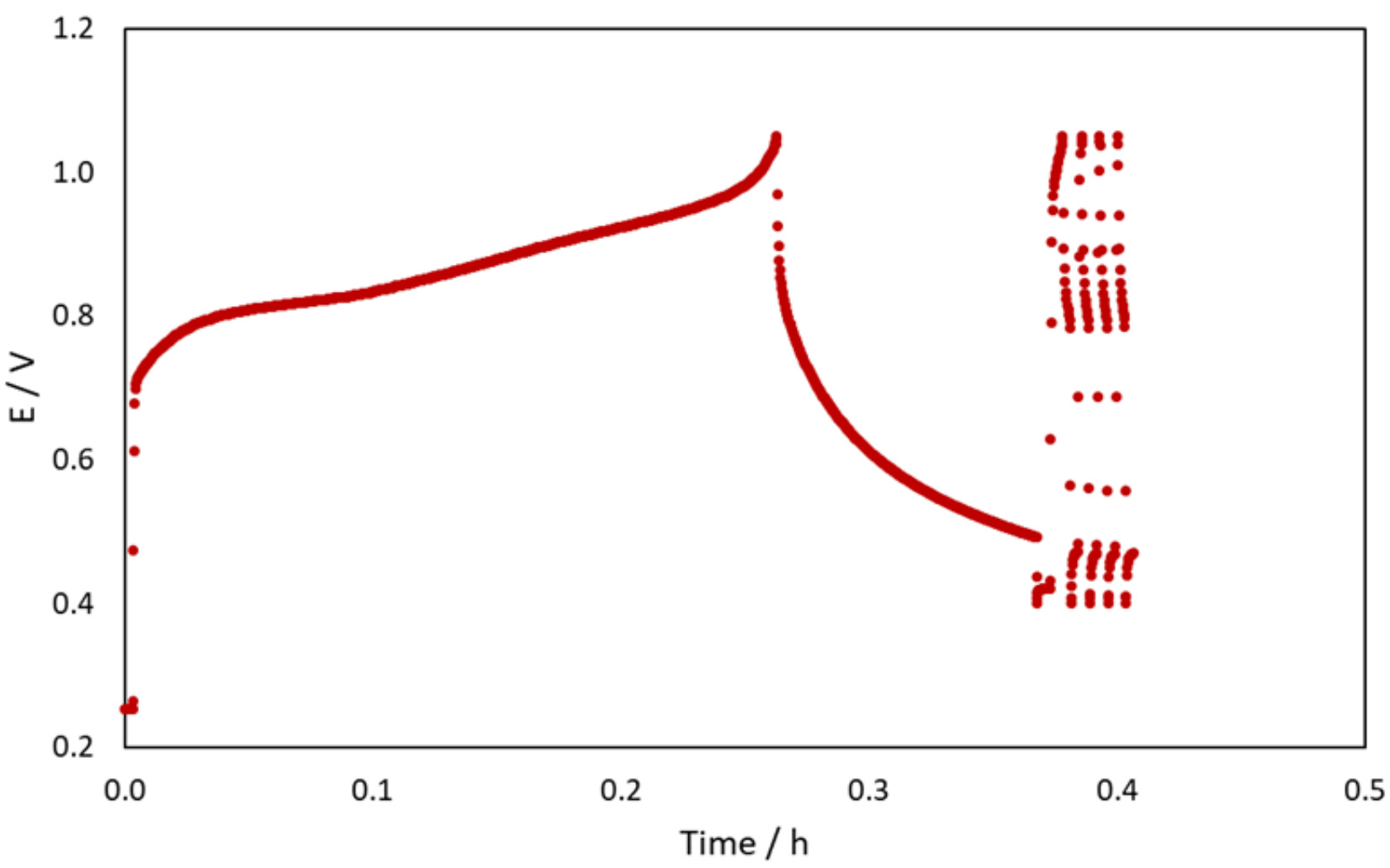

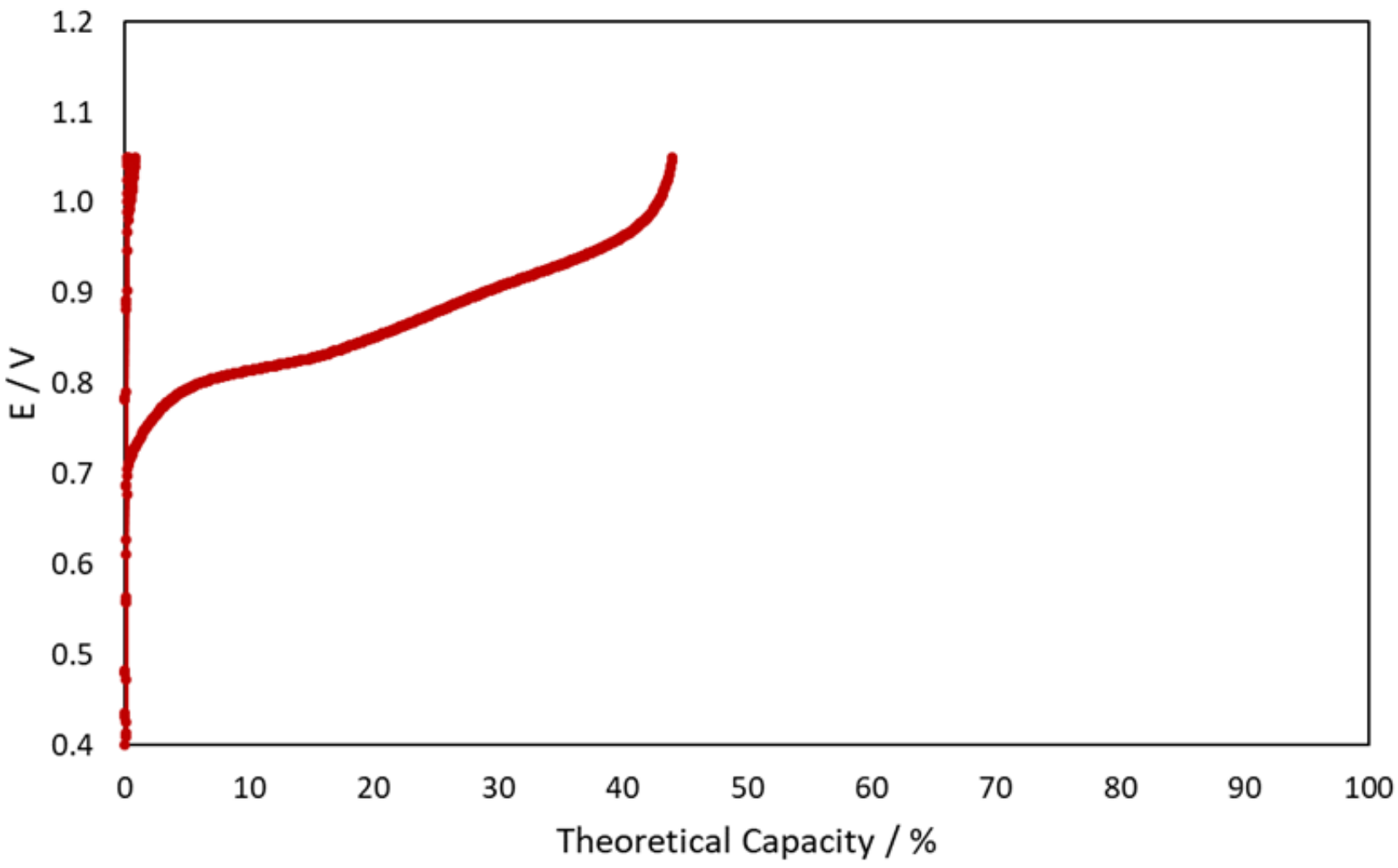

4.2.3 Battery in 1 M KCl pH 9.2

Negolyte: 11 ml of 10 mM BenPy7 in 1 M KCl (pH adjusted to 9.2)
Posolyte: 25 ml of 10 mM BTMAP-Fc in 1 M KCl (pH adjusted to 9.0)

- Cut-off voltages: 0.40 – 1.10 V
- Cycling current: 2 mA cm^{-2}

Theoretical capacity expected for 2-electrons: 5.90 mAh

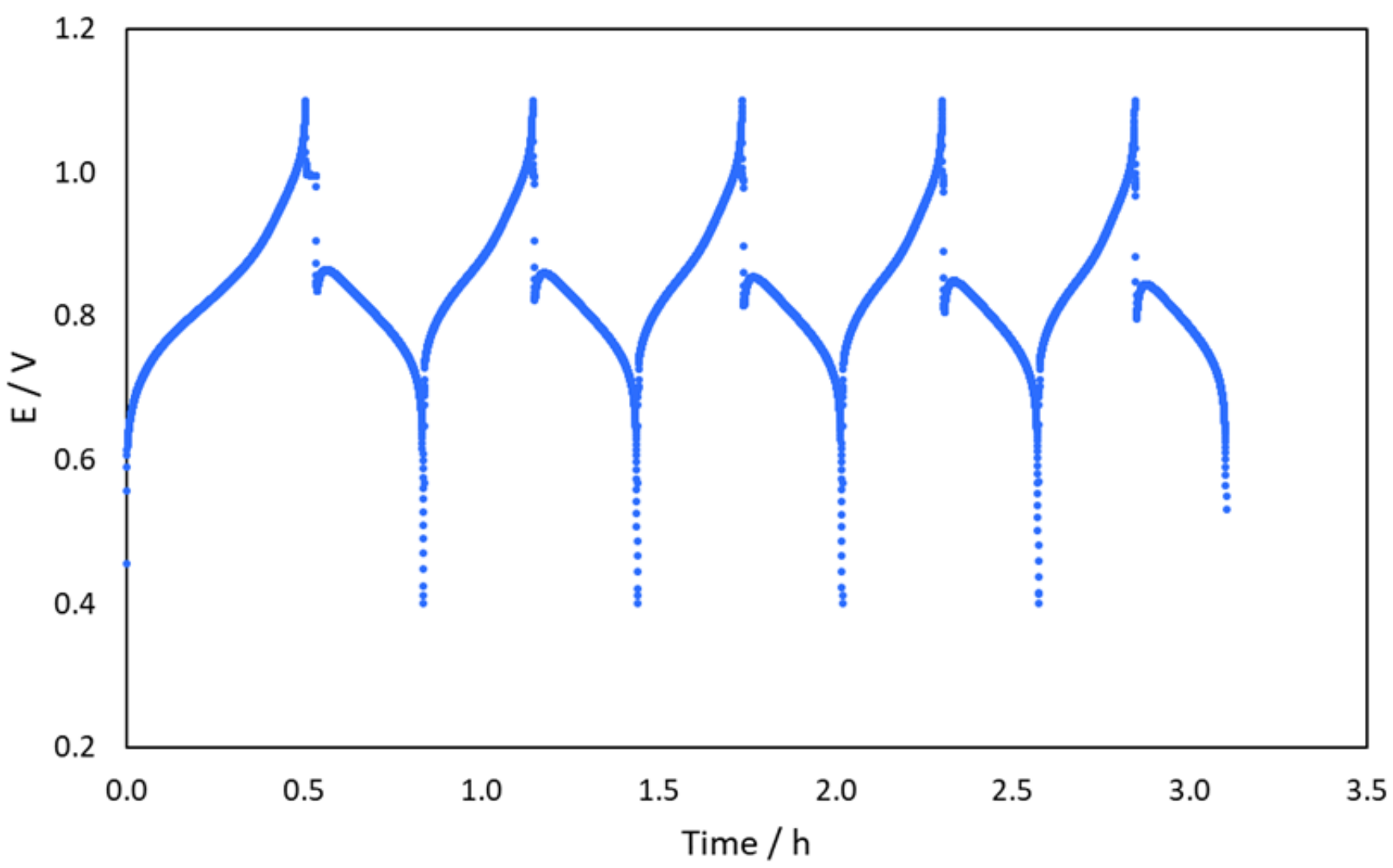

1.2
1.0
0.8
0.6
0.4
0.2
E / V
0.0
0.5
1.0
1.5
2.0
2.5
3.0
3.5
Time / h

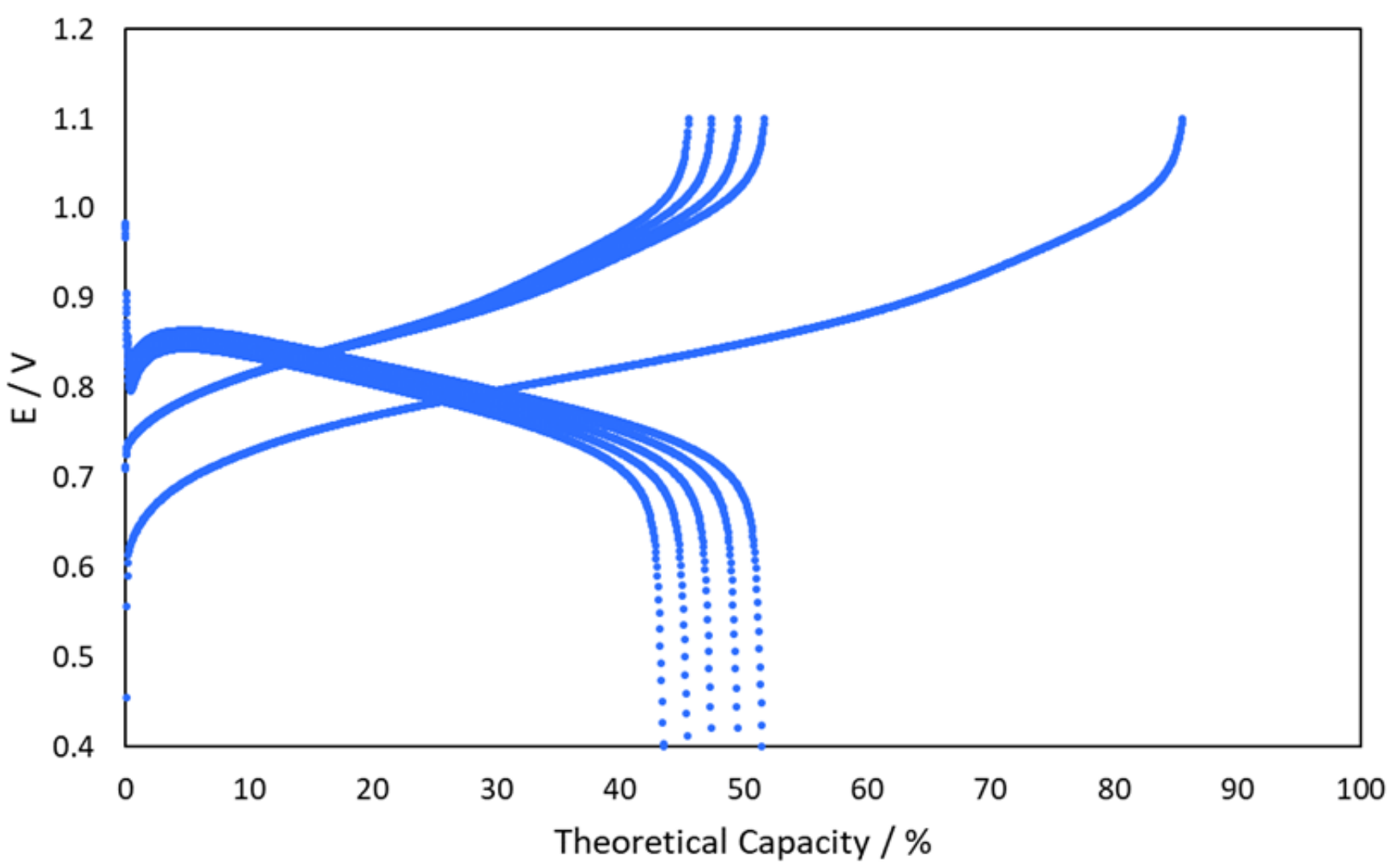

1.2
1.1
1.0
0.9
0.8
0.7
0.6
0.5
0.4
E / V
0
10
20
30
40
50
60
70
80
90
100
Theoretical Capacity / %

5 Maximum solubility measurements

The maximum solubility of **BenPy6** and **BenPy7** in water was determined using UV-Vis measurements. The tests were performed using a spectrophotometer Specord 200 (from Analytik Jena). First, a calibration curve was built up using low concentrations of the materials (from 0.01 to 0.1 mM). Then, a maximum solubility sample was prepared, adding a known amount of BenPy and adding small quantities of water until all the material was clearly dissolved. Finally, the maximum solubility sample was diluted in order to be in the range of the calibration curve. The maximum solubility was determined using the calibration curve and multiplying by the dilution factor. The UV-Vis spectra and calibration curves for **BenPy6** and **BenPy7** are presented below in the corresponding sections, 5.1 and 5.2, respectively.

5.1 BenPy6

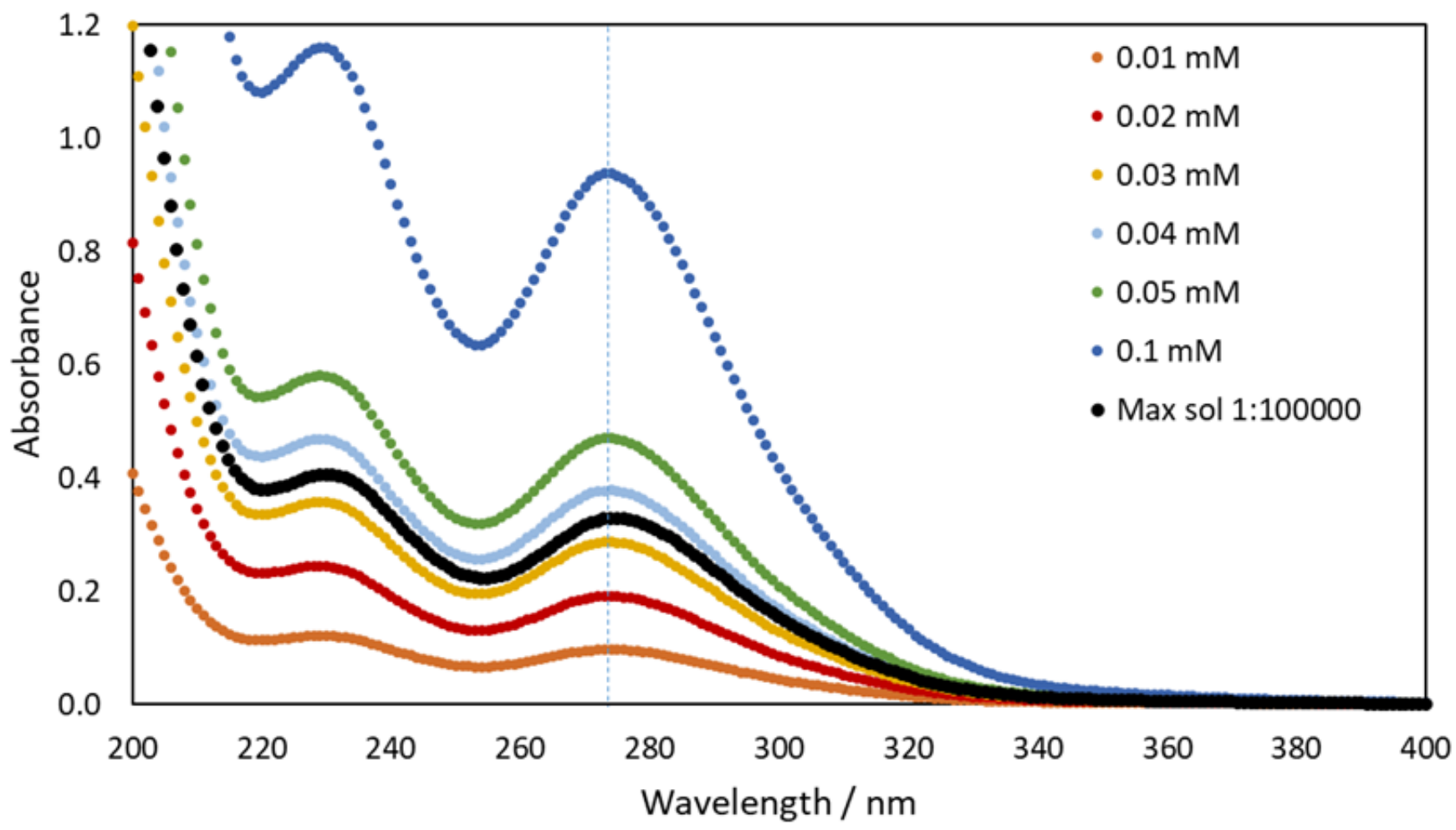

The calibration curve was built using the absorbance value at 264 nm. The maximum solubility sample (red star) was diluted 1:100000.

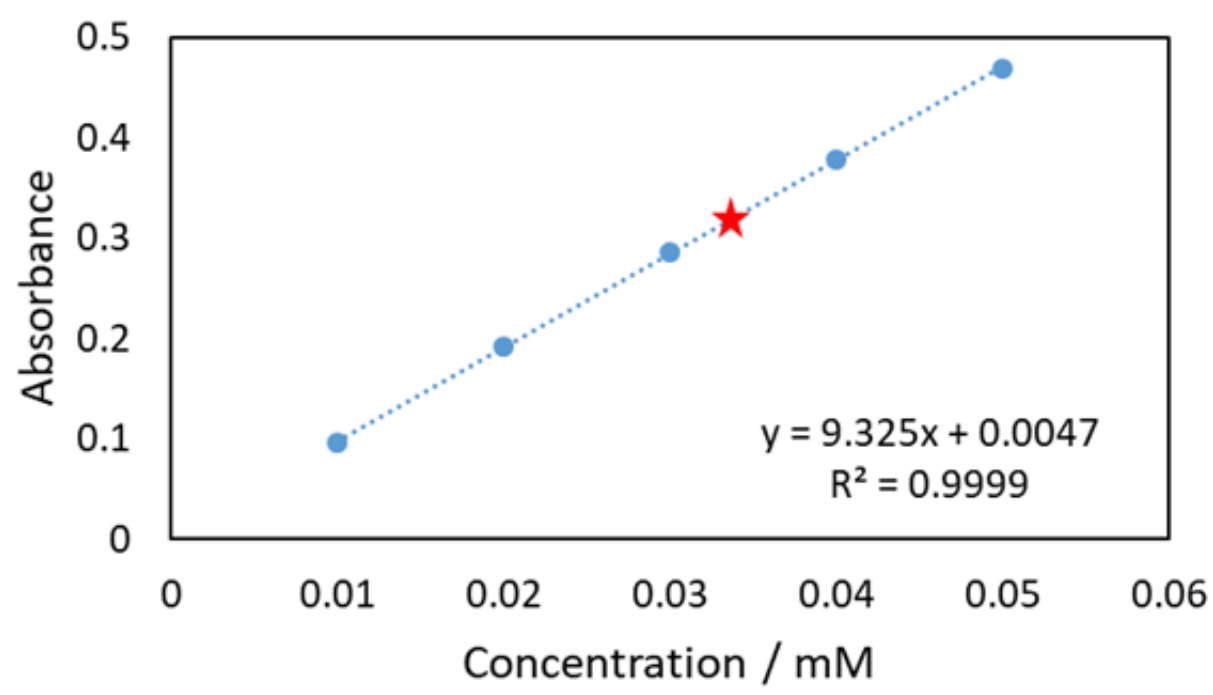

The maximum solubility of **BenPy6** in water was calculated as 3.5 M.

5.2 BenPy7

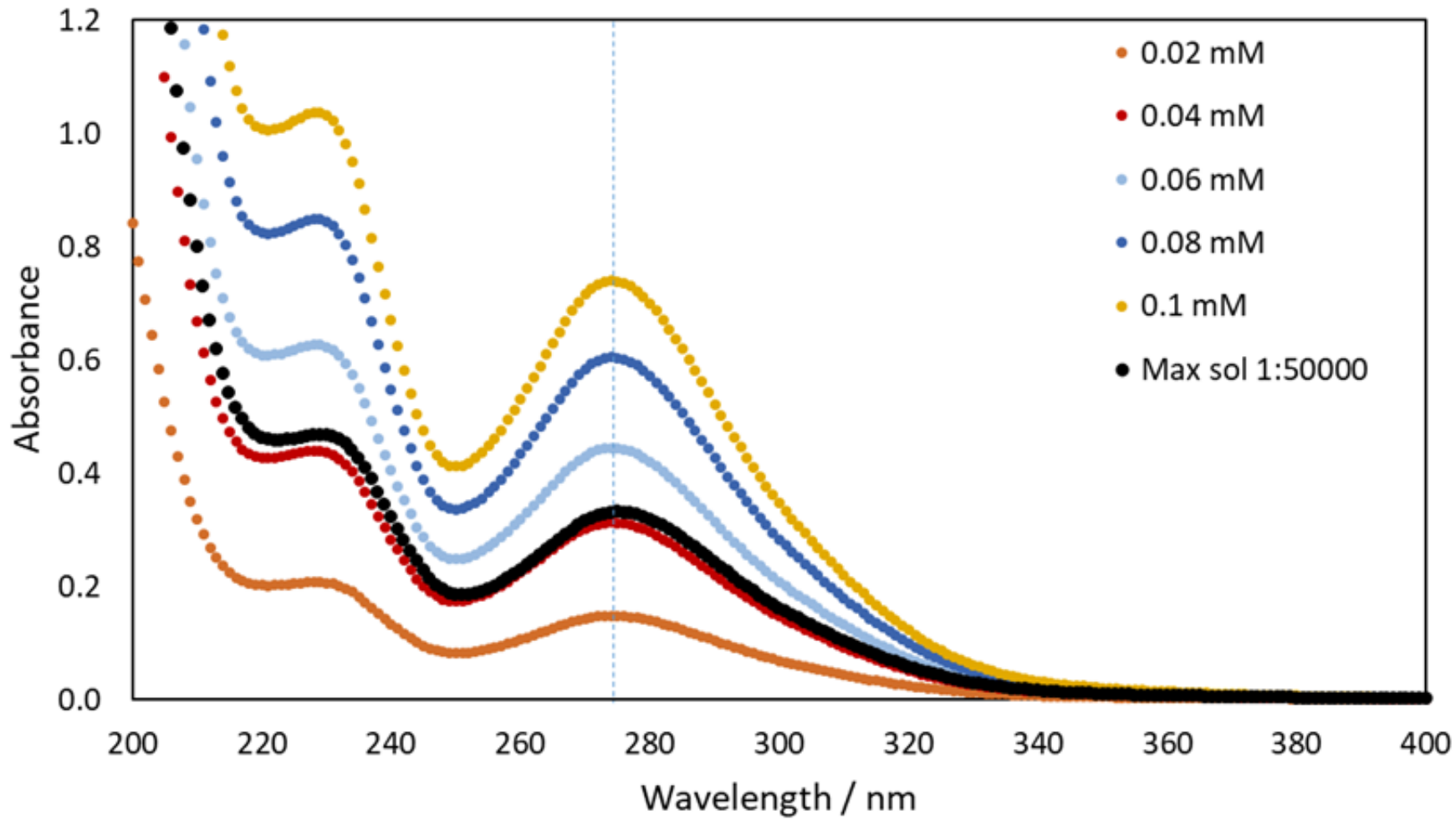

The calibration curve was built using the absorbance value at 274 nm. The maximum solubility sample (red star) was diluted 1:50000.

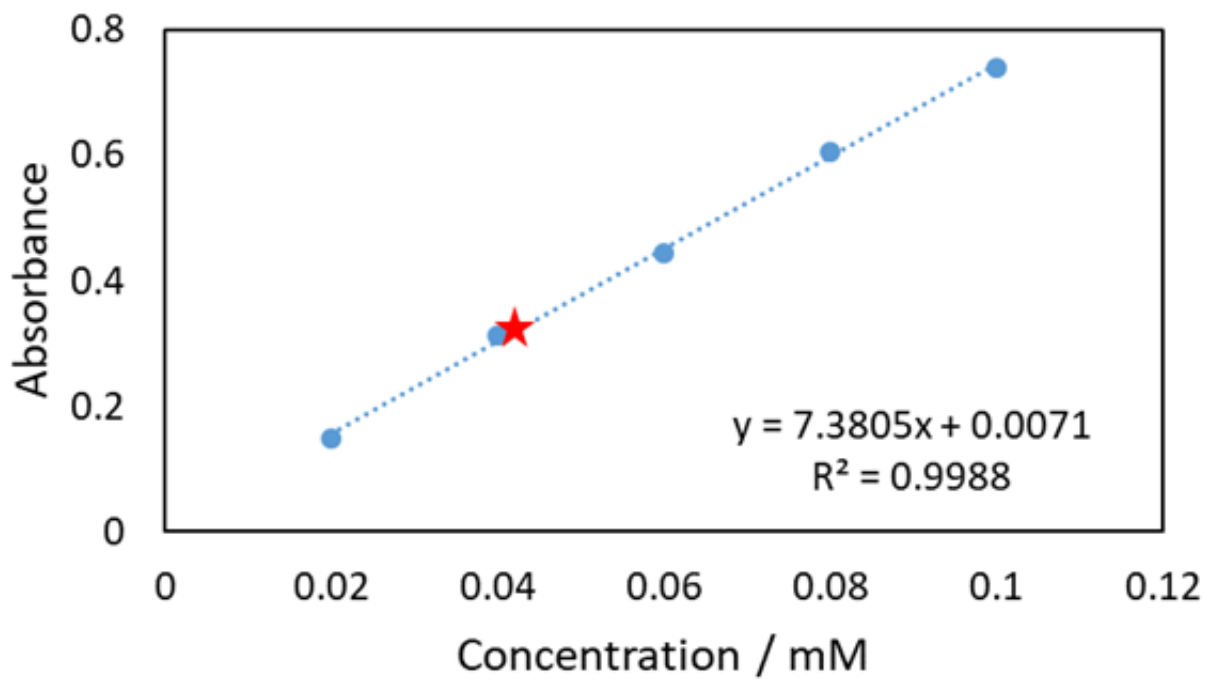

The maximum solubility of **BenPy7** in water was calculated as 2.2 M.

6 Stability tests - UV-Vis and NMR measurements

The stability of **BenPy6** and **BenPy7** in alkaline electrolytes was first studied by analyzing the change of colour of the solution over time after dissolving the material. For this study, different concentrations of NaOH were studied: 0.01, 0.1 and 2 M. Additionally, the corresponding azoniafluorenone derivatives **AZONE3** and **AZON3**, including hydroxiethyl and quaternary ammonium functional groups, respectively, were studied for comparison. All the samples were prepared using 5 mM of the active material dissolved in 3 ml of the supporting electrolyte. The picture below shows the evolution of the samples over time. From right to left: AZ3=AZON3, AZOH=AZONE3, BPy6=BenPy6 and BPy7=BenPy7.

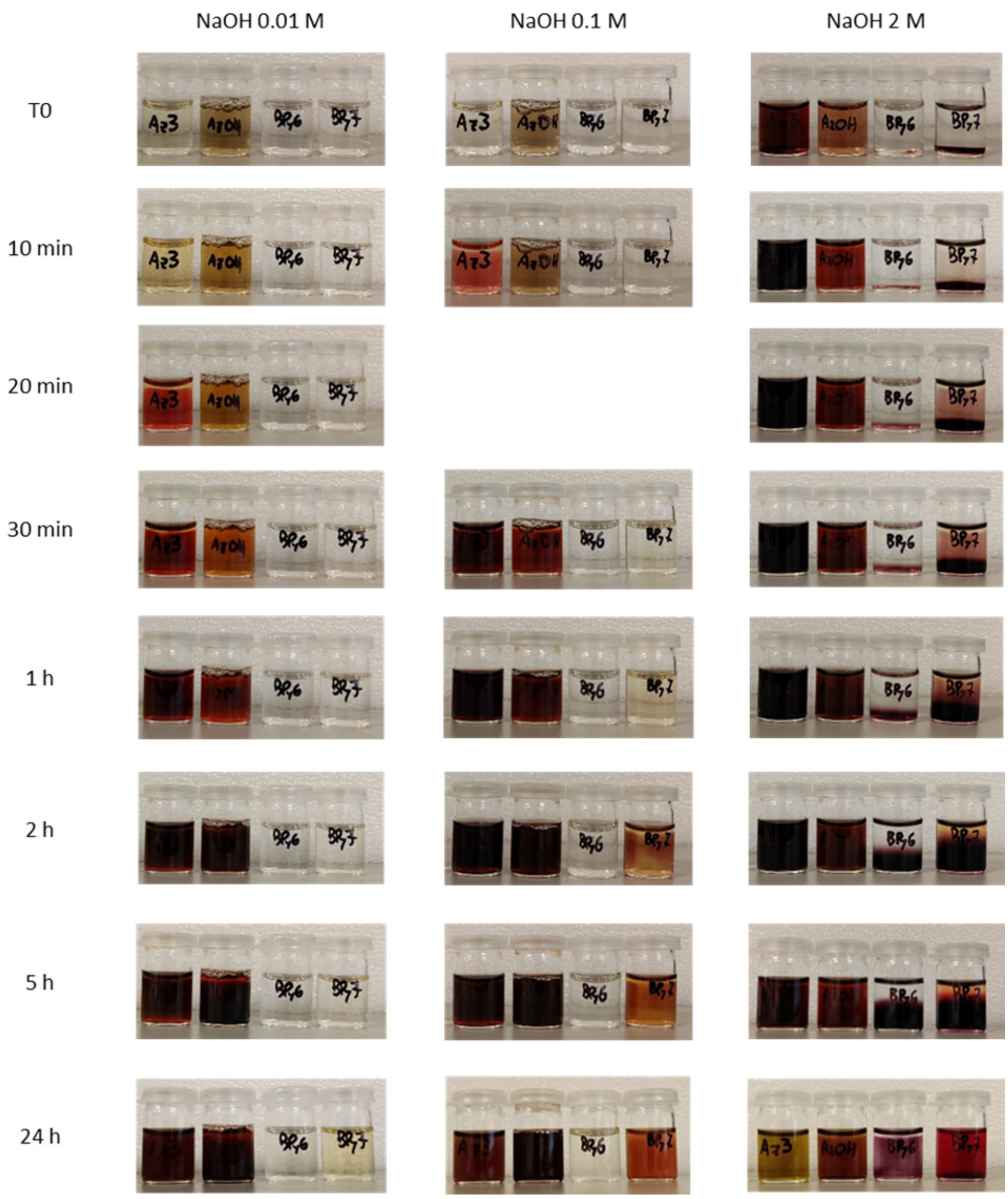

While **BenPy6** and **BenPy7** are stable in 0.01 M NaOH, both AZON derivatives decompose quite fast in this electrolyte, which evidences the lower stability of

azoniafluorenone structure in alkaline solutions. When increasing the alkalinity using 0.1 M NaOH, **BenPy7** starts showing traces of decomposition after 1 hour, while **BenPy6** remains stable for the whole duration of the test, confirming the higher stability conceded by the hydroxy group. Finally, 2 M NaOH seems to be too aggressive electrolyte even for **BenPy6** that slowly decomposes in that condition.

Next, we studied the stability of **BenPy6** and **BenPy7** via UV-Vis measurements. The absorbance spectra was measured using a spectrophotometer Specord 200 plus (from Analytik Jena). The samples for the absorbance measurements were taken from the electrolytes used during the battery studies, with a concentration of the active material of 10 mM in the studied supporting electrolytes. The samples were diluted for the UV-Vis measurements (in the case of the reduced forms, the samples were diluted inside the glovebox to avoid contact with oxygen).

The absorbance spectra of the oxidized form of **BenPy7** after dissolving the material in highly alkaline electrolytes shows a new peak in the wavelength of 510 nm, similar to **AZON3**, indicating the formation of side products under alkaline conditions. To identify the nature of the decomposition products, we examined the decay of **BenPy7** in 0.1 M NaOD by ^{1}H NMR (see Supporting Information S.6.2.4). The ^{1}H NMR spectra indicate the presence of an allylic fragmentation product (see Scheme S2), although most of the mass balance is composed of oligomeric/polymeric material.

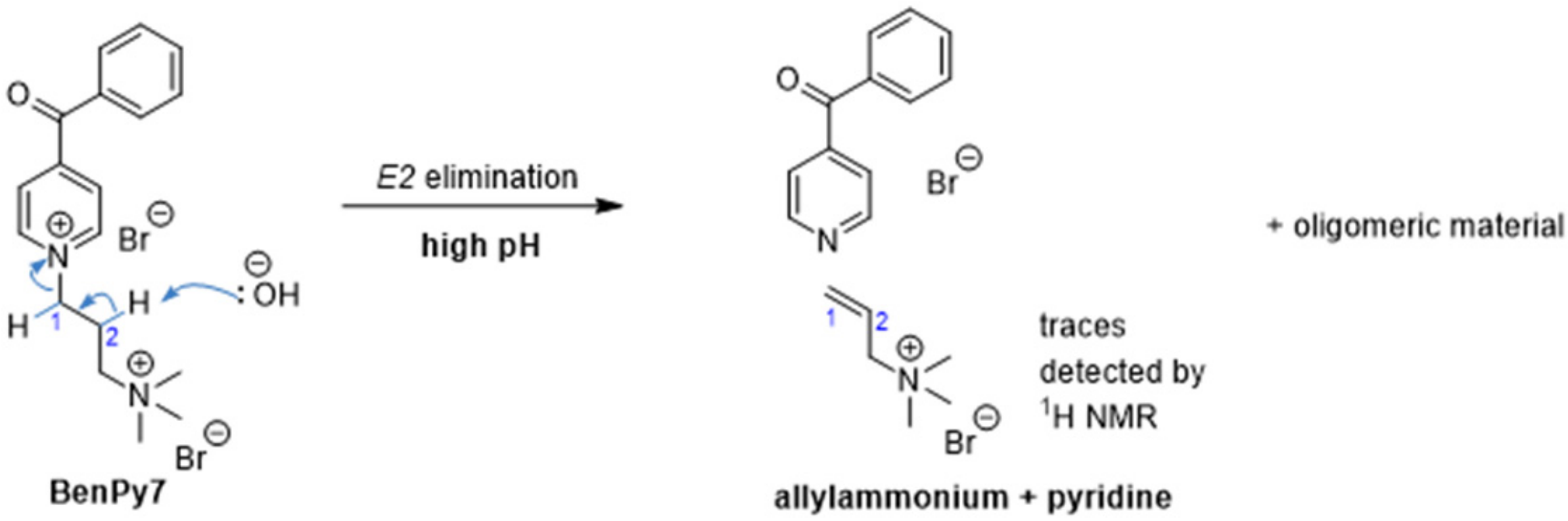

Scheme S2. Decomposition of **BenPy7** in alkaline media.

6.1 BenPy6

The batteries of **BenPy6** in 0.1 and 2 M NaOH were studied. In this case, one drop of the sample was diluted in the same supporting electrolyte in order to perform a qualitative analysis of the resulting spectra. The curves were then normalized (peak at 220-224 nm) to ease the analysis.

6.1.1 Battery in 0.1 M NaOH

The samples analyzed were:

- **BenPy6-Ox – starting sample**: measured immediately after dissolving the material (oxidized form) in 0.1 M NaOH.
- **BenPy6-Ox – after 5 hours**: measured after 5 hours of dissolving the material (oxidized form) in 0.1 M NaOH.
- **BenPy6-Red – first cycle**: reduced form of **BenPy6** electrogenerated in the flow cell after the first cycle charging step (after 2-electron reduction).
- **BenPy6-Ox – after cycling**: measured after cycling (oxidized form).

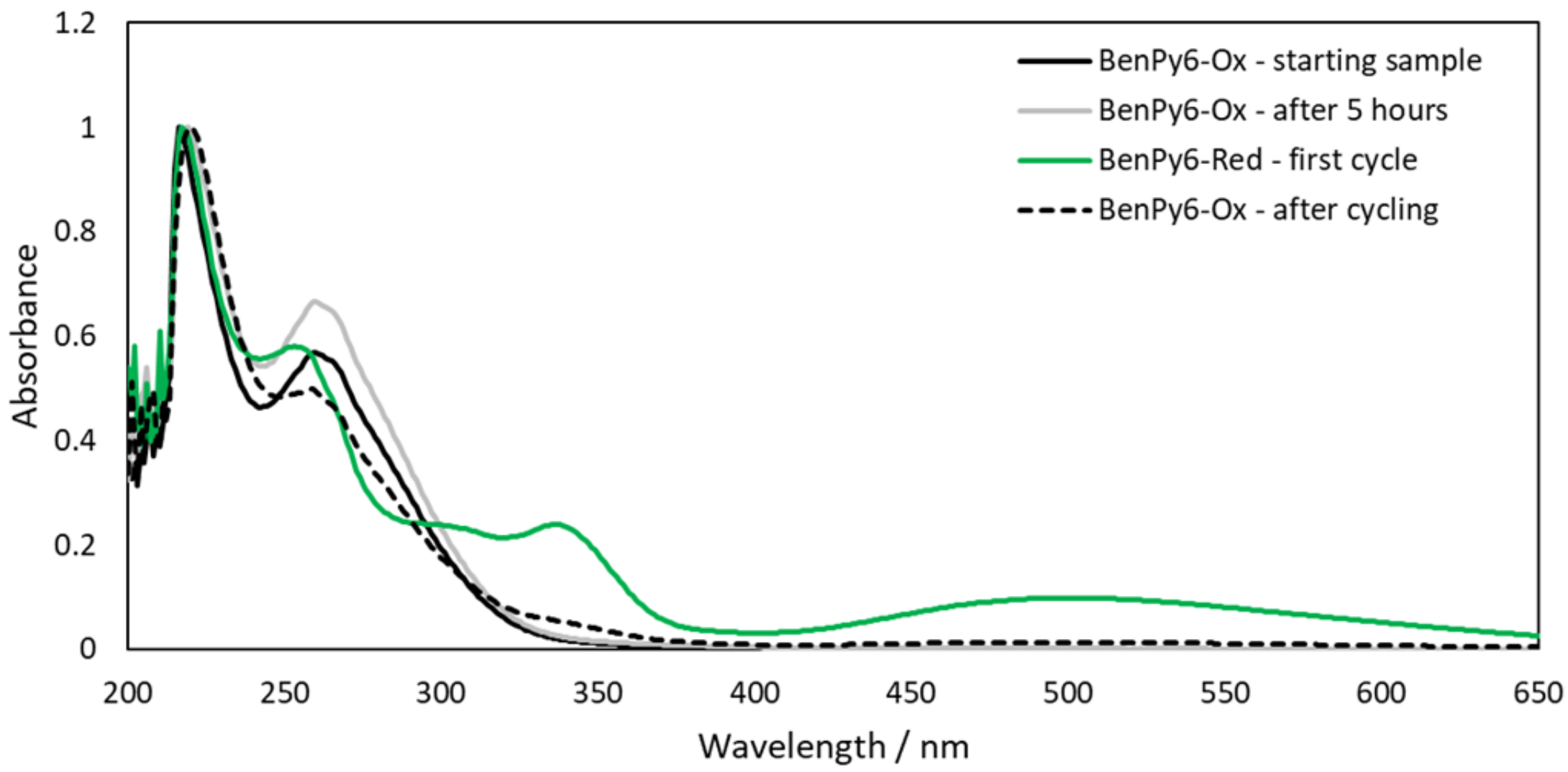

The spectra of the starting sample measured immediately and after 5 hours of diluting **BenPy6** do not show clear differences nor new peaks that suggest decomposition of this material in 0.1 M NaOH. In the case of the spectra of the reduced form, it clearly shows the reduction of the material, presenting similarities with the curves we obtained when reducing the azoniafluorenone derivatives with a shift of the peak at 260 nm and new bands at 305, 340 and 500 nm.[11] Finally, the sample of the oxidized form after cycling shows a similar spectra than the oxidized form samples with traces of the reduced form, which we believe appears as a result of not discharging the battery completely in the last cycle. To conclude, this analysis did not evidence any clear decomposition of **BenPy6** in the studied media, resulting neither from the oxidized nor the reduced form.

6.1.2 Battery in 2 M NaOH

The samples analyzed were:

- **BenPy6-Ox – starting sample**: measured immediately after dissolving the material (oxidized form) in 2 M NaOH.
- **BenPy6-Ox – after 5 hours**: measured after 5 hours of dissolving the material (oxidized form) in 2 M NaOH.

- **BenPy6-Red – first cycle**: reduced form of **BenPy6** electrogenerated in the flow cell after the first cycle charging step (after 2-electron reduction).
- **BenPy6-Red – after cycling**: measured after cycling (2-electron reduced form).

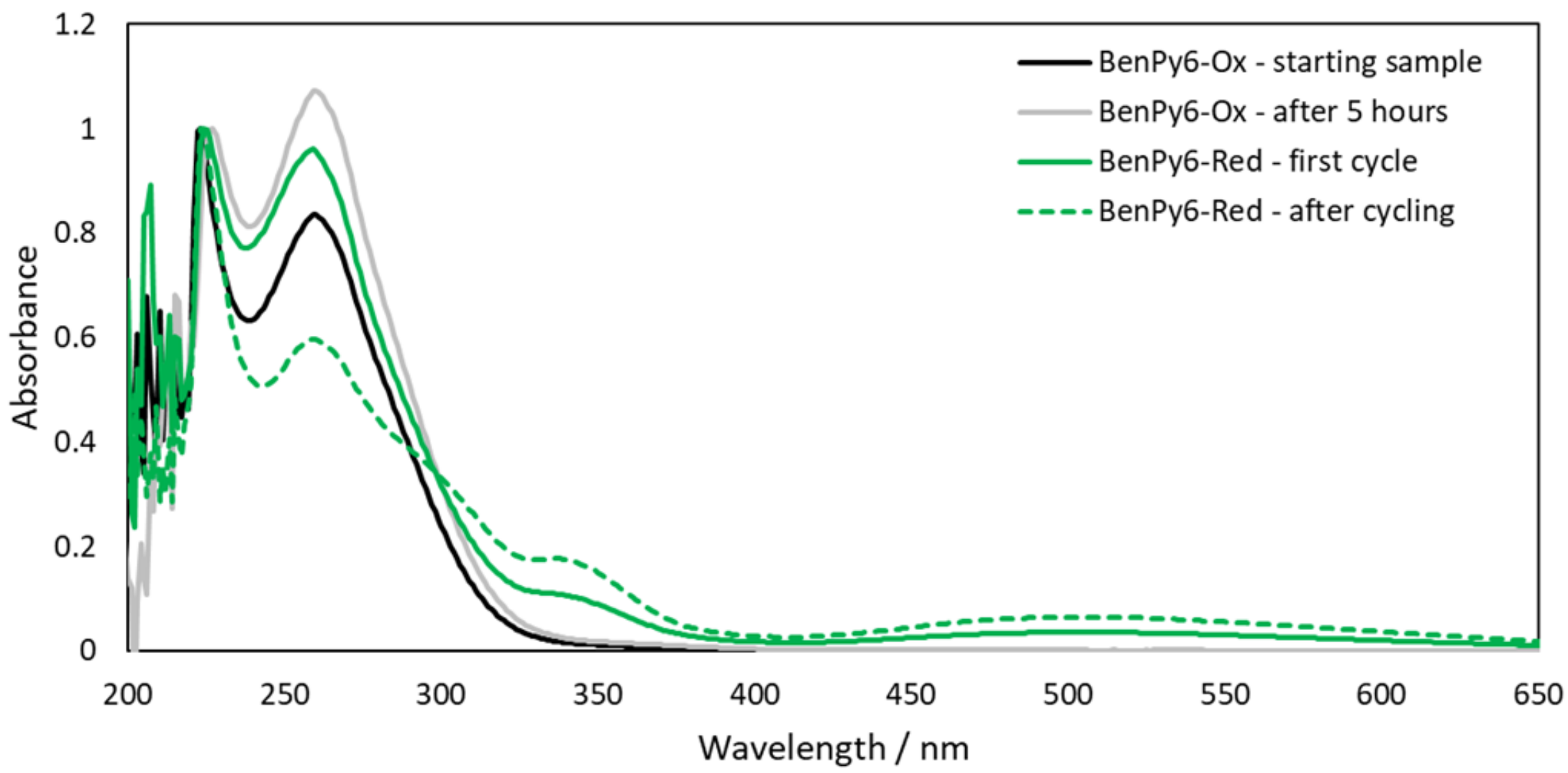

The spectra of the starting sample measured immediately and after 5 hours of diluting **BenPy6** do not show clear differences nor new peaks that suggest decomposition of this material in 2 M NaOH. In the case of the spectra of the reduced form, it clearly shows the reduction of the material with new peaks at 305, 340 and 500 nm. Finally, a similar spectra is observed for the sample of the reduced form after cycling. To conclude, this analysis did not evidence any clear decomposition of **BenPy6** in the studied media, resulting neither from the oxidized nor the reduced form.

6.2 BenPy7

The batteries of **BenPy7** in 0.1 and 2 M NaOH and 1 M KCl (pH 9.2) were studied. The samples for the UV-Vis analysis were prepared diluting 20 μl of the negolyte in 3.5 ml of the same supporting electrolyte. As we expect same concentration of the active material, in this case the curves were not normalized.

6.2.1 Battery in 0.1 M NaOH

The samples analyzed were:

- **BenPy7-Ox – starting sample**: measured immediately after dissolving the material (oxidized form) in 0.1 M NaOH.
- **BenPy7-Ox – after 3 hours**: measured after 3 hours of dissolving the material (oxidized form) in 0.1 M NaOH.

- **BenPy7-Red – first cycle**: reduced form of **BenPy7** electrogenerated in the flow cell after the first cycle charging step (after 2-electron reduction).

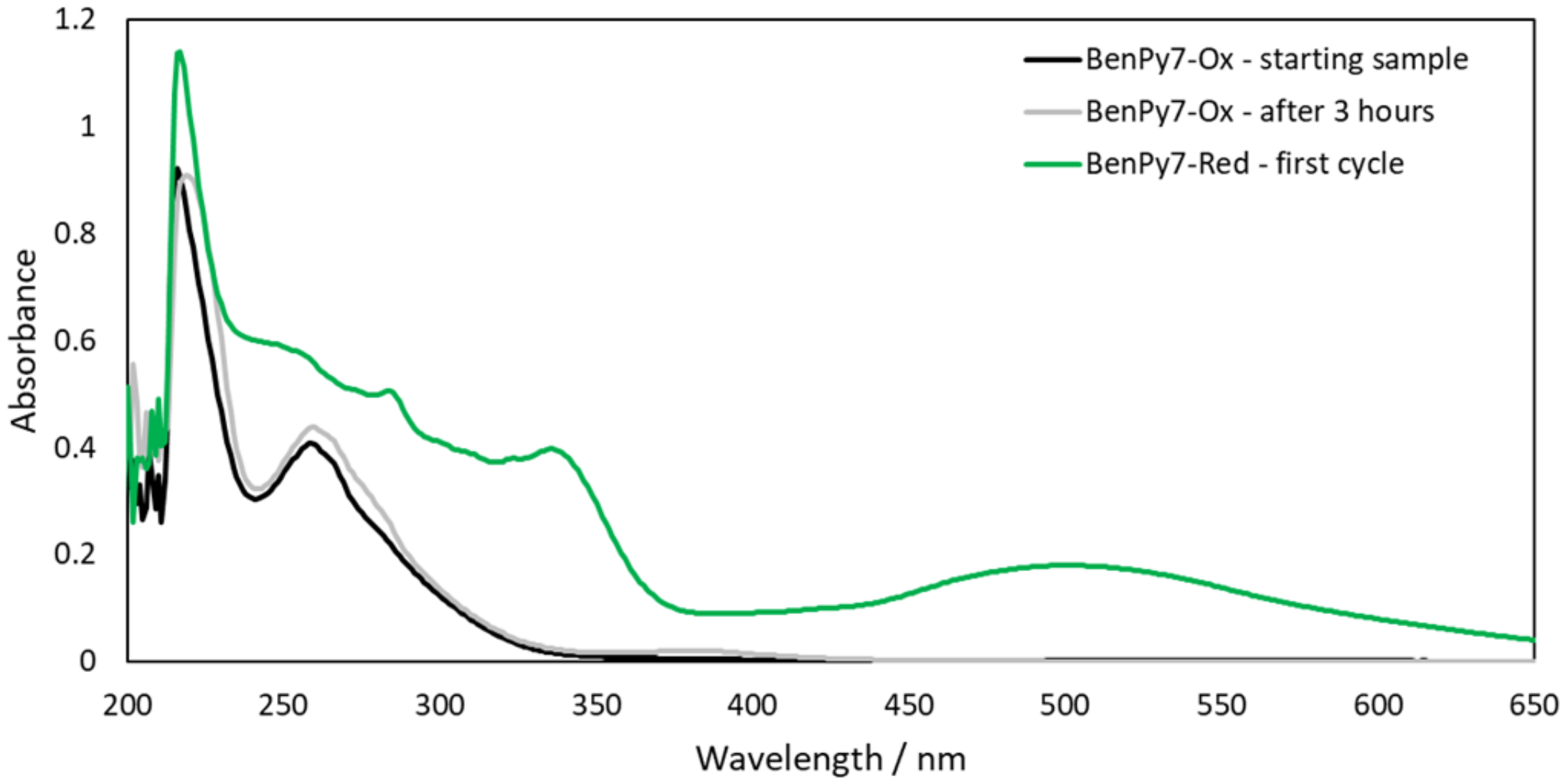

The comparison of the samples measured immediately and after 3 hours of dissolving the active material show similar spectra, although a new small peak at 360 nm is observed for **BenPy-Ox** after 3 hours, which suggests that the material is transitioning to another form as longer times are considered. In the case of the spectra of the reduced form, it clearly shows the reduction with new peaks appearing at 285, 340 and 500 nm, similar than the results obtained for **BenPy-6** and AZON3.

6.2.2 Battery in 2 M NaOH

The samples analyzed were:

- **BenPy7-Ox – starting sample**: measured immediately after dissolving the material (oxidized form) in 2 M NaOH.
- **BenPy7-Ox – after 3 hours**: measured after 3 hours of dissolving the material (oxidized form) in 2 M NaOH.
- **BenPy7-Red – first cycle**: reduced form of **BenPy7** electrogenerated in the flow cell after the first cycle charging step (after 2-electron reduction).

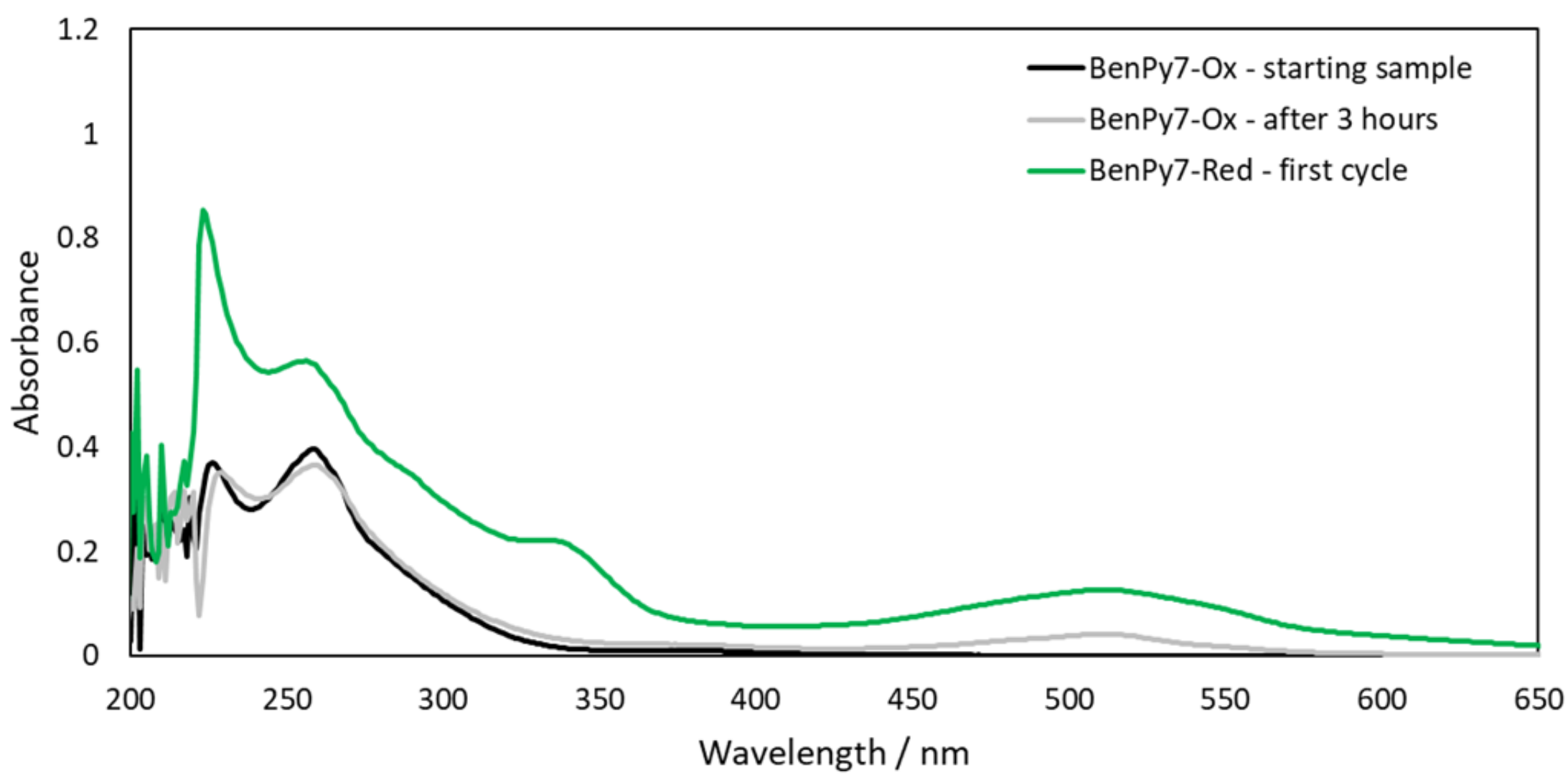

The spectra of **BenPy7** in 2 M NaOH as supporting electrolyte confirms the low stability of this derivative in highly alkaline media. When comparing the results of the oxidized form, we can see that the spectra of the sample measured after 3 hours clearly exhibits the small band at 360 nm, similar than in 0.1 M NaOH, besides a new large band at 510 nm. As we previously indicated in our work performed with azoniafluorenones,[11] we believe that, in highly alkaline media, the pyridinium derivatives are deprotonated at the N-CH_2 group to form a pyridinium ylide that presents radical character. The radical behavior of the resulting specie is confirmed by the new band at 510 nm, which agrees with the band in the range of 500-550 nm of the azoniafluorenone radicals presented in our previous work. Furthermore, this deprotonation of the pyridinium ring in basic conditions has been previously demonstrated by Liao.[18] Finally, the spectra of the reduced form evidences the reduction of **BenPy7** with a new peak appearing at 335 nm, besides the increment of the band at 510 nm (which can be attributed to either the reduction or further decomposition as a result of the high pH).

6.2.3 Battery in 1 M KCl pH 9.2

The samples analyzed were:

- **BenPy7-Ox – starting sample**: measured immediately after dissolving the material (oxidized form) in 1 M KCl.
- **BenPy7-Ox – after 3 hours**: measured after 3 hours of dissolving the material (oxidized form) in 1 M KCl.
- **BenPy7-Red – first cycle**: reduced form of **BenPy7** electrogenerated in the flow cell after the first cycle charging step (after 2-electron reduction).
- **BenPy7-Ox – after cycling**: measured after cycling (oxidized form).

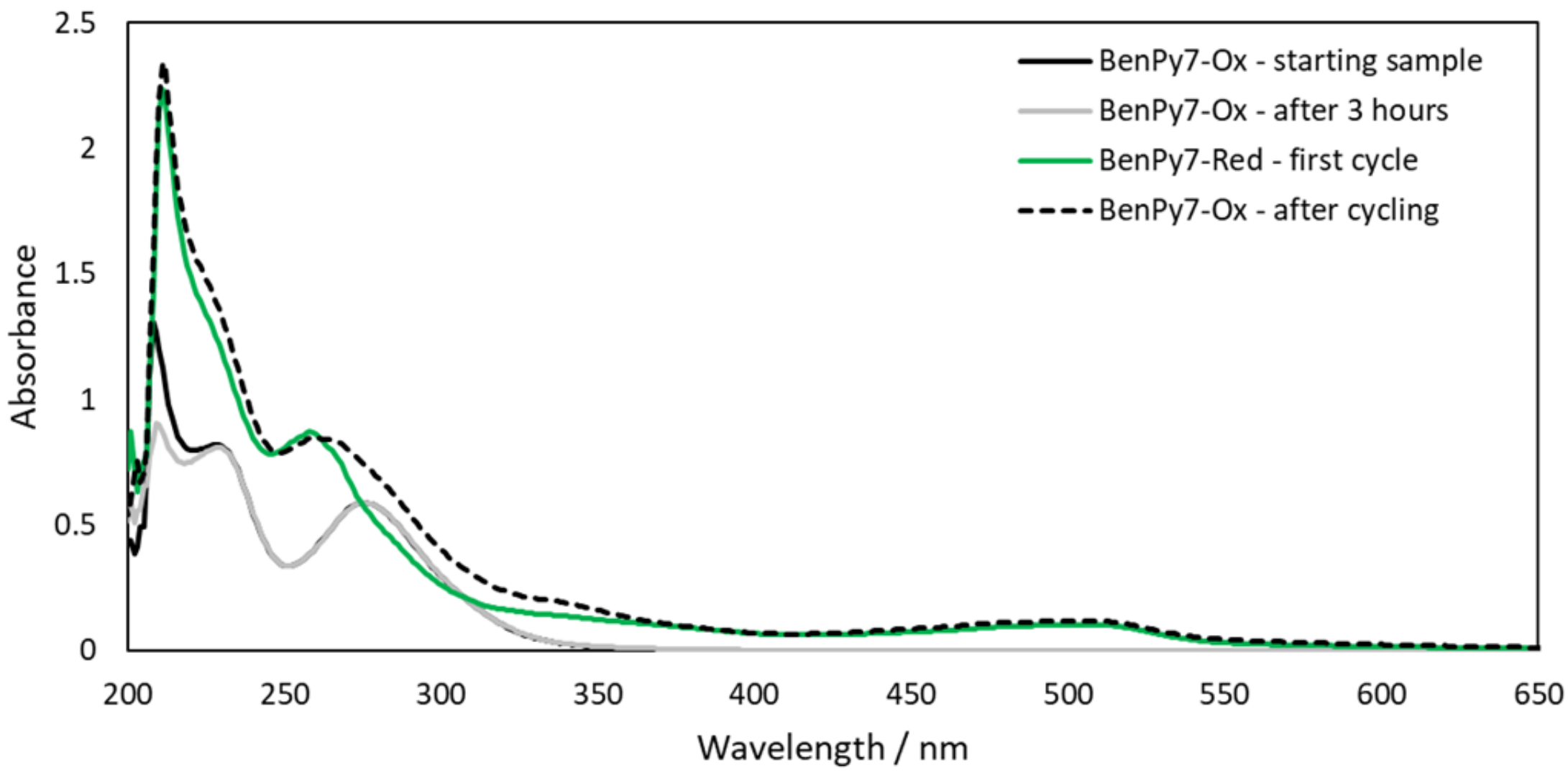

The spectra of the samples of **BenPy7** oxidized form in 1 M KCl (pH 9.2), confirm the higher stability of this derivative when considering electrolytes with lower pH, showing identical spectra without new peaks at increased exposure times. Furthermore, the spectra of the reduced form evidences the reduction after electrolysis, showing the shift of the peak at 275 nm and the new band at 510 nm. Finally, the sample of the oxidized form after cycling seems to be a mixture of the oxidized form, reduced form and decomposition product (the last accounts more than 60% of the material according to the battery cycling data).

6.2.4 NMR Measurements in 0.1 M NaOD

A sample with a 5 mM concentration of **BenPy7** in 0.1 M NaOD was prepared and left overnight for NMR analysis. ^{1}H and was performed on Bruker Avance-III 500 MHz spectrometer. The ^{1}H NMR spectra of BenPy7 indicates the presence of the allylic fragmentation product, but most of the mass balance is composed of oligomeric/polymeric material, as judged by the ^{1}H NMR spectra.

(BenPy7), ^{1}H NMR (NaOD, 500 MHz) spectrum

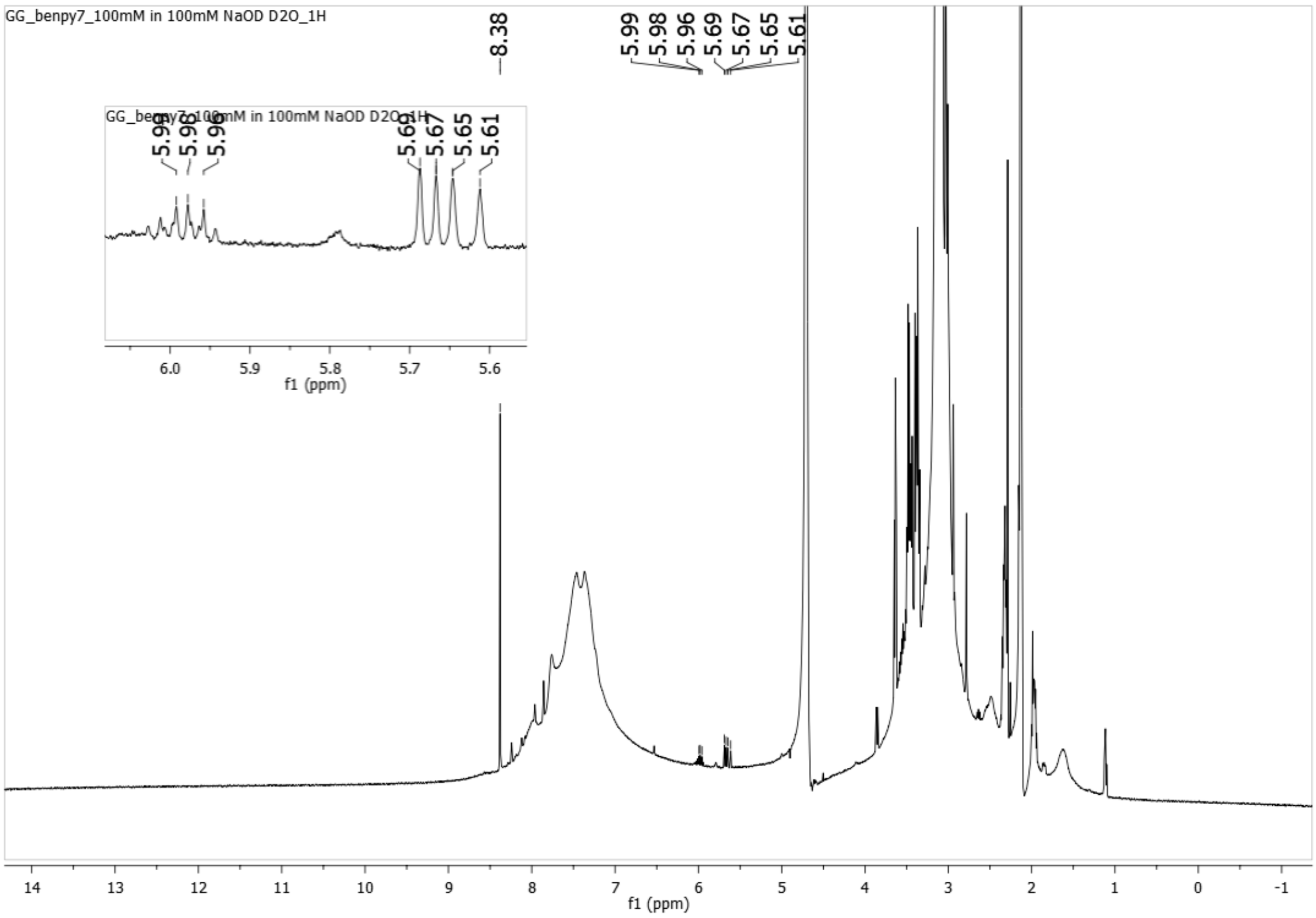

GG_benpy7_100mM in 100mM NaOD D2O_1H
8.38
5.99
5.98
5.96
5.69
5.67
5.65
5.61
f1 (ppm)

7 Pourbaix diagram and predominance diagram

The Pourbaix diagram was built from the CV measurements performed with **BenPy6** (1 mM) in Robinson-Britton buffer (0.12 M). The initial pH of the electrolyte was 7.15 and it was adjusted before each experiment with diluted potassium hydroxide (KOH). The measurements were performed at 100 mV s^{-1} with the same set-up and electrodes described in the section S3 of the SI (Cyclic Voltammetry Measurements). The potentials are reported against Ag/AgCl (3 M) reference. The species are named with the denomination provided in the square diagram, presented below, built in the computational studies (Supporting Information S8.2).

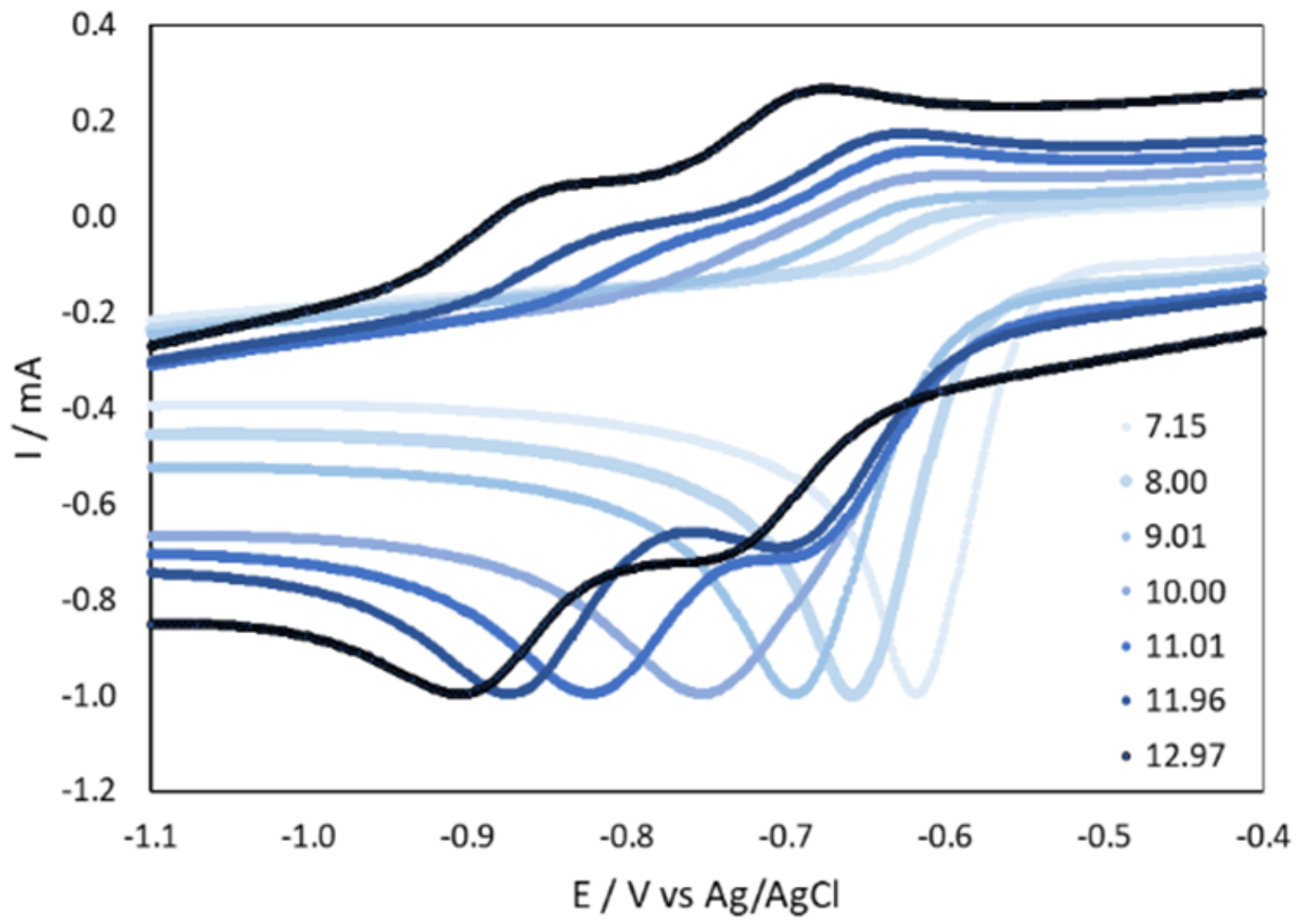

Figure S7.1. Cyclic voltammograms of BenPy6 using Britton-Robinson buffer at different pHs. Scan rate: 100 mV s^{-1}. Concentration of BenPy6: 1 mM. The current values were normalized using the reduction peak current.

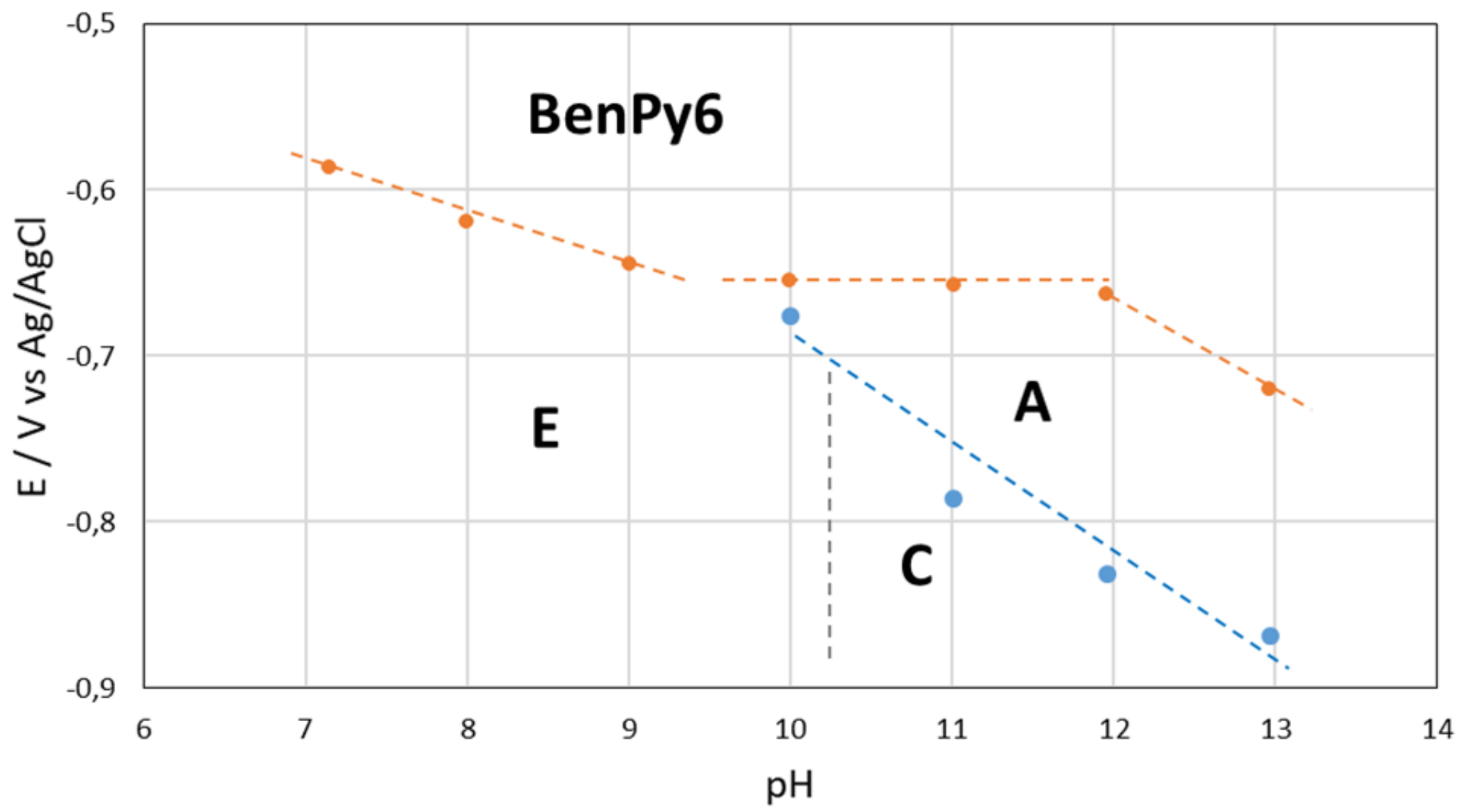

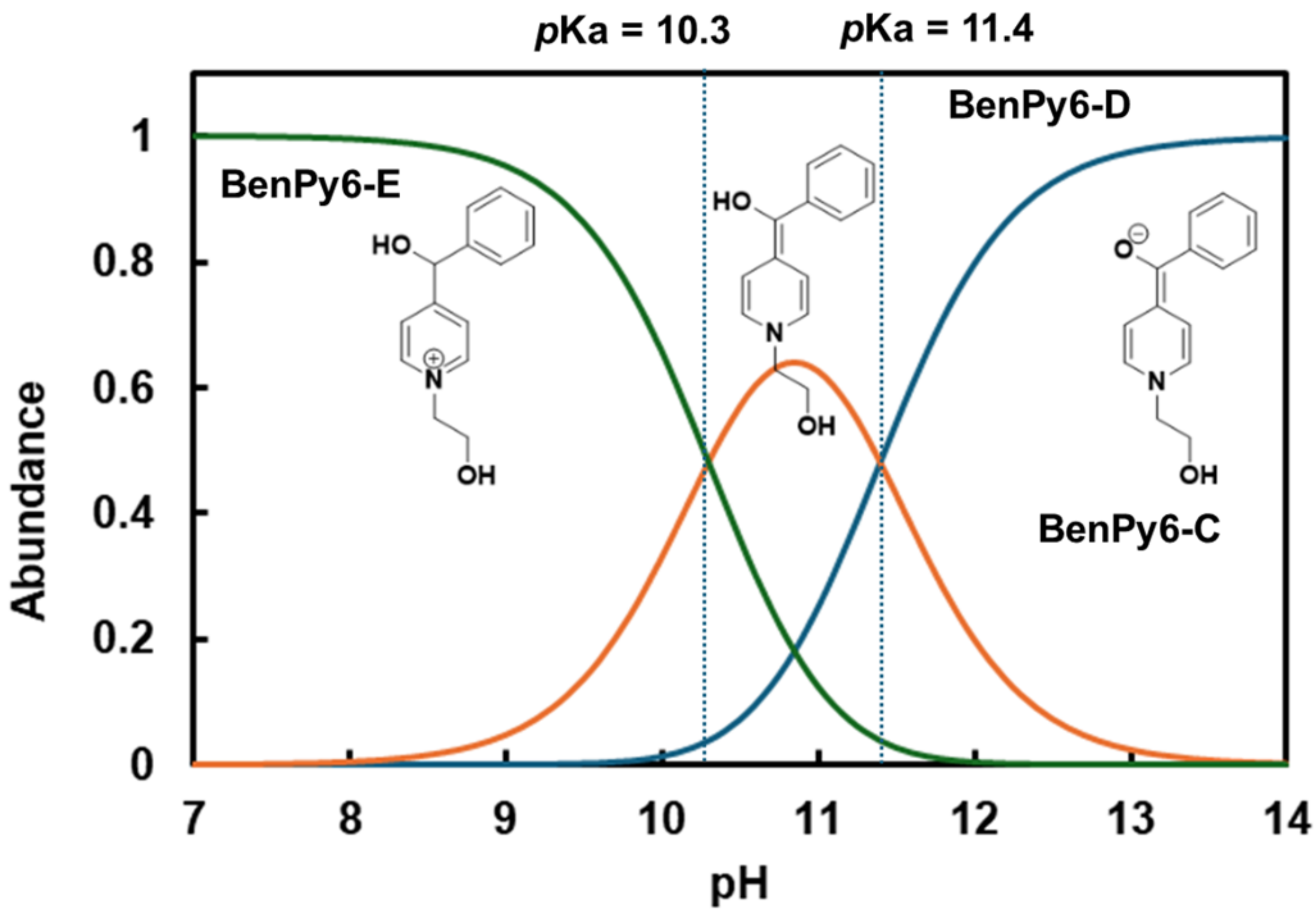

Figure S7.2 Pourbaix andPredomiance diagram of doubly reduced BenPy6 species as the function of pH calculated based on Henderson-Hasselback equation.

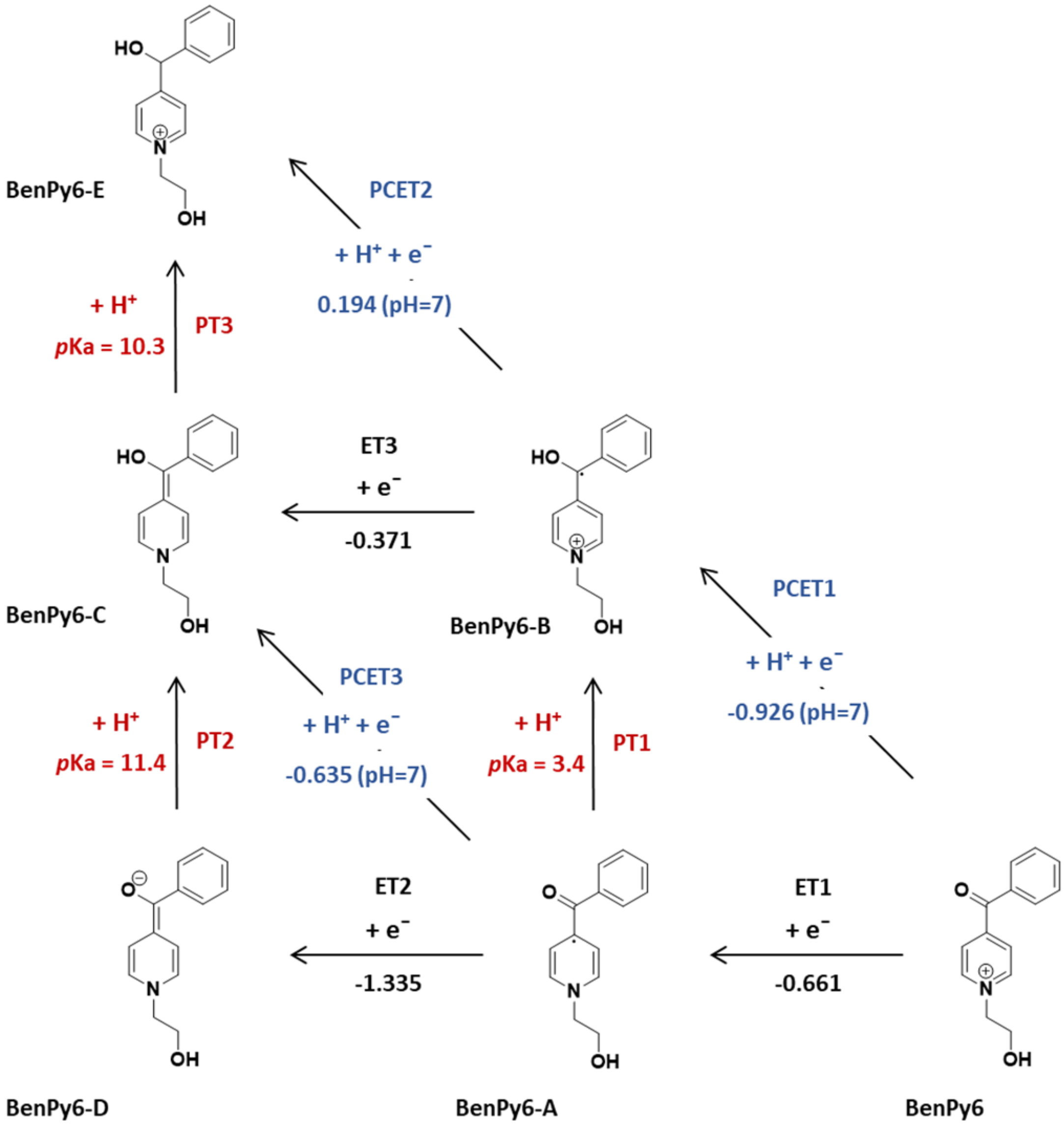

Scheme S3. Thermodynamic square scheme representing the electron transfer (ET), proton transfer (PT) and proton-coupled electron transfer (PCET) steps for the redox-active compound **BenPy6**. Horizontal arrows correspond to electron transfer steps and the numbers are standard one-electron reduction potentials (in V vs. Ag/AgCl). Vertical arrows indicate proton transfer steps labelled with pK_a values. Diagonal arrows represent concerted PCET reactions and the numbers are effective reduction potentials (in V vs. Ag/AgCl). All values were computed using DFT in solvent phase in standard conditions (T=298 K, *p*=1 atm, 1 M concentration and pH=0). Where applicable, pK_a and PCET values were adjusted to reflect *p*H-dependent free energy shifts.

8 Computational studies

8.1 Methodology

Density Functional Theory (DFT) calculations were performed for all relevant structures by using the M06-2X exchange-correlation functional[1] as implemented in the Gaussian 16 software.[2] Geometry optimizations were carried out with the 6-311G** basis set[3] water, modelled using the SMD implicit solvation model.[4] Vibrational frequency analysis were performed at the same level of theory within the rigid rotor harmonic-oscillator (RRHO) approximation to confirm the nature of the stationary points and to provide thermochemical corrections. Thermal and entropic contributions to the Gibbs free energies were computed under standard conditions (298.15 K and c=1 mol/dm^3). To improve the accuracy of the electronic energies, additional single point energy calculations were performed on the optimized structures with the larger 6-311++G** basis set. Conformational sampling of the compounds was performed by using the Conformer-Rotamer Ensemble Sampling Tool[5] implemented in the crest program.[6] For each compound, 5-7 structurally distinct conformers were selected and optimized using the DFT protocol described below. The lowest energy conformer was then used in subsequent thermochemical calculations.

The reported energies correspond to solvent-phase Gibbs free energies obtained as:

$$G = E_{lb} + \Delta G$$

where E_{lb} refer to the electronic energies computed at M06-2X/6-311++G** level of theory with the inclusion of solvent effects (SMD model, solvent = water) and ΔG involves the thermal and entropic effects calculated at M06-2X/6-311G** level also in solution (SMD model, solvent = water). These Gibbs free energies were employed in the thermodynamic calculations that support the electrochemical interpretations discussed in this work.

8.2 Electrochemical square representation and thermodynamic considerations

The reduction of compound BenPy6 to the product can occur via three elementary processes as also discussed in ref.[7]:

Electron transfer (ET):	$X + e^- \rightleftharpoons X^-$
Proton transfer (PT):	$X + H^+ \rightleftharpoons XH^+$
Proton-electron transfer (PCET):	$X + H^+ + e^- \rightleftharpoons XH$

Each of this steps plays a critical role in defining the reaction pathway and overall energetics.

1. Electron transfer (ET)

The electron transfer steps were modelled as one-electron reduction processes of the form:

$$X + e^{-} \rightleftharpoons X^{-}$$

The Gibbs free energy change associated with each ET step was computed from the solution-phase Gibbs free energies of the reduced and oxidized forms of the species as:

$$\Delta G^{ET} = G(X^{-}, aq) - G(X, aq)$$

where the free energy of the electron is referenced to the gas phase and set to $G(e^{-})$=0eV. The corresponding reduction potential was calculated according to the Nernst equation:

$$E_{red}^{ET} = -\frac{\Delta G^{ET}}{F} - E^{ref}$$

where F is the Faraday constant (F = 23.061 $\frac{kcal}{mol\,V}$) and E^{ref} = 4.491 V is the absolute potential of the reference electrode Ag/AgCl (3M KCl).[8]

2. Proton transfer (PT)

Proton transfer reactions were evaluated for each intermediate capable of accepting or donating a proton. The general form of these reactions is:

$$X + H^{+} \rightarrow XH^{+}$$

or its reverse, deprotonation:

$$XH^{+} \rightarrow X + H^{+}$$

The Gibbs free energy change for the protonation was computed as:

$$\Delta G_{PT}^{o} = G(XH^{+}, aq) - G(X, aq) - G(H^{+})$$

The free energy of the solvated proton (H^+) was not computed directly. Instead it was derived from the experimental acid dissociation constant of formic acid (pK_a=3.77)[9] using the equilibrium: $HCOOH \rightleftharpoons HCOO^{-} + H^{+}$. The standard Gibbs free energy change for this deprotonation is:

$$\Delta G^{o} = pK_a \cdot RTln(10)$$

where R is the universal gas constant, T is the temperature. Rearranging this expression and using computed free energies of formic acid and formate yielded:

$$G(H^{+}) = \Delta G^{o} + G(HCOOH) - G(HCOO^{-}) = -11.758\ eV$$

To compute pK_a values for the species in the redox pathway the deprotonation free energy ΔG_{deprot} was used:

$$\Delta G_{deprot} = G(X, aq) + G(H^{+}) - G(XH^{+}, aq)$$

The corresponding pK_a is given by:

$$pK_a = \frac{\Delta G_{deprot}}{RTln(10)}$$

A linear empirical correction was applied to account for known errors of the implicit solvent models:[10]

$$pK_a{}^{scaled} = 0.49 \cdot pK_a + 3.2$$

3. Proton-coupled electron transfer (PCET)

Proton-coupled electron transfer (PCET) involves the simultaneous transfer of a proton and an electron in a single concerted step. The general form of the reaction is:

$$X + H^+ + e^- \rightleftharpoons XH$$

The standard Gibbs free energy change for the PCET step is calculated using the gas-phase free energy of molecular hydrogen as a reference:

$$\Delta G^o_{PCET} = G(XH, aq) - G(X, aq) - \frac{1}{2}G(H_2, gas)$$

Here the $\frac{1}{2}G(H_2, gas)$ term approximates the combined free energy of a solvated proton and electron.[11] Molecular hydrogen was optimized in gas phase and its vibrational frequencies computed to obtain the thermal and entropic corrections.

The standard Gibbs free energy change was converted to standard a reduction potential:

$$E^{PCET}_{red} = -\frac{\Delta G^o_{PCET}}{F} - E'_{ref}$$

where *F* is the Faraday constant and $E'_{ref} = 0.210V$ is the potential of the reference electrode Ag/AgCl relative to the standard hydrogen electrode (SHE). At non-zero pH the potential is adjusted using the Nernst equation for $1e^-/1H^+$ process:

$$E^{PCET}_{red}(pH) = E^{PCET}_{red} - \frac{RT}{F}\ln(10) \cdot pH = E^{PCET}_{red} - 0.0592 \cdot pH$$

8.3 Square diagrams for AZON3 and BenPy7

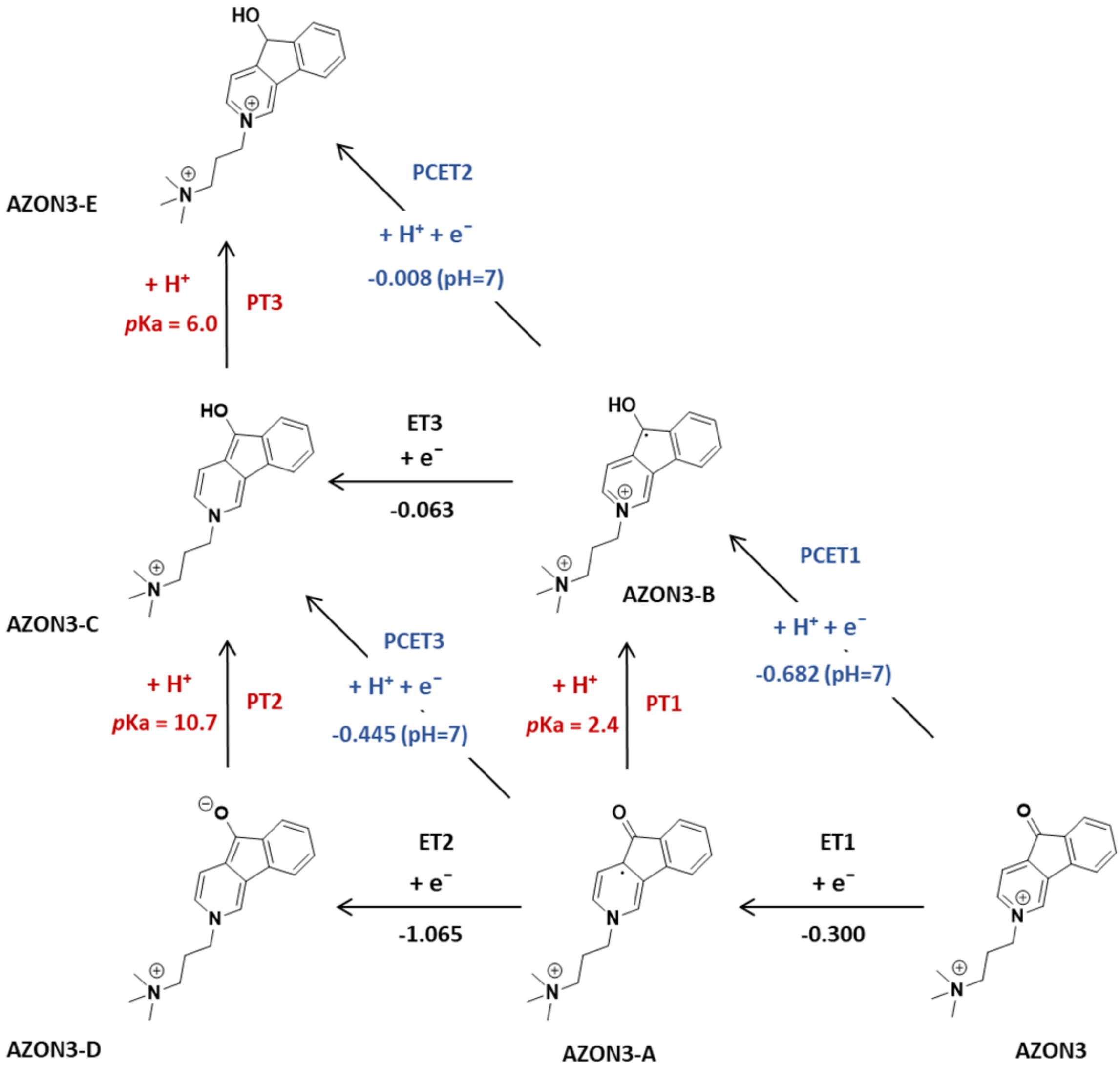

Figure S8.1: Thermodynamic square scheme representing the electron transfer (ET), proton transfer (PT) and proton-coupled electron transfer (PCET) steps for the redox-active compound **AZON3**. Horizontal arrows correspond to electron transfer steps and the numbers are standard one-electron reduction potentials (in V vs. Ag/AgCl). Vertical arrows indicate proton transfer steps labelled with pK_a values. Diagonal arrows represent concerted PCET reactions and the numbers are effective reduction potentials (in V vs. Ag/AgCl). All values were computed using DFT in solvent phase in standard conditions (T=298 K, *p*=1 atm, 1 M concentration and pH=0). Where applicable, pK_a and PCET values were adjusted to reflect *p*H-dependent free energy shifts.

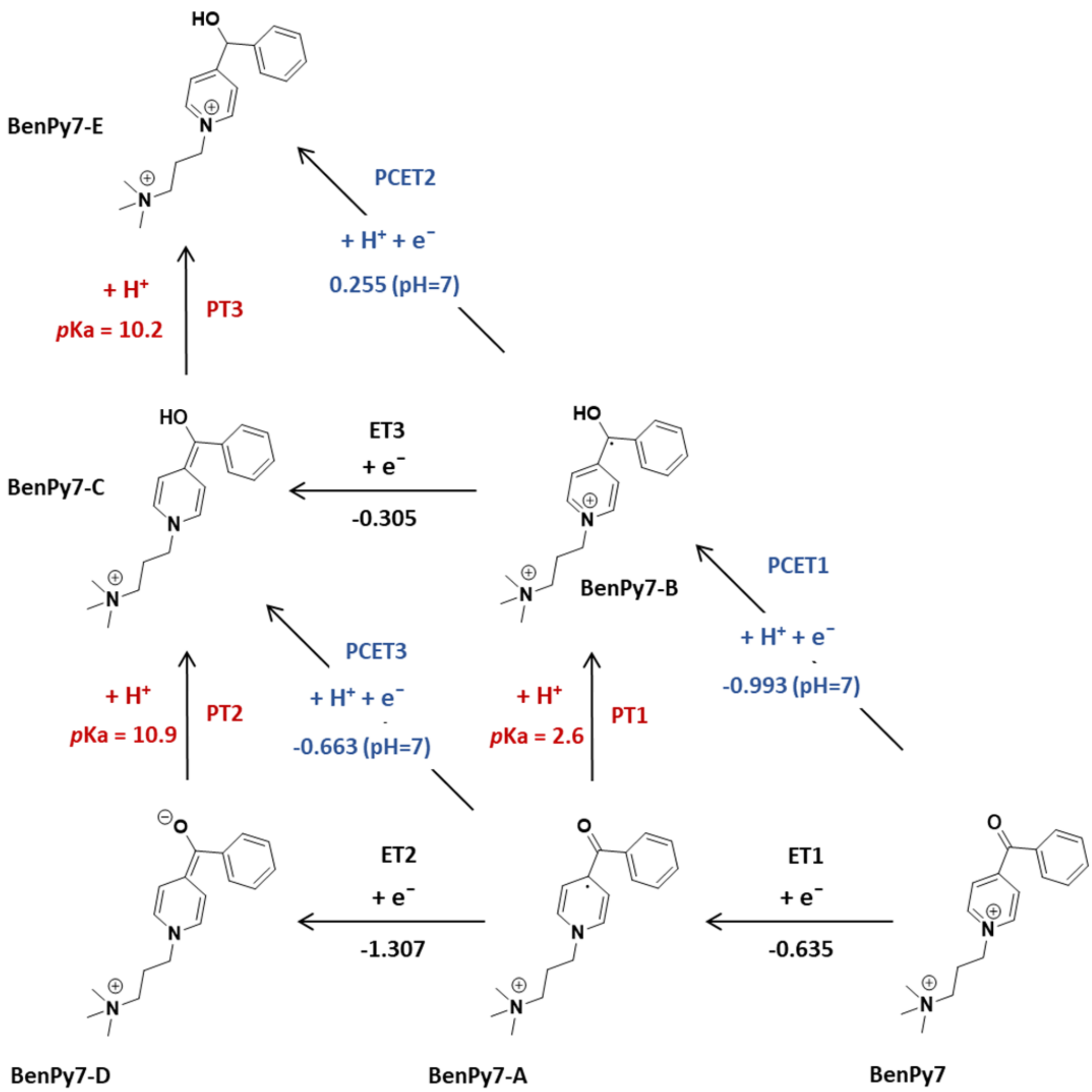

Figure S8.2: Thermodynamic square scheme representing the electron transfer (ET), proton transfer (PT) and proton-coupled electron transfer (PCET) steps for the redox-active compound **BenPy7**. Horizontal arrows correspond to electron transfer steps and the numbers are standard one-electron reduction potentials (in V vs. Ag/AgCl). Vertical arrows indicate proton transfer steps labelled with pK_a values. Diagonal arrows represent concerted PCET reactions and the numbers are effective reduction potentials (in V vs. Ag/AgCl). All values were computed using DFT in solvent phase in standard conditions (T=298 K, *p*=1 atm, 1 M concentration and pH=0). Where applicable, pK_a and PCET values were adjusted to reflect *p*H-dependent free energy shifts.

8.4 Thermodynamic cycles

In order to compare the energetic preferences of different proton and electron transfer pathways leading from **BenPy6** and **AZON3** to ***E*** and ***J*** respectively, we

constructed thermodynamic cycles based on computed Gibbs free energy changes for each elementary step corresponding to the fundamental processes: electron transfer, proton transfer and proton-coupled electron transfer.

Table S1 Computed aqueous-phase relative Gibbs free energies of the electron transfer (ET-), proton transfer (PT-) and proton-coupled electron transfer (PCET-) processes. All ΔG values were obtained from DFT-calculated Gibbs free energies in the aqueous phase, using the SMD solvent model, under standard conditions (T=298 K, *p*=1 atm, 1 M concentration and pH=0) in kcal/mol, the notation is according to Figure S8.1.

	ET1	*ET2*	*ET3*	*PT1*	*PT2*	*PT3*	*PCET1*	*PCET2*	*PCET3*	*PCET*
BenPy6	7.5	23.1	0.8	-0.6	-22.8	-19.7	7.0	-18.9	0.3	-11.9
AZON3	-0.8	16.8	-6.3	2.1	-21.0	-7.9	1.3	-14.2	-4.1	-12.9

8.5 Deprotonation sites for BenPy6 and BenPy7

To assess the acid-base behavior and identify potential deprotonation sites, we computed the pK_a values for each proton-donating position in compounds **BenPy6** and **BenPy7**. Deprotonation was systematically probed at all chemically relevant sites, and the corresponding pK_a values were determined in aqueous solution according to the equation (pka). These results provide insights into the stability and reactivity of the compounds under physiological and electrochemical conditions.

Compound **BenPy6** displays diverse deprotonation profiles across its different acidic sites. Site 1 exhibits an intramolecular cyclization upon deprotonation, with a computed pK_a = 11.1. This transformation suggests a favorable ring-closing reaction that stabilizes the deprotonated species, potentially impacting the compound's conformational behavior under moderately alkaline conditions. The high pKa value calculated for site 2 indicates that it is non-labile in aqueous environments. Site 3 yields a conventional deprotonated form without structural rearrangement.

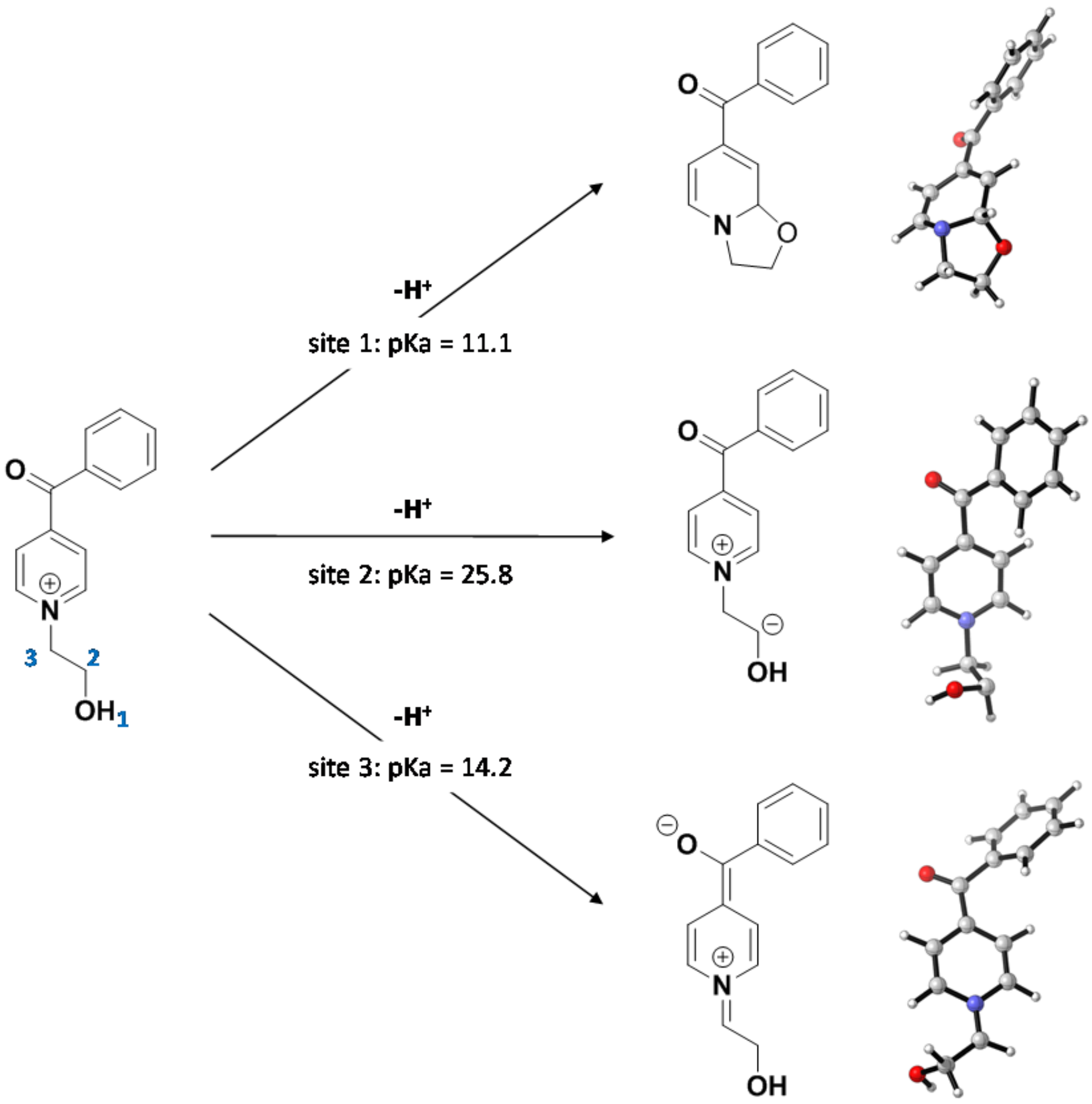

Figure S8.3: Deprotonation map for compound **BenPy6**. The calculated pKa values are indicated on the arrows for each possible deprotonation site.

Compound **BenPy7** also features three deprotonation sites with distinct characteristics. Deprotonation at site 1 is highly unlikely according to the pKa value of 24. Site 2 shows a pK_a of approximately 8, but deprotonation at this position leads to bond cleavage indicating a potential decomposition or degradation pathway under mildly basic conditions. Site 3 has a computed pK_a of 13.7 and undergoes conventional deprotonation without structural rearrangement.

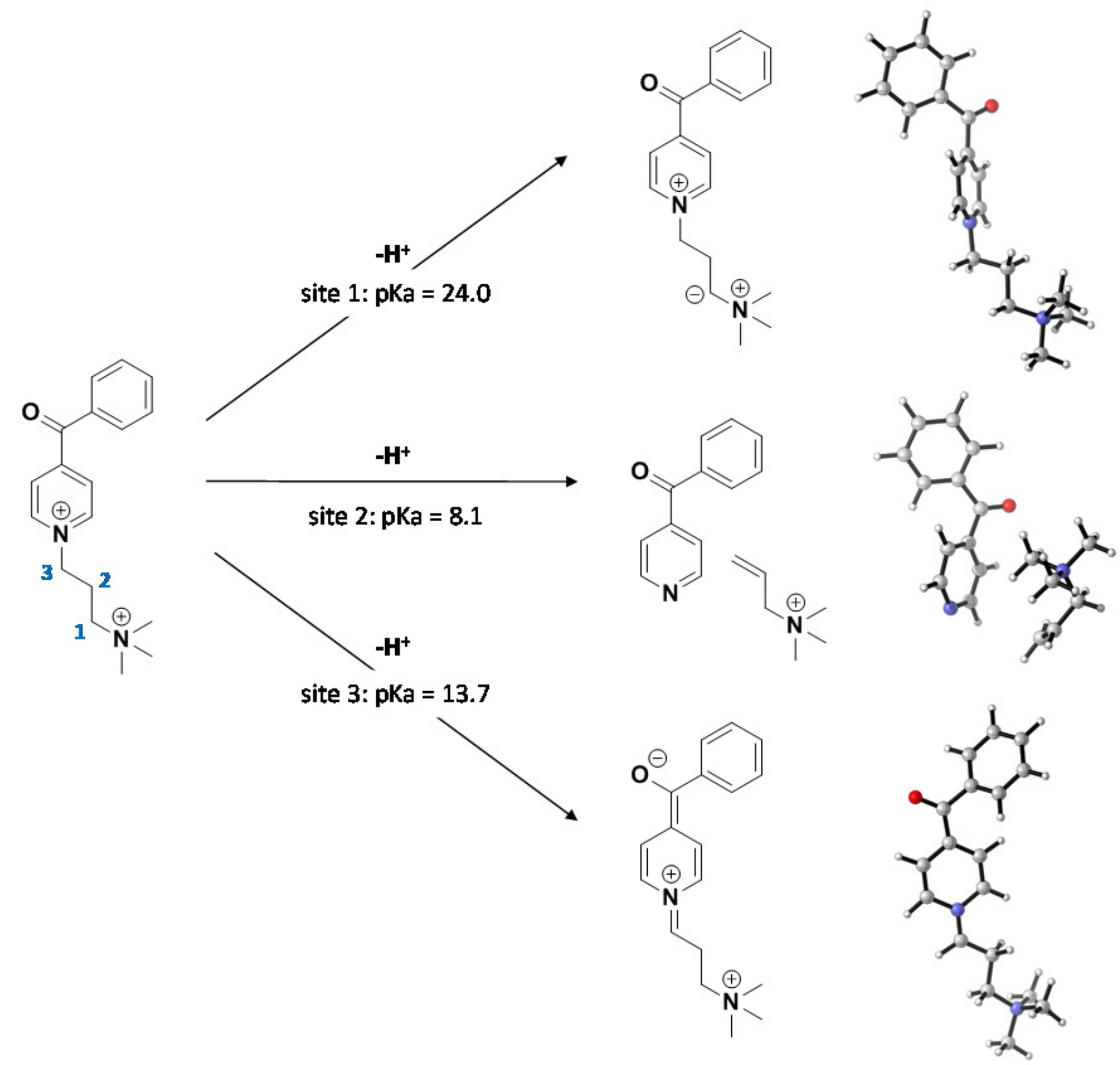

Figure S8.4: Deprotonation map for compound **BenPy7**. The calculated pKa values are indicated on the arrows for each possible deprotonation site.

8.6 Spin density analysis

To probe the electronic structure upon one-electron reduction, the spin density distributions of **BenPy7** and **AZON3** were analyzed (Figure S8.5).

For reduced **BenPy7**, the unpaired spin density is predominantly delocalized over the pyridinium ring and adjacent oxygen atom, indicating that the radical character is mainly localized in this region. In contrast, for **AZON3**, the spin density shifts away from the pyridinium ring toward the five-membered fused ring system.

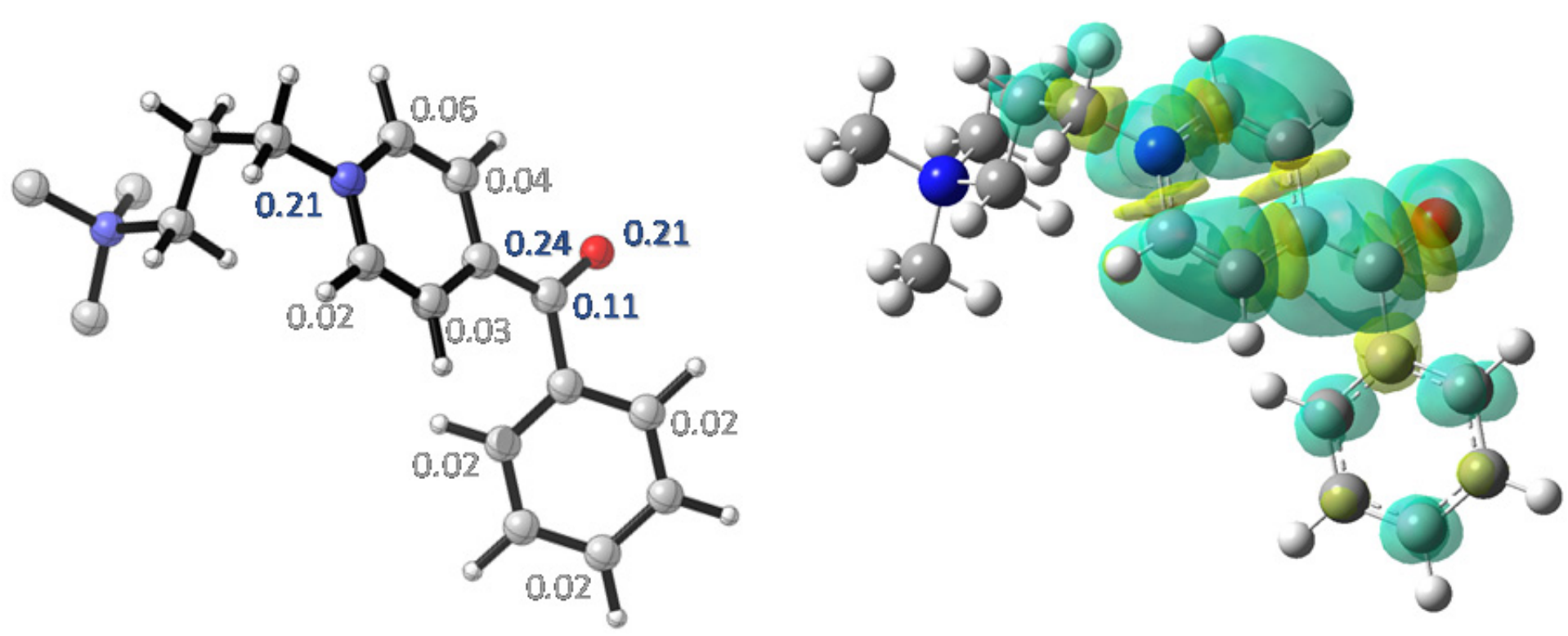

Figure S8.5: Optimized structure of the 1 electron reduced form of **BenPy7** (left), with computed fractional atomic spin densities indicated on the atoms. The right panel shows the corresponding atomic spin density isosurface map plotted at an isovalue of 0.0004 a.u..

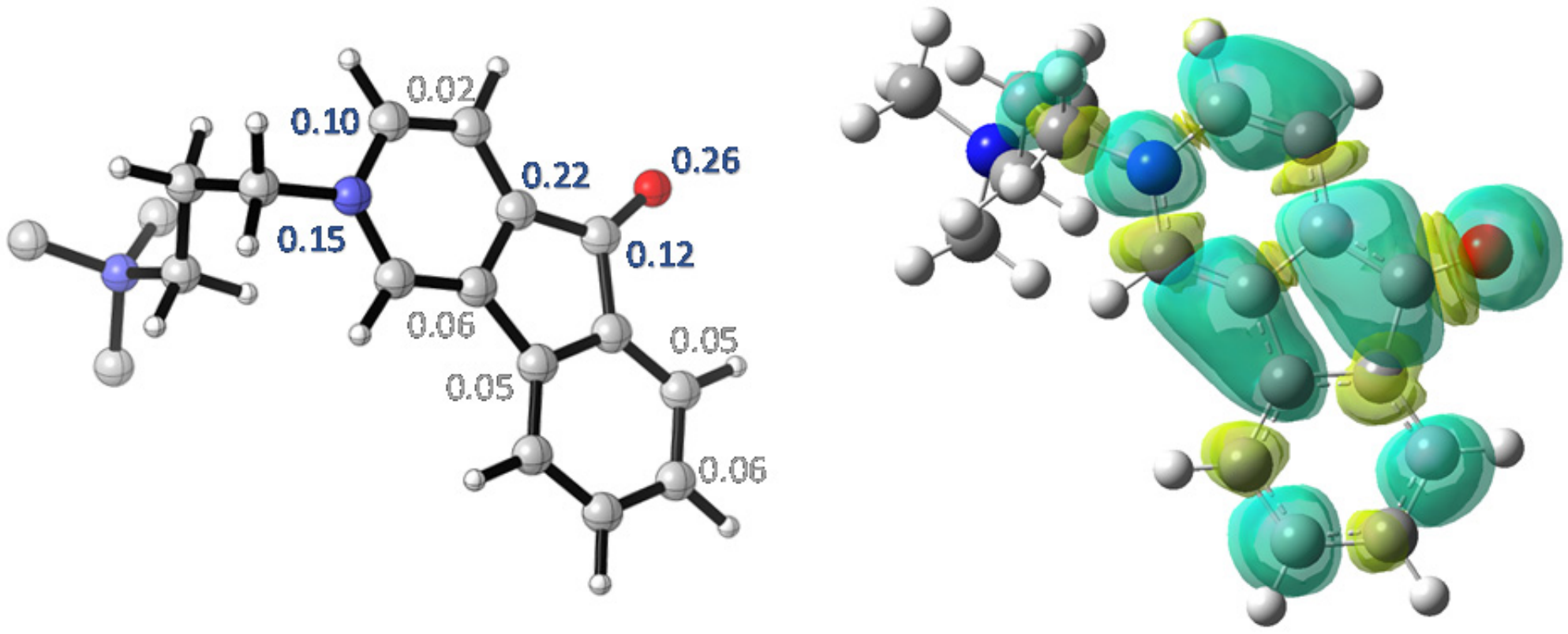

Figure S8.6: Optimized structure of the 1 electron reduced form of **AZON3 (AZON3-A)** (left), with computed fractional atomic spin densities indicated on the atoms. The right panel shows the corresponding atomic spin density isosurface map plotted at an isovalue of 0.0004 a.u..

8.7 Energetic strain analysis upon protonation-induced geometric distortion

To assess the structural effects associated with protonation, an energetic strain analysis was performed for each step of the vertical protonation pathway. The objective was to quantify the energy required to distort a species from its optimized geometry to that imposed by protonation.

The strain energy corresponds to the energy penalty associated with this geometric deformation. A larger strain energy indicates that favorable intramolecular interactions are disrupted or that the molecule must adopt a less favorable conformation to accommodate the proton. All calculations were carried out using electronic energies in solution; thermal corrections were not included, as these are only available for stationary points.

For each protonation step, the geometry of the protonated species (red$_2$-H and red$_2$-H$_2$) was taken, the added proton was removed, and a single-point energy calculation was performed on the resulting structure. These energies were compared to those of the fully optimized geometries of red$_2$ and red$_2$-H, respectively. The resulting energy differences represent the strain energies arising from protonation-induced geometric distortion, independent of the intrinsic thermodynamics of the protonation process.

8.7.1 First protonation

Since the first protonation occurs at the carbonyl oxygen, this step induces only modest geometric changes in both systems, resulting in relatively low strain energies. The local geometry around the protonation site remains largely intact, and the surrounding molecular framework does not undergo significant rearrangement.

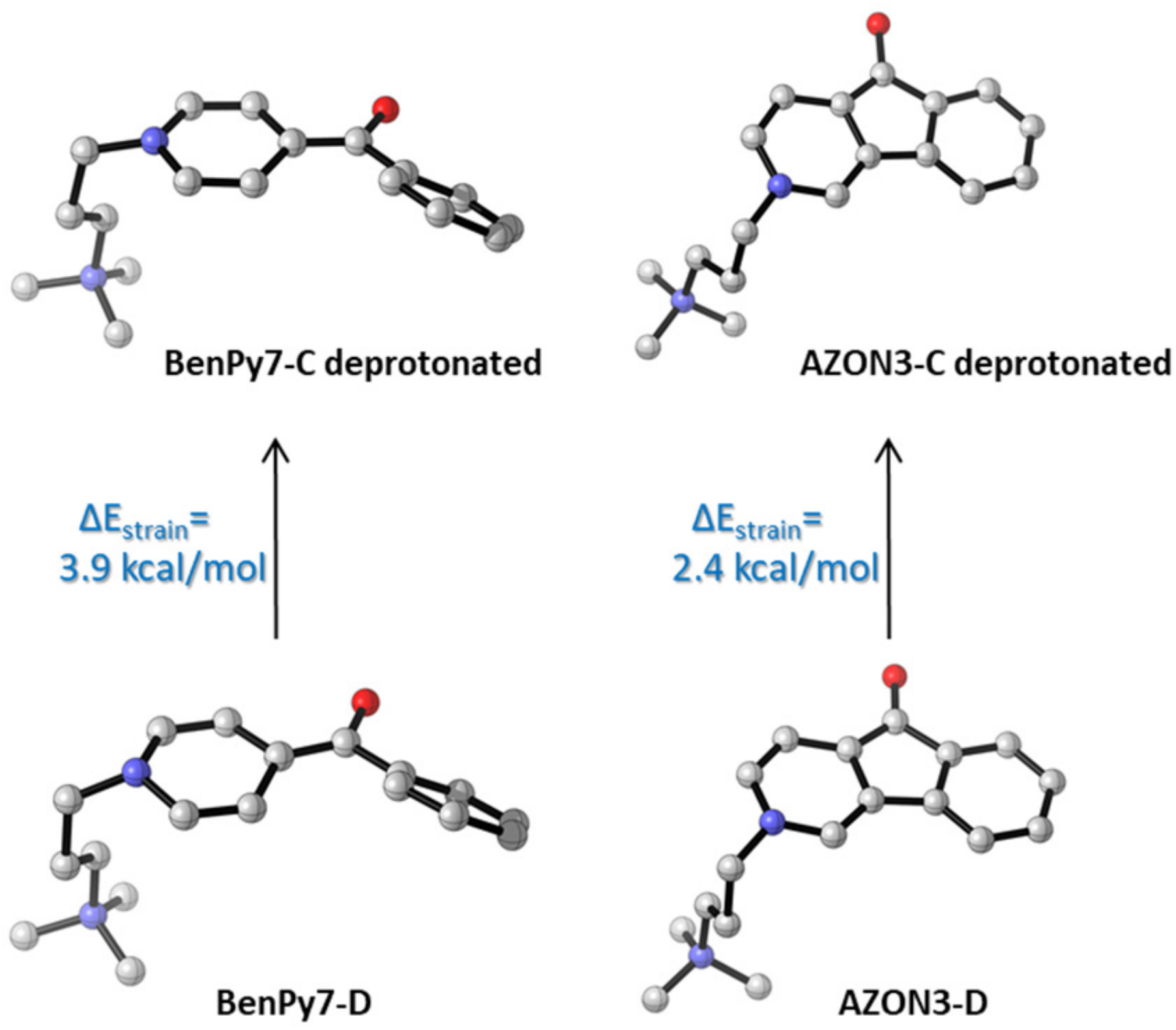

Figure S8.7: Geometries and strain energies for the first protonation.

8.7.2 Second protonation

The second protonation occurs at the carbon atom of the carbonyl group and induces significant geometric strain due to the change in hybridization from sp^2 to sp^3. As a result, the molecule must adopt a non-planar geometry, leading to distinct strain patterns in the two systems.

In **BenPy7**, the flexible molecular framework allows bending of the two aromatic rings, resulting in a large inter-ring dihedral angle (≃70°). This distortion is energetically costly and leads to a total strain energy of 32.3 kcal/mol. In contrast, **AZON3** is conformationally rigid and largely maintains planarity upon protonation. Although the total strain energy is also substantial (28.2 kcal/mol), its origin differs. In AZON3, the rigidity of the backbone prevents ring bending, and instead the OH-group is displaced out of plane.

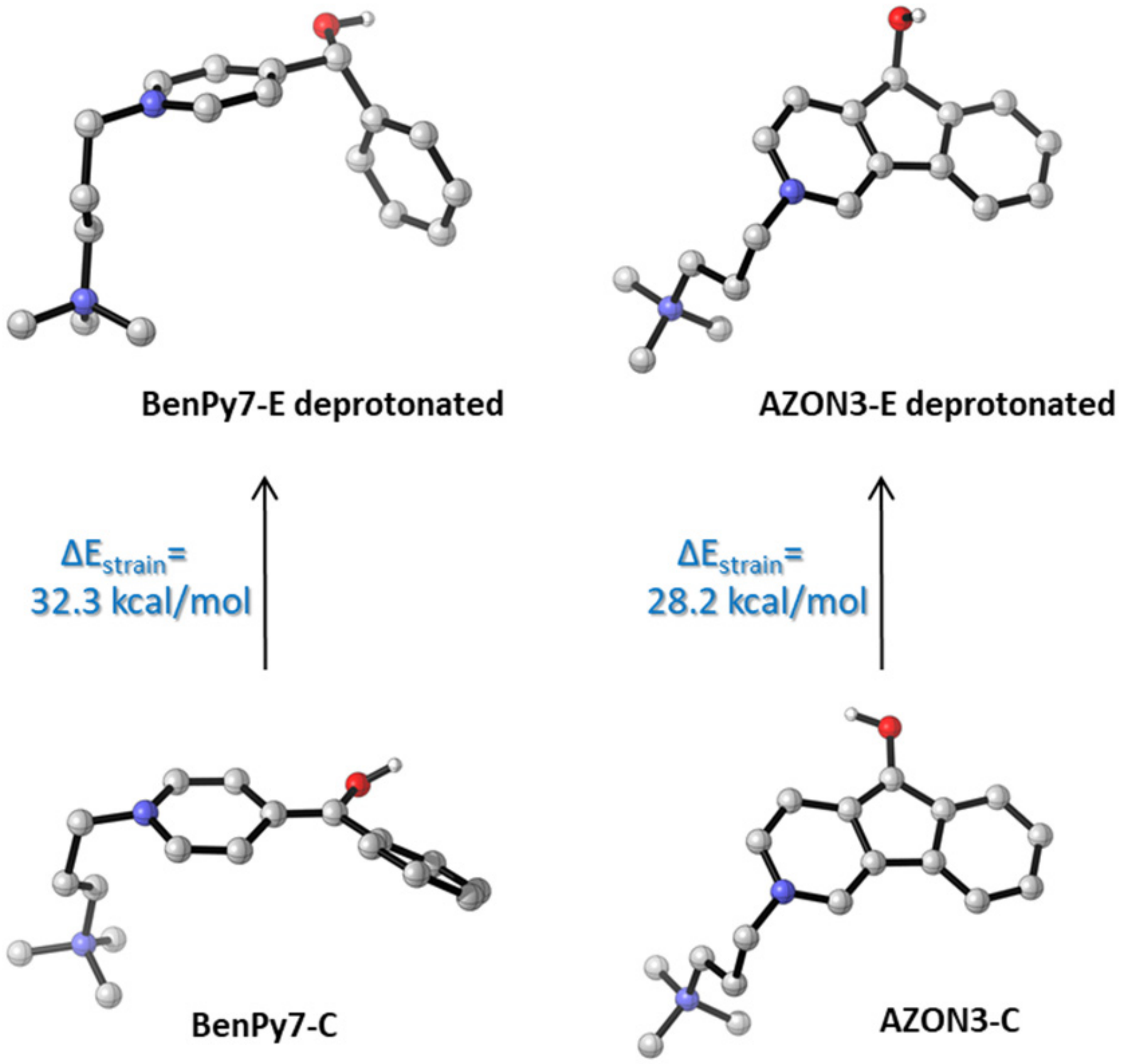

Figure S8.8: Geometries and strain energies for the second protonation.

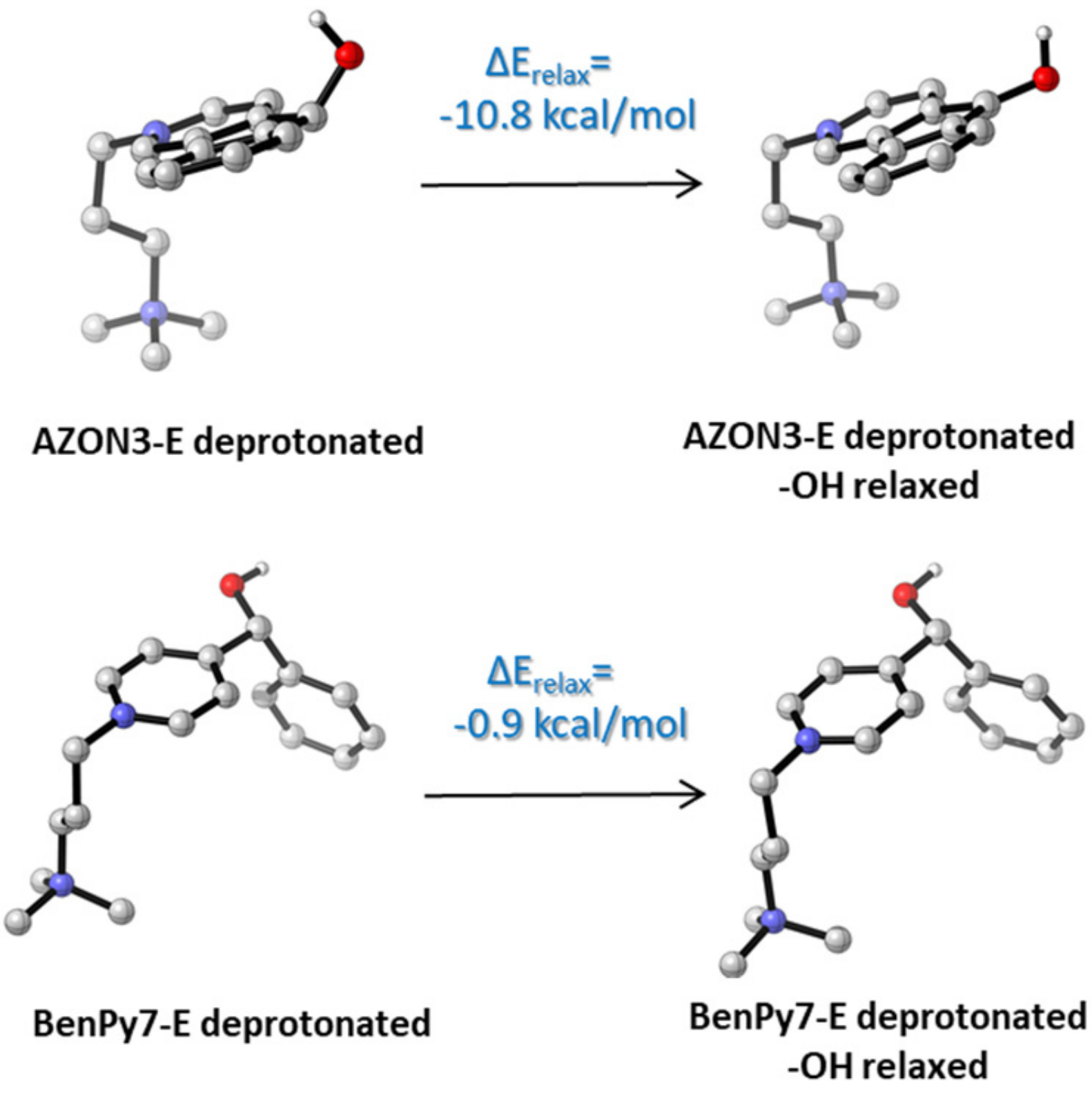

Figure S8.9: Geometries and relaxation energies of the hydroxy-groups.

To disentangle these contributions, partial relaxation of the carbonyl moiety was performed in both systems. For **AZON3**, the majority of the strain arises from the out-of-plane orientation of the OH-group, while the residual backbone strain is considerably smaller (17.4 kcal/mol). In **BenPy7**, however, the OH-group undergoes only minor distortion, and the strain is dominated by substantial ring bending. Even after partial relaxation, the total strain energy remains high (31.8 kcal/mol), reflecting the significant backbone deformation required upon protonation.

8.8 Model calculations for the second protonation

8.8.1 Protonation of model systems **AZON3_Me** and **BenPy7_Me**

To isolate the intrinsic structural effects associated with the second protonation and to disentangle them from conformational flexibility of the full system, truncated model systems (**AZON3_Me** and **BenPy7_Me**) were constructed. The 3-aminopropyl substituent was removed to reduce conformational variability and focus exclusively on the protonation event at the carbonyl carbon site. HCOOH was used as a simple proton donor. Figures S10 and S11 show the calculated reaction pathways for protonation at the carbonyl C atom. Transition states were optimized, and pseudo-IRC calculations were performed to confirm the connectivity between reactant and product minima.

The computed protonation barrier is higher for the more rigid **AZON3_Me** (15.2 kcal/mol) than for **BenPy7_Me** (9.6 kcal/mol). However, the thermodynamic relaxation following protonation differs markedly between the two systems. For **AZON3_Me**, the energy change from the transition state to the protonated minimum is smaller (+0.3 kcal/mol), indicating only limited stabilization of the product. In contrast, **BenPy7_Me** exhibits substantial post-transition-state stabilization (-13.2 kcal/mol) resulting in a significantly seeper product well. This difference reflects the pronounced sp^2 to sp^3 rehybridization at the carbonyl carbon atom and the associated backbone deformation in **BenPy7_Me**, which leads to a strongly stabilized distorted product geometry.

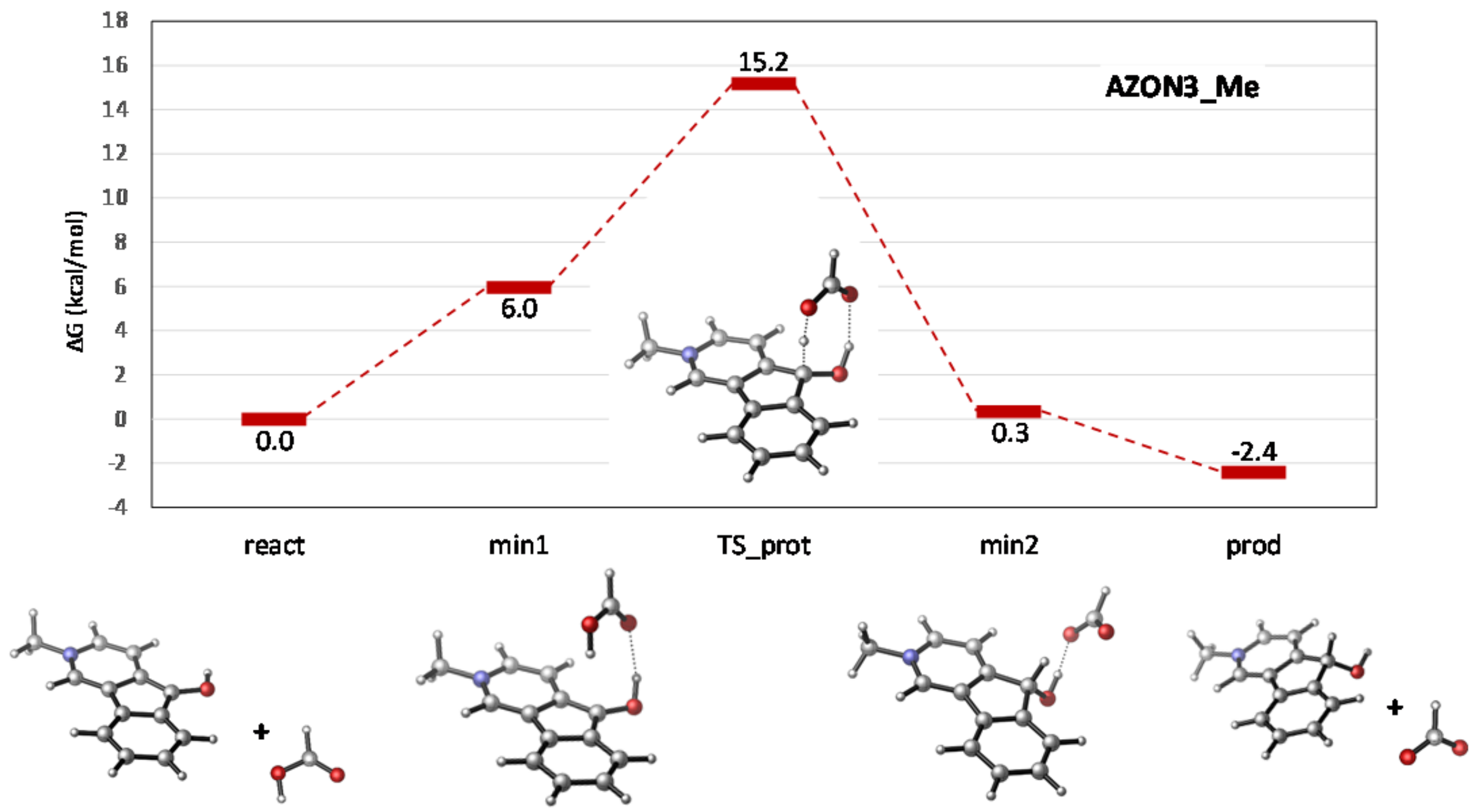

Figure S8.10: Reaction pathway for the second protonation of **AZON3_Me**. Gibbs free energies (ΔG, kcal/mol) are given relative to the separated compound and proton donor. Structures of all stationary points are shown. Notation: react = separated reactant species + proton donor, min1 = proton donor coordinated to the carbonyl carbon atom, TS_prot = transition state for second protonation, min2 = protonated intermediate, prod = final protonated species + deprotonated donor

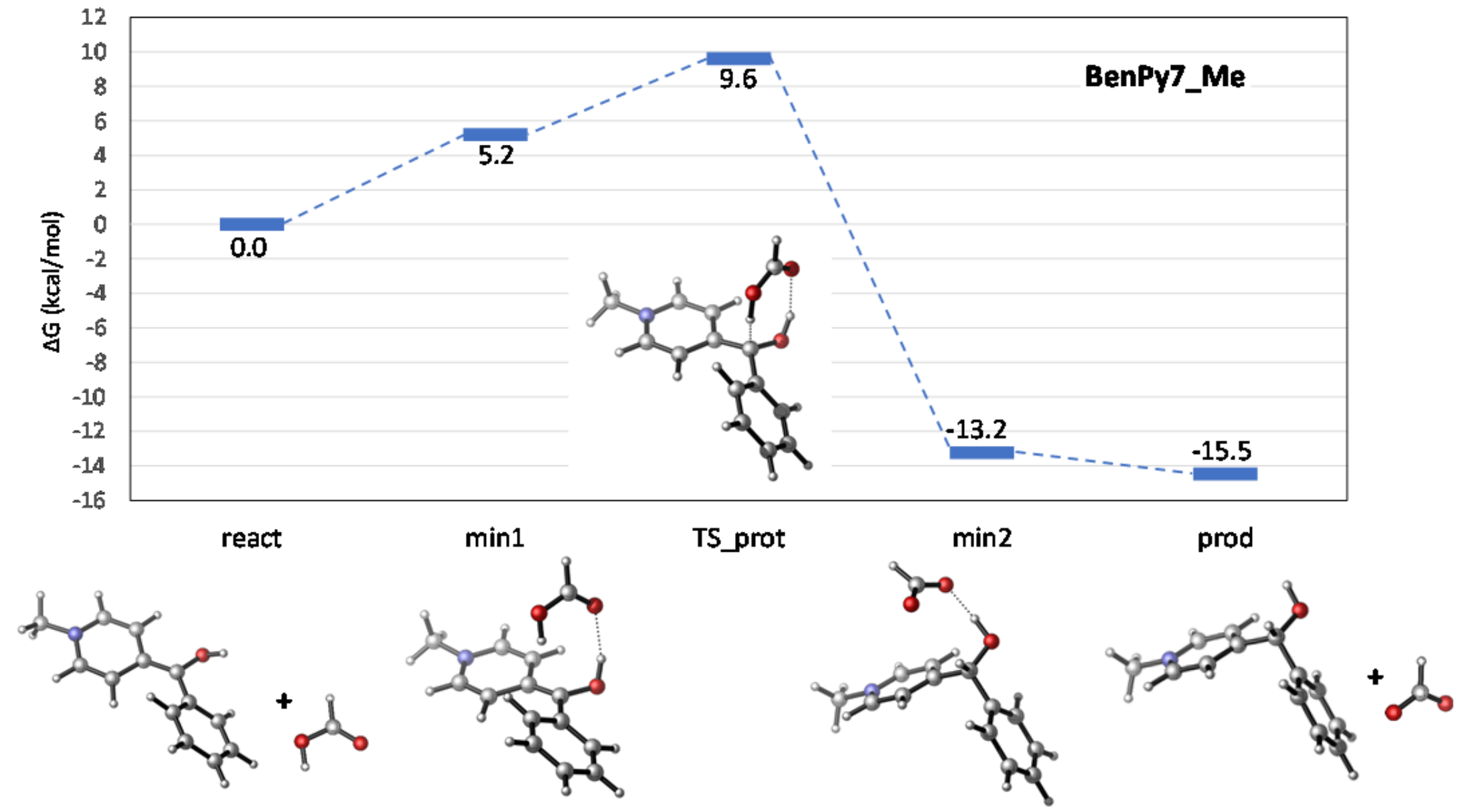

Figure S8.11: Reaction pathway for the second protonation of **BenPy_Me**. Gibbs free energies (ΔG, kcal/mol) are given relative to the separated compound and proton donor. Structures of all stationary points are shown. Notation is as in Figure S8.10.

The reactant state corresponds to the separated reduced species and proton donor, while the product state represents the protonated species and the deprotonated donor.

To rationalize the differing reversibility of the second protonation in the two systems, possible reverse pathways were examined. In addition to the direct microscopic reverse reaction (C-H deprotonation), alternative deprotonation channels were considered. In particular, deprotonation at the carbonyl oxygen was also evaluated. However, the corresponding scan shows a monotonically increasing energy profile without formation of a transition state, indicating that this pathway is not kinetically accessible.

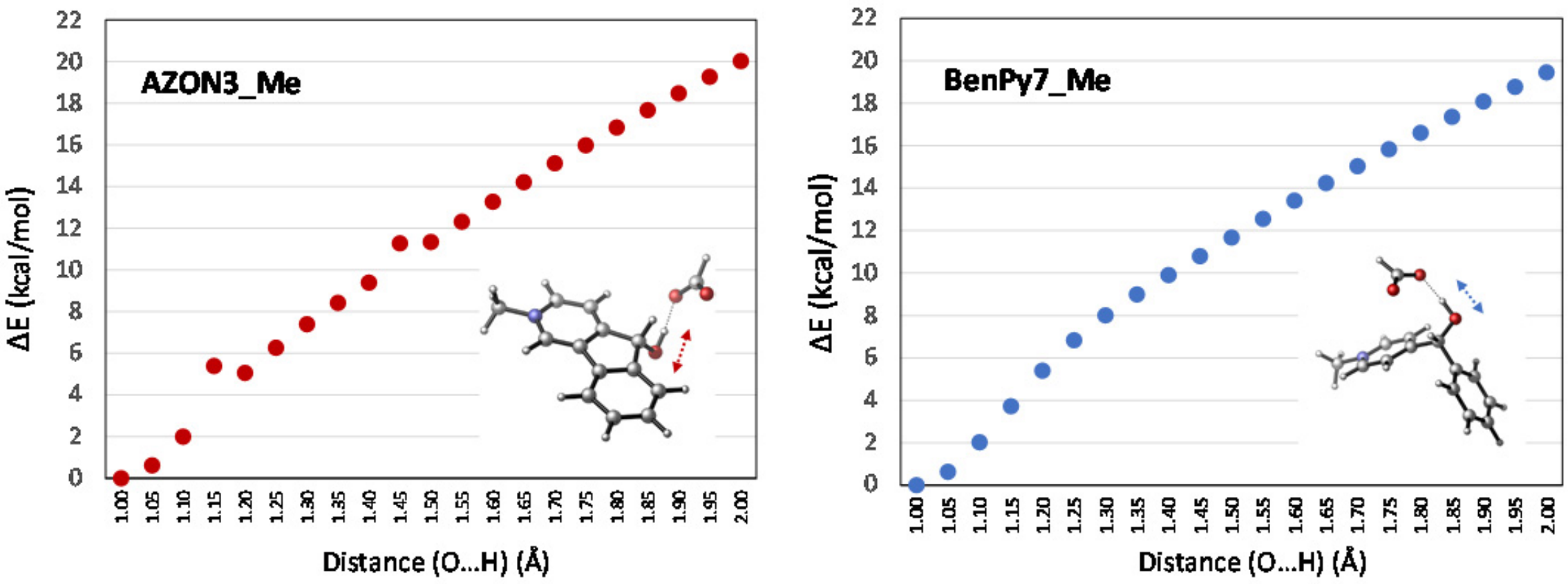

Figure S8.12: Stepwise scan for deprotonation of **AZON3_Me** and **BenPy7_Me**. The reaction coordinate corresponds to the carbonyl-O…H bond distance. Energies correspond to electronic energies (ΔE, kcal/mol) relative to the protonated intermediate.

8.8.2 TS structural evolution

Stepwise transition state scans were performed to monitor the geometric evolution along the protonation pathway. In **BenPy7_Me**, the relevant inter-ring dihedral angle increases from 5° in the nearly planar reactant structure and is already substantially distorted at the transition state, reaching 79° in the protonated product (see Figure S8.13). This progressive deformation demonstrates that substantial backbone distortion occurs along the reaction coordinate and becomes fully expressed in the product minimum. The observed geometric evolution is fully consistent with the strain analysis discussed in Section 8.7.

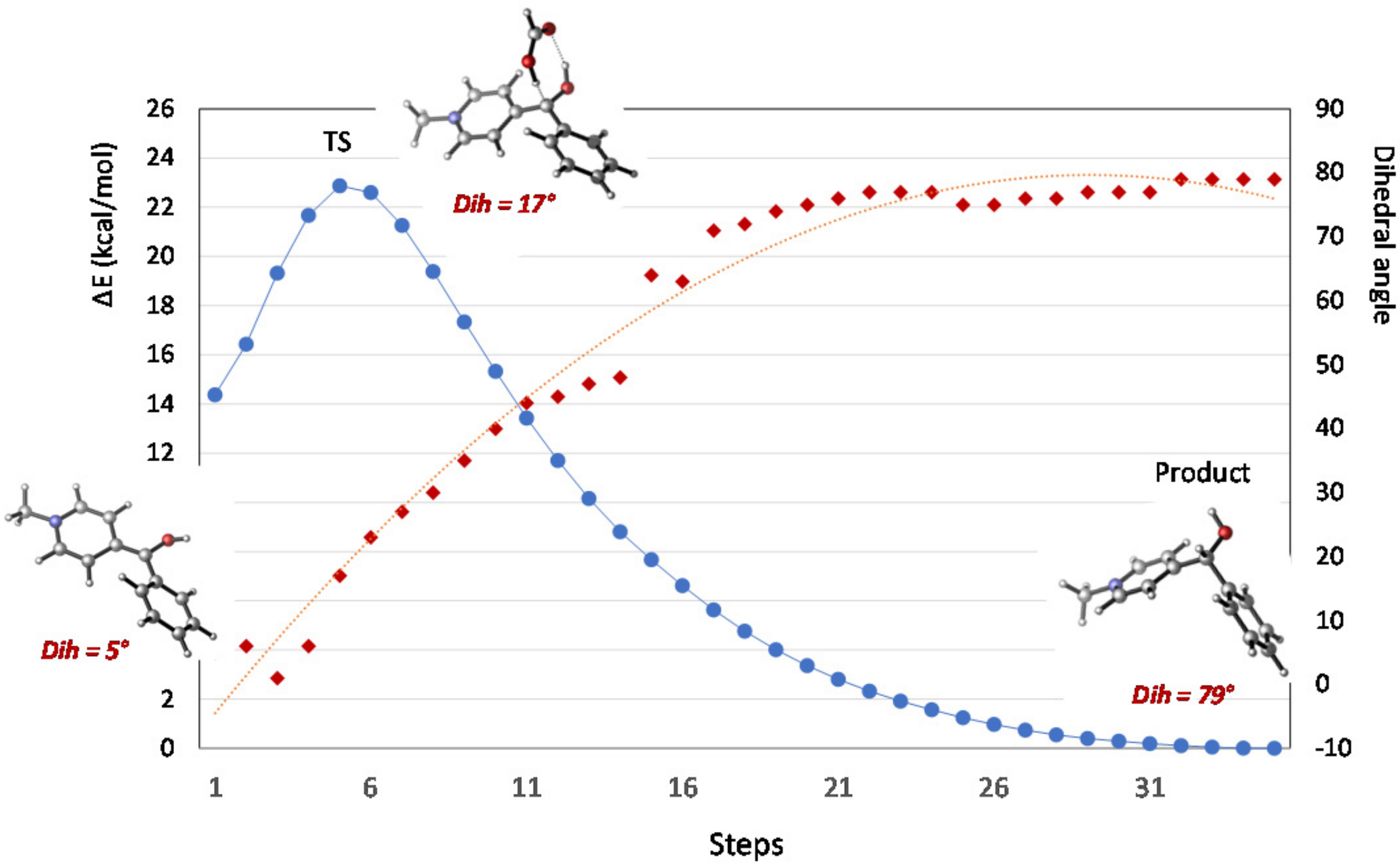

Figure S8.13: Stepwise scan for the second protonation of **BenPy7_Me**. Energies (blue dots, left y-axis) correspond to electronic energies (ΔE, kcal/mol) relative to the protonated intermediate. The evolution of the relevant dihedral angle (red diamonds, right y-axis) is shown along the scan (x-axis = step number). Representative structures are shown for the nearly planar deprotonated species, the transition state, and the doubly protonated sp^3 product.

8.8.3 Proton donor dependence

Transition state structures and derived minima along the IRC pathways were computed for both systems using four proton donors (HCOOH, CH_3COOH, H_2CO_3, CF_3COOH). The TS geometries are qualitatively similar, indicating that the protonation pathway is largely independent of the proton donor.

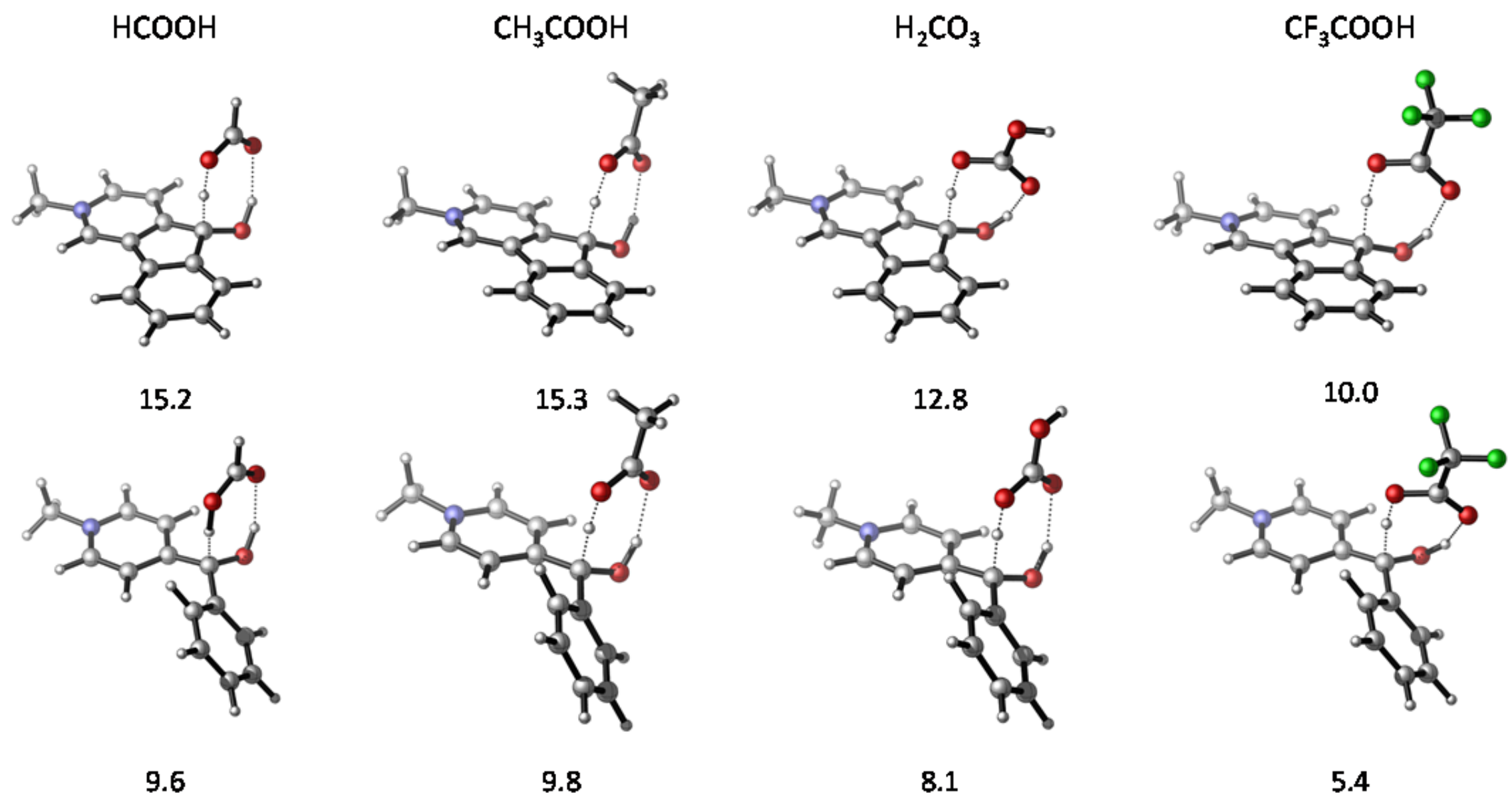

Figure S8.14: Transition state structures for the second protonation of **AZON3_Me** (top), and **BenPy7_Me** (bottom) with four proton donors (HCOOH, CH_3COOH, H_2CO_3 and CF_3COOH). Numbers indicate Gibbs free energies (ΔG, kcal/mol) of the transition states relative to the separated compound and proton donor.

The calculated free energies along the full protonation pathway for both systems and all proton donors are summarized in Table S2. The corresponding reverse barriers are shown in Figure S8.15, highlighting that the barrier is around 10 kcal/mol higher for the irreversible system **BenPy7_Me**, independent of the proton donor. While the absolute barrier heights vary with the proton donor strenght, the relative difference between the two systems remains consistent. This demonstrates that the observed irreversibility originates from intrinsic structural and energetic properties of the molecular backbone rather than proton donor-dependent.

Table S2 Computed aqueous-phase Gibbs free energies (ΔG, kcal/mol) along the second protonation pathway for **AZON3_Me** and **BenPy7_Me** with four proton donors (HCOOH, CH_3COOH, H_2CO_3, CF_3COOH). Energies are given relative to the

separated compound and proton donor. Stationary point notation is defined in Figure S8.10.

Proton donor	HCOOH		CH_3COOH		H_2CO_3		CF_3COOH	
system	AZON3_Me	BenPy7_Me	AZON3_Me	BenPy7_Me	AZON3_Me	BenPy7_Me	AZON3_Me	BenPy7_Me
react	0.0	0.0	0.0	0.0	0.0	0.0	0.0	0.0
min1	6.0	5.2	5.2	4.3	4.9	4.6	5.8	4.2
TS_prot	15.2	9.6	15.3	9.8	12.8	8.1	10.0	5.4
min2	0.3	-13.2	4.7	-10.8	-2.8	-16.0	-8.3	-22.6
prod	-2.4	-15.5	0.0	-13.1	-5.5	-18.6	-12.5	-25.6

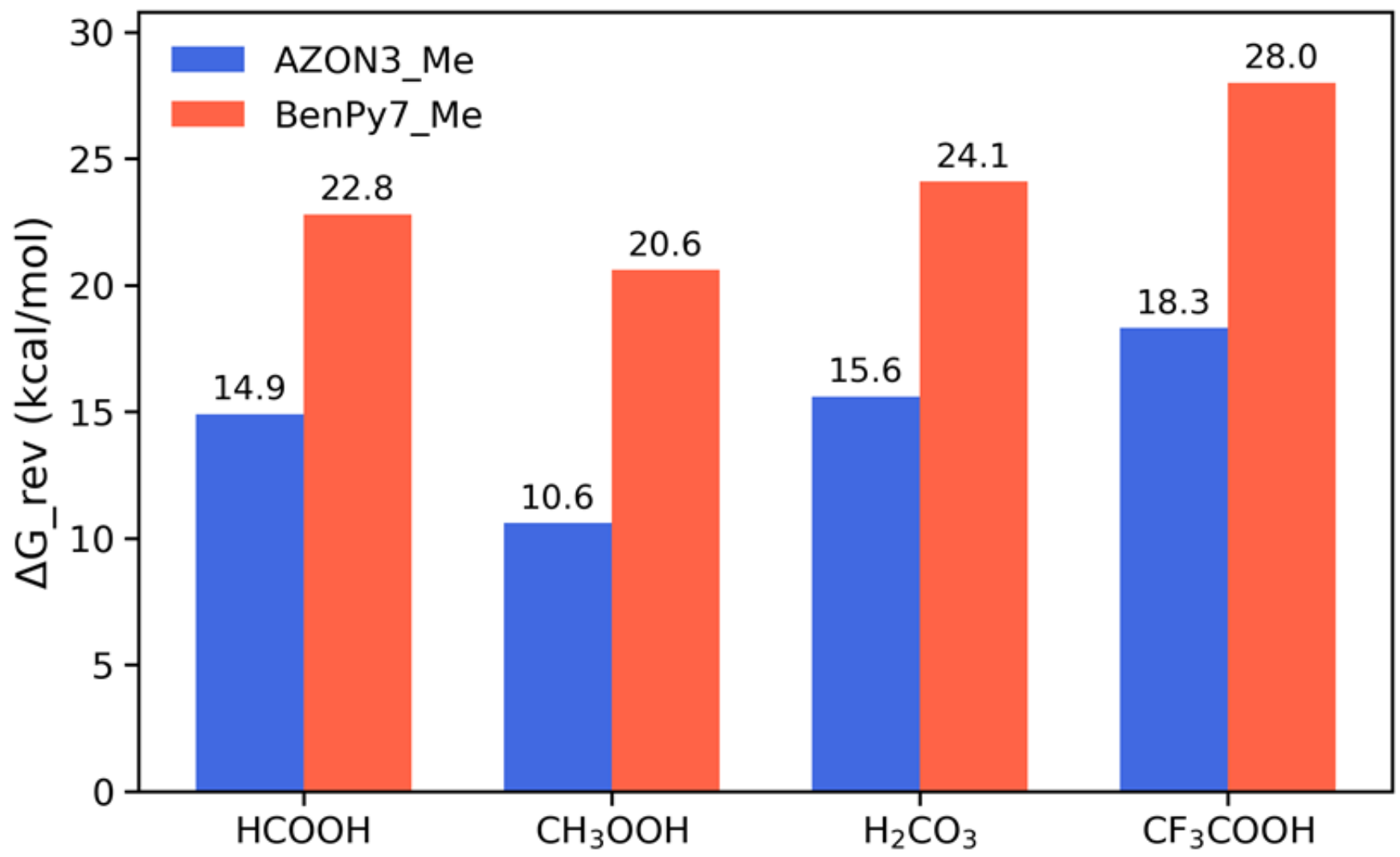

Figure S8.15: Reverse protonation barriers (ΔG_rev, kcal/mol) from the protonated intermediate (min2) to the transition state (TS_prot) for **AZON3_Me** (blue) and **BenPy7_Me** (red) with four proton donors (HCOOH, CH_3COOH, H_2CO_3, CF_3COOH). Bar heights correspond to Gibbs free energy differences relative to the protonated intermediate.

Table S3 Computed aqueous-phase Gibbs free energies (ΔG, kcal/mol) along the second protonation pathway for **AZON3** and **BenPy7** with proton donors: HCOOH and H_2CO_3. Energies are given relative to the separated compound and proton donor. Stationary point notation is defined in Figure S8.10.

Proton donor	HCOOH	H_2CO_3

system	**AZON3**	**BenPy7**	**AZON3**	**BenPy7**
react	0.0	0.0	0.0	0.0
min1	5.6	4.3	5.8	4.0
TS_prot	15.4	10.2	13.7	7.3
min2	1.5	-11.4	-1.4	-14.1
prod	-2.3	-14.3	-5.4	-17.4

Table S4: Calculated activation free-energy barriers in the truncated model systems (AZON3_Me and BenPy7_Me) using different proton donors. ΔG^{prot} corresponds to the free-energy of the proton transfer transition state relative to the separated reactants (compound + proton donor), while ΔG^{rev} represents the reverse barrier (energies are given in kcal/mol).

Proton donor	ΔG^{prot} AZON3_Me	ΔG^{prot} BenPy7_Me	ΔG^{rev} AZON3_Me	ΔG^{rev} BenPy7_Me
HCOOH	15.2	9.6	14.9	22.8
CH_3COOH	15.3	9.8	10.6	20.6
H_2CO_3	12.8	8.1	15.6	24.1
CF_3COOH	10.0	5.4	18.3	28.0

8.9 Energy data for the calculated structures

Table S5: Computed energy data for M06-2X/6-311G**(DMS, solvent=water) optimized structures (in a.u.)*

compound	E^o	G^o	E_{lb}	G
**H2**	-1.168401	-1.169698	-1.168402	-1.169699
**HCOOH**	-189.751675	-189.741482	-189.759225	-189.749032
**HCOO-**	-189.285213	-189.288089	-189.305856	-189.308731
**CH_3COOH**	-229.067996	-229.033150	-229.075704	-229.040858
**CH_3COO-**	-228.597420	-228.576757	-228.617228	-228.596566
**H_2CO_3**	-265.003761	-264.989826	-265.013166	-264.999231
**HCO_3-**	-264.543843	-264.542712	-264.564800	-264.563670
**CF_3COOH**	-526.782138	-526.774068	-526.797287	-526.789216
**CF_3COO-**	-526.334386	-526.338483	-526.360717	-526.364814
**BenPy6**	-746.851131	-746.636305	-746.861985	-746.647159
**BenPy6-A**	-746.984071	-746.773282	-746.998712	-746.787923
**BenPy6-B**	-747.435523	-747.210338	-747.446107	-747.220922
**BenPy6-C**	-747.582251	-747.359171	-747.595418	-747.372338
**BenPy6-D**	-747.090607	-746.882295	-747.112229	-746.903917
**BenPy6-E**	-748.061007	-747.824143	-748.072693	-747.835830
Deprotonation				

**BenPy6_site1**	-746.294337	-746.096366	-746.312781	-746.114811
**BenPy6_site2**	-746.352654	-746.152496	-746.366246	-746.166088
**BenPy6_site3**	-746.370788	-746.167178	-746.383809	-746.180199
**AZON3**	-883.436348	-883.107052	-883.444094	-883.114798
**AZON3-A**	-883.584170	-883.257507	-883.595483	-883.268821
**AZON3-B**	-884.029494	-883.690022	-884.036979	-883.697507
**AZON3-C**	-884.188680	-883.850283	-884.198651	-883.860254
**AZON3-D**	-883.701892	-883.376215	-883.720400	-883.394723
**AZON3-E**	-884.648199	-884.296762	-884.656984	-884.305547
Deprotonation				
**AZON3_site1**	-884.089620	-883.757647	-884.101097	-883.769124
**AZON3_site2**	-884.159217	-883.829703	-884.168847	-883.839333
**AZON3_site3**	-884.139854	-883.804827	-884.149867	-883.814840
**BenPy7**	-884.633408	-884.285710	-884.641099	-884.293401
**BenPy7-A**	-884.769618	-884.424370	-884.780346	-884.435098
**BenPy7-B**	-885.216428	-884.857370	-884.853165	-885.223746
**BenPy7-C**	-885.368229	-885.009357	-885.377386	-885.018515
**BenPy7-D**	-884.878711	-884.534835	-884.895983	-884.552107
**BenPy7-E**	-885.844755	-885.473560	-885.853035	-885.481841
Model systems				
**AZON3_Me**	-631.875670	-631.705043	-631.884903	-631.714277
**BenPy7_Me**	-633.053901	-632.862074	-633.062403	-632.870576
HCOOH donor				
**AZON3_Me_TS_prot**	-821.620615	-821.422322	-821.637605	-821.439312
**AZON3_Me_min2**	-821.644317	-821.443405	-821.663896	-821.462984
**BenPy7_Me_TS_prot**	-822.806381	-822.589086	-822.821806	-822.604511
**BenPy7_Me_min2**	-822.842914	-822.622356	-822.861469	-822.640911
CH_3COOH donor				
**AZON3_Me_TS_prot**	-860.934882	-860.713705	-860.951918	-860.730741
**AZON3_Me_min2**	-860.956738	-860.728487	-860.975962	-860.747711
**BenPy7_Me_TS_prot**	-862.120925	-861.880350	-862.136390	-861.895814
**BenPy7_Me_min2**	-862.156701	-861.910138	-862.175181	-861.928618
H_2CO_3 donor				
**AZON3_Me_TS_prot**	-896.875187	-896.674006	-896.894280	-896.693099
**AZON3_Me_min2**	-896.900409	-896.696131	-896.922278	-896.718000
**BenPy7_Me_TS_prot**	-898.059975	-897.839273	-898.077551	-897.856849
**BenPy7_Me_min2**	-898.101569	-897.875062	-898.121875	-897.895368
CF_3COOH donor				
**AZON3_Me_TS_prot**	-1158.659070	-1158.462444	-1158.684127	-1158.487501
**AZON3_Me_min2**	-1158.689615	-1158.489172	-1158.717232	-1158.516788
**BenPy7_Me_TS_prot**	-1159.844146	-1159.627797	-1159.867566	-1159.651217
**BenPy7_Me_min2**	-1159.892772	-1159.670354	-1159.918241	-1159.695823
Real systems				
HCOOH donor				
**AZON3_TS_prot**	-1073.933342	-1073.568216	-1073.951243	-1073.586118
**AZON3_min2**	-1073.955614	-1073.587675	-1073.976232	-1073.608293

**BenPy7_TS_prot**	-1075.118786	-1074.735311	-1075.135035	-1074.751560
**BenPy7_min2**	-1075.154353	-1074.766365	-1075.173912	-1074.785924
H_2CO_3 donor				
**AZON3_TS_prot**	-1149.187337	-1148.818703	-1149.207447	-1148.838813
**AZON3_min2**	-1149.212066	-1148.839961	-1149.234979	-1148.862874
**BenPy7_TS_prot**	-1150.373570	-1149.987859	-1150.391846	-1150.006135
**BenPy7_min2**	-1150.411951	-1150.018390	-1150.433781	-1150.040221

*Notations: E^o and E_{lb} are electronic energies computed at M06-2X/6-311G**(DMS, solvent=water) and M06-2X/6-311++G**(DMS, solvent=water) level of DFT, G^o are solution-phase Gibbs free energies, and the last column is obtained as: $G = E_{lb} + (G^o - E^o)$.

8.10 Cartesian coordinates of the calculated structures

Cartesian coordinates of structures optimized at M06-2X/6-311G**(DMS, solvent=water) level of theory are given in standard XYZ format (units are in Å). The first line indicates the number of atoms, the second line is the molecule name.

2
H2
H 0.000000 0.000000 0.133683
H 0.000000 0.000000 0.874317
5
HCOOH
C 0.048601 0.000000 0.000619
O -0.033948 0.000000 1.329450
O 1.079717 0.000000 -0.619016
H 0.863748 0.000000 1.700855
H -0.948998 0.000000 -0.449741
4
HCOO-
C 0.030320 0.000000 0.062408
O -0.047091 0.000000 1.311777
O 1.068262 0.000000 -0.637005
H -0.938043 0.000000 -0.491680
8
CH_3COOH
C -0.048268 0.138701 0.060748
O 0.053224 0.131962 1.265234
O 1.015991 0.292380 -0.743775
C -1.320230 -0.016317 -0.707907
H 1.806924 0.387238 -0.190494
H -2.153325 -0.136758 -0.020943
H -1.469574 0.863879 -1.335182
H -1.239529 -0.885412 -1.362678
7
CH_3COO-
C 0.044173 0.000000 0.068995

O -0.008745 0.000000 1.324455
O 1.098448 0.000000 -0.618089
C -1.275344 0.000000 -0.702000
H -2.133750 0.000000 -0.031551
H -1.317140 0.879631 -1.348154
H -1.317140 -0.879631 -1.348154
6

H_2CO_3

C -0.077885 0.042871 0.066976
O -0.016189 0.086974 1.270098
O 0.923924 0.302472 -0.766130
O -1.158886 -0.273097 -0.637876
H 1.704355 0.531004 -0.240510
H -1.879988 -0.459483 -0.019042
5

HCO_3-

C -0.023880 0.057864 0.012224
O -0.074374 0.070340 1.256837
O 0.929869 0.304527 -0.743179
O -1.204887 -0.286148 -0.650187
H -1.864529 -0.454506 0.032802
8

CF_3COOH

C -0.007155 0.143363 0.091151
O 0.013589 0.126266 1.285939
O 1.008693 0.291327 -0.733348
C -1.310248 -0.015028 -0.711087
H 1.833410 0.391660 -0.227903
F -2.341864 -0.163366 0.107333
F -1.525122 1.056202 -1.475543
F -1.244428 -1.081310 -1.509109
7

CF_3COO-

C 0.057144 0.152762 0.043201
O 0.002855 0.125533 1.280568
O 1.024083 0.292877 -0.724323
C -1.294901 -0.013169 -0.701960
F -2.342069 -0.163933 0.116440
F -1.554070 1.052119 -1.480970
F -1.273090 -1.084313 -1.515030
31

BenPy6

O -4.242604 1.286902 -1.334743
C -4.482932 1.343138 0.058748
C -4.161587 0.021918 0.740655
N -2.742662 -0.326342 0.506669
C -1.800862 0.140287 1.346445

C -0.464026 -0.078960 1.092047
C -0.102405 -0.792746 -0.047661
C 1.332876 -1.152687 -0.346784
C 2.408273 -0.159193 -0.105928
C 3.715967 -0.631629 0.047841
C 4.757560 0.263183 0.237390
C 4.502117 1.633903 0.253116
C 3.205591 2.108743 0.082000
C 2.154608 1.215394 -0.090447
O 1.562477 -2.264532 -0.775794
C -1.093891 -1.272015 -0.897484
C -2.414618 -1.010095 -0.604984
H -4.881679 0.678125 -1.723699
H -3.841972 2.130307 0.459215
H -5.524138 1.590124 0.282792
H -4.320268 0.089565 1.815500
H -4.765444 -0.787230 0.332070
H -2.154134 0.682512 2.213293
H 0.271954 0.294186 1.791557
H 3.899267 -1.699123 0.026526
H 5.768165 -0.102913 0.370224
H 5.317927 2.332567 0.396475
H 3.011399 3.174111 0.082204
H 1.149670 1.593426 -0.237101
H -0.844145 -1.840941 -1.782602
H -3.234936 -1.343008 -1.224348
31
BenPy6-A
O -4.090234 1.935319 0.777994
C -4.728056 0.693385 0.519426
C -4.187891 0.032031 -0.739532
N -2.771205 -0.308050 -0.596911
C -1.802508 0.606024 -0.923257
C -0.487168 0.376556 -0.662226
C -0.066501 -0.828694 -0.020186
C 1.296196 -1.186946 0.253900
C 2.397467 -0.183568 0.100130
C 3.617691 -0.614185 -0.430937
C 4.687534 0.264151 -0.547057
C 4.560324 1.580486 -0.108590
C 3.359069 2.010495 0.447583
C 2.282126 1.135517 0.549596
O 1.605104 -2.351573 0.609017
C -1.107844 -1.760204 0.292809
C -2.406660 -1.477299 0.021676
H -3.234490 1.757539 1.182711
H -4.621529 0.014022 1.371113

H -5.788975 0.900102 0.371217
H -4.740863 -0.884237 -0.948893
H -4.285927 0.707044 -1.589948
H -2.151389 1.501105 -1.420063
H 0.226449 1.121064 -0.986352
H 3.715941 -1.643451 -0.756052
H 5.622592 -0.077924 -0.974912
H 5.396107 2.265062 -0.192532
H 3.261250 3.027111 0.809835
H 1.357600 1.473682 1.003527
H -0.864248 -2.702622 0.763334
H -3.218504 -2.151656 0.261922
32

BenPy6-B

O -4.218940 1.123317 1.642970
C -4.835802 0.305473 0.664323
C -4.169652 0.448150 -0.695415
N -2.749914 0.068486 -0.620054
C -1.822240 0.993175 -0.266829
C -0.509303 0.656399 -0.099633
C -0.089366 -0.687457 -0.284940
C 1.250608 -1.105523 -0.116071
C 2.409325 -0.224861 0.024007
C 2.535308 0.937776 -0.750141
C 3.659305 1.741283 -0.621265
C 4.668460 1.398396 0.275463
C 4.556747 0.238702 1.037942
C 3.438902 -0.573962 0.911400
O 1.437075 -2.430707 -0.050118
C -1.099431 -1.636014 -0.607533
C -2.392411 -1.233210 -0.765893
H -4.375367 2.043806 1.401717
H -4.744698 -0.724603 1.013250
H -5.897249 0.543463 0.550133
H -4.654245 -0.196048 -1.428120
H -4.216899 1.480657 -1.041833
H -2.185507 2.002068 -0.130582
H 0.181529 1.429950 0.203176
H 1.771012 1.191417 -1.474891
H 3.753239 2.631789 -1.230715
H 5.542788 2.030287 0.374931
H 5.339187 -0.031179 1.736583
H 3.349703 -1.467803 1.519405
H -0.855909 -2.678839 -0.748732
H -3.190140 -1.917403 -1.023207
H 2.377983 -2.650313 -0.109349
32

BenPy6-C

O -4.527475 0.538915 -1.519267
C -4.625655 -0.767164 -0.967160
C -4.175062 -0.799264 0.485885
N -2.781556 -0.386409 0.631160
C -1.776441 -1.253032 0.251624
C -0.490722 -0.871496 0.119288
C -0.081578 0.512063 0.342280
C 1.194065 0.974574 0.245074
C 2.417166 0.189397 0.041724
C 2.609463 -1.071116 0.632362
C 3.789410 -1.776995 0.436472
C 4.817002 -1.238653 -0.335363
C 4.653763 0.023142 -0.900342
C 3.473061 0.731028 -0.711361
O 1.364678 2.362624 0.240953
C -1.201818 1.411672 0.607645
C -2.462413 0.950401 0.738359
H -3.596415 0.721100 -1.688825
H -5.675624 -1.059632 -1.019476
H -4.038622 -1.480055 -1.555812
H -4.788405 -0.122771 1.082450
H -4.284226 -1.809371 0.884724
H -2.091514 -2.273671 0.071670
H 0.225738 -1.618066 -0.192558
H 1.842636 -1.486474 1.274884
H 3.914737 -2.746008 0.905908
H 5.737671 -1.790375 -0.482771
H 5.448594 0.459141 -1.494644
H 3.356842 1.710535 -1.160718
H -1.025760 2.470843 0.726621
H -3.295187 1.607562 0.955306
H 2.191660 2.571163 0.692409
31

BenPy6-D

O -4.040377 1.821168 0.134468
C -4.695275 0.586528 -0.130052
C -4.164717 -0.551676 0.729010
N -2.750950 -0.847421 0.528423
C -1.807828 -0.000128 1.138327
C -0.538030 0.111514 0.714330
C -0.050551 -0.595844 -0.475934
C 1.237720 -0.611865 -0.975251
C 2.365669 0.009267 -0.203316
C 2.512818 -0.112391 1.185440
C 3.609151 0.438121 1.842722
C 4.589929 1.120130 1.127059

C 4.470925 1.228782 -0.257005
C 3.380145 0.668834 -0.911361
O 1.557280 -1.119952 -2.135632
C -1.135941 -1.245241 -1.221787
C -2.383648 -1.338999 -0.736343
H -4.138985 2.006632 1.075205
H -5.776426 0.675281 0.026507
H -4.520459 0.370884 -1.186621
H -4.313346 -0.313009 1.785653
H -4.742810 -1.453343 0.509742
H -2.178541 0.546494 1.996602
H 0.108899 0.786584 1.260002
H 1.775236 -0.665129 1.755647
H 3.704074 0.321884 2.916603
H 5.443094 1.549173 1.639404
H 5.233897 1.746445 -0.827753
H 3.299993 0.733600 -1.990065
H -0.923319 -1.682995 -2.188754
H -3.181354 -1.832767 -1.279677
33
BenPy6-E
O 4.562029 1.162137 0.985297
C 4.876945 0.357227 -0.136095
C 3.845553 0.500849 -1.243673
N 2.509849 0.106388 -0.746983
C 2.122684 -1.178407 -0.831789
C 0.923559 -1.590463 -0.292915
C 0.108163 -0.665165 0.351909
C -1.216327 -1.068801 0.972712
C -2.352933 -0.211584 0.447094
C -2.580133 -0.123352 -0.928201
C -3.645724 0.624694 -1.413710
C -4.486379 1.296864 -0.528176
C -4.259583 1.216024 0.841576
C -3.194971 0.461155 1.328413
O -1.475973 -2.451467 0.818491
C 0.538127 0.660300 0.434373
C 1.744972 1.020585 -0.115936
H 4.633978 2.085979 0.717649
H 5.855672 0.609032 -0.553725
H 4.901168 -0.676122 0.213799
H 3.776020 1.534733 -1.580135
H 4.092455 -0.139108 -2.089080
H 2.800137 -1.850044 -1.342410
H 0.636656 -2.628972 -0.376783
H -1.918995 -0.639972 -1.618618
H -3.818188 0.689516 -2.481465

H -5.314417 1.883890 -0.907486
H -4.910382 1.738491 1.532635
H -3.016521 0.392383 2.396123
H -0.063293 1.415631 0.924704
H 2.131388 2.029577 -0.086679
H -1.728765 -2.617149 -0.098824
H -1.122931 -0.901722 2.049275
30
BenPy6_site1
O -4.419064 1.398510 -1.280262
C -4.401737 1.434338 0.187157
C -4.180268 0.032383 0.663911
N -2.734222 -0.354654 0.452178
C -1.805745 0.158870 1.273642
C -0.464283 -0.070919 1.052470
C -0.084265 -0.823953 -0.057877
C 1.355049 -1.177356 -0.332673
C 2.420356 -0.170162 -0.094847
C 3.727561 -0.629063 0.097828
C 4.759764 0.277281 0.285224
C 4.495877 1.646182 0.259436
C 3.200211 2.107365 0.049061
C 2.158445 1.202665 -0.121006
O 1.605608 -2.292631 -0.743118
C -1.063172 -1.332981 -0.902844
C -2.391256 -1.072293 -0.624701
H -5.057200 0.730059 -1.567612
H -5.417867 1.688948 0.534222
H -4.369010 -0.059580 1.734545
H -4.733813 -0.760843 0.134522
H -2.177229 0.755653 2.095278
H 0.262926 0.329093 1.746394
H 3.917919 -1.695504 0.108259
H 5.769586 -0.078541 0.448668
H 5.304231 2.353838 0.401006
H 2.999299 3.171031 0.016995
H 1.154241 1.569313 -0.297965
H -0.800600 -1.920976 -1.771723
H -3.203821 -1.429404 -1.242194
30
BenPy6_site2
O -5.349346 -0.941551 -0.362059
C -5.109646 0.453717 -0.155773
C -3.920764 0.739154 0.692238
N -2.696268 0.419867 0.320201
C -1.590446 0.725264 1.135194
C -0.318351 0.459875 0.748174

C -0.022914 -0.160098 -0.490806
C 1.303762 -0.520320 -0.944400
C 2.511039 0.037800 -0.249409
C 3.609682 -0.803159 -0.054995
C 4.768077 -0.318055 0.538380
C 4.848794 1.020747 0.915558
C 3.767909 1.870249 0.699393
C 2.598853 1.380670 0.125238
O 1.483329 -1.288652 -1.898028
C -1.145887 -0.515341 -1.269418
C -2.421148 -0.244420 -0.889415
H -5.442372 -1.348302 0.506997
H -4.982032 0.878447 -1.156949
H -5.977273 0.929080 0.305558
H -4.014082 1.241489 1.644285
H -1.842135 1.186371 2.078529
H 0.466584 0.718885 1.447491
H 3.543263 -1.839154 -0.366521
H 5.609777 -0.980489 0.701864
H 5.755154 1.401998 1.370952
H 3.834789 2.915959 0.974862
H 1.765157 2.049586 -0.054876
H -1.002168 -1.026641 -2.213482
H -3.280347 -0.538768 -1.467666
30
BenPy6_site3
O -2.744948 1.272499 1.013742
C -4.117687 0.854220 0.945387
C -4.284911 0.259156 -0.460697
N -2.914234 -0.149727 -0.791066
C -2.542797 -1.431452 -0.404977
C -1.297744 -1.739576 -0.002910
C -0.314220 -0.674188 0.086039
C 1.083973 -1.041071 0.464869
C 2.232928 -0.190724 0.035737
C 3.368246 -0.160792 0.851340
C 4.478361 0.580921 0.473979
C 4.471917 1.275820 -0.734331
C 3.353756 1.229921 -1.560938
C 2.230973 0.504734 -1.176318
O 1.275009 -2.035489 1.144441
C -0.688256 0.609481 -0.063113
C -2.128841 0.946824 -0.260282
H -4.285758 0.099356 1.716675
H -4.776097 1.707546 1.111501
H -4.965862 -0.587586 -0.498125
H -4.607640 1.020901 -1.170605

H -3.315015 -2.182835 -0.522362
H -1.016946 -2.761676 0.204622
H 3.364142 -0.713769 1.783149
H 5.349598 0.616122 1.116744
H 5.341313 1.850005 -1.032147
H 3.354438 1.757921 -2.506738
H 1.366808 0.461900 -1.827431
H 0.007581 1.432777 0.035215
H -2.252226 1.813004 -0.914285
43

AZON3

C 2.571351 -1.850468 1.105741
C 3.780272 -2.518819 0.889317
C 4.793419 -1.958731 0.112403
C 4.627296 -0.704474 -0.475731
C 3.429938 -0.045238 -0.260192
C 2.410453 -0.607822 0.521169
H 3.932576 -3.493768 1.336324
H 5.717473 -2.503752 -0.034335
H 5.405633 -0.255404 -1.081558
C 1.276156 0.341126 0.557626
C 0.052783 0.278166 1.180394
N -0.798591 1.320861 1.044298
C -0.499498 2.411827 0.318061
C 0.721951 2.525470 -0.329693
C 1.597755 1.473310 -0.194515
H -0.285638 -0.553287 1.784697
C -2.142881 1.244974 1.676912
H -1.258909 3.182103 0.281675
H 0.955407 3.411118 -0.905593
C 2.991641 1.285356 -0.756647
O 3.584355 2.069421 -1.452836
H 1.790618 -2.293530 1.711958
H -2.351620 2.229943 2.088415
C -3.221228 0.852747 0.672955
H -2.069664 0.526619 2.492329
H -3.198575 1.549395 -0.168026
C -3.043349 -0.587919 0.218524
H -4.171282 0.979817 1.194274
H -3.064904 -1.260096 1.078712
N -4.110331 -1.084713 -0.723146
H -2.100097 -0.727215 -0.314377
C -4.098986 -0.313129 -2.001661
C -3.812585 -2.518772 -1.024797
C -5.463756 -0.997748 -0.097270
H -2.822821 -2.578352 -1.473418
H -3.845068 -3.080749 -0.093233

H -4.567560 -2.885302 -1.717455
H -3.094712 -0.356182 -2.420209
H -4.813942 -0.777110 -2.678728
H -4.390681 0.715133 -1.804035
H -5.741022 0.047241 0.015279
H -6.170910 -1.497254 -0.756819
H -5.428870 -1.495688 0.870553
43

AZON3-A

C 2.623900 -1.814665 1.182251
C 3.828928 -2.460389 0.919228
C 4.765939 -1.909968 0.034324
C 4.516257 -0.701340 -0.605820
C 3.312957 -0.050361 -0.346907
C 2.370178 -0.605028 0.543544
H 4.047178 -3.403344 1.406376
H 5.695196 -2.436002 -0.150438
H 5.237429 -0.270189 -1.291628
C 1.230321 0.310568 0.594604
C 0.055472 0.263693 1.283934
N -0.847120 1.275820 1.149441
C -0.603294 2.339871 0.318085
C 0.556194 2.439931 -0.398151
C 1.510531 1.413902 -0.276166
H -0.221808 -0.540514 1.953879
C -2.159058 1.192385 1.813265
H -1.384161 3.088018 0.281992
H 0.718937 3.298672 -1.036931
C 2.802487 1.230614 -0.886415
O 3.383662 1.983298 -1.701599
H 1.903272 -2.245531 1.868351
H -2.373598 2.167208 2.249502
C -3.270526 0.806825 0.839433
H -2.072180 0.464742 2.620258
H -3.285496 1.525400 0.016743
C -3.067833 -0.615713 0.343712
H -4.214329 0.892931 1.380490
H -3.202748 -1.320870 1.165858
N -4.022176 -1.050373 -0.740646
H -2.070725 -0.756982 -0.078458
C -5.440154 -0.829522 -0.330407
C -3.750793 -0.323172 -2.017061
C -3.807466 -2.511733 -0.970992
H -4.418392 -0.722965 -2.778294
H -3.943148 0.738031 -1.880133
H -2.712016 -0.492773 -2.295234
H -5.634780 0.239110 -0.274676

H -6.082649 -1.284499 -1.082226
H -5.595449 -1.300920 0.639061
H -4.057545 -3.044075 -0.055098
H -4.455596 -2.828281 -1.785751
H -2.762462 -2.668833 -1.232372
44
AZON3-B
C 3.359358 -1.548968 -0.319064
C 4.691418 -1.804019 0.006145
C 5.524075 -0.795063 0.505783
C 5.049769 0.496713 0.694670
C 3.717419 0.757007 0.370607
C 2.876249 -0.263348 -0.134207
H 5.090550 -2.801675 -0.130471
H 6.553745 -1.028007 0.747945
H 5.690118 1.280710 1.080640
C 1.562106 0.334756 -0.366070
C 0.390622 -0.185378 -0.838963
N -0.692723 0.620372 -0.965273
C -0.655872 1.931611 -0.614442
C 0.488394 2.516323 -0.129681
C 1.626162 1.712765 0.001509
H 0.262377 -1.219237 -1.133223
C -1.975124 0.034623 -1.403528
H -1.579353 2.478911 -0.746674
H 0.481433 3.565745 0.134069
C 2.952150 1.973232 0.456723
O 3.465819 3.105671 0.901547
H 2.721177 -2.334972 -0.705243
H -1.745465 -0.788125 -2.079881
C -2.778573 -0.443046 -0.197857
H -2.512783 0.802807 -1.958313
H -2.951492 0.406905 0.466061
C -4.089386 -1.020104 -0.706316
H -2.194365 -1.194251 0.338186
H -3.904531 -1.848099 -1.393272
N -4.997371 -1.569636 0.364090
H -4.667810 -0.253917 -1.225217
C -4.358610 -2.719018 1.072076
C -5.365631 -0.514447 1.354570
C -6.243575 -2.052886 -0.305827
H -6.121264 -0.928288 2.019706
H -4.484526 -0.234921 1.926838
H -5.763327 0.342468 0.812676
H -5.092528 -3.139934 1.756721
H -4.062146 -3.458269 0.329628
H -3.495796 -2.364084 1.629784

H -6.719161 -1.207233 -0.799603
H -5.970041 -2.816079 -1.032141
H -6.900461 -2.469337 0.455044
H 2.812664 3.821381 0.903078
44
AZON3-C
C 2.974670 1.012373 -1.817083
C 4.295057 1.430599 -1.745207
C 5.154869 0.927352 -0.749883
C 4.713156 0.004778 0.183204
C 3.378451 -0.427839 0.122023
C 2.509140 0.081548 -0.885178
H 4.672193 2.151808 -2.460445
H 6.182506 1.271260 -0.716327
H 5.381935 -0.376788 0.946650
C 1.224953 -0.546892 -0.677692
C 0.036836 -0.438566 -1.328213
N -1.041650 -1.170027 -0.932693
C -0.954209 -2.017170 0.157984
C 0.191990 -2.180805 0.861842
C 1.357706 -1.442726 0.475649
H -0.117176 0.211197 -2.182583
C -2.340497 -0.926117 -1.570805
H -1.867333 -2.544742 0.399142
H 0.202028 -2.867674 1.699031
C 2.646981 -1.357551 0.937421
O 3.250340 -2.005658 1.995017
H 2.313578 1.400999 -2.584610
H -2.977965 -1.784681 -1.358895
C -2.995660 0.369177 -1.092916
H -2.184539 -0.877224 -2.648296
H -3.909419 0.500994 -1.675333
C -3.304757 0.286872 0.392568
H -2.326517 1.204163 -1.313460
H -2.391202 0.166348 0.978450
N -3.989753 1.502481 0.968092
H -3.969063 -0.555574 0.597201
C -5.303500 1.747643 0.302915
C -4.228751 1.241153 2.420224
C -3.128559 2.715178 0.836048
H -3.267573 1.070907 2.902233
H -4.720410 2.112441 2.848170
H -4.864118 0.362368 2.512592
H -5.801326 2.557489 0.832992
H -5.135725 2.032736 -0.732456
H -5.894205 0.834814 0.361403
H -2.157095 2.496038 1.276584

H -3.024735 2.973104 -0.214737
H -3.613012 3.530745 1.370174
H 2.595782 -2.550915 2.446591
43
AZON3-D
C 2.843566 1.065579 -1.743089
C 4.142835 1.542885 -1.609729
C 4.971019 1.084686 -0.568828
C 4.514411 0.147103 0.346668
C 3.208273 -0.344274 0.223795
C 2.373538 0.123183 -0.827795
H 4.523456 2.274178 -2.313338
H 5.980492 1.472523 -0.485812
H 5.154580 -0.206048 1.149122
C 1.105561 -0.564000 -0.676855
C -0.043916 -0.510874 -1.387124
N -1.115827 -1.307825 -1.053421
C -1.041308 -2.134356 0.074314
C 0.077555 -2.232027 0.826394
C 1.235544 -1.446399 0.492008
H -0.187366 0.129230 -2.251276
C -2.414201 -1.019109 -1.657420
H -1.941398 -2.698545 0.280662
H 0.081849 -2.907025 1.674769
C 2.501709 -1.332229 1.056422
O 3.018112 -1.934488 2.091482
H 2.207843 1.421205 -2.547951
H -3.079787 -1.852703 -1.429491
C -3.019998 0.299969 -1.168893
H -2.292319 -0.973818 -2.740914
H -3.985393 0.423374 -1.663938
C -3.176189 0.272390 0.341828
H -2.369748 1.121588 -1.478354
H -2.205268 0.170292 0.830805
N -3.796991 1.509960 0.943177
H -3.813725 -0.559756 0.648218
C -5.176468 1.732204 0.418371
C -3.874085 1.302734 2.421078
C -2.957978 2.714007 0.670627
H -2.865871 1.142061 2.798933
H -4.311586 2.192219 2.870361
H -4.498068 0.431938 2.614424
H -5.616864 2.558690 0.973306
H -5.123455 1.983366 -0.637909
H -5.753532 0.821344 0.570442
H -1.942294 2.508380 1.005671
H -2.975428 2.930573 -0.394393

H -3.379679 3.550591 1.224930
45
AZON3-E
C 2.980999 1.027322 -1.796103
C 4.315096 1.425338 -1.824137
C 5.217422 0.977174 -0.857693
C 4.804174 0.128872 0.169566
C 3.476715 -0.262952 0.203022
C 2.576738 0.173871 -0.776832
H 4.659288 2.087108 -2.609665
H 6.252043 1.294948 -0.906393
H 5.503446 -0.217751 0.921825
C 1.275860 -0.456036 -0.507359
C 0.074941 -0.389295 -1.174745
N -0.961826 -1.135478 -0.732800
C -0.858583 -1.944980 0.337654
C 0.324669 -2.040196 1.050005
C 1.390188 -1.274035 0.625553
H -0.104200 0.220111 -2.051879
C -2.269360 -0.992759 -1.423576
H -1.747059 -2.502205 0.601874
H 0.384026 -2.696933 1.907563
C 2.781954 -1.150887 1.217942
O 3.415669 -2.377551 1.503427
H 2.281695 1.366466 -2.551358
H -2.865341 -1.867707 -1.169847
C -2.971713 0.301554 -1.028152
H -2.066897 -1.000293 -2.492519
H -3.846903 0.382331 -1.674640
C -3.386330 0.262352 0.434709
H -2.311526 1.144692 -1.242698
H -2.527007 0.125795 1.094697
N -4.067865 1.513785 0.928770
H -4.092382 -0.553330 0.602611
C -5.287397 1.818937 0.122255
C -4.475291 1.275104 2.347370
C -3.137810 2.682416 0.891071
H -3.582332 1.053055 2.928892
H -4.958325 2.175578 2.721139
H -5.166841 0.435109 2.370994
H -5.805145 2.648939 0.599597
H -4.991084 2.100099 -0.885599
H -5.920413 0.933016 0.106403
H -2.237339 2.423610 1.445610
H -2.899811 2.921829 -0.141984
H -3.642993 3.526538 1.357054
H 3.557454 -2.840324 0.667962

H 2.697580 -0.623524 2.173768
44
AZON3_site1
O -2.972400 0.586794 2.280107
C -2.888726 0.267510 1.112113
C -4.042940 -0.275043 0.353709
C -5.100566 -0.836974 1.076328
C -6.214550 -1.321596 0.408666
C -6.287724 -1.227500 -0.980658
C -5.245993 -0.652179 -1.701596
C -4.118222 -0.181829 -1.038959
C -1.553981 0.427328 0.426274
C -1.046589 -0.542093 -0.433502
C 0.215568 -0.369361 -0.963325
N 0.935218 0.727097 -0.674606
C 2.319046 0.849303 -1.204350
C 3.314847 0.171942 -0.271683
C 4.690810 0.320682 -0.915933
N 5.779156 -0.207451 0.026914
C 5.496838 -1.557134 0.599691
C 7.039420 -0.295822 -0.755533
C 6.002956 0.742708 1.148922
C 0.467974 1.670724 0.162416
C -0.777696 1.540534 0.736017
H -5.031790 -0.897248 2.155754
H -7.027212 -1.770260 0.966603
H -7.161306 -1.601512 -1.501300
H -5.310679 -0.568853 -2.779341
H -3.315942 0.276789 -1.605128
H -1.604914 -1.433530 -0.685936
H 0.677506 -1.085538 -1.629125
H 2.319123 0.389654 -2.192772
H 2.532065 1.911011 -1.305698
H 3.256712 0.684449 0.696000
H 2.962106 -0.860452 -0.105537
H 4.729891 -0.406466 -1.741068
H 6.361989 -1.900677 1.168325
H 5.290963 -2.240076 -0.223427
H 4.632893 -1.491916 1.257762
H 7.237352 0.681205 -1.190942
H 6.904964 -1.033842 -1.545104
H 7.852841 -0.597580 -0.095679
H 5.093006 0.820123 1.740171
H 6.256756 1.712390 0.726975
H 6.814528 0.370352 1.775122
H 1.120478 2.513602 0.347170
H -1.139585 2.303025 1.412279

44

AZON3_site2

O -0.379315 1.644325 0.990034
C -1.014589 0.890940 0.274486
C -2.325450 0.337787 0.709710
C -2.602651 0.295286 2.079869
C -3.825867 -0.181476 2.526603
C -4.787071 -0.598468 1.606826
C -4.523007 -0.540578 0.241934
C -3.291159 -0.080576 -0.209813
C -0.444887 0.507278 -1.059893
C -0.530853 -0.789041 -1.558916
C 0.132858 -1.084278 -2.744656
N 0.829080 -0.188131 -3.448594
C 4.160742 1.251761 -2.298544
C 4.696894 0.294216 -1.551088
C 4.743469 0.385791 -0.056916
N 3.801196 -0.577868 0.636429
C 4.152414 -1.989288 0.305312
C 3.931268 -0.374103 2.109162
C 2.392838 -0.305391 0.225410
C 0.894863 1.055850 -2.960854
C 0.292947 1.448119 -1.773766
H -1.848816 0.629282 2.782628
H -4.033657 -0.227345 3.588643
H -5.744654 -0.966300 1.956094
H -5.275338 -0.853316 -0.471625
H -3.094570 -0.026744 -1.273890
H -1.074008 -1.564793 -1.033599
H 0.097874 -2.091325 -3.145165
H 4.159517 1.188973 -3.381081
H 3.714466 2.134026 -1.847661
H 5.148792 -0.578379 -2.012910
H 5.737773 0.148056 0.325125
H 4.453499 1.381166 0.280885
H 3.497642 -2.642379 0.879504
H 5.193308 -2.158583 0.576205
H 4.000335 -2.153364 -0.759212
H 3.658728 0.653996 2.340655
H 4.963277 -0.570037 2.395272
H 3.259072 -1.067797 2.610893
H 2.296334 -0.518427 -0.837093
H 2.171229 0.741085 0.432038
H 1.738513 -0.956502 0.804273
H 1.457984 1.774351 -3.546179
H 0.393776 2.463859 -1.411628

44

AZON3_site3

O -3.896923 2.325560 0.071062
C -3.260151 1.263903 -0.008421
C -4.037200 -0.020674 -0.036525
C -5.162129 -0.129461 0.785326
C -5.943313 -1.277642 0.759177
C -5.623481 -2.317636 -0.111114
C -4.520894 -2.204576 -0.952471
C -3.725630 -1.063200 -0.912780
C -1.819365 1.238911 -0.066242
C -1.017801 0.093738 0.129945
C 0.342216 0.138498 0.115132
N 1.036249 1.343241 -0.119042
C 2.344232 1.409495 -0.184125
C 3.209287 0.207603 -0.037074
C 4.656646 0.639964 -0.218547
N 5.667333 -0.470732 -0.112513
C 5.435120 -1.514645 -1.155143
C 7.017323 0.134380 -0.332765
C 5.641646 -1.100541 1.241636
C 0.245355 2.507098 -0.273957
C -1.102171 2.454924 -0.243426
H -5.413316 0.691007 1.447451
H -6.804236 -1.360338 1.411897
H -6.237043 -3.210446 -0.138415
H -4.280641 -3.003317 -1.644037
H -2.877425 -0.973725 -1.581734
H -1.463931 -0.871302 0.336092
H 0.952656 -0.731754 0.289534
H 2.766469 2.391492 -0.354267
H 3.051781 -0.245589 0.949728
H 2.924980 -0.543101 -0.784409
H 4.799699 1.081059 -1.206113
H 4.934407 1.372006 0.540990
H 6.265553 -2.217305 -1.115252
H 5.395538 -1.026110 -2.127475
H 4.503282 -2.033899 -0.945382
H 7.184059 0.892228 0.430317
H 7.036940 0.579967 -1.325568
H 7.761561 -0.655441 -0.255356
H 4.703474 -1.633562 1.374013
H 5.748977 -0.315059 1.988115
H 6.474006 -1.799760 1.301169
H 0.809785 3.415588 -0.424403
H -1.644750 3.383484 -0.371188
45

BenPy7

O -2.996850 0.774273 2.230734
C -2.910085 0.370465 1.089824
C -4.064311 -0.205394 0.357683
C -5.144462 -0.689234 1.103139
C -6.257427 -1.202012 0.455084
C -6.306637 -1.213599 -0.938460
C -5.241994 -0.716073 -1.683404
C -4.115325 -0.218605 -1.039203
C -1.566284 0.457456 0.407136
C -1.059254 -0.595402 -0.348161
C 0.207254 -0.484389 -0.881946
N 0.930654 0.633190 -0.697251
C 2.304105 0.704059 -1.248335
C 3.304051 0.192503 -0.217261
C 4.691383 0.293887 -0.829282
N 5.806928 -0.207619 0.049973
C 5.642783 -1.660465 0.353667
C 7.087404 -0.017003 -0.699196
C 5.877897 0.563612 1.326462
C 0.464163 1.659279 0.039415
C -0.785328 1.591078 0.614311
H -5.094092 -0.667675 2.185054
H -7.088009 -1.590761 1.031261
H -7.179522 -1.609380 -1.443967
H -5.287894 -0.714803 -2.765313
H -3.294198 0.178578 -1.624593
H -1.622457 -1.503443 -0.516907
H 0.669900 -1.265635 -1.469183
H 2.319423 0.101141 -2.154959
H 2.493148 1.744145 -1.509320
H 3.222799 0.803813 0.683977
H 3.052788 -0.840485 0.032492
H 4.749853 -0.292529 -1.747705
H 4.933406 1.333182 -1.057232
H 6.535118 -1.994505 0.879714
H 5.531156 -2.198183 -0.586407
H 4.768709 -1.800284 0.984693
H 7.209131 1.044716 -0.906580
H 7.030899 -0.584649 -1.626282
H 7.903698 -0.380932 -0.078442
H 4.999185 0.346478 1.928519
H 5.929626 1.624441 1.086320
H 6.774864 0.249416 1.856983
H 1.118787 2.514502 0.142279
H -1.147385 2.418337 1.209554
45
BenPy7-A

O -3.035290 -1.722787 -1.541338
C -2.578208 -0.935401 -0.676955
C -3.452909 0.190488 -0.217531
C -2.968930 1.488378 -0.026668
C -3.834969 2.516963 0.330818
C -5.191565 2.260334 0.506795
C -5.683739 0.972402 0.306436
C -4.822055 -0.051495 -0.065989
C -1.263774 -1.114433 -0.127551
C -0.393010 -2.090859 -0.708951
C 0.849126 -2.308728 -0.209091
N 1.308251 -1.630398 0.892294
C 2.703419 -1.797529 1.310800
C 3.676701 -1.070668 0.382769
C 3.429095 0.427985 0.432897
N 4.355667 1.254856 -0.423662
C 4.206438 0.901825 -1.866970
C 3.982170 2.690390 -0.238542
C 5.778565 1.072760 -0.009953
C 0.495201 -0.703317 1.492097
C -0.753798 -0.437900 1.023052
H -1.917625 1.702119 -0.182445
H -3.450159 3.521136 0.464367
H -5.863722 3.061428 0.790772
H -6.740147 0.768340 0.435940
H -5.201883 -1.051929 -0.238190
H -0.719060 -2.660312 -1.568190
H 1.536872 -3.027332 -0.636430
H 2.788373 -1.427597 2.332795
H 2.928916 -2.863832 1.311923
H 4.685032 -1.312393 0.723795
H 3.555503 -1.459822 -0.630728
H 2.422665 0.673120 0.087694
H 3.551460 0.800120 1.452350
H 4.812753 1.595650 -2.446750
H 3.155871 0.995269 -2.137764
H 4.556114 -0.115314 -2.024756
H 4.109004 2.946727 0.811855
H 2.944307 2.818397 -0.540871
H 4.637213 3.297811 -0.860017
H 6.383772 1.786424 -0.566022
H 6.094920 0.059580 -0.244479
H 5.853275 1.263334 1.059521
H 0.905986 -0.213225 2.364965
H -1.353468 0.277788 1.567937
46
BenPy7-B

O -3.303363 1.354442 1.899895
C -2.939722 0.644549 0.824513
C -4.017522 -0.171891 0.274173
C -4.989363 -0.694789 1.141348
C -6.038526 -1.450658 0.639677
C -6.143809 -1.680839 -0.730279
C -5.197162 -1.144682 -1.599978
C -4.140844 -0.392757 -1.105994
C -1.625346 0.726810 0.301826
C -1.098643 -0.262377 -0.573879
C 0.176719 -0.156679 -1.045847
N 0.969606 0.893856 -0.711890
C 2.359144 0.947370 -1.195345
C 3.291843 0.222140 -0.229124
C 4.702430 0.312007 -0.787455
N 5.766798 -0.331415 0.064439
C 5.517399 -1.794694 0.225609
C 7.076558 -0.143419 -0.632771
C 5.843209 0.312583 1.409780
C 0.515090 1.842542 0.143204
C -0.746786 1.787461 0.659670
H -4.904187 -0.516953 2.205986
H -6.775838 -1.862237 1.318201
H -6.966124 -2.268851 -1.119906
H -5.289136 -1.301770 -2.667732
H -3.432054 0.052694 -1.793660
H -1.674450 -1.133054 -0.851878
H 0.617177 -0.897142 -1.700216
H 2.379652 0.488468 -2.183424
H 2.630816 1.998070 -1.292172
H 3.221672 0.700023 0.750491
H 2.964398 -0.816049 -0.139096
H 4.761010 -0.179203 -1.760173
H 5.001829 1.355521 -0.900862
H 6.373483 -2.224427 0.742910
H 5.408211 -2.236503 -0.763577
H 4.617046 -1.943272 0.816887
H 7.258179 0.923897 -0.743953
H 7.020675 -0.626210 -1.606807
H 7.854716 -0.601227 -0.025130
H 4.933175 0.096702 1.964046
H 5.966307 1.385644 1.271622
H 6.701688 -0.105929 1.932029
H 1.202371 2.646140 0.372418
H -1.058667 2.598509 1.304099
H -2.544016 1.695077 2.392971
46

BenPy7-C

O -2.501815 -1.286064 -1.963467
C -2.321467 -0.694074 -0.709956
C -3.402935 0.197528 -0.272761
C -3.760376 0.341400 1.078310
C -4.803623 1.178214 1.453549
C -5.530830 1.877687 0.492690
C -5.208167 1.720959 -0.852505
C -4.162055 0.889021 -1.232165
C -1.137714 -0.930588 -0.083742
C -0.173652 -1.892214 -0.614002
C 0.991040 -2.145097 0.016385
N 1.352944 -1.523917 1.192404
C 2.722950 -1.610089 1.695761
C 3.576953 -0.401881 1.302668
C 3.617221 -0.263775 -0.209935
N 4.425728 0.902705 -0.721750
C 4.381490 0.865201 -2.215235
C 3.842339 2.196528 -0.257962
C 5.849051 0.807479 -0.282275
C 0.526123 -0.525985 1.667089
C -0.658852 -0.223580 1.100462
H -3.237683 -0.229713 1.836058
H -5.062505 1.269683 2.502189
H -6.346611 2.526224 0.788714
H -5.771580 2.251377 -1.611606
H -3.917989 0.778565 -2.282468
H -0.396650 -2.433206 -1.522114
H 1.704183 -2.866601 -0.363884
H 2.689844 -1.686218 2.783433
H 3.159075 -2.531294 1.307410
H 3.157809 0.494138 1.766197
H 4.577197 -0.558912 1.711347
H 4.052869 -1.155763 -0.665356
H 2.613597 -0.117687 -0.615144
H 4.949429 1.711870 -2.596202
H 3.341454 0.931082 -2.529971
H 4.823597 -0.071586 -2.549687
H 3.952241 2.273439 0.820760
H 2.790998 2.220434 -0.541419
H 4.387167 3.003899 -0.743633
H 6.234436 -0.168625 -0.572976
H 5.901749 0.937056 0.795636
H 6.407339 1.599936 -0.777323
H 0.893117 0.002005 2.538927
H -1.232498 0.582344 1.535467
H -3.431177 -1.527985 -2.057698

45
BenPy7-D
O -2.466258 -1.351374 -1.961706
C -2.214467 -0.778459 -0.816736
C -3.264667 0.173375 -0.319374
C -3.658582 0.260165 1.022908
C -4.676439 1.123609 1.418097
C -5.330392 1.917642 0.479641
C -4.967027 1.825124 -0.862420
C -3.957692 0.954425 -1.254969
C -1.050351 -0.982451 -0.100550
C -0.048708 -1.943556 -0.582463
C 1.061411 -2.234534 0.113608
N 1.344832 -1.674199 1.370744
C 2.738048 -1.619595 1.805705
C 3.506865 -0.375133 1.343109
C 3.493911 -0.272223 -0.172046
N 4.260082 0.901200 -0.736468
C 4.139778 0.847089 -2.225011
C 3.683807 2.191865 -0.255865
C 5.705257 0.831183 -0.370251
C 0.527430 -0.588613 1.743537
C -0.600050 -0.257966 1.090754
H -3.181431 -0.371724 1.763153
H -4.968167 1.165975 2.461552
H -6.122154 2.590263 0.788135
H -5.476290 2.429414 -1.604915
H -3.692084 0.868263 -2.302097
H -0.214204 -2.446792 -1.526241
H 1.797055 -2.947934 -0.240148
H 2.761228 -1.648218 2.897201
H 3.232236 -2.522943 1.443166
H 3.050929 0.510151 1.792368
H 4.527600 -0.458908 1.722636
H 3.933678 -1.163106 -0.625982
H 2.474758 -0.156540 -0.545495
H 4.681130 1.692911 -2.644473
H 3.084704 0.902381 -2.487118
H 4.570791 -0.090301 -2.571991
H 3.858862 2.286502 0.812651
H 2.616524 2.194014 -0.473321
H 4.182860 3.000975 -0.785975
H 6.089986 -0.142307 -0.670332
H 5.813398 0.974099 0.701810
H 6.225563 1.625443 -0.902660
H 0.864573 -0.046314 2.618965
H -1.156578 0.592164 1.464591

47
BenPy7-E
O -3.099812 -2.463792 -0.913223
C -2.797325 -1.629340 0.185878
C -3.150758 -0.172844 -0.075789
C -3.739945 0.599934 0.921469
C -4.037359 1.939538 0.682951
C -3.750554 2.506924 -0.554335
C -3.164261 1.733359 -1.554417
C -2.862014 0.397884 -1.316022
C -1.315234 -1.738412 0.458685
C -0.443202 -2.308034 -0.463294
C 0.905721 -2.353279 -0.180506
N 1.383403 -1.866007 0.977269
C 2.843280 -1.866436 1.245919
C 3.443355 -0.474632 1.082089
C 3.310636 0.002171 -0.355514
N 3.888062 1.369341 -0.620814
C 3.750485 1.638096 -2.085393
C 3.139717 2.420860 0.131181
C 5.334913 1.428713 -0.255427
C 0.556994 -1.308899 1.887356
C -0.793362 -1.234540 1.652377
H -3.972150 0.150124 1.881177
H -4.499933 2.535625 1.460622
H -3.986301 3.547773 -0.741905
H -2.941918 2.172211 -2.519904
H -2.405987 -0.207503 -2.091910
H -0.798937 -2.717352 -1.397354
H 1.631708 -2.776322 -0.861849
H 2.980648 -2.218008 2.266568
H 3.295529 -2.581597 0.560869
H 2.957690 0.211095 1.779597
H 4.493046 -0.561588 1.367359
H 3.828382 -0.682285 -1.030135
H 2.263568 0.070222 -0.658020
H 4.130751 2.637643 -2.286168
H 2.696609 1.571602 -2.349729
H 4.329471 0.893270 -2.628498
H 3.291565 2.277584 1.198016
H 2.084265 2.340302 -0.124149
H 3.531654 3.390533 -0.169944
H 5.848109 0.599452 -0.739948
H 5.435803 1.368317 0.825445
H 5.729656 2.379617 -0.608597
H 1.020772 -0.943264 2.794077
H -1.436684 -0.787456 2.400072

H -4.035424 -2.352017 -1.118162
H -3.325606 -1.961330 1.086646
26
AZON3_Me
C 2.999162 0.977222 -1.846294
C 4.326845 1.372028 -1.790537
C 5.182924 0.875903 -0.787031
C 4.729000 -0.015684 0.169083
C 3.385248 -0.425378 0.125666
C 2.519352 0.077222 -0.890356
H 4.713860 2.069034 -2.524319
H 6.217007 1.201225 -0.766572
H 5.395171 -0.392040 0.937472
C 1.227227 -0.523506 -0.665462
C 0.036683 -0.410928 -1.316936
N -1.051976 -1.100414 -0.891846
C -0.973784 -1.930524 0.211882
C 0.171652 -2.098617 0.915849
C 1.348077 -1.392850 0.506434
H -0.105410 0.217823 -2.187595
C -2.336823 -0.984991 -1.582658
H -1.896800 -2.433365 0.467542
H 0.172375 -2.762402 1.771710
C 2.641353 -1.321018 0.963730
O 3.230480 -1.959368 2.036875
H 2.341449 1.360200 -2.619615
H 2.570676 -2.513079 2.470285
H -2.651544 -1.963546 -1.946128
H -3.091599 -0.593371 -0.900138
H -2.227102 -0.306793 -2.425243
28
BenPy7_Me
O -2.511102 -1.290565 -1.960568
C -2.323214 -0.682412 -0.713687
C -3.405343 0.206473 -0.278538
C -3.735621 0.384687 1.076225
C -4.783041 1.215787 1.451873
C -5.543951 1.876380 0.489326
C -5.249930 1.684499 -0.857840
C -4.199560 0.858295 -1.238574
C -1.132193 -0.908435 -0.093389
C -0.175080 -1.880457 -0.614184
C 0.991538 -2.129824 0.015967
N 1.364873 -1.484596 1.170637
C 2.706348 -1.656265 1.714674
C 0.548513 -0.480398 1.636669
C -0.641114 -0.183063 1.073523

H -3.189036 -0.156960 1.838494
H -5.018955 1.333221 2.503382
H -6.362525 2.521119 0.785837
H -5.839554 2.183266 -1.618598
H -3.979628 0.720478 -2.290829
H -0.405572 -2.436337 -1.511217
H 1.697461 -2.864174 -0.351558
H 0.928023 0.064214 2.492207
H -1.204933 0.636340 1.495802
H -3.431372 -1.573121 -2.027643
H 2.683614 -1.476570 2.788514
H 3.034882 -2.679014 1.536598
H 3.415117 -0.965912 1.250303
31

AZON3_Me_TS_prot-HCOOH

C 2.668923 -0.574180 -1.792056
C 3.938738 -0.012676 -1.788554
C 4.461572 0.565588 -0.622411
C 3.736781 0.580796 0.562524
C 2.459697 0.019569 0.569008
C 1.925204 -0.543985 -0.612609
H 4.531360 -0.015642 -2.695392
H 5.451617 1.006311 -0.648328
H 4.148870 1.021866 1.463053
C 0.575727 -0.995790 -0.301010
C -0.392852 -1.605074 -1.060704
N -1.597031 -1.875586 -0.514870
C -1.880206 -1.562159 0.778261
C -0.954909 -0.969249 1.595921
C 0.306469 -0.679384 1.061023
H -0.259399 -1.879200 -2.099192
C -2.648775 -2.524899 -1.316157
H -2.879237 -1.813909 1.107501
H -1.209160 -0.737562 2.622029
C 1.512951 -0.189900 1.673782
O 1.500119 0.654788 2.793319
H 2.259409 -1.016441 -2.693566
H 1.937088 -1.378953 2.130652
H 1.498143 0.065188 3.572823
H -3.532337 -1.889677 -1.323204
H -2.283360 -2.661676 -2.329701
H -2.888337 -3.490465 -0.874195
O 1.449850 -1.465219 4.532660
C 1.970117 -2.425823 3.954022
O 2.339016 -2.455721 2.730668
H 2.147299 -3.358344 4.512382
31

AZON3_Me_min2-HCOOH
C -1.606289 2.733480 -0.205558
C -0.891256 3.925800 -0.129984
C 0.492974 3.919843 0.053481
C 1.197162 2.720142 0.154780
C 0.489602 1.532606 0.073761
C -0.900960 1.540958 -0.094834
H -1.415602 4.870426 -0.209242
H 1.026045 4.860998 0.116908
H 2.272229 2.714521 0.294258
C -1.363533 0.145468 -0.082055
C -2.623363 -0.399864 -0.168265
N -2.766406 -1.739158 -0.061839
C -1.715977 -2.558594 0.130683
C -0.427286 -2.061312 0.214411
C -0.256941 -0.696989 0.094069
H -3.529769 0.173212 -0.312279
C -4.120552 -2.312042 -0.205189
H -1.941716 -3.612794 0.210539
H 0.403952 -2.737622 0.366805
C 1.023258 0.117025 0.128716
O 1.837119 -0.126837 1.248735
H -2.681862 2.735708 -0.339188
H 1.578528 -0.097573 -0.796188
H 2.386378 -0.928391 1.014048
H -4.131195 -3.300774 0.243110
H -4.826533 -1.661461 0.303994
H -4.361906 -2.374189 -1.264890
O 3.145101 -2.247265 0.474905
C 3.841928 -1.945043 -0.536963
O 3.932729 -0.828188 -1.073122
H 4.427822 -2.777772 -0.976863
33
BenPy7_Me_TS_prot-HCOOH
C -3.237809 0.078650 1.136217
C -4.371046 0.007469 0.171975
C -5.202286 -1.118553 0.127118
C -6.278379 -1.174884 -0.752150
C -6.551974 -0.106413 -1.602179
C -5.744789 1.027049 -1.553740
C -4.673413 1.087789 -0.669036
C -1.886785 0.223542 0.687806
C -0.815817 -0.032343 1.590305
C 0.477276 0.207457 1.230706
N 0.797866 0.680815 0.001944
C 2.194446 0.991010 -0.328756
C -0.189534 0.919959 -0.894382

C -1.503455 0.708456 -0.592975
H -4.999589 -1.951941 0.788279
H -6.905418 -2.059097 -0.774255
H -7.391260 -0.151527 -2.286010
H -5.959158 1.875080 -2.194014
H -4.076717 1.992409 -0.612103
H -1.018198 -0.419849 2.578097
H 1.309030 0.029279 1.899595
H 0.130922 1.275675 -1.864650
H -2.234734 0.898321 -1.365300
H -3.355744 1.387245 1.671827
O -3.390911 -0.784561 2.248982
H -3.393591 -0.215647 3.040483
H 2.323852 0.918615 -1.405586
H 2.437975 1.999101 0.005796
H 2.841977 0.271146 0.166358
O -3.166404 1.274392 4.092647
C -3.342408 2.333708 3.491432
O -3.500906 2.459637 2.221685
H -3.374638 3.282901 4.045748
33
BenPy7_Me_min2-HCOOH
C 0.922796 0.767331 0.358091
C 1.944095 -0.319837 0.073690
C 2.297765 -0.623142 -1.241289
C 3.211124 -1.638896 -1.501570
C 3.771955 -2.362625 -0.451426
C 3.418106 -2.064219 0.860689
C 2.507698 -1.043644 1.122278
C -0.466652 0.173343 0.199151
C -1.201251 0.358120 -0.967857
C -2.446777 -0.220109 -1.085936
N -2.961999 -0.954495 -0.085458
C -4.294549 -1.573559 -0.220196
C -2.272536 -1.143855 1.059024
C -1.026631 -0.591754 1.224484
H 1.861448 -0.058079 -2.057198
H 3.485704 -1.866912 -2.524869
H 4.483762 -3.153536 -0.656207
H 3.856087 -2.619220 1.681976
H 2.238580 -0.800944 2.145032
H -0.817986 0.956955 -1.782032
H -3.061074 -0.112524 -1.969080
H -2.762415 -1.740854 1.816713
H -0.497040 -0.749654 2.155823
H 1.021449 1.072761 1.405390
O 1.127421 1.852753 -0.512090

H 0.382643 2.500238 -0.363839
H -4.182085 -2.655021 -0.176548
H -4.923638 -1.228191 0.597190
H -4.720286 -1.277354 -1.173750
O -0.948956 3.442454 -0.218723
C -1.501924 3.318672 0.909763
O -1.043602 2.737990 1.909594
H -2.503077 3.787938 1.004344
34
AZON3_Me_TS_prot-CH3COOH
C 2.645877 -0.592743 -1.809885
C 3.922941 -0.047329 -1.807970
C 4.456910 0.518006 -0.640853
C 3.736216 0.535606 0.546966
C 2.452739 -0.009650 0.555075
C 1.907514 -0.559194 -0.627325
H 4.512198 -0.052527 -2.716993
H 5.452127 0.946794 -0.667869
H 4.157164 0.966543 1.448339
C 0.552404 -0.993973 -0.313864
C -0.428082 -1.578335 -1.077673
N -1.636083 -1.831925 -0.532503
C -1.910845 -1.526972 0.763948
C -0.972695 -0.957479 1.584857
C 0.291974 -0.685425 1.050368
H -0.300573 -1.843281 -2.118952
C -2.695138 -2.466587 -1.336355
H -2.913027 -1.765510 1.093616
H -1.220156 -0.730383 2.613723
C 1.508521 -0.215287 1.665740
O 1.500360 0.641369 2.777222
H 2.227178 -1.024733 -2.712141
H 1.927909 -1.387718 2.133025
H 1.525901 0.054055 3.560479
H -3.595195 -1.858129 -1.277679
H -2.361250 -2.534255 -2.367669
H -2.892935 -3.462401 -0.943223
O 1.517594 -1.421559 4.539501
C 2.025091 -2.415217 3.992158
O 2.353170 -2.460727 2.750563
C 2.292984 -3.671054 4.782328
H 3.355713 -3.911690 4.721228
H 1.998434 -3.545676 5.821825
H 1.739955 -4.498456 4.334268
34
AZON3_Me_min2-CH3COOH
C -2.077153 2.761074 -0.342150

C -1.418594 3.988023 -0.335524
C -0.032031 4.055926 -0.185339
C 0.730828 2.896247 -0.048013
C 0.079218 1.674692 -0.060934
C -1.313101 1.609144 -0.196577
H -1.989628 4.902315 -0.443411
H 0.456196 5.023168 -0.176569
H 1.808069 2.945587 0.064834
C -1.705256 0.194039 -0.116151
C -2.940587 -0.414281 -0.148376
N -3.014146 -1.752313 0.006578
C -1.917998 -2.514182 0.194777
C -0.655392 -1.953709 0.228522
C -0.556332 -0.585457 0.061748
H -3.874685 0.113982 -0.283156
C -4.328836 -2.423843 -0.054534
H -2.095492 -3.574649 0.312747
H 0.213183 -2.582150 0.378877
C 0.683225 0.290385 0.050007
O 1.507755 0.135306 1.177080
H -3.154288 2.707299 -0.449944
H 1.245742 0.065161 -0.867787
H 2.152898 -0.602355 0.950011
H -4.412518 -3.099587 0.793104
H -5.107343 -1.668775 -0.011828
H -4.392516 -2.980129 -0.987776
O 3.090776 -1.775290 0.479503
C 3.869974 -1.359971 -0.435190
O 3.821444 -0.223787 -0.950756
C 4.939524 -2.319754 -0.929410
H 5.921943 -1.883397 -0.737119
H 4.868985 -3.290170 -0.441883
H 4.840717 -2.439940 -2.009811
36
BenPy7_Me_TS_prot-CH3COOH
C -3.244849 0.096388 1.143817
C -4.366801 0.023426 0.163980
C -5.182038 -1.112515 0.091251
C -6.242777 -1.171436 -0.806427
C -6.516183 -0.095523 -1.647128
C -5.725345 1.047769 -1.570575
C -4.669112 1.110506 -0.667581
C -1.888185 0.221785 0.697142
C -0.822396 -0.060294 1.595513
C 0.475334 0.160565 1.236684
N 0.801243 0.639748 0.012652
C 2.200440 0.931262 -0.324467

C -0.182682 0.904282 -0.880258
C -1.499600 0.712172 -0.579011
H -4.979214 -1.951696 0.745323
H -6.857405 -2.063454 -0.850700
H -7.342921 -0.143005 -2.345921
H -5.940167 1.900841 -2.203958
H -4.083055 2.020670 -0.590816
H -1.031047 -0.453284 2.579793
H 1.304305 -0.038614 1.903182
H 0.143204 1.264240 -1.846974
H -2.229255 0.923229 -1.347498
H -3.367304 1.387570 1.676680
O -3.410768 -0.771745 2.251448
H -3.404605 -0.199975 3.042321
H 2.348152 0.761788 -1.388271
H 2.430478 1.967781 -0.079216
H 2.843831 0.265187 0.244546
O -3.180138 1.270348 4.077227
C -3.369769 2.351775 3.506423
O -3.530896 2.469371 2.229064
C -3.422167 3.643292 4.277581
H -4.373090 4.140823 4.080482
H -3.308915 3.460060 5.343539
H -2.624824 4.299811 3.924573
36
BenPy7_Me_min2-CH3COOH
C -1.346674 0.536243 -0.158359
C -2.174397 -0.728692 -0.010762
C -2.438399 -1.255905 1.252983
C -3.163704 -2.436440 1.378360
C -3.623723 -3.100217 0.243659
C -3.358851 -2.576884 -1.018587
C -2.637433 -1.393384 -1.144919
C 0.120623 0.140137 -0.190327
C 0.881323 0.109951 0.977296
C 2.196751 -0.289983 0.919693
N 2.756975 -0.652660 -0.249890
C 4.188241 -1.007820 -0.286508
C 2.051452 -0.606200 -1.395772
C 0.731843 -0.215957 -1.391340
H -2.081768 -0.737129 2.135177
H -3.370146 -2.838923 2.363212
H -4.189255 -4.019113 0.343300
H -3.720124 -3.085177 -1.904871
H -2.437207 -0.977934 -2.127252
H 0.458317 0.406027 1.927281
H 2.837512 -0.336025 1.789878

H 2.579849 -0.889153 -2.295948
H 0.187907 -0.181061 -2.326863
H -1.579571 0.995981 -1.124503
O -1.624781 1.416649 0.903760
H -0.975943 2.167228 0.845573
H 4.359489 -1.675171 -1.126413
H 4.771916 -0.095719 -0.403189
H 4.447118 -1.503911 0.645191
O 0.337092 3.183753 0.778281
C 0.794514 3.169100 -0.405288
O 0.098708 3.041833 -1.436146
C 2.303492 3.248908 -0.563598
H 2.569724 3.767751 -1.484167
H 2.770137 3.729747 0.294907
H 2.683669 2.223449 -0.634039
32

AZON3_Me_TS_prot-H2CO3

C 2.600490 -0.572443 -1.529511
C 3.880062 -0.056566 -1.372146
C 4.308442 0.432415 -0.128993
C 3.475428 0.402445 0.982537
C 2.186398 -0.107136 0.830865
C 1.749130 -0.584684 -0.424384
H 4.555826 -0.027156 -2.218368
H 5.309793 0.836976 -0.036117
H 3.812263 0.768457 1.946195
C 0.366078 -1.011226 -0.260486
C -0.545349 -1.531065 -1.145553
N -1.802775 -1.794864 -0.733774
C -2.195862 -1.553748 0.546400
C -1.334135 -1.052897 1.485067
C -0.014333 -0.783271 1.095253
H -0.322219 -1.736204 -2.184296
C -2.792856 -2.350131 -1.672031
H -3.229543 -1.785798 0.764242
H -1.682498 -0.875955 2.493806
C 1.140042 -0.365223 1.835472
O 1.020624 0.388327 3.012129
H 2.267312 -0.947973 -2.490739
H 1.535765 -1.591373 2.226598
H 1.645758 -0.012709 3.642707
H -3.120572 -3.322722 -1.309125
H -3.641678 -1.671999 -1.736719
H -2.329101 -2.456666 -2.648289
O 2.936503 -1.213910 4.189062
C 2.790788 -2.338857 3.702498
O 2.021747 -2.663434 2.737550

O 3.489294 -3.386540 4.195211
H 4.035717 -3.060763 4.923422
32
AZON3_Me_min2-H2CO3
C -0.634354 3.019568 -0.373006
C 0.535099 3.769549 -0.276607
C 1.755559 3.158783 0.018720
C 1.838200 1.780016 0.214327
C 0.677451 1.031357 0.114547
C -0.548187 1.647666 -0.166652
H 0.497275 4.841561 -0.428123
H 2.649982 3.765595 0.095489
H 2.786201 1.302374 0.436600
C -1.586575 0.606616 -0.145384
C -2.951704 0.676346 -0.318482
N -3.685436 -0.447041 -0.186474
C -3.127493 -1.636542 0.115466
C -1.763123 -1.762458 0.291219
C -0.990276 -0.626052 0.145835
H -3.488080 1.586344 -0.549372
C -5.152285 -0.390039 -0.356797
H -3.812418 -2.468762 0.205415
H -1.336675 -2.727381 0.530974
C 0.508137 -0.471409 0.259230
O 1.004137 -1.033508 1.450077
H -1.583831 3.493091 -0.594469
H 0.962636 -0.968047 -0.610427
H 1.965081 -1.216251 1.277679
H -5.437083 -1.083999 -1.144468
H -5.622324 -0.672731 0.582732
H -5.431595 0.623245 -0.628166
O 3.504763 -1.404677 0.757974
C 3.505759 -2.264323 -0.163169
O 2.553557 -2.905658 -0.619963
O 4.731635 -2.526823 -0.741665
H 5.384198 -1.969056 -0.300659
34
BenPy7_Me_TS_prot-H2CO3
C -3.244151 0.088921 1.154298
C -4.372079 -0.000710 0.185455
C -5.188244 -1.138129 0.143635
C -6.257060 -1.216081 -0.742731
C -6.539050 -0.158335 -1.603352
C -5.748250 0.986571 -1.557269
C -4.683915 1.069047 -0.665663
C -1.893794 0.244645 0.712918
C -0.822164 -0.007685 1.616852

C 0.469904 0.243474 1.261857
N 0.789389 0.735195 0.039944
C 2.195667 0.933988 -0.332763
C -0.197190 0.962881 -0.860494
C -1.509970 0.739607 -0.564642
H -4.979150 -1.963717 0.812679
H -6.871385 -2.109197 -0.762271
H -7.372034 -0.220689 -2.293470
H -5.969725 1.826736 -2.205435
H -4.101157 1.982821 -0.612302
H -1.024674 -0.396191 2.604155
H 1.301252 0.064520 1.931293
H 0.122964 1.323632 -1.828806
H -2.240385 0.932405 -1.336951
H -3.377190 1.421211 1.667901
O -3.397394 -0.769785 2.271615
H -3.385914 -0.200842 3.059794
H 2.252673 1.720197 -1.081384
H 2.754419 1.231521 0.551424
H 2.605662 0.008169 -0.735673
O -3.103258 1.343028 4.061356
C -3.330385 2.380531 3.442308
O -3.537420 2.488647 2.178980
O -3.383588 3.568065 4.073427
H -3.223260 3.412910 5.014754
34
BenPy7_Me_min2-H2CO3
C -1.089907 0.620224 -0.223581
C -2.097058 -0.500784 -0.045107
C -2.500146 -0.888862 1.232259
C -3.395598 -1.941676 1.389920
C -3.887159 -2.616559 0.275139
C -3.482409 -2.232915 -1.000011
C -2.591055 -1.175974 -1.159530
C 0.310175 0.036279 -0.126270
C 1.005880 0.034975 1.080751
C 2.279612 -0.484589 1.128281
N 2.854966 -0.998675 0.025603
C 4.251832 -1.468987 0.086075
C 2.206197 -1.002854 -1.153891
C 0.930970 -0.496852 -1.254165
H -2.118003 -0.361838 2.098766
H -3.710237 -2.235832 2.384374
H -4.585211 -3.435813 0.400208
H -3.866446 -2.750170 -1.871365
H -2.281329 -0.867561 -2.152739
H 0.573266 0.460373 1.975777

H 2.874547 -0.504464 2.031227
H 2.745568 -1.414116 -1.996201
H 0.436080 -0.497395 -2.217149
H -1.198375 1.033551 -1.232287
O -1.306040 1.612765 0.753769
H -0.586439 2.273710 0.652133
H 4.909801 -0.630215 -0.136648
H 4.448991 -1.848504 1.084727
H 4.385269 -2.257796 -0.648832
O 0.942977 3.087008 0.401890
C 1.447533 2.736946 -0.694151
O 0.924470 2.711932 -1.814594
O 2.761183 2.302796 -0.647531
H 3.038559 2.332161 0.277058
34
AZON3_Me_TS_prot-CF3COOH
C 2.664618 -0.633402 -1.423583
C 3.938973 -0.126583 -1.209176
C 4.300980 0.400282 0.040377
C 3.404389 0.420862 1.100583
C 2.118573 -0.079611 0.891220
C 1.749550 -0.599606 -0.370581
H 4.663404 -0.133880 -2.014646
H 5.300820 0.795779 0.178065
H 3.689240 0.819543 2.068125
C 0.357902 -1.011862 -0.271148
C -0.503039 -1.566604 -1.184131
N -1.782519 -1.815111 -0.834682
C -2.247251 -1.523306 0.412514
C -1.441365 -0.985197 1.377573
C -0.098193 -0.729177 1.054234
H -0.221241 -1.814691 -2.199171
C -2.717954 -2.411725 -1.802077
H -3.292221 -1.747202 0.580305
H -1.847193 -0.768453 2.356593
C 1.009891 -0.281792 1.832995
O 0.842479 0.484397 2.993085
H 2.383155 -1.038275 -2.389487
H 1.369223 -1.548719 2.272664
H 1.440461 0.109801 3.660971
H -3.046801 -3.380860 -1.430576
H -3.574190 -1.751009 -1.924059
H -2.208557 -2.534444 -2.753234
O 2.716234 -1.177212 4.303087
C 2.545577 -2.273190 3.793189
O 1.809020 -2.585202 2.802136
C 3.292979 -3.490555 4.372046

F 2.427003 -4.409406 4.813186
F 4.049819 -4.069637 3.434180
F 4.083119 -3.150809 5.386031
34
AZON3_Me_min2-CF3COOH
C 1.842897 -2.581466 -1.015793
C 0.849316 -3.556443 -0.984953
C -0.391346 -3.295133 -0.400457
C -0.673972 -2.048656 0.159142
C 0.312579 -1.076037 0.128782
C 1.560828 -1.345006 -0.447338
H 1.041933 -4.530926 -1.417295
H -1.146317 -4.072230 -0.384373
H -1.639647 -1.842258 0.606710
C 2.397051 -0.147312 -0.285394
C 3.703515 0.104966 -0.636458
N 4.251633 1.295118 -0.311605
C 3.562271 2.242207 0.352309
C 2.244700 2.041351 0.722513
C 1.659261 0.837619 0.384268
H 4.339196 -0.597614 -1.158633
C 5.635239 1.583644 -0.742212
H 4.098968 3.155351 0.568979
H 1.712290 2.817176 1.256324
C 0.248934 0.357400 0.631702
O -0.123154 0.528258 1.980134
H 2.808844 -2.782865 -1.464297
H -0.420408 0.932743 -0.023666
H -1.101591 0.435819 2.006067
H 5.604991 2.033607 -1.733060
H 6.085231 2.268888 -0.029903
H 6.192407 0.651343 -0.768257
O -2.763468 0.102130 1.707671
C -3.140861 0.766492 0.716643
O -2.689686 1.810183 0.240724
C -4.347207 0.127399 -0.014245
F -4.815171 0.881734 -1.011102
F -4.000714 -1.060004 -0.542720
F -5.365761 -0.104127 0.826546
36
BenPy7-Me_TS_prot-CF3COOH
C -2.891758 0.020680 1.195106
C -4.142358 0.101850 0.392501
C -5.056950 -0.958924 0.401014
C -6.247376 -0.877723 -0.313003
C -6.555777 0.267548 -1.042286
C -5.666395 1.338961 -1.037881

C -4.478773 1.262916 -0.318582
C -1.601088 0.168309 0.634082
C -0.441231 -0.197292 1.383770
C 0.809075 0.031640 0.895888
N 1.011553 0.590917 -0.323536
C 2.369508 0.886595 -0.793251
C -0.063451 0.926976 -1.081865
C -1.340542 0.733322 -0.651339
H -4.826110 -1.850736 0.970387
H -6.936866 -1.714237 -0.299885
H -7.484371 0.330149 -1.596999
H -5.905131 2.245506 -1.582047
H -3.814227 2.120002 -0.289755
H -0.544846 -0.660560 2.353457
H 1.702687 -0.226631 1.448486
H 0.159999 1.344555 -2.055066
H -2.145526 0.999414 -1.321068
H -2.864938 1.329551 1.850940
O -2.948711 -0.888207 2.274169
H -3.544392 -0.493127 2.930105
H 3.061712 0.191310 -0.324541
H 2.404384 0.762376 -1.873443
H 2.640439 1.909281 -0.531090
O -4.486559 1.084724 3.663085
C -3.923282 2.119333 3.363390
O -3.008868 2.305631 2.484552
C -4.294461 3.426065 4.089648
F -3.238135 3.910494 4.750763
F -4.685687 4.358510 3.216521
F -5.277720 3.239256 4.963758
36
BenPy7_Me_min2-CF3COOH
C -0.657238 -1.169114 -0.017837
C -0.195633 -2.607816 -0.155791
C 0.230796 -3.318002 0.966253
C 0.683944 -4.625973 0.831402
C 0.722395 -5.228291 -0.423977
C 0.300836 -4.519386 -1.544749
C -0.159164 -3.212437 -1.410421
C 0.559070 -0.259005 -0.052643
C 1.141712 0.209940 1.122701
C 2.216997 1.066305 1.049818
N 2.716220 1.444672 -0.140805
C 3.774611 2.470695 -0.181759
C 2.179444 0.994550 -1.290925
C 1.105559 0.136057 -1.272838
H 0.199748 -2.848205 1.942553

H 1.008712 -5.175738 1.707026
H 1.075861 -6.247388 -0.526756
H 0.321771 -4.985108 -2.522975
H -0.499084 -2.660751 -2.280767
H 0.742811 -0.057606 2.091305
H 2.698244 1.483347 1.924098
H 2.626326 1.361015 -2.205036
H 0.677447 -0.195288 -2.210533
H -1.276969 -0.912069 -0.883760
O -1.382912 -1.023263 1.182582
H -1.615342 -0.077001 1.273800
H 4.429417 2.331704 0.674395
H 4.334880 2.359165 -1.105608
H 3.301221 3.451181 -0.141092
O -1.584938 1.723364 1.351390
C -1.325312 2.173580 0.217709
O -1.704571 1.797556 -0.896444
C -0.382749 3.407838 0.204388
F 0.382140 3.491635 1.300266
F 0.447121 3.393882 -0.850508
F -1.092277 4.545262 0.129158
49
AZON3_TS_prot-HCOOH
C 4.688755 0.769288 0.081505
C 5.354073 1.474896 1.075784
C 4.755779 1.688319 2.326094
C 3.489740 1.192071 2.613075
C 2.813287 0.488917 1.617816
C 3.409443 0.285762 0.354762
H 6.345418 1.867702 0.884711
H 5.294662 2.248289 3.081776
H 3.032927 1.347562 3.584312
C 2.445508 -0.443777 -0.459633
C 2.483640 -0.878517 -1.758713
N 1.408788 -1.504714 -2.289265
C 0.272448 -1.698938 -1.568405
C 0.171711 -1.300946 -0.260251
C 1.276072 -0.671594 0.327767
H 3.334681 -0.745182 -2.415535
C 1.441687 -1.867827 -3.723272
H -0.536834 -2.188204 -2.093594
H -0.745880 -1.477433 0.284774
C 1.529919 -0.236994 1.671465
O 0.483719 0.156587 2.517583
H 5.149197 0.605507 -0.886553
H 1.962211 -1.398370 2.169827
H 0.674546 -0.262881 3.379429

C 1.278780 -0.644195 -4.619687
H 2.398109 -2.350404 -3.917715
H 0.644272 -2.589973 -3.891365
O 1.563267 -1.265057 4.598471
C 2.154807 -2.231235 4.102849
O 2.340226 -2.441270 2.856248
H 2.574339 -3.002371 4.768069
H 1.341404 -1.002933 -5.648590
C -0.053826 0.037251 -4.350082
H 2.111599 0.040847 -4.446850
N -0.394751 1.157354 -5.300598
H -0.876664 -0.677313 -4.422782
H -0.064292 0.486950 -3.354932
C -1.670619 1.778343 -4.829276
C 0.676621 2.197516 -5.305587
C -0.600646 0.643094 -6.687745
H 0.326181 3.038226 -5.901690
H 0.858892 2.508142 -4.277675
H 1.578236 1.780292 -5.747681
H -1.942890 2.570728 -5.523375
H -2.439987 1.008470 -4.808930
H -1.508474 2.184909 -3.832483
H -0.936362 1.473604 -7.306101
H 0.338171 0.253758 -7.072802
H -1.358564 -0.138024 -6.655194
49
AZON3_min2-HCOOH
C 1.715050 2.855093 -1.071735
C 2.818639 3.600017 -0.664119
C 3.852093 3.009284 0.066355
C 3.811913 1.655094 0.397959
C 2.717819 0.910822 -0.010589
C 1.675477 1.508231 -0.729898
H 2.874795 4.652948 -0.912598
H 4.696517 3.612563 0.377838
H 4.615370 1.190939 0.959692
C 0.646093 0.483467 -0.958346
C -0.584677 0.544532 -1.569705
N -1.360932 -0.561744 -1.592636
C -0.977138 -1.719876 -1.022420
C 0.252753 -1.835632 -0.399303
C 1.069281 -0.723295 -0.381374
H -0.989966 1.430471 -2.042097
C -2.714419 -0.455544 -2.196650
H -1.679935 -2.539117 -1.087634
H 0.543469 -2.776422 0.048832
C 2.451869 -0.568877 0.206869

O 2.504680 -1.009245 1.541283
H 0.908777 3.314237 -1.631974
H 3.148118 -1.154269 -0.411855
H 3.465913 -1.232350 1.711310
C -3.687713 0.262144 -1.268165
H -2.605326 0.094417 -3.129224
H -3.046179 -1.468400 -2.417845
O 5.040330 -1.500849 1.805845
C 5.346221 -2.458541 1.037318
O 4.577736 -3.095662 0.298016
H 6.417267 -2.746049 1.039628
H -4.626925 0.342047 -1.818224
C -3.871441 -0.518762 0.023574
H -3.320189 1.271706 -1.072670
N -4.924923 0.034410 0.948637
H -4.169066 -1.548183 -0.188003
H -2.949388 -0.530895 0.608481
C -4.924042 -0.805757 2.185624
C -4.620919 1.447212 1.326169
C -6.280420 -0.034452 0.325539
H -5.328558 1.747932 2.096834
H -3.601440 1.490223 1.706596
H -4.733567 2.083941 0.451900
H -5.696608 -0.430596 2.853573
H -5.133396 -1.835490 1.901507
H -3.944175 -0.728020 2.653241
H -7.007245 0.282417 1.071388
H -6.318099 0.632850 -0.531624
H -6.468662 -1.063393 0.023629
51
BenPy7_TS_prot-HCOOH
C 2.266548 -0.233371 0.546112
C 2.985836 1.018103 0.171484
C 3.374112 1.941138 1.149598
C 4.071510 3.093878 0.803686
C 4.401512 3.346697 -0.525174
C 4.037708 2.426833 -1.505155
C 3.347440 1.270064 -1.159254
C 0.959879 -0.517687 0.038812
C 0.191420 -1.562473 0.628825
C -1.008496 -1.933261 0.100805
N -1.520137 -1.324931 -0.999267
C -2.873191 -1.682933 -1.463308
C -0.835247 -0.305689 -1.572790
C 0.377333 0.104063 -1.099100
H 3.126692 1.748489 2.186170
H 4.357530 3.798342 1.576534

H 4.945799 4.244510 -0.792995
H 4.306668 2.600677 -2.540750
H 3.102475 0.542446 -1.926315
H 0.561434 -2.079671 1.502076
H -1.609900 -2.728981 0.522726
H -1.311927 0.165322 -2.422397
H 0.861782 0.929648 -1.600025
H 2.928597 -1.220582 -0.209914
O 2.455688 -0.616568 1.895497
H 2.978756 -1.439872 1.880478
H -2.997057 -1.254488 -2.457193
H -2.923763 -2.768018 -1.543063
C -3.952379 -1.178660 -0.508919
O 3.740514 -2.979347 1.282296
C 3.934787 -3.002747 0.066700
O 3.567866 -2.100979 -0.771693
H 4.465324 -3.852229 -0.387586
H -4.911762 -1.485280 -0.929677
H -3.833568 -1.669216 0.459704
C -3.867447 0.333075 -0.369165
N -4.961940 0.953035 0.462761
H -2.934311 0.626211 0.117237
H -3.922621 0.816146 -1.347129
C -4.679831 2.417154 0.561715
C -6.297849 0.765690 -0.176854
C -4.980899 0.372574 1.837817
H -5.688707 0.944773 2.434521
H -5.298282 -0.665793 1.783464
H -3.978633 0.449192 2.256822
H -7.032223 1.310865 0.413353
H -6.253345 1.162591 -1.189662
H -6.547243 -0.292212 -0.188010
H -5.478008 2.881930 1.136967
H -3.722156 2.550020 1.061788
H -4.646019 2.828931 -0.445379
51
BenPy7_min2-HCOOH
C 2.802484 0.241804 -0.055683
C 2.462854 1.722190 -0.054442
C 2.064954 2.346678 1.127730
C 1.719946 3.693919 1.123096
C 1.763208 4.423866 -0.062768
C 2.157887 3.802399 -1.243511
C 2.509005 2.455047 -1.238602
C 1.506168 -0.542634 -0.172711
C 0.930505 -1.161711 0.935994
C -0.264287 -1.829309 0.793811

N -0.878189 -1.898430 -0.402712
C -2.207130 -2.552258 -0.488498
C -0.340116 -1.312186 -1.490631
C 0.852519 -0.634997 -1.401339
H 2.033403 1.776415 2.049138
H 1.416473 4.175423 2.045354
H 1.494724 5.473647 -0.064580
H 2.201292 4.367170 -2.167324
H 2.828073 1.970710 -2.155652
H 1.410204 -1.134979 1.904050
H -0.761700 -2.327799 1.615871
H -0.895048 -1.412124 -2.413652
H 1.269269 -0.180858 -2.291628
H 3.390572 0.019227 -0.952874
O 3.508691 -0.085160 1.114168
H 3.647706 -1.074354 1.117998
H -2.426436 -2.692555 -1.545429
H -2.122276 -3.526609 -0.010952
C -3.281242 -1.717557 0.200947
O 3.717946 -2.700703 1.193814
C 3.597772 -3.249791 0.062962
O 3.597584 -2.689460 -1.047171
H 3.483683 -4.353433 0.079122
H -4.212112 -2.280063 0.111662
H -3.039860 -1.628084 1.262047
C -3.400744 -0.351764 -0.456236
N -4.446612 0.549376 0.150009
H -2.464295 0.205057 -0.378555
H -3.663859 -0.454381 -1.511061
C -4.410364 1.845038 -0.595823
C -5.812342 -0.040178 0.017260
C -4.155854 0.818933 1.590587
H -4.854756 1.578406 1.935994
H -4.292213 -0.094775 2.163646
H -3.130974 1.177807 1.673277
H -6.530672 0.697582 0.370102
H -5.986224 -0.269187 -1.032942
H -5.879409 -0.939117 0.624702
H -5.163231 2.505264 -0.170023
H -3.417548 2.277620 -0.484775
H -4.626290 1.645025 -1.643931
50

AZON3_TS_prot-H2CO3

C 4.734598 0.725690 0.126019
C 5.402735 1.424425 1.122916
C 4.792032 1.664510 2.362630
C 3.511220 1.200624 2.636834

C 2.831426 0.504340 1.638660
C 3.439994 0.276085 0.385196
H 6.405742 1.791875 0.942592
H 5.333402 2.219067 3.120502
H 3.045361 1.375349 3.600406
C 2.470407 -0.435721 -0.437554
C 2.513670 -0.878334 -1.734062
N 1.430196 -1.481350 -2.274246
C 0.281878 -1.647479 -1.564586
C 0.173918 -1.238585 -0.261019
C 1.285175 -0.629212 0.336114
H 3.375150 -0.767772 -2.381645
C 1.467022 -1.853643 -3.705529
H -0.531547 -2.123272 -2.096006
H -0.753675 -1.391590 0.273989
C 1.531110 -0.189930 1.677971
O 0.479678 0.225608 2.507576
H 5.204252 0.541809 -0.833912
H 1.937555 -1.374051 2.170836
H 0.651608 -0.184807 3.374211
C 1.289474 -0.639442 -4.612694
H 2.428721 -2.326629 -3.896690
H 0.678352 -2.586563 -3.867839
O 1.573983 -1.220945 4.583672
C 2.107712 -2.208370 4.069783
O 2.276107 -2.428129 2.824473
O 2.587811 -3.201876 4.851313
H 1.381231 -1.001549 -5.638368
C -0.067035 0.005438 -4.374047
H 2.100615 0.067921 -4.426924
N -0.410186 1.130125 -5.318073
H -0.868005 -0.729236 -4.481661
H -0.120136 0.441909 -3.374599
C -1.721807 1.700474 -4.881804
C 0.625805 2.204492 -5.267202
C -0.550115 0.634211 -6.719644
H 0.269320 3.044372 -5.860832
H 0.761487 2.501940 -4.228234
H 1.555471 1.824575 -5.684165
H -1.994424 2.498256 -5.569616
H -2.465928 0.906226 -4.905076
H -1.609643 2.091126 -3.871841
H -0.889092 1.464785 -7.336347
H 0.413817 0.280389 -7.075961
H -1.284001 -0.170092 -6.727420
H 2.413523 -2.962117 5.771791
50

AZON3_min2-H2CO3

C 1.283868 2.995945 -1.111591
C 2.346043 3.807909 -0.722790
C 3.414126 3.287917 0.011587
C 3.450993 1.939441 0.366317
C 2.398018 1.128740 -0.023097
C 1.321125 1.655188 -0.746519
H 2.342677 4.857811 -0.989716
H 4.225462 3.942373 0.307284
H 4.282820 1.530917 0.929839
C 0.350696 0.570892 -0.955578
C -0.879650 0.553351 -1.569425
N -1.591492 -0.595555 -1.579643
C -1.144104 -1.721570 -0.992107
C 0.089297 -1.758098 -0.365081
C 0.841519 -0.601299 -0.361912
H -1.330869 1.408726 -2.056708
C -2.942541 -0.573213 -2.197345
H -1.797667 -2.581445 -1.048944
H 0.432643 -2.675002 0.095318
C 2.215783 -0.359917 0.218128
O 2.297423 -0.775737 1.559781
H 0.450823 3.398927 -1.675893
H 2.940367 -0.915240 -0.394701
H 3.261293 -0.953531 1.724944
C -3.956349 0.129457 -1.301292
H -2.849354 -0.048006 -3.146116
H -3.225743 -1.606306 -2.389528
O 4.875850 -1.171372 1.749839
C 5.152250 -2.141320 0.994453
O 4.381635 -2.852342 0.340550
O 6.491734 -2.454025 0.880156
H -4.886169 0.172429 -1.870724
C -4.144296 -0.637540 -0.001994
H -3.622122 1.152505 -1.116637
N -5.214983 -0.087119 0.904953
H -4.427256 -1.672477 -0.206473
H -3.229717 -0.632589 0.594502
C -5.241994 -0.935290 2.136142
C -4.914891 1.322252 1.298148
C -6.557970 -0.147082 0.254322
H -5.642803 1.622131 2.050188
H -3.906483 1.358658 1.707587
H -5.000304 1.964254 0.424715
H -6.019761 -0.555312 2.795298
H -5.459337 -1.960628 1.842328
H -4.268361 -0.872405 2.618728

H -7.298984 0.159940 0.990248
H -6.578673 0.532023 -0.594157
H -6.740654 -1.171685 -0.065427
H 6.981133 -1.832034 1.432429
52
BenPy7_TS_prot-H2CO3
C 1.990307 -0.098016 0.634641
C 2.602937 1.184612 0.176310
C 2.944169 2.178060 1.101333
C 3.549333 3.358955 0.685075
C 3.834176 3.568351 -0.661739
C 3.519639 2.577738 -1.588214
C 2.921687 1.392800 -1.172355
C 0.690338 -0.498646 0.213849
C -0.005509 -1.527857 0.912858
C -1.198114 -1.999783 0.454947
N -1.773943 -1.512143 -0.672932
C -3.099734 -2.007098 -1.084195
C -1.163838 -0.504358 -1.345960
C 0.033502 0.008772 -0.943037
H 2.731720 2.018015 2.151140
H 3.799185 4.119003 1.416657
H 4.307620 4.488058 -0.984199
H 3.755481 2.719527 -2.636643
H 2.714737 0.612348 -1.897061
H 0.412153 -1.952217 1.813364
H -1.743089 -2.786791 0.960542
H -1.688193 -0.133323 -2.217701
H 0.457334 0.817578 -1.520611
H 2.695509 -1.061785 -0.106613
O 2.272634 -0.423173 1.981476
H 3.198339 -0.719900 1.983735
H -3.222217 -1.768551 -2.140339
H -3.094702 -3.090273 -0.973357
C -4.225063 -1.405540 -0.246858
O 4.787026 -1.271746 1.087755
C 4.566937 -1.839241 0.019975
O 3.458527 -1.851024 -0.628563
O 5.532978 -2.544917 -0.602025
H -5.145688 -1.907699 -0.549321
H -4.042653 -1.631170 0.806238
C -4.318470 0.092001 -0.488255
N -5.431484 0.787531 0.255070
H -3.402626 0.599385 -0.178653
H -4.493339 0.296089 -1.546480
C -5.373761 2.238636 -0.100221
C -6.765433 0.250340 -0.147018

C -5.260749 0.648092 1.732277
H -6.011143 1.270516 2.216406
H -5.406224 -0.391461 2.015047
H -4.259119 0.984283 1.996133
H -7.528737 0.863953 0.328149
H -6.847771 0.310121 -1.231033
H -6.856655 -0.778664 0.191241
H -6.173603 2.752793 0.428658
H -4.404221 2.630269 0.201851
H -5.507362 2.334778 -1.176326
H 6.329812 -2.502637 -0.055416
52
BenPy7_min2-H2CO3
C 2.076940 -1.240404 -0.185215
C 2.247209 0.275514 -0.168610
C 2.148658 0.977324 1.032273
C 2.306715 2.359095 1.047035
C 2.558875 3.047823 -0.137597
C 2.658347 2.348810 -1.336682
C 2.507431 0.965142 -1.350715
C 0.599665 -1.560654 -0.243387
C -0.074673 -2.074521 0.862124
C -1.427932 -2.313799 0.778274
N -2.103392 -2.067160 -0.359003
C -3.573089 -2.268150 -0.374156
C -1.473524 -1.572743 -1.443896
C -0.123912 -1.315026 -1.411077
H 1.964505 0.437583 1.954542
H 2.235622 2.899055 1.983980
H 2.686204 4.123808 -0.123606
H 2.869617 2.877224 -2.258819
H 2.611274 0.413297 -2.279244
H 0.445965 -2.292495 1.783026
H -2.007080 -2.709672 1.602656
H -2.084058 -1.400997 -2.320424
H 0.358439 -0.921533 -2.297071
H 2.535993 -1.621762 -1.105750
O 2.627059 -1.865452 0.949707
H 3.531815 -1.498022 1.087060
H -3.878753 -2.344926 -1.416166
H -3.771560 -3.214910 0.124438
C -4.293821 -1.132502 0.343310
O 4.969790 -0.620838 1.289747
C 5.254293 -0.166620 0.151671
O 5.277613 -0.762331 -0.932157
O 5.576771 1.175313 0.095930
H -5.352363 -1.397182 0.348075

H -3.948586 -1.089571 1.378429
C -4.059039 0.190547 -0.369799
N -4.869804 1.343797 0.165496
H -3.015944 0.502192 -0.282178
H -4.315722 0.110320 -1.428270
C -4.440452 2.576012 -0.565313
C -6.329249 1.133178 -0.073934
C -4.626193 1.540321 1.626202
H -5.121124 2.462208 1.926484
H -5.044423 0.700563 2.175710
H -3.551894 1.613093 1.789560
H -6.853443 2.029122 0.253573
H -6.482385 0.970834 -1.139655
H -6.671125 0.275819 0.499732
H -5.041857 3.410539 -0.210056
H -3.386357 2.749871 -0.356601
H -4.599440 2.416835 -1.630324
H 5.471701 1.532628 0.986612

9 References

1. Y. Zhao, D.G. Truhlar, The M06 suite of density functionals for main group thermochemistry, thermochemical kinetics, noncovalent interactions, excited states, and transition elements: two new functionals and systematic testing of four M06-class functionals and 12 other functionals, *Theor Chem Account* 120 (2008) 215–241. https://doi.org/10.1007/s00214-007-0310-x
2. Gaussian 16, Revision C.01, M. J. Frisch, G. W. Trucks, H. B. Schlegel, G. E. Scuseria, M. A. Robb, J. R. Cheeseman, G. Scalmani, V. Barone, G. A. Petersson, H. Nakatsuji, X. Li, M. Caricato, A. V. Marenich, J. Bloino, B. G. Janesko, R. Gomperts, B. Mennucci, H. P. Hratchian, J. V. Ortiz, A. F. Izmaylov, J. L. Sonnenberg, D. Williams-Young, F. Ding, F. Lipparini, F. Egidi, J. Goings, B. Peng, A. Petrone, T. Henderson, D. Ranasinghe, V. G. Zakrzewski, J. Gao, N. Rega, G. Zheng, W. Liang, M. Hada, M. Ehara, K. Toyota, R. Fukuda, J. Hasegawa, M. Ishida, T. Nakajima, Y. Honda, O. Kitao, H. Nakai, T. Vreven, K. Throssell, J. A. Montgomery, Jr., J. E. Peralta, F. Ogliaro, M. J. Bearpark, J. J. Heyd, E. N. Brothers, K. N. Kudin, V. N. Staroverov, T. A. Keith, R. Kobayashi, J. Normand, K. Raghavachari, A. P. Rendell, J. C. Burant, S. S. Iyengar, J. Tomasi, M. Cossi, J. M. Millam, M. Klene, C. Adamo, R. Cammi, J. W. Ochterski, R. L. Martin, K. Morokuma, O. Farkas, J. B. Foresman, and D. J. Fox, Gaussian, Inc., Wallingford CT, 2016.
3. a) A. D. McLean, G. S. Chandler, Contracted Gaussian-basis sets for molecular calculations. 1. 2nd row atoms, Z=11-18, J. Chem. Phys. 72 (1980) 5639-5648. https://doi.org/10.1063/1.438980. b) K. Raghavachari, J. S. Binkley, R. Seeger, J. A. Pople, Self-Consistent Molecular Orbital Methods. 20. Basis set for correlated wave-functions, J. Chem. Phys. 72 (1980) 650-654. https://doi.org/10.1063/1.438955.
4. A. V. Marenich, C. J. Cramer, D. G. Truhlar, Universal Solvation Model Based on Solute Electron Density and on a Continuum Model of the Solvent Defined by the Bulk Dielectric Constant and Atomic Surface Tensions, *J. Phys. Chem. B*, 113 (2009) 6378–6396. https://doi.org/10.1021/jp810292n.
5 P. Pracht, F. Bohle, S. Grimme, Automated exploration of the low-energy chemical space with fast quantum chemical methods, Phys. Chem. Chem. Phys. 22 (2020) 7169–7192. https://doi.org/10.1039/c9cp06869d.
6. P. Pracht, S. Grimme, C. Bannwarth, F. Bohle, S. Ehlert, G. Feldmann, J. Gorges, M. Müller, T. Neudecker, C. Plett, S. Spicher, P. Steinbach, P. A. Wesołowski, F. Zeller, CREST—A program for the exploration of low-energy molecular chemical space Open Access, *J. Chem. Phys.* 160 (2024) 114110. https://doi.org/10.1063/5.0197592.
7. A. A. Nechaev, G. Gonzalez, P. Verma, V. A. Peshkov, A. Bannykh, A. Hashemi, J. Hannonen, A. Hamza, I. Pápai, K. Laasonen, P. Peljo, P. M. Pihko, Exploration of Vitamin B6-Based Redox-Active Pyridinium Salts Towards the Application in Aqueous Organic Flow Batteries, Chem. Eur. J. 30 (2024) e202400828. https://doi.org/10.1002/chem.202400828
8 . A. Mehranfar, J. Hannonen, A. Tuna, M. Jafarishiadeh, A. Kiesilä, P. Pihko, P. Peljo, K. Laasonen, Computational Evaluation of Redox Potentials of Metal Complexes for Aqueous Flow Batteries, ChemPhysChem 26 (2025) e202500046. https://doi.org/10.1002/cphc.202500046
9. R. P. Bell, W. B. T. Miller, Dissociation constants of formic acid and formic acid-d, Trans. Faraday Soc. 59 (1963) 1147-1148. https://doi.org/10.1039/TF9635901147.
10. M. Busch, E. Ahlberg, K. Laasonen, How to Predict the pKa of Any Compound in Any Solvent, ACS Omega 7 (2022) 17369–17383. https://doi.org/10.1021/acsomega.2c01393.

11. J. Rossmeisl, A. Logadottir, J. Nørskov, Electrolysis of water on (oxidized) metal surfaces, Chem. Phys. 319 (2005) 178–184. https://doi.org/10.1016/j.chemphys.2005.05.038.
