# Supplementary material for: Rationalizing (in)stability of pyridinium ketones in aqueous organic flow batteries
Source: Chem Sci. 2026 Sep 15. Online ahead of print. doi: 10.1039/d6sc04767j (PMC13629303; doi:10.1039/d6sc04767j)

1 Copies of NMR spectra

1.1.1 4-Benzoyl-1-(3-methoxy-3-oxopropyl)pyridin-1-ium bromide (**BenPy1**), ^{1}H NMR ($CDCl_3$, 300 MHz) spectrum

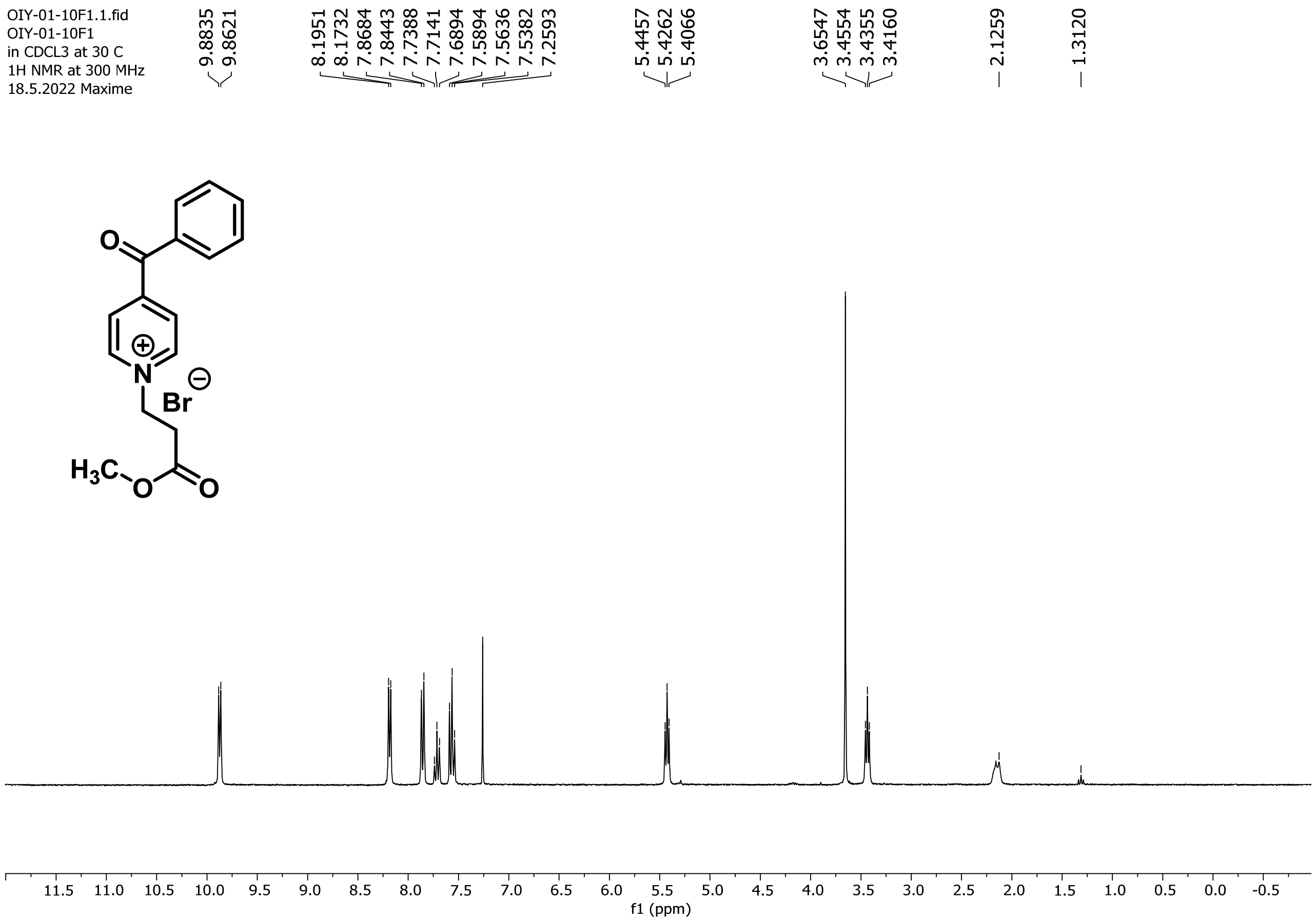

1.1.2 4-Benzoyl-1-(3-methoxy-3-oxopropyl)pyridin-1-ium bromide (**BenPy1**), ^{13}C NMR ($CDCl_3$, 75 MHz) spectrum

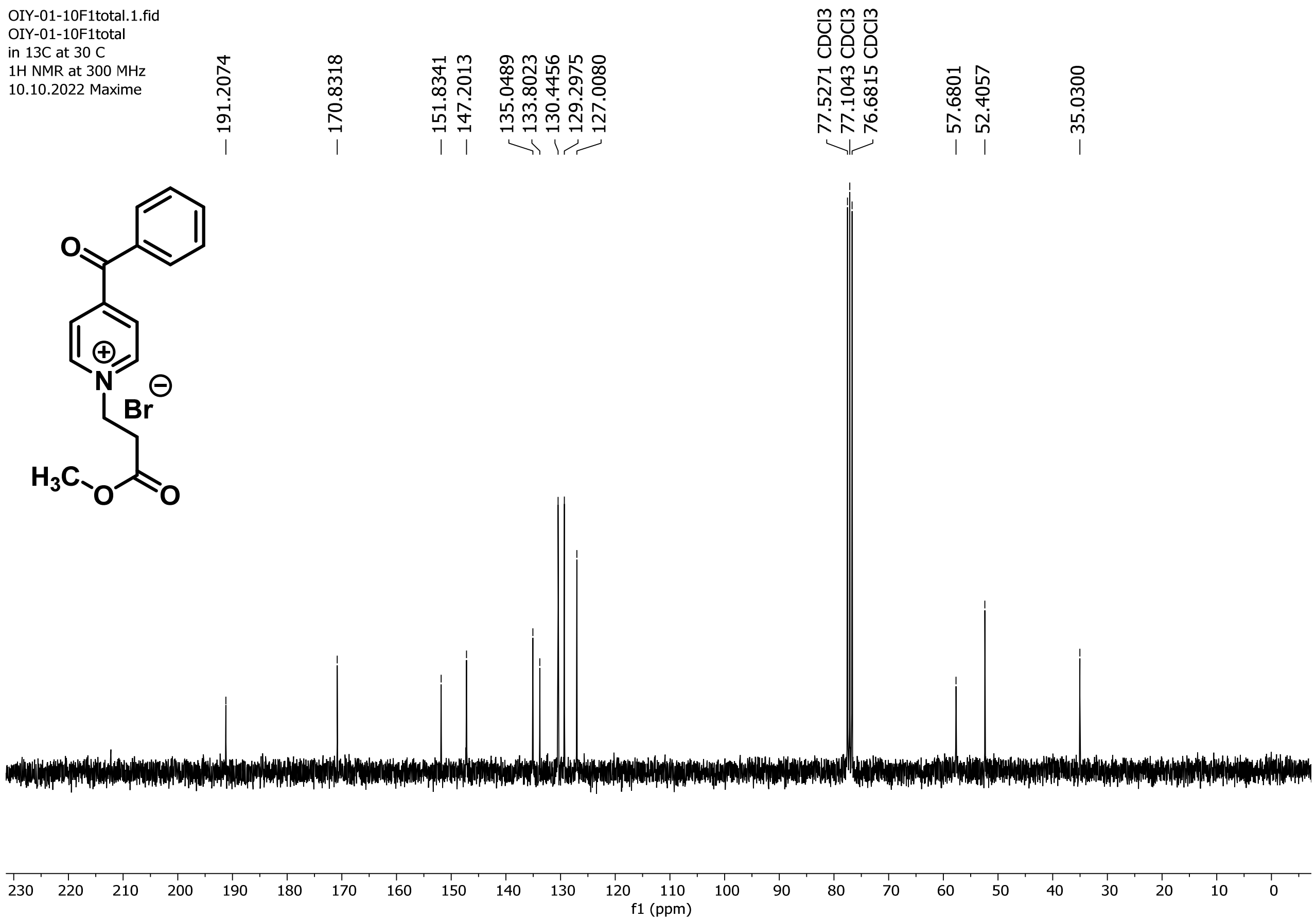

1.1.3 4-benzoyl-1-(carboxymethyl)pyridin-1-ium bromide (**BenPy2**), ^{1}H NMR (D_2O, 300 MHz) spectrum

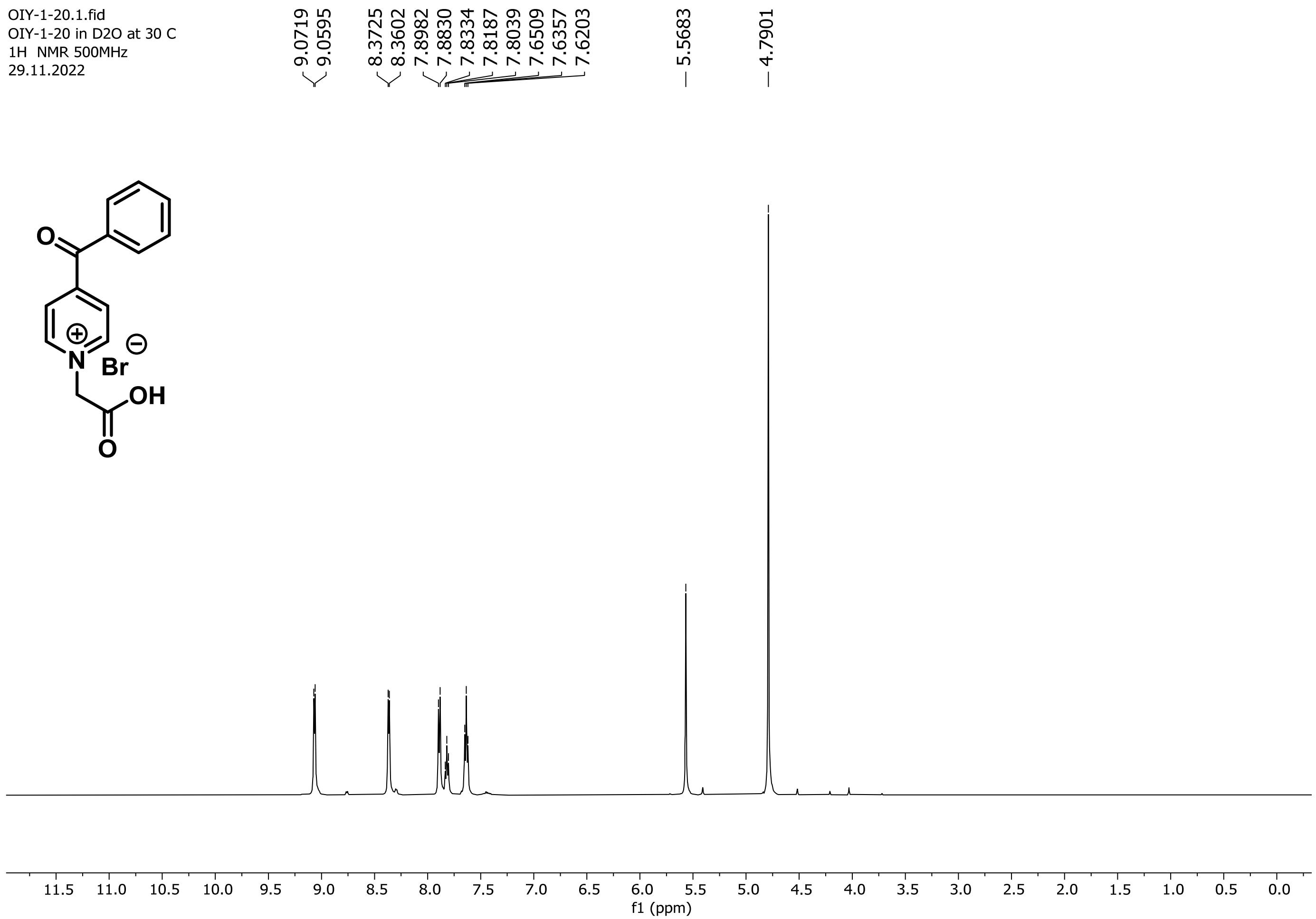

1.1.4 4-benzoyl-1-(carboxymethyl)pyridin-1-ium bromide (**BenPy2**), ^{13}C NMR (D_2O, 75 MHz) spectrum

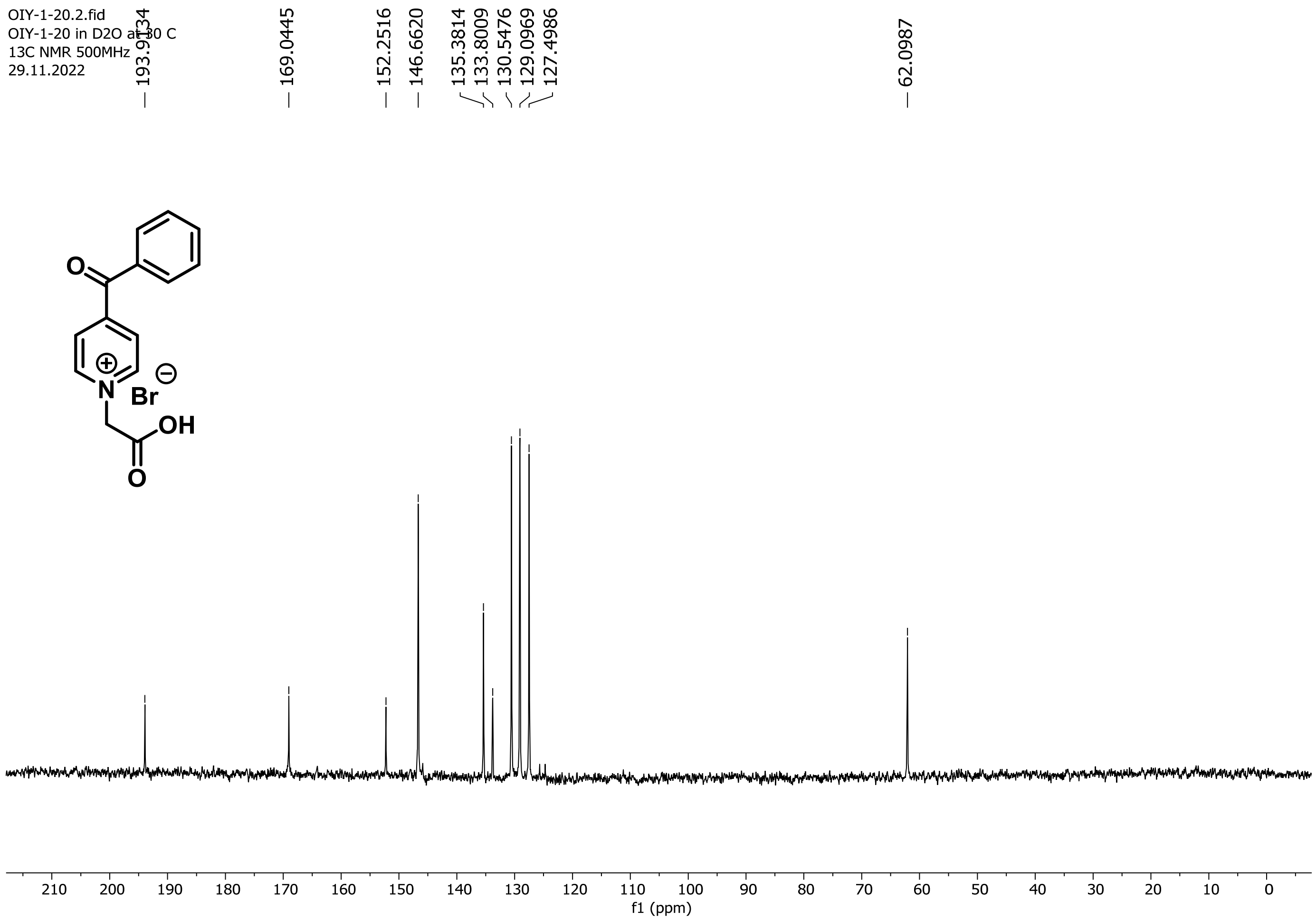

1.1.5 4-benzoyl-1-(3-phosphonopropyl)pyridin-1-ium bromide (**BenPy3**), ^{1}H NMR (DMSO, 300 MHz) spectrum

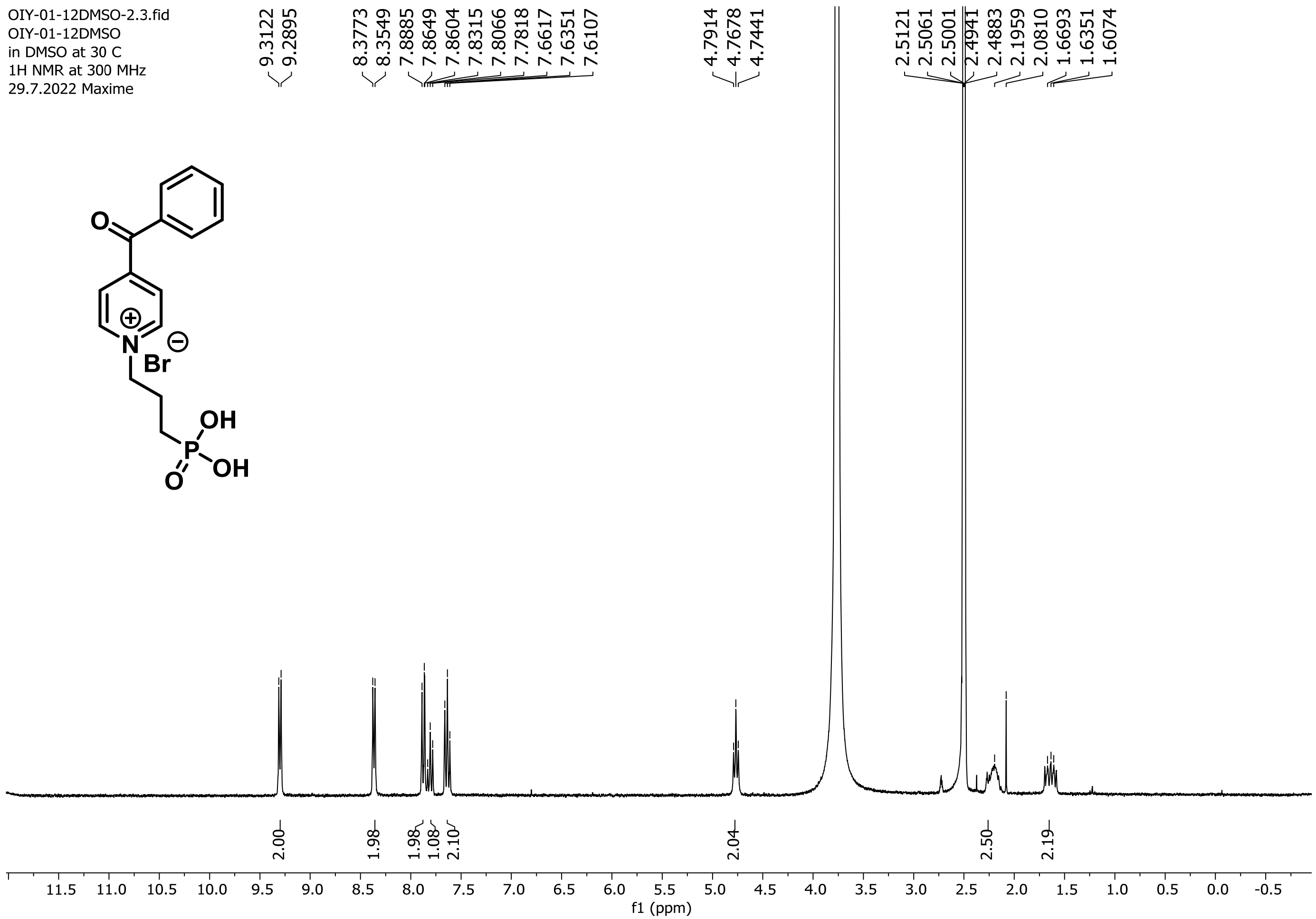

1.1.6 4-benzoyl-1-(3-phosphonopropyl)pyridin-1-ium bromide (**BenPy3**), ^{13}C NMR (DMSO, 125 MHz) spectrum

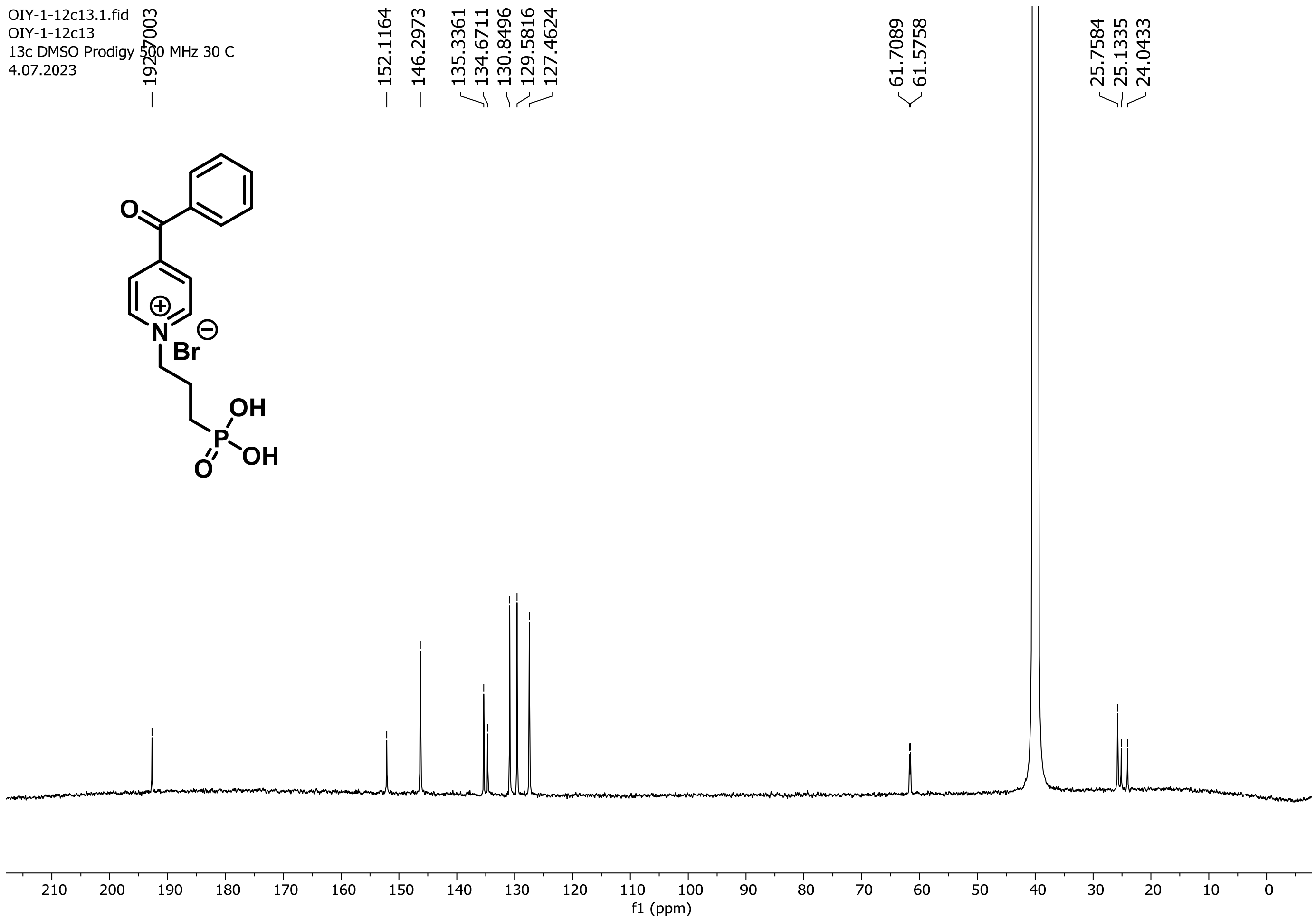

1.1.7 4-benzoyl-1-(3-phosphonopropyl)pyridin-1-ium bromide (**BenPy3**), ^{31}P NMR (DMSO, 122 MHz) spectrum

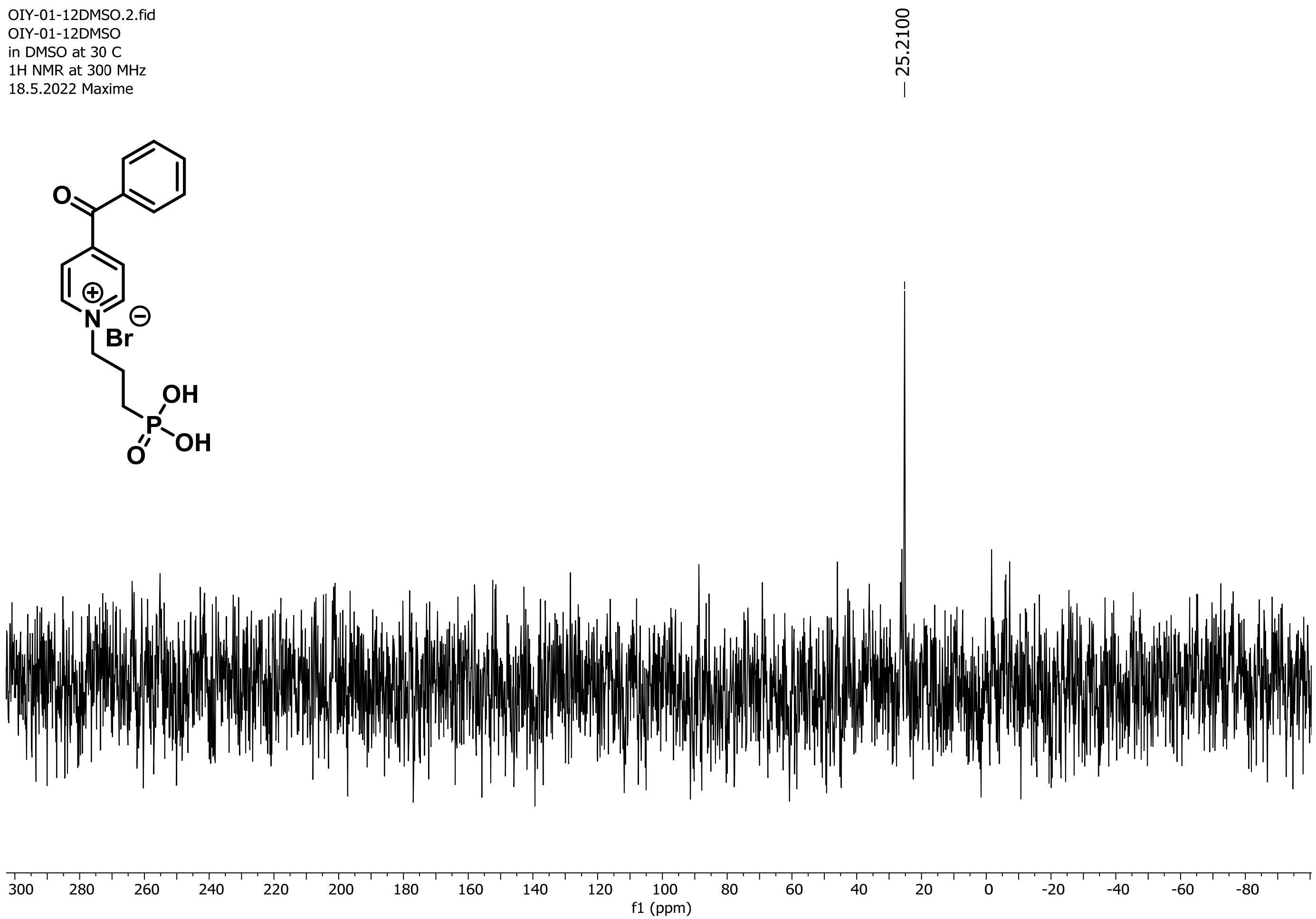

1.1.8 4-benzoyl-1-(2-phosphonoethyl)pyridin-1-ium bromide (**BenPy4**), ^{1}H NMR (D_2O, 500 MHz) spectrum

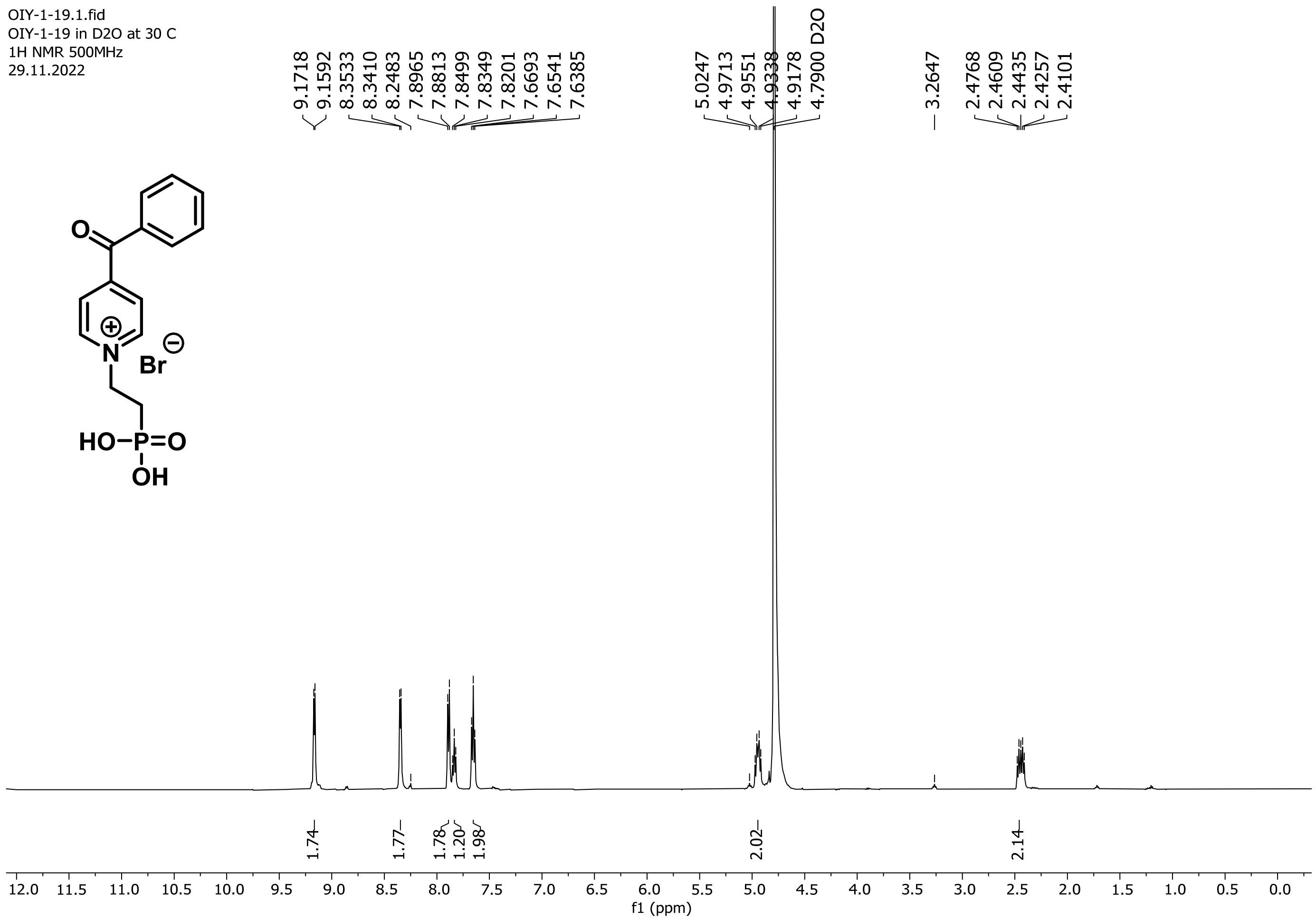

1.1.9 4-benzoyl-1-(2-phosphonoethyl)pyridin-1-ium bromide (**BenPy4**), ^{13}C NMR (D_2O, 125 MHz) spectrum

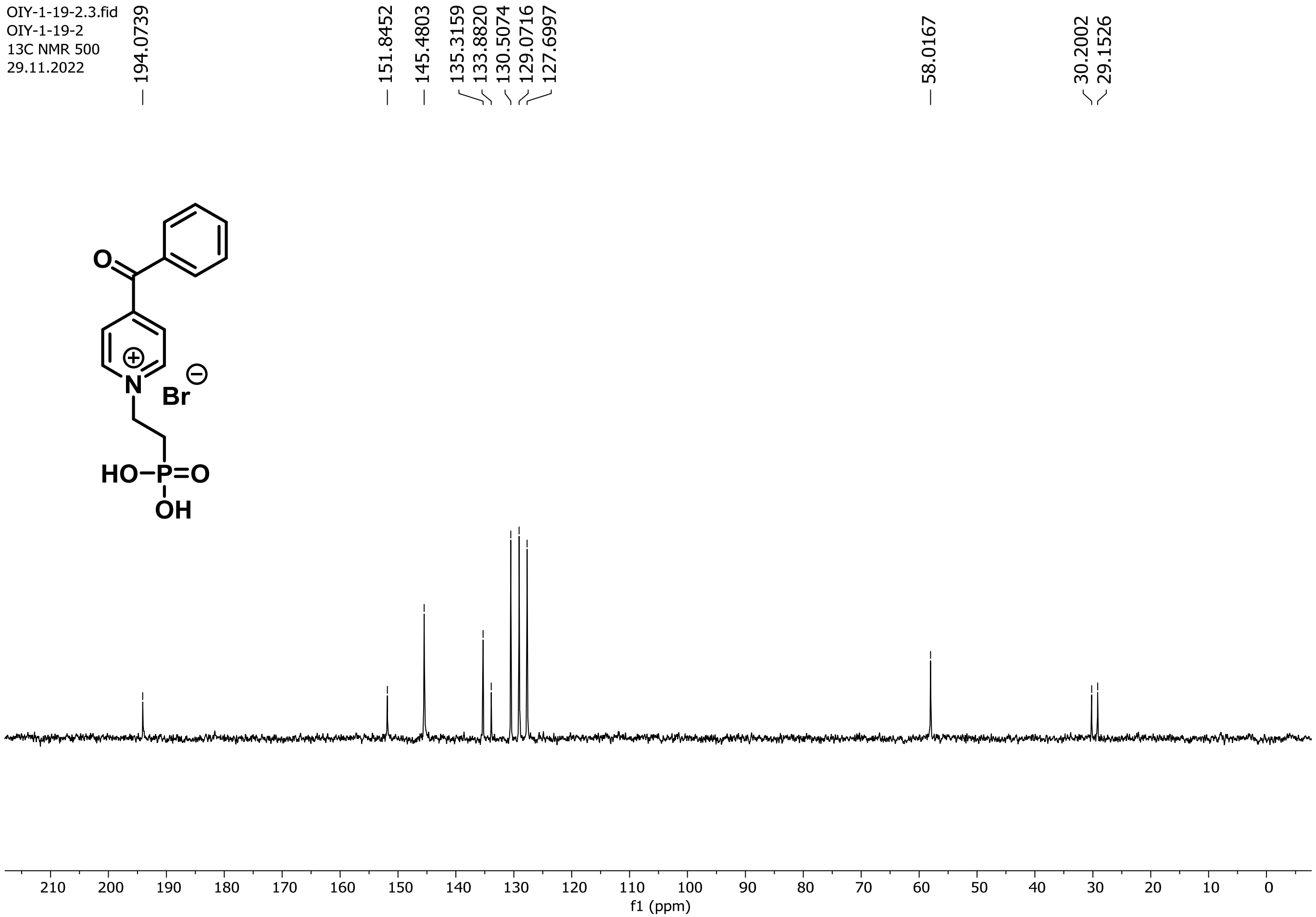

1.1.10 4-benzoyl-1-(2-phosphonoethyl)pyridin-1-ium bromide (**BenPy4**), ^{31}P NMR (D_2O, 122 MHz) spectrum

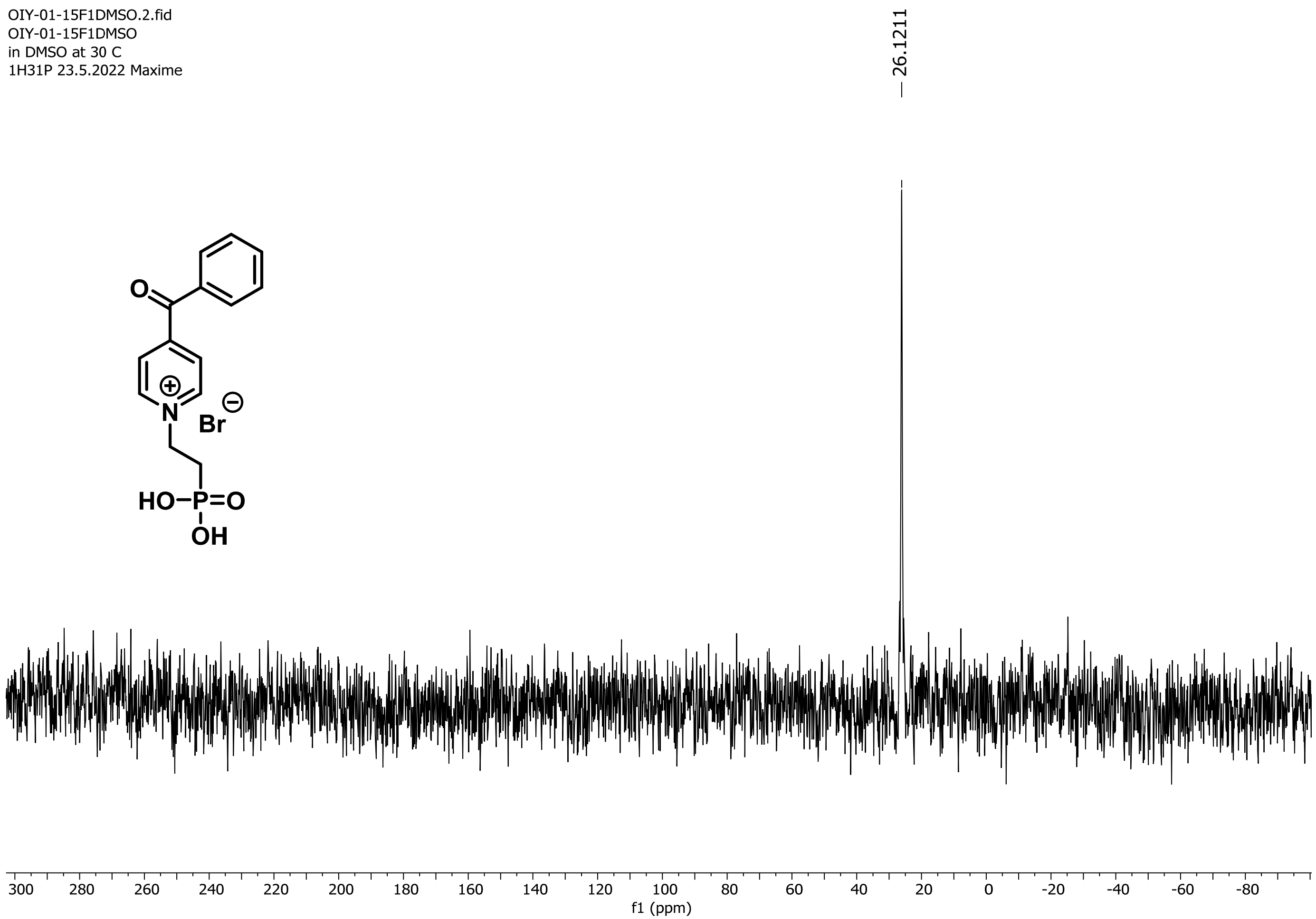

1.1.11 2-(4-benzoylpyridin-1-ium-1-yl)ethane-1-sulfonate (**BenPy5**), ^{1}H NMR (D_2O, 300 MHz) spectrum

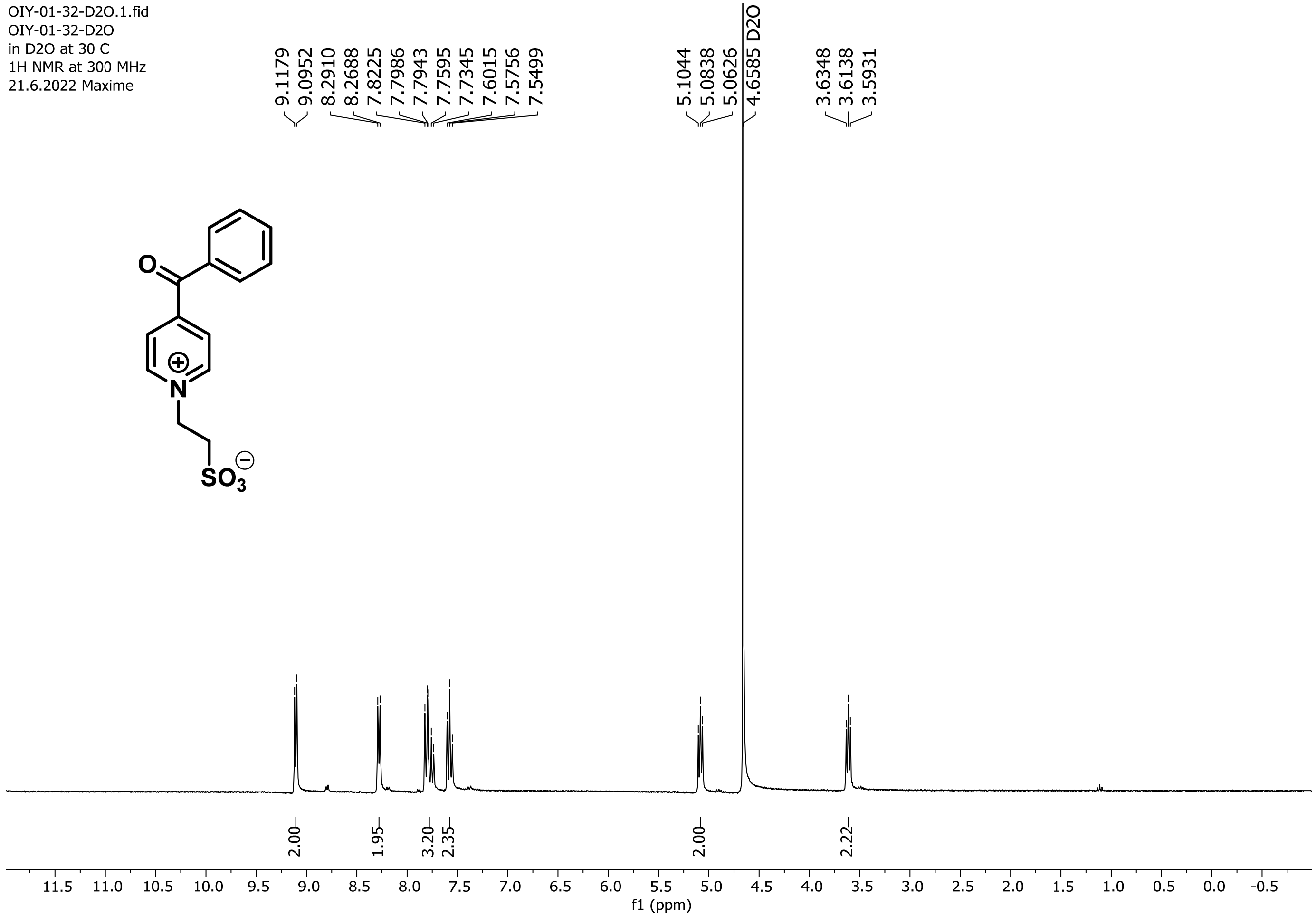

1.1.12 2-(4-benzoylpyridin-1-ium-1-yl)ethane-1-sulfonate (**BenPy5**), ^{13}C NMR (D_2O, 125 MHz) spectrum

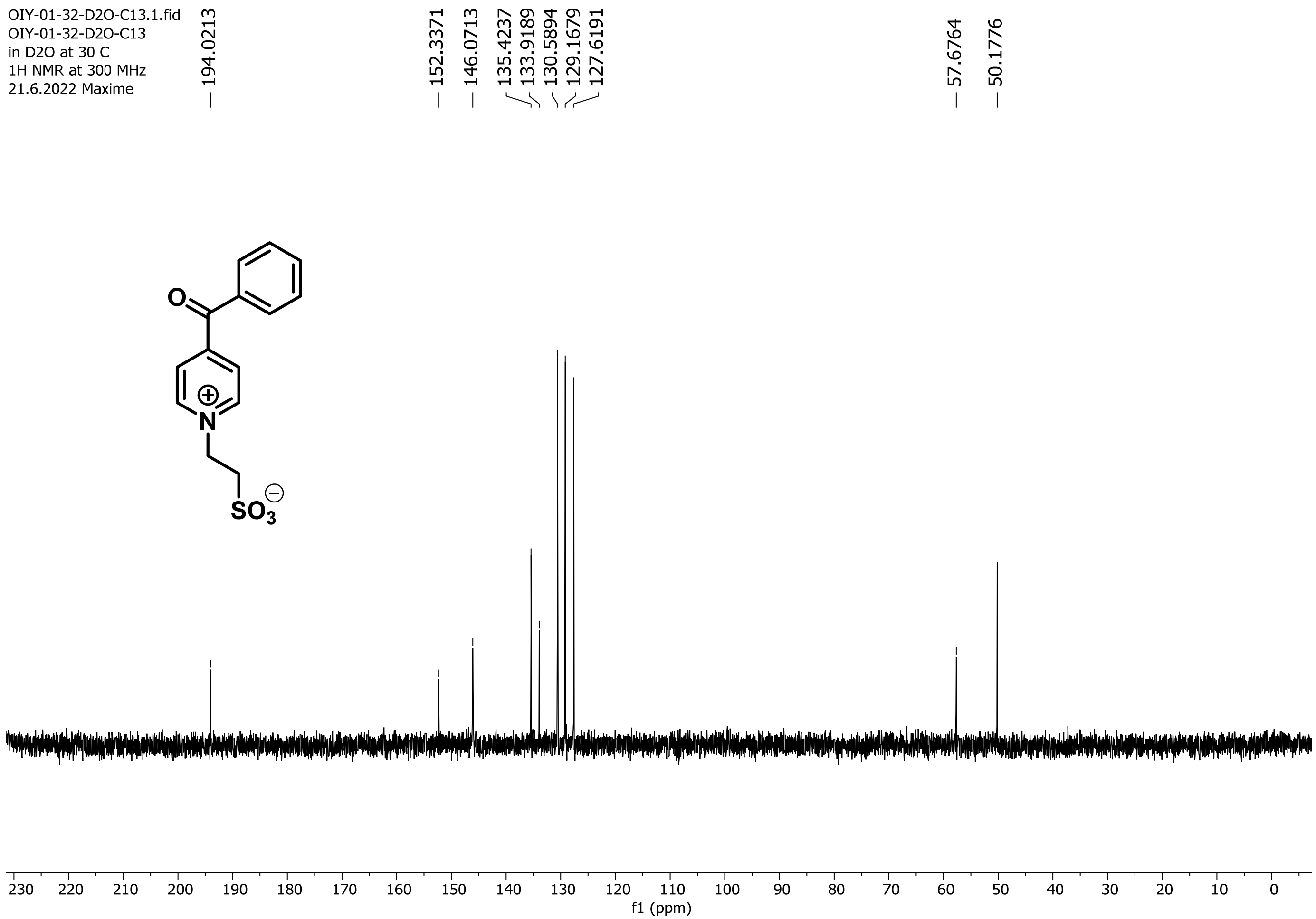

1.1.13 4-benzoyl-1-(2-hydroxyethyl)pyridin-1-ium bromide (**BenPy6**), ^{1}H NMR (D_2O, 300 MHz) spectrum

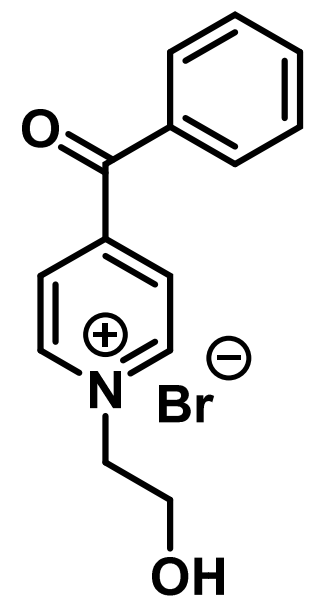

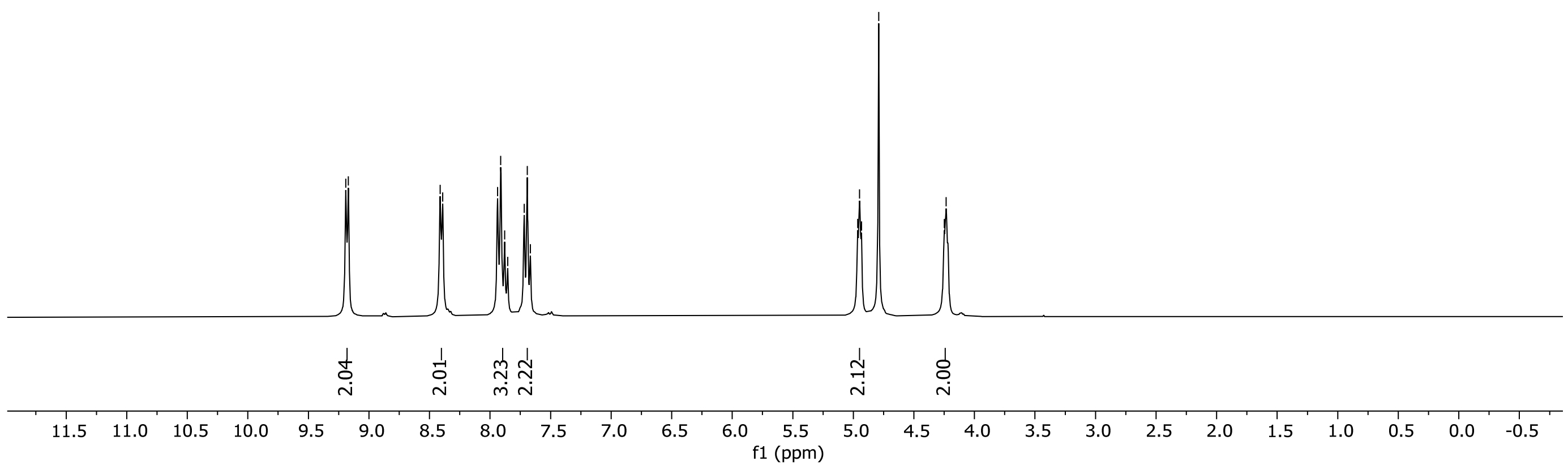

1.1.14 4-benzoyl-1-(2-hydroxyethyl)pyridin-1-ium bromide (**BenPy6**), ^{13}C NMR (D_2O, 75 MHz) spectrum

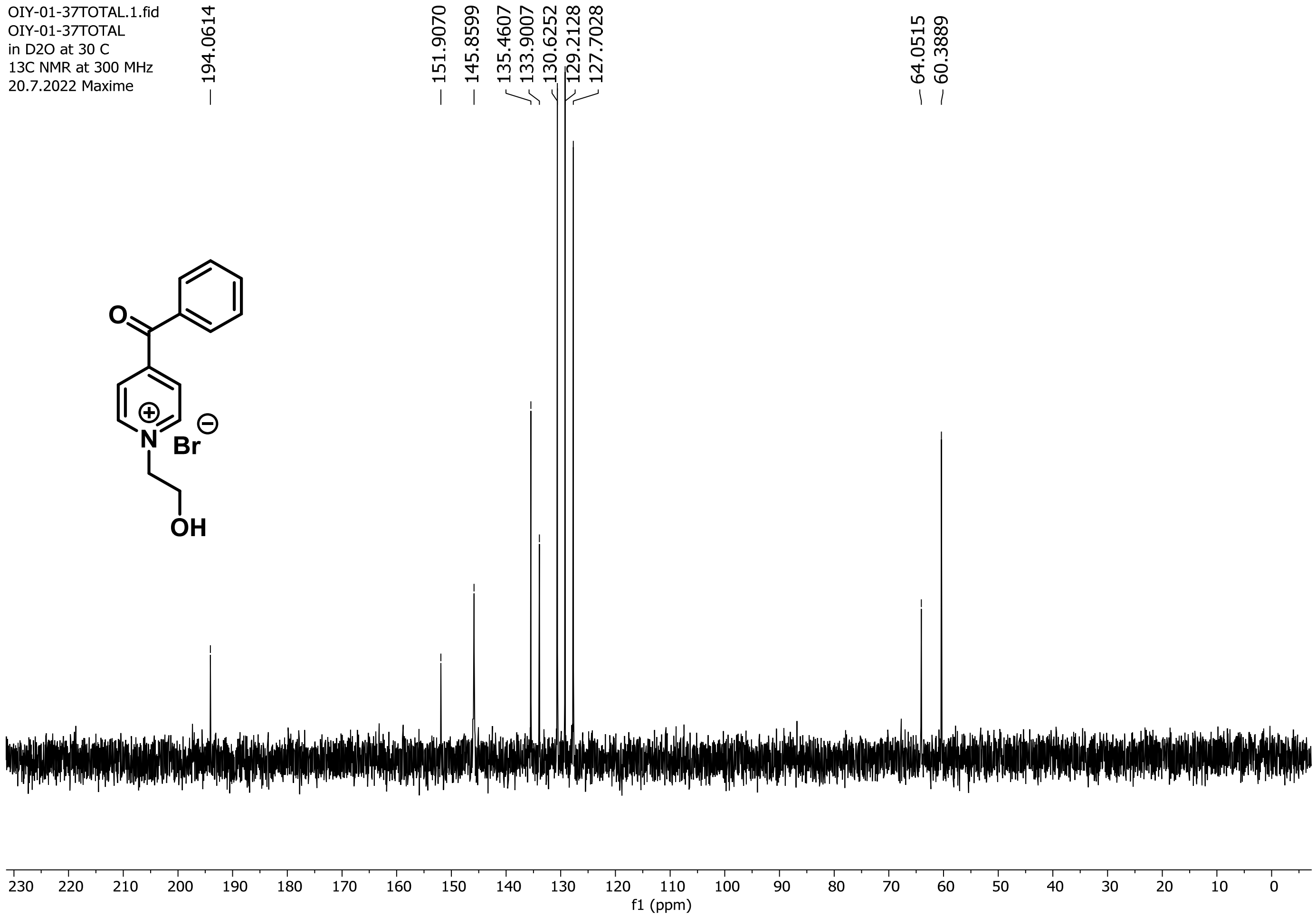

1.1.15 4-benzoyl-1-(3-(trimethylammonio)propyl)pyridin-1-ium bromide (**BenPy7**), ^{1}H NMR (D_2O, 300 MHz) spectrum

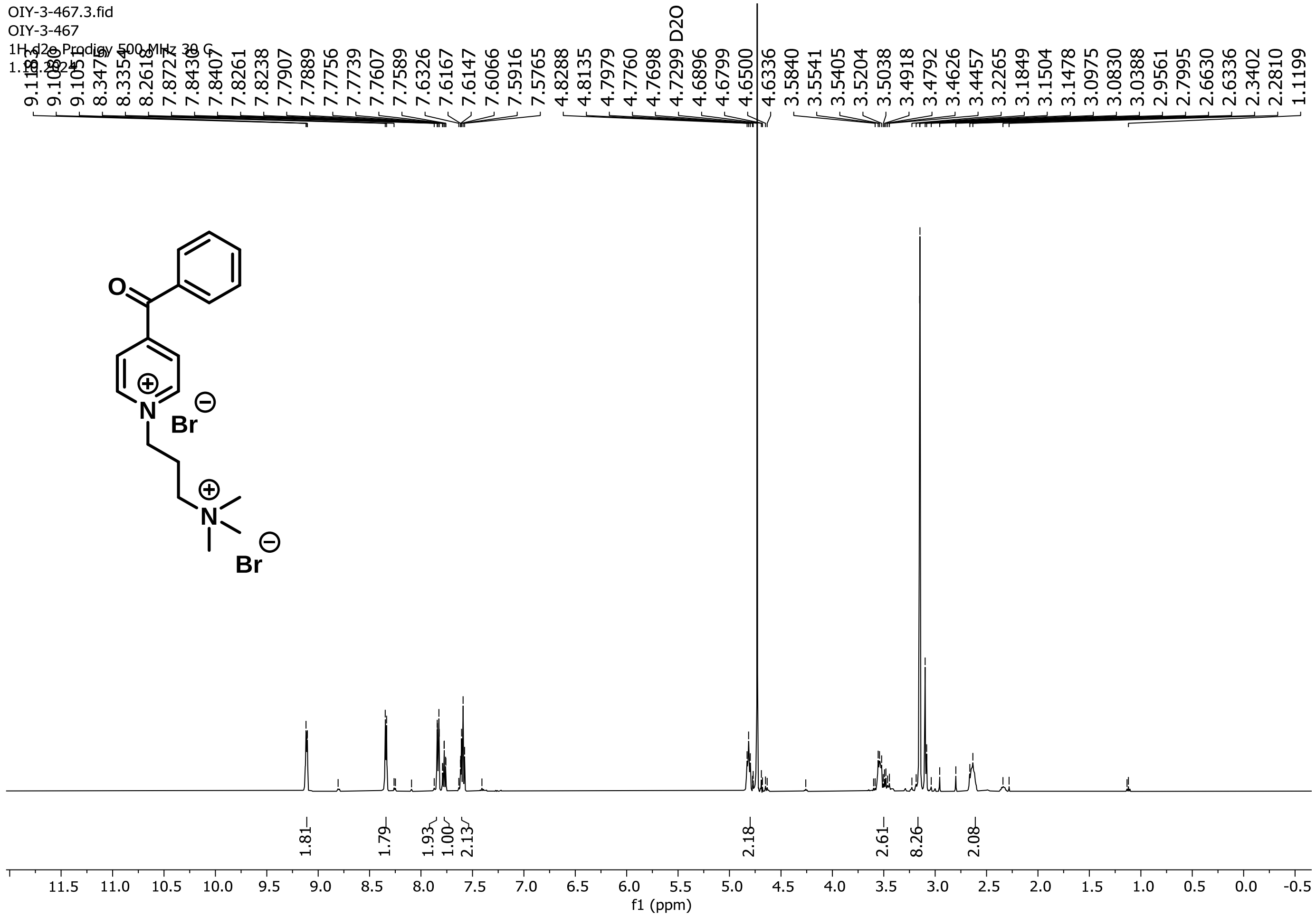

1.1.16 4-benzoyl-1-(3-(trimethylammonio)propyl)pyridin-1-ium bromide (**BenPy7**), ^{13}C NMR (D_2O, 125 MHz) spectrum

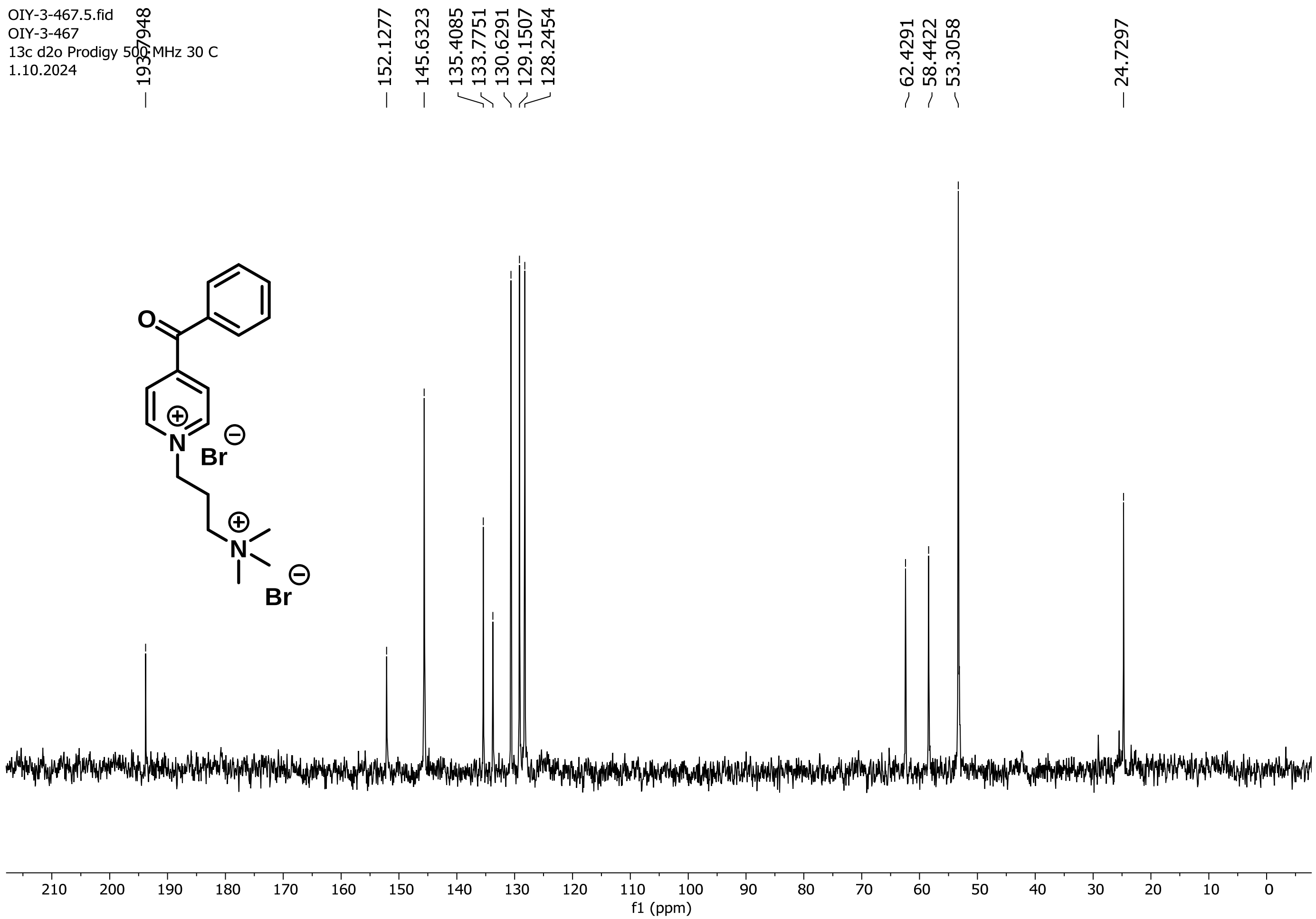

Supplement: Supplementary file 2 [file SC-OLF-D6SC04767J-s002.pdf]
